# Blooming of Unusual Cytochrome P450s by Tandem Duplication in the Pathogenic Fungus *Conidiobolus coronatus*

**DOI:** 10.3390/ijms19061711

**Published:** 2018-06-09

**Authors:** Mathula Lancelot Ngwenya, Wanping Chen, Albert Kotze Basson, Jabulani Siyabonga Shandu, Jae-Hyuk Yu, David R. Nelson, Khajamohiddin Syed

**Affiliations:** 1 Department of Biochemistry and Microbiology, Faculty of Science and Agriculture, University of Zululand, KwaDlangezwa 3886, South Africa; NgwenyaM@unizulu.ac.za (M.L.N.); BassonA@unizulu.ac.za (A.K.B.); ShanduJ@unizulu.ac.za (J.S.S.); 2 College of Food Science and Technology, Huazhong Agricultural University, Wuhan 430070, China; chenwanping@mail.hzau.edu.cn; 3 Department of Bacteriology, University of Wisconsin-Madison, 3155 MSB, 1550 Linden Drive, Madison, WI 53706, USA; jyu1@wisc.edu; 4 Department of Microbiology, Immunology and Biochemistry, University of Tennessee Health Science Center, Memphis, TN 38163, USA

**Keywords:** cytochrome P450 monooxygenase, *Conidiobolus coronatus*, drug resistance, entomopathogens, P450 blooms, rare and neglected diseases, rhinoentomophthoramycosis, tandem duplications

## Abstract

While the Zygomycete fungus *Conidiobolus coronatus* primarily infects insects, it can be pathogenic to mammals as well, including humans. High variability in the treatment of this fungal infection with currently available drugs, including azole drugs is a very common phenomenon. Azoles bind to the cytochrome P450 monooxygenases (P450s/CYP) including CYP51, a sterol 14-α-demethylase, inhibiting the synthesis of cell membrane ergosterol and thus leading to the elimination of infecting fungi. Despite P450’s role as a drug target, to date, no information on *C. coronatus* P450s has been reported. Genome-wide data mining has revealed the presence of 142 P450s grouped into 12 families and 21 subfamilies in *C. coronatus*. Except for CYP51, the remaining 11 P450 families are new (CYP5854-CYP5864). Despite having a large number of P450s among entomopathogenic fungi, *C. coronatus* has the lowest number of P450 families, which suggests blooming P450s. Further analysis has revealed that 79% of the same family P450s is tandemly positioned, suggesting that P450 tandem duplication led to the blooming of P450s. The results of this study; i.e., unravelling the *C. coronatus* P450 content, will certainly help in designing experiments to understand P450s’ role in *C. coronatus* physiology, including a highly variable response to azole drugs with respect to P450s.

## 1. Introduction

Cytochrome P450 monooxygenases (CYPs/P450s) are heme-thiolate proteins that are ubiquitously distributed in organisms belonging to different biological kingdoms [1]. P450s are well known for their stereo- and regio-specific enzymatic reactions [2] and thus play a key role in organisms’ physiology, both in primary and secondary metabolism [3]. Because of their important catalytic activities, P450s’ role in drug metabolism and drug discovery, in the generation of commercial products including antibiotics, in bioremediation, and in the production of biofuels has been explored [3].

Analysis of fungal species genomes has revealed the presence of a large number of P450s, with few exceptions. It is now well known that the number of P450s and diversity of P450s in a fungal species is largely affected by the fungal species’ lifestyle or their ecological niche [4,5,6,7]. This indicates that P450s play a key role in fungal species’ physiology in terms of their primary and secondary metabolism. Fungal P450s’ role in fungal species’ physiology and their biotechnological application has been thoroughly reviewed [8,9,10,11]. Among all fungal P450 applications, the use of P450s as drug targets against fungal pathogens is well explored [12,13] because of their binding affinity to azole drugs, which are widely used for treating fungal infections [14]. Most of the azole drugs bind and inhibit CYP51, a sterol 14α-demethylase, thus stopping the synthesis of cell membrane ergosterol, which is an indispensable component of fungal membranes [12]. Ergosterol depletion affects not only the structure of the membrane, but also several of its functions, including its hormone-like role that stimulates the growth and proliferation of fungal cells that eventually lead to the death of fungi [14,15,16]. Fungal pathogens were found to develop drug resistance [17] to anti-fungal drugs such as azole drugs and other drugs belonging to the classes of allylamine, echinocandin, nucleoside analog, and polyene [18]. Understanding the fungal pathogens’ physiology at the molecular level will help to unravel novel drug targets and develop novel drugs.

Among fungal pathogens, *Conidiobolus coronatus,* an entomopathogen (pathogen of insects) developed the ability to infect animals (horses, sheep, and dogs), including humans [19]. *C. coronatus* lives in soil and decaying matter, especially dead leaves [20], in addition to the feces of amphibians, sheep, insects, and horses [19]. *C. coronatus* has a worldwide distribution, especially in the tropical rain forests of Africa. It has been found in the United Kingdom [21], on the eastern coast of the United States [22], in India [23,24], the western region of Africa, and recently in South Africa [25].

*C. coronatus* causes conidiobolomycosis, an entomophthoramycosis in humans, and is considered a rare and neglected disease [19]. The definitive route of infection has not been established, but it is believed that *C. coronatus* enters the human body via inhalation of spores or from minor trauma [26]. Conidiobolomycosis is a disease of the nasal submucosa and paranasal sinuses, which slowly spreads to the nasal skin, glabella, cheek, upper lip, and pharynx. Rarely, contiguous lymph nodes may be involved [24,27,28,29,30]. Infection of the nasal and paranasal mucocutaneous tissue is known as rhinoentomophthoramycosis. Recently, *C. coronatus* infection in the vagina has been reported [22], indicating that this fungus is capable of infecting other sites of the human body as well.

*C. coronatus* is capable of producing mycotoxins that are known to be very toxic to insects [31,32]. This parasitic fungus will attack normal or stressed insects, gaining access by penetrating the host’s inner skin, and the host will eventually die because of tissue demolition, nutrient depreciation, and the production of toxins [33]. This entomopathogenic fungus has the potential of becoming a biological insect control, owing to its ability to infect and kill a varied range of insects [34].

In order to understand *C. coronatus*’s physiology at the molecular level, genome sequencing of this fungus has been carried out [35]. However, the genome sequencing study was focused on analyzing the evolution of cell wall digestion enzymes and no information on *C. coronatus* P450s has been presented [35]. Interestingly, conidiobolomycosis treatment employing amphotericin B, azoles and/or potassium iodide was found to be highly variable [19]. In a case study, a patient infected with *C. coronatus* showed no response when treated with amphotericin B; azoles (ketoconazole, fluconazole and itraconazole) and potassium iodide individually, and a combination of azole drug therapies were needed to treat the patient [36]. Sometimes treatment includes both the surgical removal of infected tissue and the use of different drugs [37]. High variability in susceptibility to azole drugs by *C. coronatus* was widely observed [19], including *C. coronatus* species that are resistant to azole drugs [38]. 

Since P450s were found to play a key role in organisms’ physiology, including serving as azole drug targets [12,14,15,16,17], it is necessary to unravel the P450 content of this fungus to be able to design experiments to understand P450s role in *C. coronatus* physiology and the variability in susceptibility of this fungus to azole drugs with respect to P450s.

## 2. Results and Discussion

### 2.1. C. coronatus Has the Highest Number of P450s among Entomopathogenic Fungi

**Figure 1:**
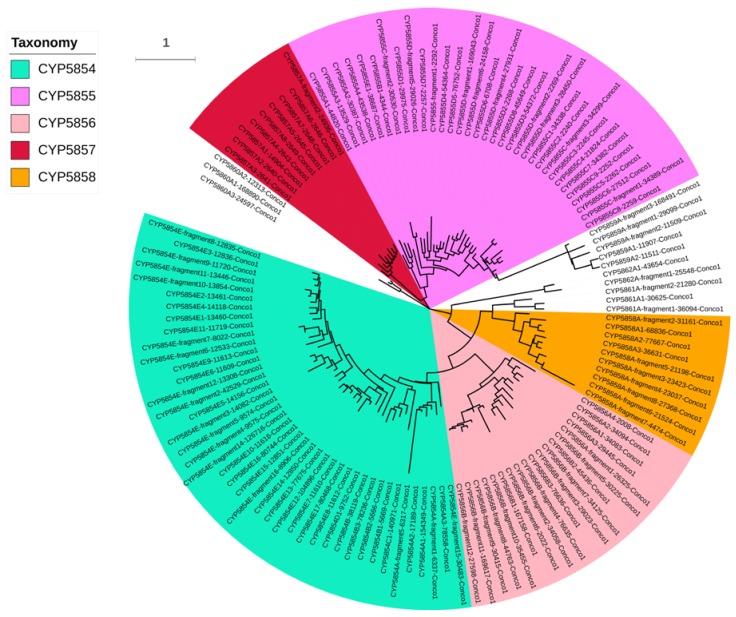
Phylogenetic analysis of *C. coronatus* P450s. P450 families bloomed in *C. coronatus* are highlighted in different colors. Bootstrap replications used to evaluate each node of the phylogenetic tree are presented in Figure S1.

Genome data mining, identification, and annotation of P450s in *C. coronatus* revealed the presence of 142 P450s in its genome (Figure 1 and Table 1). *C. coronatus* was found to have the highest number of P450s compared to other entomopathogenic and animal (including human) pathogenic fungi (Table 2).

### 2.2. Large Number of New P450s Found in C. coronatus

*C. coronatus* P450s can be grouped into 12 P450 families and 21 P450 subfamilies (Table 1). Except for CYP51, the most conserved family in fungi [43], the remaining P450 families are new. It is quite interesting that *C. coronatus*, apart from CYP51, does not share a P450 family with other entomopathogenic fungi listed in Table 2. Interestingly, *C. coronatus* lacks CYP61, the P450 involved in fungal membrane ergosterol synthesis [44], compared with pathogenic fungi listed in Table 2. Among 142 P450s, one P450 (protein ID: 71373) is not assigned to a P450 family because of a short amino acid sequence and low sequence identity to other P450s. In addition, one P450 (protein ID: 2292) belonging to the CYP5854 family has not been assigned to a subfamily because of a short amino acid sequence and low identity to other subfamilies. P450s that are not full-length (<300 amino acids) were indicated with word “fragments” after their P450 subfamily (Figure 1 and Tables S1 and S2). The new P450 families identified in *C. coronatus* include CYP5854-CYP5864 (Table 1). Despite having a large number of P450s, *C. coronatus* has the lowest number of P450 families compared to entomopathogenic and animal (including human) pathogenic fungi (Table 2), suggesting P450 blooms (a few families with many genes) in *C. coronatus*. Phylogenetic analysis revealed that CYP5859 P450s show a close relationship with CYP5855 P450s and seem to have evolved from CYP5855 P450s (Figure 1).

### 2.3. P450 Signature Motifs EXXR and CXG Are Conserved in C. coronatus P450s

**Figure 2:**
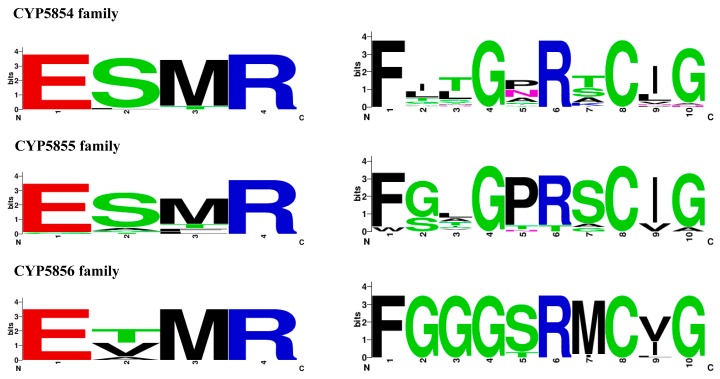
Analysis of amino acid patterns at the EXXR and CXG motifs in P450 families CYP5854-CYP5856. P450 protein sequences used to deduce EXXR and CXG signature sequences were presented in Table S4.

Among different motifs that are characteristic of P450s, two motifs, namely EXXR in the K-helix and FXXGXRXCXG (also known as CXG) in the heme-binding domain, are found to be conserved in P450s, with few exceptions [45,46,47,48]. These two motifs are largely explored in the identification of P450s across biological kingdoms [48]. Analysis of EXXR and CXG motifs in *C. coronatus* P450s revealed that all P450s have both signature motifs (Table S3). Only a few P450s that are named as fragments (as mentioned in Section 2.2) did not have one of these motifs (Table S3). Thus, these P450s named as fragments represent pseudo-P450s. Analysis revealed that *C. coronatus* P450s have highly conserved amino acids, such as glutamic acid (E) and cysteine (C), in the motifs EXXR and CXG, with the exception of CYP5857A4, which has glycine instead of glutamic acid in the EXXR motif (Table S3). Non-conservation of glutamic acid in EXXR motifs is not new and has been reported for other P450s as well [48]. Analysis of amino acid patterns for the P450 families, CYP5854-CYP5856, revealed family-specific amino acid patterns at the EXXR and CXG motifs (Figure 2). CYP5854 and CYP5855 have the same E-S-M-R amino acid pattern, whereas CYP5856 has an E-T/V-M-R amino acid pattern in the EXXR motif (Figure 2). Comparative analysis with P450s across biological kingdoms [48,49] revealed that the CYP5854 and CYP5855 families’ EXXR amino acid pattern matched that of the CYP94 family, whereas the CYP5856 family EXXR amino acid patterns partially matched those of the CYP53, CYP92, and CYP176 P450 families, indicating a possible evolutionary relation between the families. Contrary to the EXXR motifs, the amino acid patterns at the CXG motifs of the CYP5854-CYP5856 families did not match any P450s [48,49]. This strongly supports the hypothesis that each P450 family has its unique signature amino acid patterns at the EXXR and CXG motifs [48].

### 2.4. P450 Blooming in C. coronatus

**Figure 3:**
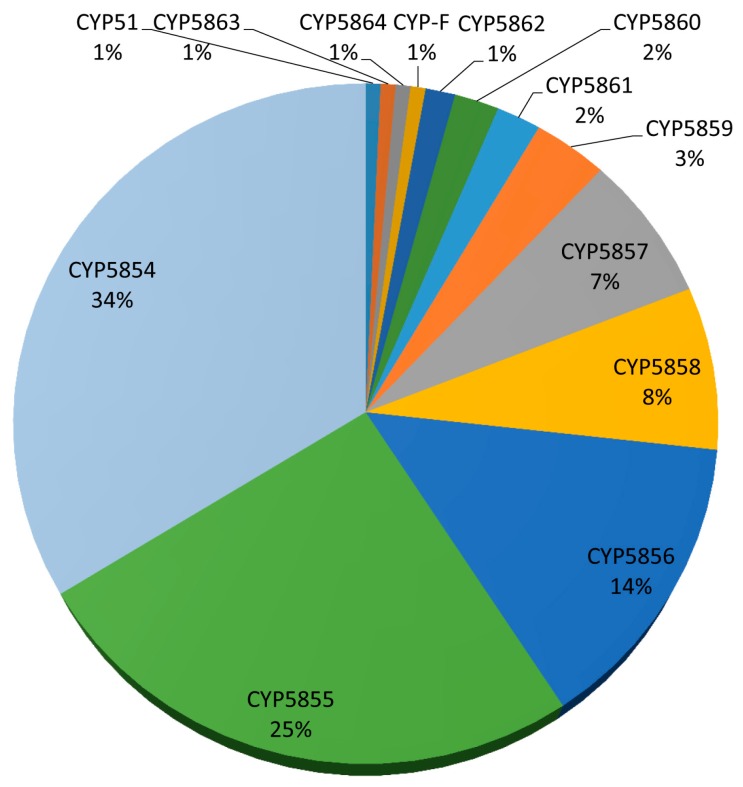
Comparative analysis of P450 family members in *C. coronatus*. The P450 family name and percentage in the total number of P450s are shown in the figure. The total number of P450s in a P450 family is presented in Table 1.

Comparative analysis of P450s revealed that among 11 P450 families, five P450 families contributed 88% of P450s to the total number of P450s in *C. coronatus* (Figure 2 and Table 1). This indicates that the five P450 families, namely CYP5854, CYP5855, CYP5856, CYP5858, and CYP5857, are highly bloomed in *C. coronatus*. The highest number of member P450s were found in CYP5854 (48 P450s) followed by CYP5855 (36 P450s), CYP5856 (20 P450s), CYP5858 (11 P450s), and CYP5857 (10 P450s) (Figure 3). Blooming of certain P450 families in an organism indicates that these family members play a key role in an organism’s physiology and thus they are expanded [4,50]. In some Basidiomycetes, P450 families were found to be expanded in order to help those organisms to colonize on wood and wood-derived materials [4]. At present the reason or need for blooming of these five P450 families in *C. coronatus* is not clear. It is noteworthy that none of the P450 families is expanded in other entomopathogenic fungi listed in Table 2.

### 2.5. Extensive Tandem Duplications Led to P450 Blooming in C. coronatus

**Figure 4:**
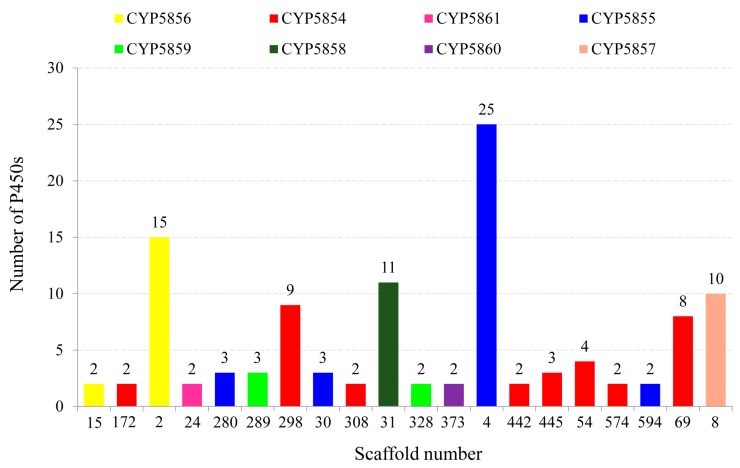
Analysis of tandem duplications of P450s in *C. coronatus*. The numbers next to bars indicate the number of P450s located on the scaffold.

P450 blooming is only possible when certain P450s belong to the same P450 family duplicated in large numbers in an organism [50]. The duplicated P450s (paralogs) can be identified by observing their location in the genome, intron-exon organization, and percentage identity at protein level, as described elsewhere [4,6,13]. Tandem arrangement of P450s (P450s located one behind the other) belonging to the same family is a good indication of P450 duplications. Analysis of the *C. coronatus* genome revealed that 79% of P450s (112 P450s) belonging to the same P450 family are tandemly located (Figure 4 and Table S5).

As shown in Figure 4, the five P450 families bloomed in *C. coronatus* were found to have the highest number of tandemly duplicated P450s. This indicates that P450 tandem duplications led to the blooming of these five P450 families in *C. coronatus*. The CYP5855 family was found to have the highest number of tandemly duplicated P450s (33 P450s), followed by the CYP5844 family with 32 P450s, the CYP5856 family with 17 P450s, the CYP5858 family with 11 P450s, and the CYP5857 family with 10 P450s. The number of tandemly duplicated P450s in the respective families was shown in Figure 4. In addition to the five P450 families bloomed in *C. coronatus*, three P450 families were found to have P450s that are tandemly duplicated, namely CYP5861 (2 P450s), CYP5859 (5 P450s) and CYP5860 (2 P450s) (Figure 4 and Table S5). It is interesting to note that P450s that are tandemly located on different scaffolds (portion of the genome sequence reconstructed from end-sequenced whole-genome shotgun clones) belong to the same subfamily (Table S5). This strongly indicates that these P450s indeed evolved by tandem duplications.

### 2.6. C. coronatus P450s Have Same Gene Structure

**Figure 5:**
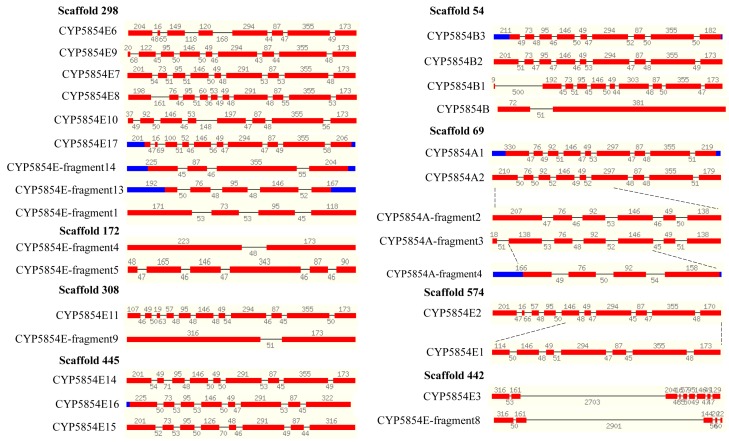
Gene-structure analysis of P450s in the CYP5854 family. Each P450 gene structure is presented with the P450 name, exons (red color bars), and introns (gap between bars). The size of the exons (number at the top of the red bars) and introns (number in the gaps) is also shown in the figure. Some P450s’ evolution was deduced from other P450s and their origin is indicated with dotted lines. Blue regions indicate an untranslated DNA region. The location of the respective P450s is indicated with their scaffold number.

**Figure 6:**
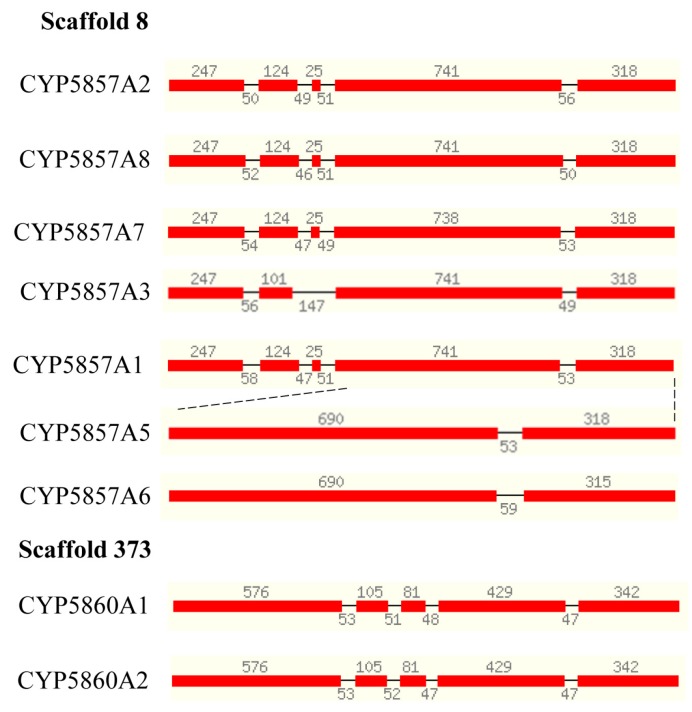
Gene structure analysis of P450s in CYP5857 and CYP5860 family. Each P450 gene structure is presented with the P450 name, exons (red color bars) and introns (gap between bars). The size of exons (number at the top of the red bars) and introns (number in the gaps) is shown in the figure. Some P450s’ evolution was deduced from other P450s and their origin is indicated with dotted lines. The location of the respective P450s is indicated with their scaffold number.

Another characteristic of tandemly duplicated genes is that they have the same gene structure; i.e., the same number and size of introns and exons. Fungal P450s with the same gene structure have previously been reported and the authors suggested that this is a typical characteristic of gene duplications [4,6,13]. In this study, gene structure analysis was carried out for P450 families such as CYP5854, CYP5857, and CYP5860 (Figure 5 and Figure 6), since most of the members in these families are full-length P450s.

As shown in Figure 5, the size of the exons in CYP5854 P450s is conserved. The number of introns and exons in some P450s is also highly conserved. P450s that are short contain the same size exons as full-length P450s, indicating their origin from full-length P450s. This was clearly observed for some CYP5854 P450s such as CYP5854-fragment2, which originated from CYP5854A2, CYP5854A2-fragment4, which originated from CYP5854-fragment3 and CYP5854E1, which originated from CYP5854E2 (Figure 5).

The phenomenon of the same gene structure was also observed in CYP5857 and CYP5860 P450s (Figure 6). The origin of P450s from other P450s in the CYP5857 family was observed as well, where CYP5857A5 P450s seem to have originated from CYP5857A1 (Figure 6). The two members of the CYP5860 family have the same gene structure (Figure 6).

### 2.7. C. coronatus Has the Lowest P450 Diversity among Entomopathogenic Fungi

**Figure 7:**
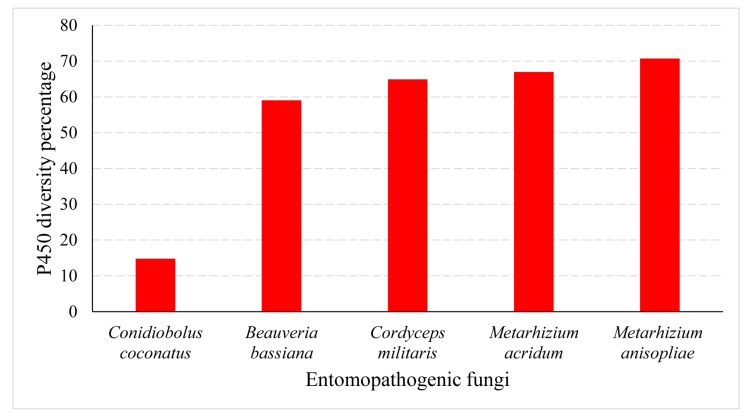
Comparative analysis of P450 diversity percentage among entomopathogenic fungi. The corresponding values are presented in Table S6.

Another characteristic of tandem duplication of P450s is that organisms have the lowest P450 diversity percentage [7,49,51]. The P450 diversity percentage is a good indication of P450 family blooming. The lowest P450 diversity means that certain P450 families are bloomed in an organism. Analysis of the P450 diversity percentage revealed that *C. coronatus* has only 8% P450 diversity, despite having a large number of P450s in its genome (Figure 7).

Comparative analysis of the P450 diversity percentage with other entomopathogenic fungi revealed that *C. coronatus* has the lowest diversity (Figure 7). This further indicates that extensive P450 duplication led to blooming of certain P450 families and thus the lowest P450 diversity percentage.

### 2.8. Functional Prediction of C. coronatus P450s

Except for CYP51 P450, all P450s in *C. coronatus* are new, implying that functional analysis based on characterized homologs is not possible. However, as indicated earlier, CYP51 of *C. coronatus* can be predicted to be involved in ergosterol biosynthesis based on the characterized homolog P450 [43]. It is interesting to see the role of bloomed P450s in *C. coronatus*, as these families are unique to this fungus compared to other entomopathogenic fungi. It is interesting to note that another P450, CYP61, involved in fungal membrane ergosterol synthesis [44] is not present in *C. coronatus*.

## 3. Materials and Methods

### 3.1. Genome Data Mining for P450s

The genome sequence of *C. coronatus* NRRL28638 v1.0 has been published [35] and is available at the JGI MycoCosm database (Available online: https://genome.jgi.doe.gov/Conco1/Conco1.home.html), which was data-mined for P450s. The data mining of P450s was carried out as described elsewhere [4,6,52], with slight modifications. Briefly, the *C. coronatus* genome was mined for P450s using InterPro code “IPR001128”. The hit protein sequences were downloaded and subjected to the NCBI *Batch* Web CD-Search Tool [53]. Proteins that grouped under the P450 superfamily were selected for further analysis.

### 3.2. Annotation of P450s

The above selected P450 proteins were subjected to BLAST analysis against all named fungal P450s at the Cytochrome P450 Homepage [54] to identify a homolog P450. The name of the homolog P450 and percentage of identity to the homolog P450 were recorded (Table S1). Based on the percentage identity to homologous P450s, a family and subfamily were assigned to *C. coronatus* P450s. This was done by following the rules set by the International P450 Nomenclature Committee [55,56,57]; i.e., >40% identity for a family and >55% identity for a subfamily. *C. coronatus* P450s that had <40% and <55% identity to the named fungal P450s were assigned to new families and new subfamilies, respectively (Table S1). Percentage identity among *C. coronatus* P450s was also used to assign P450 families and subfamilies. Information on homolog P450s that are used in the naming of *C. coronatus* P450s was listed in Table S1 along with the *C. coronatus* P450s identified and annotated in this study. *C. coronatus* P450 protein sequences along with their amino acid length are presented in Table S2.

### 3.3. Phylogenetic Analysis of P450s

The phylogenetic tree of *C. coronatus* P450s was constructed as described previously [51], with slight modifications. Briefly, the protein sequences were aligned by MUSCLE embedded in MEGA 7 [58]. Then, the best-fit substitution model for alignment was determined by the IQ-TREE web server (Available online: http://iqtree.cibiv.univie.ac.at/) [59]. Finally, the tree was constructed in MEGA7 by the maximum likelihood method, along with the best-fit substitution model and 100 bootstrap replications [60]. iTOL was used to view and highlight the tree [61].

### 3.4. Analysis of EXXR and CXG Motifs

An analysis of EXXR and CXG motifs in *C. coronatus* P450s was carried out as described elsewhere [48]. Briefly, a multiple sequence alignment of P450s was carried out using Clustal Omega [62] and then a manual search was performed for the presence of EXXR and CXG motifs (Table S3). The WebLogo for EXXR and CXG motifs for the P450 families, CYP5854-CYP5856, was deduced as described elsewhere [48] following the WebLogo program [63]. WebLogos were deduced using the default parameter with the amino acid sequence type. The criteria for the selection of P450 families for EXXR and CXG amino acid pattern analysis are based on the number of P450s in a P450 family [49]. Three P450 families, CYP5854-CYP5856, have more than 15 P450s for analysis (Table S4) compared to other new P450 families, thus they qualify for EXXR and CXG motif analysis.

### 3.5. Identification of Tandemly Duplicated P450s

Tandem P450 gene duplications were assessed as described elsewhere [4,6]. Briefly, the physical location of P450 genes, such as scaffold number, start and end point of the P450 gene on the DNA strand, was recorded by scanning the *C. coronatus* genome using the P450 protein ID (Table S5). P450s that are tandemly arranged and belong to the same family are regarded as duplicated P450s. The percentage P450 duplications are calculated as the percentage of P450s duplicated in the total number of P450s.

### 3.6. Gene Structure Analysis

Gene structure analysis of P450s was carried out as described elsewhere [4,6,7,13]. Briefly, the *C. coronatus* genome was mined using P450 protein ID. P450 gene structure graphics downloaded from the *C. coronatus* genome database were aligned in such a way that exons with the same size would align together. The length of exons was noted as an indication of possible gene duplication, if P450s showed conservation in the size of exons. P450s whose gene structures contained the same sizes of exons or introns were presented in the figures.

### 3.7. P450 Diversity Percentage Analysis

P450 diversity percentage analysis was carried out as described elsewhere [7,49,51]. Briefly, the P450 diversity percentage in *C. coronatus* was measured as a percentage contribution of the number of P450 families in the total number of P450s.

### 3.8. Comparative Analysis of P450s

Entomopathogenic and animal (including human) pathogenic fungi P450s were retrieved from published articles [7,39,40,41,42] and used for comparative analysis with *C. coronatus* P450s.

## 4. Conclusions

This study is the first of its kind on an *in silico* analysis of P450s in *C. coronatus*. Except for CYP51 P450, a conserved P450 in fungi and a primary target of azole drugs, all P450s were found to be new in *C. coronatus*. Unprecedented blooming of novel P450s in *C. coronatus* compared to other fungal species was observed. It would be interesting to observe the role of bloomed P450s in *C. coronatus* physiology, considering this fungus has a broad host range and is capable of infecting humans and other animals, in addition to insects.

## Figures and Tables

**Table 1:** Annotation (family and subfamily assignment) of *C. coronatus* P450s.

P450 Family	P450 Subfamily		No. of P450s in a Family
	Subfamily Name	No. of P450s	
CYP51	F	1	1
CYP5854	A	8	48
	B	4	
	C	1	
	D	1	
	E	34	
CYP5855 *	A	5	36
	B	2	
	C	12	
	D	15	
	E	1	
		1	
CYP5856	A	5	20
	B	15	
CYP5857	A	10	10
CYP5858	A	11	11
CYP5859	A	5	5
CYP5860	A	3	3
CYP5861	A	3	3
CYP5862	A	2	2
CYP5863	A	1	1
CYP5864	A	1	1
CYP-fragment ^#^	-	1	1
12 Families	21 subfamilies	142	142

* Belonging to the same family but not assigned to a subfamily because of short amino acid sequence; - not applicable; ^#^ not assigned to family because of short amino acid sequences (284 amino acids) and only showed 24% identity to named P450s.

**Table 2:** Comparative P450 analysis in entomopathogenic and animal (including human) pathogenic fungi.

Fungus	Host	P450 Count	No. of P450 Families	Reference
**Entomopathogenic fungus**				
*Beauveria bassiana*	Arthropods (termites, thrips, whiteflies, aphids, and beetles)	83	49	[39]
*Cordyceps militaris*	Butterflies and caterpillars	57	37	[40]
*Metarhizium acridum*	≥200 insects	100	67	[41]
*Metarhizium anisopliae* (formerly *Metarhizium robertsii*)	Locusts	123	87	[41]
**Animal including human pathogen**				
*Sporothrix schenckii*	Humans and other animals (cats, dogs, rodents, squirrels, horses, and birds)	40	32	[7,42]
**Animals (including humans) and entomopathogenic fungus**				
*Conidiobolus coronatus*	Insects, humans, and other animals (horses, sheep and dogs)	142	12	This work

## Supplementary Materials

Supplementary materials can be found at http://www.mdpi.com/1422-0067/19/6/1711/s1.
