# Supplementary material for: Blooming of Unusual Cytochrome P450s by Tandem Duplication in the Pathogenic Fungus Conidiobolus coronatus
Source: Int J Mol Sci. 2018 Jun 9;19(6):1711. doi: 10.3390/ijms19061711 (PMC6032100; doi:10.3390/ijms19061711)
Supplement: Supplementary file 1 [file ijms-19-01711-s001.pdf]

Supplementary materials: Blooming of unusual cytochrome P450s by tandem duplication in the pathogenic fungus *Conidiobolus coronatus*

Table S1. Annotation of *C. coronatus* P450s. *C. coronatus* P450 names (assigned in this study), their protein IDs and percentage identity to reference P450s (from Cytochrome P450 Homepage that are used to name *C. coronatus* P450s) were shown in the table.

Conidiobolus coronatus P450s		Reference P450s from Cytochrome P450 Homepage	
P450 name	Protein ID	Percentage identity	Named P450
CYP51F14	41890	44.06	CYP51F1
CYP5854A1	154349	34.02	CYP509F1
CYP5854A2	17189	34.16	CYP509F1
CYP5854A3	78558	37.74	CYP509F1
CYP5854A-fragment1	6337	37.04	CYP5365A1
CYP5854A-fragment2	6332	31.25	CYP509F2
CYP5854A-fragment3	6331	31.37	CYP509F2
CYP5854A-fragment4	78559	29.91	CYP509A1
CYP5854A-fragment5	6317	35.82	CYP567E3
CYP5854B1	5669	31	CYP509F1
CYP5854B2	5666	31.73	CYP509F1
CYP5854B3	78236	33.95	CYP509C8
CYP5854B-fragment1	38119	39.6	CYP509F3
CYP5854C1	140971	35.75	CYP509B2
CYP5854D1	9752	31.23	CYP509C10
CYP5854E1	13460	33.67	CYP509C10
CYP5854E2	13461	31.91	CYP509B2
CYP5854E3	12836	36.42	CYP509C12
CYP5854E4	14118	32.02	CYP509H1
CYP5854E5	14156	28.09	CYP509C10
CYP5854E6	11609	28.32	CYP509C1
CYP5854E7	11610	34.15	CYP509B2
CYP5854E8	11612	31.04	CYP509C10
CYP5854E9	11613	31.34	CYP509C10
CYP5854E10	11616	32.25	CYP509B2

CYP5854E11	11719	30.62	CYP509C10
CYP5854E12	104896	31.45	CYP509C10
CYP5854E13	77675	38.18	CYP509B1
CYP5854E14	12850	32.53	CYP509B2
CYP5854E15	12851	30.47	CYP509C10
CYP5854E16	80744	32.16	CYP509B1
CYP5854E17	80468	33.63	CYP509B1
CYP5854E-fragment1	11608	27.85	CYP509C10
CYP5854E-fragment2	42529	37.62	CYP509F1
CYP5854E-fragment3	14082	39.1	CYP509C10
CYP5854E-fragment4	9575	36.09	CYP509F3
CYP5854E-fragment5	9574	33.45	CYP509F3
CYP5854E-fragment6	12533	37.84	CYP509L1
CYP5854E-fragment7	8022	43.1	CYP509D1
CYP5854E-fragment8	12835	36.73	CYP509C12
CYP5854E-fragment9	11720	36.54	CYP509C9
CYP5854E-fragment10	13854	30.93	CYP509H1
CYP5854E-fragment11	13446	35.88	CYP509H1
CYP5854E-fragment12	13308	31.99	CYP509H1
CYP5854E-fragment13	126715	32.26	CYP509A1
CYP5854E-fragment14	126716	33.86	CYP509H1
CYP5854E-fragment15	30483	45	CYP509F1
CYP5854E-fragment16	8906	41.63	CYP509C9
CYP5854E-fragment17	6815	31.11	CYP509A1
CYP5855A1	44820	28.5	CYP5081C2
CYP5855A2	30387	29.56	CYP5269A1
CYP5855A3	13529	26.87	CYP5081C2
CYP5855A4	43538	30.77	CYP5104B1
CYP5855A-fragment1	11397	27.14	CYP5313A5
CYP5855B1	4344	25.78	CYP5313A5
CYP5855B-fragment1	4348	27.53	CYP5313A5
CYP5855C1	34338	29.82	CYP53A29
CYP5855C2	2240	28.3	CYP5081A1
CYP5855C3	2245	29.27	CYP65AM1
CYP5855C4	21824	27.65	CYP65AM1
CYP5855C5	2262	27.65	CYP5313A5
CYP5855C6	27512	31.03	CYP567E5
CYP5855C7	34382	31.29	CYP548D2
CYP5855C8	2259	28.83	CYP5313A5

CYP5855C9	2252	29.75	CYP65CK3
CYP5855C-fragment1	34389	35.27	CYP65CC6
CYP5855C-fragment2	30530	39.02	CYP5142U1
CYP5855C-fragment3	34299	35.89	CYP65CC1
CYP5855D1	29975	28.74	CYP5081A1
CYP5855D2	2268	26.96	CYP5081A1
CYP5855D3	54372	27.96	CYP5313A4
CYP5855D4	54364	26.94	CYP5081A1
CYP5855D5	76752	31.27	CYP548D2
CYP5855D6	6708	27.22	CYP5081A1
CYP5855D7	2257	29.31	CYP5081A1
CYP5855D8	45649	28.26	CYP5081A1
CYP5855D-fragment1	169043	40.56	CYP53K2
CYP5855D-fragment2	2269	39.68	CYP53K2
CYP5855D-fragment3	28450	43.33	CYP53J1
CYP5855D-fragment4	27931	38.82	CYP677A2
CYP5855D-fragment5	29026	33	CYP5081A1
CYP5855D-fragment6	24158	46.15	CYP5142J5
CYP5855D-fragment7	2254	31.84	CYP5313A6
CYP5855E1	38687	30.07	CYP5313A6
CYP5855-fragment1	2292	47.92	CYP5136A20
CYP5856A1	34083	33.79	CYP509F3
CYP5856A2	34094	34.38	CYP509F3
CYP5856A3	29445	37.72	CYP509F1
CYP5856A4	2008	32.6	CYP509F1
CYP5856A-fragment1	26325	46.32	CYP509F3
CYP5856B1	167158	34.88	CYP509C1
CYP5856B2	45436	35.04	CYP509C9
CYP5856B3	76636	35.62	CYP509F3
CYP5856B-fragment1	2011	32.66	CYP509C5
CYP5856B-fragment2	34058	39.42	CYP509E1
CYP5856B-fragment3	29023	46.83	CYP509C10
CYP5856B-fragment4	76635	38.83	CYP509E1
CYP5856B-fragment5	30225	43.46	CYP509E1
CYP5856B-fragment6	2022	28.74	CYP509C11
CYP5856B-fragment7	34125	41.15	CYP509C12
CYP5856B-fragment8	44763	39.64	CYP509E1
CYP5856B-fragment9	30415	38.66	CYP509E1
CYP5856B-fragment10	35455	38.74	CYP509E1

CYP5856B-fragment11	169617	38.29	CYP509E1
CYP5856B-fragment12	27598	41.49	CYP509C11
CYP5857A1	14904	29.7	CYP5104B1
CYP5857A2	2640	29.86	CYP548A25
CYP5857A3	2641	30.89	CYP5142R1
CYP5857A4	2643	34.7	CYP630B19
CYP5857A5	2645	33.13	193681(CYP5142C)
CYP5857A6	2646	31.86	CYP65CC5
CYP5857A7	2648	29.45	CYP627A3
CYP5857A8	2649	30.31	CYP65EB2
CYP5857A-fragment1	12733	31.05	CYP684A8
CYP5857A-fragment2	24936	43.5	CYP677A2
CYP5858A1	68836	31.43	CYP509F2
CYP5858A2	77667	33.48	CYP509F2
CYP5858A3	36631	30.26	CYP509B1
CYP5858A-fragment1	68816	28.93	CYP509F3
CYP5858A-fragment2	31161	37.65	CYP509F3
CYP5858A-fragment3	23423	38.92	CYP509B2
CYP5858A-fragment4	23037	44.58	CYP509B2
CYP5858A-fragment5	21198	39.76	CYP509B2
CYP5858A-fragment6	21524	38.92	CYP509C11
CYP5858A-fragment7	4474	34.82	CYP509B1
CYP5858A-fragment8	27368	42.86	CYP509G1
CYP5859A1	11907	28.02	CYP684A1
CYP5859A2	11511	27.78	CYP684A1
CYP5859A-fragment1	29099	38.67	CYP677A2
CYP5859A-fragment2	11509	37.04	CYP53B4
CYP5859A-fragment3	168491	37.25	CYP53B4
CYP5860A1	168890	32.47	CYP5313A5
CYP5860A2	12313	30.33	CYP5313A5
CYP5860A3	24597	31.45	CYP684B1
CYP5861A1	30625	30.98	CYP509H1
CYP5861A-fragment1	36094	40.1	CYP509H1
CYP5861A-fragment2	21280	42.38	CYP509B1
CYP5862A1	43654	30.2	CYP5213B1
CYP5862A-fragment1	25548	33.61	CYP54D1
CYP5863A1	2687	25.53	CYP5148B14
CYP5864A1	18764	29.96	CYP65A4
CYP-fragment	71373	24.04	CYP509E1

Table S2. *C. coronatus* P450 protein sequences were presented along with their size and assigned name. Abbreviation: AA, amino acid.

AA length	P450	P450 sequence
335	CYP51F14	ILHDMAKLIIKTASGCLLGHEIRAKLDDSVADIYHDLDGGFTPMQFMFEWLPLPSYYKRDAAHKKMSDLFYDIVQERRKSEKQNDDVVDALMRNSYKDGTMMTDRQVAHMMIALLMGGQHTSSTTSSWTLLLLAQNPEIQEELLKEQKEVFGEDLKDLTLDGLKKCTLLESCIKETLRMRPPIINVMRYVKQNCEIPNTSYVIPQGYYVMSAPIITQLDEKCFPNAESYLPKRWLDPSIAAGTNKTPTENVIEEHVWATMNSSSARSSYLPFGAGRHRCVGEQFAYMQISTIISVILRNYRIKLNTEAGFPNINFATLICSPLDPNLIEYEPRN*
496	CYP5854A1	MYLIAILIVAITYLVLKWRNFYKKPKEWEHIPSVSLWGELYRLFTDPQPIDVAFEKYTLPILDDFGLATFCDLAKGYTLFVTNPGALKEIFKNTDIFVKEPFAEKNETLFIEHFGEQQVVNANGEDWKRYRKIMNPIFNQSWKPELFGECFNQVIEEWDRLEGKDILIHDQIQRMTLDVMGKAFFDFDFESVKNPESKLYKIYHDITSGLFSNILYLVLPVLDHIPYLKRYDLHAKVAEYNKFVDDLIESRKKQILNGEAHKDNLLTQFVQASMPSSEKDAVMTDRELRDNLKIFFVAGHDTTANTITAIIYYLSRYPEVQDKLRSEIIEVMGEPECVRVPTVDELKQMNYLNMVIKESTRCMSAAPAAQRRTTKSFTLSNGVEIPEGKTVTLHMWGLFHNPKYFPEPNKFDPERFRDPNCEANKSWIPFITGARTCIGMSFSLMEQRVTLAMLLQTYTFKISPGHPDYERLRLPVMGFPKPTDFKVDLIRRIPN*
497	CYP5854A2	MMYLIAASVVVVTYFTLKWLNIYKKPKEWKYIPTISALEGLYRAFTNPVPIDVDFEKHTLPYIDDNGMTTICDLFSGYSLFVTDPDSLKQILKNTDIFVKEPFAEKNEALFKEHFGVQQVVSANGEDWKRMRKVMNPIFNRSWKPELFGECFNQVIEEWDKLEGKDILIHDQIQRMTLDVMGKAFFDFDFEAVRNPESKLYKLYHDITNGLFKDIAYLIFPILDHIPYLKRYALHAKVAEYNLFVENLIERRRQEILDGKANNDNLLTQFVQASMPTDKEEAVLSDRELRDNLKIFFVAGHDTTANTISAIIYYLARYPDVQAKLRSEIIEIMGESDSVRVPTVDQLKQMDYLNMVIKESTRCMTTAAVLSRRAAKPFTFSNGVQVPEGRMVNLHMWGLFHNPKYFPEPNKFDPERFRDPNCEANKSWIPFLTGSRTCIGMSFSLMEQRVALSMLVQAYIFQINSDHPDYEKLRLPVFGFPRPEDFKVDLIRRFTN*
317	CYP5854A3	MGKAFFDFDFQAVKNSKSELYQLYHNIVNGIFKDVSYLLFPIIDHIPFFKRYELHKKVAQYNEFVDNLVDCRKKEILEGKANKDNLLTQFAQASVPLDGKDPLLTPRELRDNLKIFFIAGHDTTANTITAIIYYLARYPEIQEKLRAEVIEIMGKSDKTLIPNNEQLKKMEYLNMIIKETIRIMTTAAVLDRRADKPFTFSNGVHVPVGKIVSIHMWGLLHSPKYYPNPDKFDPERFRDPNCEANKNWMPFLIGARTCIGMSFSLMEQRVALAMLVQTYTFEITPENPDYEKLRIVTFGFPRARDFHVDLVRINQS*
53	CYP5854A-fragment1	LMEQRVALVMLVQAYTFQIPADHPDYDKLRLSLFGFPRPRDFKVDLIRRVPN*
236	CYP5854A-fragment2	MYLIAITLIIITYYSYKWYNFYKLPEEWRDLPSIPFWKRLHRMLTKPVPINIAFTKHTLPKMEKLGIAVSCGIDKGQTLFISDPECLKQIMKNPDVFVKIPFAVENHTVLREHFGVQQVVTANGEDWRRMRKVMNPMFNQSWKPELFGECFNQVIEEWDKLEGKNILVQDQIQRMTLDVMGKAFFDFDFEAVKNPESELYQLYHNVVSGLFKNIAYIIFPILDHIPFMKRHELYNN
219	CYP5854A-fragment3	MYLVTAIPEVWKGIPVIPFWERIHRTLTKPVPIDVSFSKYMLPFMQKLGIAVYCSVNKGYTLFISDPECLKQVMSNIDVFIKMPFVGKNHTLLRDYFGAQQVVATNGEDWKRMRKVMNPMFNQSWKPELFGECFNQVIEEWDKLEGKNVLIHDQIQRMTLDVMGKAFFDFDFEAVKNPESELYQLYHNLVSGLYKKIIYRLFPILDRIPYLKRYELFDR
134	CYP5854A-fragment4	MLTNPAPMDTVFSNNILPYTEKIGIAVNLTLDRGQTLYVTDPECLKQIMKNPDIFIKIPFGEDNETLVREYFGVQQVVTTNGEDWKRMRNVMNPIFNQSWKPELFGGCRFYQVIEEWDKLEGKNIDIHDQIQR*
127	CYP5854A-fragment5	MPKAMNSAFNKTWKPELFEECFNQAVEEWYKLEGKKFSFMIKFKAPEYNIFADSLIKRRKADKDSLFTKFVQASISSNSQEAAVSTRELRLENMFVTGQKTTANIIIATIYYLSRYPEIQENCILK*
494	CYP5854B1	MNCFLIPLLSIYSGYKLYKILKCPPELQNIPSLDLSTLLKISTTKDGFDEELKKHVGPLLDKYGIVKYFSHHGWSVIISDPQLTKLVFNNSDIFQKDTNSAINSNPHAVKFFGKQQIVNTNGEDWKRMRKLMNPIFHKTWPIDKLSSCARDLIDSWSLTSGENVEIHENIQKLTLDVLGQAVFNMDFECIKNPQSKLYNQYHEISTQLFANLSYLFFPVLDKLPYFKRPELYKQIDSYDEFVKQMVALKKEELIENPNLAGKDDLLSKMLLSSMQADDENSKMTEKEIIDNLKSFFIAGHDTTSNTLTATLYYLARYPEIQDKLRSEILQVMNNPQVLTNPTVDQLKQMEYLNLVIKESMRAMATVSVLERVCTSEFQLTDSIKLTKNTPVFLMMYQVHQNPRYFSNPEKFDPERFRDPASVESKNWQPFITGPRACIGMTLSLMEQRISLVLLLQKFKFYIKESNPDYKKLRLNSSGILRPRDLHLDFIKLI*
491	CYP5854B2	MYEWILSLLSIYSGYKIYKIFKCPPELAHIPRLDILTIFKIASTKDPFDVEMAKNVGPLLDKHGVVRWFSQHGWSILVADPQLTKVIFNNPDIFEKDSNSAINANPHAIKFFGKEQIVNVNGEDWKRMRKLMNPIFHQTWPIQTLSQCTRDVIDSWSQTDGKGIDIRDNIQKMTLDVLGHTVFNMDFESVKNPDSKLYRIYHTASSELMGKLAYLFFPILEKLPFFKRPELHKQIDDYDNFVKELIVKKRAELKESPEIEKNDLLSKLIMASMEDGKALMSEREIRDNLKSIFIAGHDTTSNTLTTTLYYLARYPEIQDKLRAEILGVMNNPQVLTNPTLEQLKQMDYLNMVIKESMRIMATASVLERVATKPFQLTNSLYIPARTAVFVMLWKVHHQSEYFAKPEVFDPERFRDPASVESKNWQPFITGPRACIGMTLSLIEQRISLVLLLQKFYFEIDPSNPDYDRLRLNSSGMLCPKDLHLDIRQRI*
451	CYP5854B3	MGASKDAFDDEMAKHMGPMLDKHGIVRYFGPHGWSIIIADPQLAKIIYNNSDVFYKNTNSVINLNPHSQKFFGKDQIVNINGEDWKRMRKLMNPIFHQTWPIDTLSKCTRDVIDIWSQTDGLNVEVHDNIQKLTLDVLGHTVFNTDFESVKNPDSELYNRYHTIAKDVFGQIIYFFFPILDKVPYFKRPKLQSLISSYDKYVFDMVTTRREELKLNPDSKAQDLLSKLVLASEEDGKTLISDREIADNLKAFFIAGHDTTSSTLAATFYYLARYPEVQDKLRTEILEVMNNPQVLTNPTLEQLKQMDYLNMVIKESMRRMATVSIIERVSNAPFNLTNTIYLPAKTPVFILPWQTHQQSQYFSNPKEFNPERFRDPSSVESKNWQPFITGPRACIGMTLSLMEQRVSLVLLLQKFIFSIDESNPDYKQLRLTTNGIIRPRDLRLNLTQRI*
151	CYP5854B-fragment1	FLIAGHDTTSNTLTATLYYLARYPDVQNKLRTEILEVMNSPQVLTNPTFEELKQMKYLNMVIKESMRIMTTAVAVERISTNPFQLTNSIYLPSSTPVYLLMWQVHKQSKHFPDPKLFNPERFRDPSSPESKNWLPFLQGPRACNNFYNTL*

400	CYP5854C1	MPQYEEFYENSDYTNSDKFIGAESMGALNGHDWKRHRKILLPYFTKPWPIELFSNYCQLVINKWTMKGDNQEILLLDDLQRMTLDCLGKGLFDIEFNASIEPNNRLFHLWNTLNTKSNSPWYETFPILDQLPLFKRPEFDDEILEYDRLIEDFISQRMKLIEKKKADPKDNLLNAMISSYLKGLMSGLELEEIRDNMKLLILAGHDTTAYTLTSILYLLAKNPDIQSKLRSEILNTLNNPEKLTTPTSEQLPQMEYLTLVIKESMRLLTPASEAEREASQEYTLSNGITIPKGTVVSLNLWAIHHNPEFYENPGEFNPERFKLNKNGENQNWQPFLMGQRSCIGMSFSLTEIKVSLILLLQQFEFTLSENNPDFEKVRLNSNFMIFPKDLRLNVKNRLY*
488	CYP5854D1	MLTYIITGLGLATVYKVSKAIKCPKELEKVTSMPMSSFFTFVSSQESFDDKMKKDFQPVMDANGIIRVFGMTGWCLMISDYAIIKEIASKPEIFCKPDPTKQPTNLNSLKFFGKSHVAINNGQEWKRHRKIINTIFSSKWDLEIFENSTHDLIDILKLSEGEEIEVYDNIQRLTLDILGKSIFNIDFKSLKDPDSKLYSTYTYIINRLFNEPMYFIMPFLEHLPYFKRSDLSRKIDEYDQFVESIIDSRYEAIKNGEVNTENKDLITKLILSNMEEDESKRLTGDEIRDNIKIFIMAGQDTTANTITTALYYLARYHDIQTKLRSEILNVLGNPTLLTTPTLDQLKSMPYLSQVIKESMRIVTTSSAVTRIAAQDYTLSNGVTIPKGTDIYCHLWASHHNSSFPNAHEFKPERFVDGDGEWCPFFLGTRKCIGMNFSLLELKVNLVLILQQFDLSMSESNPDYEKLRLNSMNVIKPLDLKLKFKNLL*
406	CYP5854E1	MSIKPLSDVFQKVDISKNTSKLFLRFFGLSQVASANGEDWKKQRKVINPIFNQTWSTEIFGKCVQDLIDEWEKMDGKEFKIHDKIQRMALDVFGKYIFDIEFKSVKNNDSKLYNLYHSISEELFAHPIYILFPFLENIPFFRRPELSDKIEQYHEFIQELIKLKQNELNNGTLNSNGDLISAFIQSNENSEEYKLTIEQIRDNLGIFIIAGHDTTSNTLTSTLYYLARYPETQDKLRGQVLEAMDYPKNVQIPTNDQLKSVPFMDMVIKESMRIMTTASAVQRTSSTTYTLSNGVTIPKNTPVFVQLWGVHHNPSAFPNPFEFNPNRFEDISNQESKNWQPFLYGNRTCLGSTFSLMEQRVTLAMLLQKFEFSISSDNPDYHSLRINSDFIVRPYELSIRIKVRA*
490	CYP5854E2	MIYYIILGLVIYTVYKIINFVRVPPELKNIPAVPLSTFFCFNLDKRYYREKVNHYLQSYFNEFGVIRVFTHKGWSVFIADAKICKEVSILSDVFQKPEFSKNVASKHLLRFFGESQVASANGEEWKKQRKVINPIFNQTWSTELFGNCAQDLIDEWEKMEGKEFKVRDKIQRMTLDVLGKSIFDMEFKSVKNADSKLYSLYHDIAEDIFGHPIYFLFPFLEYVPFFKRPELSRKLDQYHEFIEELIKLKKEELKNVTLKSNRDLISALIQSNENSEESNLRIEEIRDNLNIFIIAGHDTTSNTMTSTIYYLARYPEIQDKLRRQILEAMDYPKNVQIPTIDQLKKMPYMDMVNKESMRIMTAATELQRVSSSSAYTLSNGITIPKNTPIFLHLWGVHHNPSNFSNPFEFNPNRFEDISNQESKNWQPFILGNRTCIGSTFSLVEQRVTIAMLLQKFEFSISSDNPDYHSLRVSSGIARPKDLSVQVKIIA*
391	CYP5854E3	MDYPKNVQIPTNDQLKKIPFMDMVNKESMRIMTTASAIQKRPSSTYTLSNGMKIPKDTPVFLHLWGVHHNPSAFPNPFEFNPNRFEDISNQESKNWQPFTLGNRTCIGGTFSLMEQRVTLAMLLQKFEFSISSDNPDYHKLRISSTKILRPKDLSIQIKKMIFYFILGLITYIGYKINKFVKVPPELKSIPAVPLLTFLHYILDKRCYRDKVNDYLQGYFNEFGVIRVLTHLGWTVFIADAKICKEVNALSDVFQKSSSSKNSSSKLLRRFIGVSQVAAVNGAEWKKQRKVINPIFNQTWSTELFGNCAQDLIDEWEKMDGKEFKIHDKIKRMTLDVFGKSIFDMEFKSVKNDDSKLYNLYHDIFEELFGHPIYILFPILENLPFFKRPQL
490	CYP5854E4	MIYYFTLGLVIYAAYKINKLLKVPPELKNIPALPLLTFFRFSRDKRFYIDKMNHYFQSYFNEFGIVRVFTHLGWTVYIADAKICKEVTTLPEIFQKQTSSKNAADNFLRRFFGASQVGSVNGEEWRKQRKIINPIFNQTWSTELFGTCAQDLIDEWEKMDGKELKVHDKIQRMTLDVFGKSIFDMEFKSVKNADSKLYNLYHDIAKEVFGHPFYILFPFLEKVPFLKRPQLANKLDQYHEFIQELIKLKQNELNNGTLKSNGDLISALVQSNENSEEGKLTMEEIRDNLNFFIMAGHDTTSNTLTGTLYYLARYPEIQDKLRGQVLDAMNYPKNVQIPTIDQLKNIPFIDMVIKESMRIMTTVAAIQRISSCTYTLRNGVTIPKNTPVFVQLWGVHHNPSTFPNPFEFNPNRFEDISSQESKNWQPFLYGSRTCIGSTFSLMEQRVTLAMLLQKFEFSISSDNPDYHKLRIGSLGIVRPKELSIRVKVRA*
474	CYP5854E5	MLQYIAVGVATFAVYKAYNIFKCPDELKQLYAAPLLSFIFFTLDMGCYRDKIDRYYQPYLNKHGIIRVLTPVGWCLYVGNARLCKEIASQADIYHRFNLLLIAPVNLRRFLGKSHVGFHNGEEWKKHRKIINPIFNQTWSTKLFGTSIQNLIDELEKMAGKDVKFYHVINSKNPNSKIYNLYDSIFKQLFGQPVYLLFPFMEYVPFFRRTKLSHQLDEYHEFIQEMIALRMDELKKGTLVENRDLVSALVKSNDNSSETKLTMEEIRENLSVFISTGHDTVTNTLTSTLYYLARYPDVQEKLRAQVLSALGDPSPSKVQIPTIEQLKNIPLLDMVIKESMRIMTTVPAIPKKSTIISTLSNGLTIPAKTAVFLHYWVVHHNPNDFPDPYEFKPERFADISNEATKNWQSFGTGPRKCIANTFALMETRVTISMLLQKFEFCISEDNPNYHKLNIASNFLLYPKDLSLEIKIRT*
466	CYP5854E6	MLYYFFFGLLAYIFYKLYIFARCPDELKHLPAAPLTAFFAFHANKKLDFAEKYKKYFAPYLNEHGVVRYLTHTGWAIYIGEAQMAKEIQMKNDIFEKPLLTASLSAIFVRFFGLSQVLAINGNEWKRHRKVINPIFNQTFSTELFGNCASDLIDEWVKMEGEEAVKNAKVKLYTLYAYIAKEIFGKQLYIMFSILEYLPFTRRPKLAQNLKEYHGYIEDLIKSKTQELREGKLSNKGDLISALIESNEKSQEYKLTMEEIRDNLNIFIIAGHDTTSNTLTSTLYYLARYPELQDKLRAQIINATGDKTQVKIPTIDQLRKIPLLDKVNKESMRIMVTVPVVQRIANSIYTLSNGLVVPKGTYIYLHLWGIHHNPSTFPCPDEFNPNRFDDISNKQSKLFQAFTLGNRTCLGSAFSLMEQRVTISMLLQKFEFSISNDNPDYYRLRITDSVIIHPKEVRLVIKSRF*
490	CYP5854E7	MLQYLATVLIAYASYKIYNWTRCPDEIKHLPALPMMAFFHFAFSKDSLPEKMKKAFQPMFDEYGVVRAFTHFGWSVFIADPKLCKEACAKPEIFIKTDFTKIPVSEHLMKFFGGSQVLSNNGSEWKRHRKVINPIFNQTWNTELFGECAKDVIAEWEKQAGGEVKVHDTIQRMTLDVFGRAIFDINFNAVKDKASRLYHLYNDITQQCFGQALYLIAPFMEHVPYFRRPNLGKQLDEYHGFVEEMMAQKKKQLHEGSAKSKDLITAFIESNEKEDEFKLTNDEIRDNITIFILAGHDTTSNTLASTLYYLARYPEIQDKLRKEVLEALDHPTELVTPTVDQLKHVPYMDLVTKESMRIMTTAVNLQRDTAQTYTLSNGLTIPKGTAVFFHLWGLHKNPTAFPNPEEFNPDRFSDLHNEESRNWQPFLTGPRSCIGMTFSLMEQRVTIAMLLQKFEFSIGKEDPNYENLNISPSGLVHPKDLALTIKLRA*
479	CYP5854E8	MFLNIILIPIAYIAYRVYQWGKCPSELKDLPSIGLIEFIKSIVSKGSYPDKFKLIQEKLNEHGIIRIFHISYGWSVMIGNPKLAKEVSSKDNIFPKHDIKKEVLSPHLKRFLGLSQVLGSVGDEWKRHRKVVNPIFNQSWDTKWKKQCNNSEVNIQDKIQRMTLDVFGKAIFDYDFKAVKEQDSELYNLYVSIFDGTFNPLYSVFPFLDNLPYFKRPEVSKKLDAYHEFIEGIIKLKEKEIVEGKENNSKNLVSSLIQSNIKLGDDKLSNDEIRDDLSIFIIAGHDTTSNTLISTLYYLARYPQVQDKLRTQILGVLENPSSVVIPTTDQLKNMPYLDLVNKESMRIMSTAAQIQRICTKEHTLSNGLTIPKNTNIMLHLWGIHHNPNAFENPDEFLPERFDQLTNEESRNWQPFATGARSCIGMSFSLVEQRVTIAMLLQAFEFSISPQNPSYEKIHISTSALVKPDNLKLIIKLRE*
447	CYP5854E9	MNTASSDLYPLNFLFVSCDRDTYLLKSLGWSVFIGSPSIAKEVLYKSDVFYKPKFIDSYMSINFKRFLGFSQVVQNNGDEWKRHRKVINPIFNQTWSTELFGNFAQDLIDEWGKVEGKEVKVQDKVQRLTLDVFSKAIFDIDFKSIKNEDSKLFNLYHKVSKQIIDYPVYIIMPFLEYLPFTRRPELAKKLGEYHEFIEDMIENKKIDLKKGKLSKKGDLISAFIESNENSKDQKLTMEEIRDNLNIFIFAGHDTTSNTLTSTLYYLARYPEIQDKLRAQVLAAMGSPKQVTIPTVEQLKKIPLMDMVSKESMRMMTTVNTTERVSKSHHTFSNGLSVPKDTPIFVHMCGVHYNPSAFSNPFEFNPERFSDISSEESKNWLAFGLGNRTCLGFTFSLMEQRVTLAMLLQKFEFSINKENPDYKQLRVTSSFISRPQDLAITIKARV*
380	CYP5854E10	ARCLQKIFLSKNDIFIKEELKKFLSSVSIKLGGPSQVVTNNGEEWKRHRKVINPIFNQTWNTRLFGSCAQDLIEEWAKEDGKEVKVRDLIQRMTLDVFGKAIFDINFDVDDLKLFRRTELDNKLNEYLDFVQSIINKKKEELQIDSEKSGDNLVSAFLKSNEKTDDQKLTMEEIRDNLLVFIIAGHDTTSNALTSTLYYLARYPEIQDKLRAQVLEALGNPSSVQIPNVKQLKNIPLLDMVNKESMRIMTTAAAVTRTAVQDCALINGLTIPKGTKVMVNLWGIHHNDKAFKNPEEFNPYRFESLTTEDSRNWQPFITGARTCVGNTFSLVEQRVTIAMLLQKFEFSIDSNNPDFHSLRLTQGSII

		RPIDLHLKVKVRN*
477	CYP5854E11	MIIYLILGIFIFIAYKIYNFFKVPPELKDIPSVPFWVDEYFQSHFNKFGVIRFFTVLGGWNVIIADAKICKEVSTLSDIFQKPDFTKFIPSKLFRKFFGVSQIFSANGAKWKRQRKVINPIFNQSWSTELFGNCIKDLIDEWEKMDGNEVKIIDKIQRMALDVFGKLIFDIDFKSVKNDDSKLYNLYHYISEQMFGNVIYIMFPFLEYVPFFRRPELSNKLNQYHEFIEEILKLKNEELKNGTLNSNGDLISAFLQSNENSEENKLSMEEIRDNLNVFIIAGHDTTSNTLASTIYYLARYPEIQDKLRGQVLDAMNYPKNVQIPTVDQLKNIPFMDLVNKESMRIMTTVSSVQRESSSTYTLSNGITIPKDTFVIVHLWGAHNNPSAFPNPFEFNPNRFEDISNQESKNWQPFTLGNRTCLGSTFSLMEQRVTLAMLLQRFEFSISSDNPDYHSLRIGSIGIVRPKDLSIRIKARN*
490	CYP5854E12	MLKYLGTTLIAYISYKVYNWTSCPEEIKHLPALRYWAFFHFVFSKDSFDDKCRKDFQPMFDKYGVVRAFTPFGWGVFIGDPKLCKEAAALVDVFPKQDLKNAPISGLLMKYFGRSQVISNNGDEWKRHRKVINPIFNQTWNTELFGDCAKDVISEWEKHVGDEIKVRDIIQKMTLDVFGRAIFDVNFNAVKGNESRLYVLYNSIIGQAFGQVLYLFAPFMEHVPFFRRPKLSKEIDEYYELIEEMIAEKKRQIQEGSTRSKDLITAFIESNEKEGEFKLTTDEIKHNINAFISAGHDTTSNTLTTALYYLARYPETQEKLRAEILEALDNPTELVTPTIDQLKRIPYLDMTIKESMRILTTAANVQRDTVKTHTLSNGLTIPKGTQIFFHLWGIHNNASAFPNPEKFNPERFSDIHNQESRNWQPFMTGPRSCIGMTLSLMEQRVTIAMLLQKFEFIIQKENPNYEKLFITPMGLVHPKDLTLTVKLRD*
334	CYP5854E13	EWKRHRKVINPIFNQTWNTELFGECVRDVVSEWEKQAGSEIKVHDIIQRMTLDVFGRAIFDVNFDAVKDKSSRLYNLYNDITDKIAGQALYLVAPFMEHVPYFRRHKLAQQLNEYHEFVEEMIKIKKKQYHESSEKPKDLITALIESNEKEVEFKLTNDEIRDNITIFILAGHDTTSNTLSTTLYYLARYPEIQDKLRKEVLEALGHPIDLTTPTVDQLKNIHYMDMVNKESMRIMATAGVLQRDTAQNHTLSNGLTIPKGTSLFLHLWGLHKNSSAFHKPDEFNPNRFSDFIAKKVFEFSINKINPDYEKLRITPFGIVRPLDLHLVIKLRQ*
482	CYP5854E14	MIQYLSATLIAYLSYKIYNLTRCPNEIKHLPSLPLWSFFCFSFSNESFPEKLKKEFQPMFDKYGIVRVFTHFGWSVFIADSKLCIFIKTDFTKIPVSENFMKFFGPSQVLSNNGEEWKRHRKVINPIFNQSWNTELFGECVRDVISEWEEQTDKEIKIHDNIQRMTLDVFGKAIFDVNFEAVIDKSSRIYHLYNDITDKISGQTLYLIAPFMEYIPYFRRPKLRQQINKYHEFVEEMMEMKKKQYRKGTEKPKDLITAFIESNEKEDQYKLTNDEIRDNITIFILAGHDTTSNTLSSTLYYLARYPEIQDKLRKEVLEALGHPAELTTPTIDQLKNIPYMDLVNKESMRIMATAVDVQRDAAQPYTLSNGVTIPKGTHIFLHLWGLHMNPSAFSKPEEFNPDRFSDLHSEESRNWLAFSTGPRSCIGMTFSLMEQRVTIAMLLQKFEFSITKNNPDYEKLRITPSGIVRPRDLHLVVKLRK*
412	CYP5854E15	MIQYLFSVLIAYATYKIYKYTRCPDEIKHLPSLPLWSFFTLSLSNESLPEKMKKEFQPMFDKYGVVRAFTHFGWSVFIADPKLCKEACAKNDIFIKQDFKKAPASENFLKFFGPSQVLRNNGTEWKRHRKVINPIFNQTWNTELFGECARDVINDWEKHAGGEIKNDFGCFGKAIFDINFKAVEDKSSRLYHLYNDIIEKVLGQAIYNIAPFMEYMPFFIRTKLRKQLNEYHKFIEEMMEMKKKQYHEGTEKSKDLITAFIESNEKEGELKLTNEEIRDNITIFILAGHDTTSNTLSSTLYYLARYPEIQDKLRKEVLEALGHPTELVTPTIDQLKHIPYMDLITKESMRIMTTVASLQRSTAQTHTLSNGITIPKGTAVFLHLWGLHHSSAFSKPDEFNPERFSDAHGEES
421	CYP5854E16	MILYLVAALVVYISYKIYTWTRCPDEIKHLPSLPFWSYFRLSLSDESFTEKMKKEFQPMFDEYGIVRAFTHFGWSVLISDPKLCKEVSVKHEIFVKQSFSEAQFSENLTKFFGVSQVLSNNGAEWKRHRKVINPIFNQSWNTELFGECARDVINDWEKHAGGEIKVDDNIQRMTLDVFGKAIFDINFKSVEDKSSRLYHLYNDITEKVLGQAMYIVAPFMDNMPYFSRPKLRQQLNEYHEFIEEMMELKKKQYYEGTEKSKDLITAFIESNEKEGEFKLTNDEIRDNIAMFILAGHDTTSNTLTSTLYYLARYPEIQDKLRKEVLEALGHPTELTTPTIDQLKNVPYMDLVNKESMRIMTTTASLQRYAAQTHTLSNGVTIPKNTQVLLHLWGLHHSSAFSKPDEFNPERFSDAHGEESRN
440	CYP5854E17	MQKEFQPLIDEHGVIRVFTHLGWTLFIGSPSACKEISNKPDIFTKPSFEDAKISRHLTSFFGKSQVVSNNGDEWKRHRKVINPIFTQSWSTQLFGACAHDLINGWEKQVDKDVKVHDLIQRMTLDVFGRAIFDIDFKAVKDPDSKLFHLYNDITEEMFSNPLYILIGFLDDLPYIGRTKLENQIKKYHEFVEEMLNLRSRELKEGKSNNSQNLITSFLKSNEKEDESKLTMDEIRDNITIFILAGHDTTSNTLASTLYYLARYPEIQDKLRSEVLEVLGNPSELTVPTIDQLKNMPYMDLVNKESMRIMTTASSIQRDTFQTHTLSNGLTIPKSTPIFLHLWGIHHNSKAFPNPFEFNPDRFKDMGSEESRNWQPFLTGIRSCIGTTFSLMEQRVTIAMLLQKFEFSITNSNPDYDKLRITASGIVRPRDLHLHIKSRA*
152	CYP5854E-fragment1	MFAAYRLYRLFKVPPELKDIPAAPLMTFIRYIKDKRSFGDKVEEYFQSQLNEFGAIRVLTHLGWTVFIGSPKLCKEVSTLSNIFEKIVLNKSKASFNFLRFVGDSQVASTNGQEWKKQRKIINPIFNQTWSTEMFGNSVQDLIDEWEKMEGD
100	CYP5854E-fragment2	TIEQLKNIPLLDMVIKESMRIMTAVSAFPKKSTIISTLSNGLTIPAKTALFLHYWVVHHNPNDFPDPYEFKPERFDDISNEATKNWQPFGTGPRTCKNI*
128	CYP5854E-fragment3	LSALGDPNPLKVQIPTIEQLKNILLLDLVNKELMRIMTTVSAFLKKSTTISTLIHHNPNDFPDPYKFKLERFADNSNEESRNWQPFGNGPRKCIGSTFALLEQRVTLSICYRSLNFVLARIIQITIS*
132	CYP5854E-fragment4	MTTASAVQRKSASISTLSNGLTIPAKTTIFLQLWAIHHNPKAFPDPFEFKPERFADNSNEESKNWQAFITGPRTCLGSTFSLTEQRVTLSMLLQKFEFCISEDNPNYHKLNVTSIGIVCPNDLSLEIKIRT*
293	CYP5854E-fragment5	MDRHYQPYLNKHGIIRVFTPFGWSLFVGDAKLCKEISTKSDLYLKPGADCIMSVNMRRFFRKSQVGLSNSKEWKKHRKIINPIFNQTWSTELFGNCAQDLIDEYEKMAGKDVKIHDKIQRMTLDVFGKAIFDVDFKSVKNPSSKLYNLYHKIFEQLFGQPVYLLFPFMEYMPFFRRTELSHQLDEYQEFIQEMIALRKDELKKGTLVDNRDLISALVKSNENSSEVKLTMEEIRANLNVFIVAGHDTTSNTLTSTLYYLARYPDIQEKLRSQVLSALDNPSPQKLKFQLLSN*
146	CYP5854E-fragment6	MPLMDMVNKESMRIMTTTASMQRVCSSIHTLGKGLIVPKNTPIFVHLWGIHHNPITFPDPYEFKPERFSDISNEANKNWMPFTLGARICPGSTFSFMEQRVTLAMLLQKFDFSISNDNPDYNSLRVTAAIIAKPKDLAICIKSRS*
67	CYP5854E-fragment7	MLFILGARICSGSTFTFMEQRVTLAMLLQKFDFSISNDNPDYNSLRVTAAIIAKPKDLAICIISRS*
221	CYP5854E-fragment8	MDYPKNVQIPTNDQLKKMPFMDIVNKESMRIMTTSSEIQRLSYFDHTLSNGMTIPKNTPIFLHLWGVHHNPSAFPNPFEFNPNRFEDISNQESKNWQPFISGNRACIGSTFSLMEQRVTLAMLLQKFEFSISSDNPDYHKLRIGSLGIVRPKDLSIRVEIVKVPPELKNIPAVPLFTFFRLLLDKRCFRDKIDHYLQSYFNEFGVIRVFTHLGRMDCVYR*
163	CYP5854E-fragment9	MNYPKNVQIPTIDQLKHVPFMDMVNKESMRIMTTASAVQRRASTTYTLSNGMTIPKDTPIFLHLWGVHHNPSAFPNPFEFNPNRFEDISNQESKNWQPFILGNRTCLGSTFSLMEQRVTLAMLLQKFEFSISSDNPDYHKLRISSTGIVRPKDLSIQVKVRA*
283	CYP5854E-	MLYKLYHGIFEELLGSPIYFLFSFLENIPFFRRPELSRKIDQYHEFIQELIKLKQNELKNGTLKSNGDLISALVHSNENSEEYKLTMDQIRDNLNVFTIAGHDTTSNTLISTLYYLARYPEI

	fragment10	QDKLRRQILEAMEYPKNVQIPTIDQLKKMPYMDMVNKESTRIMTTVAELQREPASAFTLSNGITIPKNTPIFLHLWGVHHNPSAFPNPFEFNPNRFEDISNQESIGSTFSLMEQRVTIAMLLQKFEFSISSDNPDYHKLRIATGFIVRPDDLSIRVKVRV*
139	CYP5854E-fragment11	MLYKLYHGIFEELLGSPLYFLFPFLENIPFFKRPELSRKIEQYQEFIQELIKLKQNELNNGALKSNGDLISALLHSNENSEEYKLTMDQIRDNLNVFIIAGHDTTSNTLVSTIYYLARYPEIQDKLRDQVLEAMDYPKN
285	CYP5854E-fragment12	MEYYYQPLLNKYGVVRVLTYEGWSLFVGDGAIAKEVFSNPDIYCKPDFKNARLSSNLKRFFGLNQALSANGLEWKRQRKIINPIFNQTWSTELFGNCVKDLIEEWDKEEGTEVKISDKIQRMTLDVFGKAIFNIEFKSVKNANSKLYTLYTDIFEQLFSNPVYLFFPFLEHTPFFKRPKLTKDLDEYHEFIEEMINVKKEELKNGTLNSSKDLISALIHSNENSQEYKLTMEEIRDNLNLFIIAGHDTTSNTLMSTLYYLARYPEIQNELRSQVLTAMGSPKHVQ
145	CYP5854E-fragment13	MVAFQPLFNEYGVVRAFTVFWGWSLIVGSPEVAKEVFVKNNIFAKQVFKQSFKSSTIEKLFGPSQVVSNNGDEWKRHRKIINPIFNQTWNTQLFGSCAQDVIDEWTKEDGKDVKVGDLIQRMTLDVFGKAIFDYNFNVIILNLK*
249	CYP5854E-fragment14	MDIINKKKEELASGSEKSNQNLISTFLMSNEKTDDQKLTMNEIRDNIIIFILAGHDTTSNTLTSTLYYLARYPEIQDKLRSQILKALENPTPVQIPTIEQLKNIPLLDMVNKESMRAMTTVVSVQRNTTEDYTLSNGLTIPKDTNLWVNLWGIHHNDKAFKNPEEFNPYRFESLSSDDSRNFLSFISGARSCVGNTFSLVEQRVTIAMLLQKFEFSINSNNPDFHKLRLTNNTIIHPVDLHLNIKVRN*
100	CYP5854E-fragment15	EIQQKLYEEVMTALENSKSLTIPTVAELKSMPYLDMINKESMRIMTTVTALQREAMEDCVLSNGLMVPKGTQVHLQLWAIHHDPKIFNNPDVFDPERFRE
230	CYP5854E-fragment16	SKDLITAFIESNEKEDEFKLTNDEIRDNISVFILAGHDTTSNTLSSTLYYLARYPEIQEKLRKEVLEALGHPVELTTPTVDQLKNLPYMDLINKESMRIMTTAANLQRHTAQTHTLSNGVTIPKNTKVFLHLWGLHHSSAFSKPEEFNPERFSDPHGEESRNWQPFLTGPRSCIGMTFSLMEQRVTIAMLLQKFEFSIAKSNPDYDKLRISPSGNVRPLDLSLSIKLRS*
243	CYP5854E-fragment17	MFDEYGLVRAFTHFGWSVFIADPKLCKEASAKNDIFAKPDINNQPASKYLKKFFGPSQVISNNGDEWKRHRKVINPIFNQTWNTELFGECVRDVINDWEKHAGGEIKIHDNIQRMTLDVFGKAIFDINFNAVKGKSSRLYHLYNDIVEKISGQIIYLIAPFIENIPYFSRTKLRQQINEYHGLIEEIMEVKKKQYHESSEKSKDLITAFIESNEKEDEFKLTTDEIRVIDVTLSDKYILISN*
418	CYP5855A1	MHKENGKVFLFVPNAVLVSDINAFKLLNSYQLKKSLLYKAFDINNPTIFSTREKEYHSKRKRLLSPAFSAKQVGLMESLILEMGTLNLVEHLDSQLNGEIINIRVNIYELFYRSTLDVISHLVFGESLHCIENPEEFKHYMKIISNIQKMFSLYPLFPILKRLERPTKPFERIVEENMRKRRENKSSHSDILQSMMDTQDEDSGIKLRDDEIIDEAMTLLVAGFDTTSNSLIFTIYEVLKNPTIYNKLVAEILKEFPNPDAKITVEDCRKRLPLLEATILESFRFRPVAFGPITRIVPAGGVTIDGHFIPEGTLIALNSYAINYSEEHFPHASEFNIDKWLSPERETYKSKLYTFSSGPRSCIGRELAWMEMFLVLSHLLHRFELELVPDAKLTPVSRFLLSPKENSLYVNLKKRVF*
386	CYP5855A2	KIHETYGKVVCTAKNVALVSDIESYKFLNGYKYKKGKLYSAFDLKNPNIFSSREKEFHSKRKRLLSPAFNAKSMQAMEPIILQTGTLNLIDYLSNEMELSREAELRVNVLEILYRSTLDVISKLVFGESLNCIDNPEDFKYYMKMIFRAQIILGTSPIIPFIKRYLPTAIFETIIVENMKIRRLEKTQHSDILQSMLDTQDENTGKKLRDDEIIDEAITLLFAGIDTTSNTLIFTIYEILKNRDLYNRITGQILKEFPDPNAKITVEDCRKRLSFLEATLFESLRYYPVAFGPIPRIVPSGGVTIDGRFIPEDTVIAYNTFAVNRNEEYFPTPNEFNIDKWLSPEKEVYKSRLYAFSTGPRGCIGKELAWTEMLLILSHLLHRFNI
367	CYP5855A3	MSSLIDKVLEFWSNTSYTELILVILVYQFIKFYYKDIYKVHYSGKIAHLPQPHYPLYHHFYRYYLRIFGDIADYAKDHEKYGSVFVSASNVVLTSNLEAFKLLNSYQIKKGRVYSAFDLSNPTIFSSRERQYHAKRKRLISPAFNVKSMLSMEPKILKTGTLNLLEFLNSQFNAETKQIKVNIINVFYQSTLDVISELVFGQSLNTLSGDASNFKFYMREVQKIQWVLGMSPLFPFLKKYYSAGEIFKKMIVENMESRRLEKSKNTDILQSLMDTQDGEVDGGGSGLRDDEIIDEAITLMFAGIDTTSNTLIFTIYEILKDRNLYNRITDQILKEFPDPNAKITVEECRTKLTLLEAKLFQKAGLW*
417	CYP5855A4	HKTYGKVILIDKNSVLISDIKAYKFLNGYKFKKGKIYSTFDFKNSNIFSTRNKEFHSKRKRLLSPAFNAKSMQAMESIILQTGTLNLIEYLSNEMELLNASELRVNVLELLYRSTFDVIGKLVFGGSRNFVNNPKEFRNIMKLIFRTQLVLGLIVMIPFVKKYFLPTDIVGRVIGQIINKRRLEKSQHWDILQSMIDTQKGLDLKLRDDEIIDEALVLLFAGIDTTANTLIFTIYEILKNKDLYTRIVDEILREFPDPNAIIAAEDCRKRLPLLEATLFESLRFHPVAFGPIPRIVPCEGATIDGHFIPEGTEIMFNTFAVHRSEENFPSPNEFNIDKWLGPEKEEYKSKLYAFSTGPRACIGKELAWMEMLLILSHLLHRFDIEIDPKANIIPANGFLLTPKEKCIYATFKKRIF*
255	CYP5855A-fragment1	MVSELAANIKQFWGTVSYTEIVVLALVFSFVWLYYSSIWLGFYTGPHVHLPRVKYPLYWYFYKFYLRIYGDVSAYSKIHDEHGKVILFAKNGVMISDLNAFKMLNSYQLKKSVIREREYHSKRKRLLSPAFSVRQMGLMEPLVLETGTLNLVEHLDSQFNGEIKNIRVNIYELFYRSTLDVISQLVFGESLHCIENPEEFKYYMSVISRIQLFLGLVIPFPFLRKFERPSKPFERIVEENMRKRRENKSSHSDIL
369	CYP5855B1	MLDYSSSMNLAFSLLLVLLIVYNIYRFYYWIIYPYYLGPYKNVPRVQNGLVHYMKLYYHRLLGSPNYYLQLSNDHGPVVHLVKNWVLVNDPILKKLWSNYQFPKSYSYRVFNIYGPNIFSSFDKDFHNFRKKMILPSFTKKNLKYNKDIYTIGSQNLVDMFKDKISQGQSNIFDLYHYFECSALDVITTLSIGHSLNTVRDETMSKEFFKVWTHTQYVLFLKGLIPSISTINVPTVERYKYLIEQSVKYRQSENIQFDDTLQSLMDGQDPQTGENLTLTEIIEEFFIILYAGLDTTSNTMTWTLYEILKNPKLYNLIKQEILNNFPDLTKPINLSDCEAKLVHLEAAIWESLRMHSVVETFARRVPEGG
251	CYP5855B-fragment1	MMDYNSSINVAFSLLIAVLIVYNIYNFYYWIIYPYYLGPYKNIPRVQNGLVHYMKLYYHRLLGSPNYYLQLSNDHGPVVHLVKNWVLVNDPILKKLWSSYQFPKSYSYRAFNIYGPNIFSSFDRDFHNFRKKMILPSFTKKNLKYNELVYQIGSQNLVDMFKDKISQGHSNIFDLYHYFECSALDVITTLSIGHSLNTVRDETISKEFFKFWTHTQYVLFLKGLIPSISAMSVPIVERYKYLIEQSIKRR*

497	CYP5855C1	MYPEVILIFIIGFLSYFSYKYVIYPYYLGPLSYLPRPKNAFSYIYNLYTSKLKGDCEYLLNLNLEHGPVVHLLGSIVLLSDTLMKKCITNSKFKKSSHYESFDFSGKVNLFSVRDRDQHSKI KKLVSPAFSLKTLSSIENIVYNIGSQGLVNYIESKMKSNNQEVFDFYHLFHCSTFDVITQIVFGTNYETLSNEENAQKYVNVVAETQKSMFLRSIVPFIKYFAFPIEKLFKEIIIENIKLRE NSPNPDILQSLIDSEDPETGEKLTHEEIAQECMILEDPETGEKLSHDEIARECLILLLAGMDTTAITLSWTLYMILKNPEIYKLVEDEVLEKFPNFNEPVSSEKAKNSLKYLEAALLESMRM HPVASGGIPRVVPEGGITANGHFLPHKTVIFFPIYSQHHDPTLWEDPSKFDISRWLGPNKEKNKSQLISFSVGPRSCIGRELAWNEMYLVLTNIIRNFKMELVDTDLTPTNKFLYRPLEKRM RVRMEKRY*
476	CYP5855C2	MYKEIFILTIIFYLSRLVYKYIIYPYYLGPLKNIPRPAINPFKYVFYLYKKRFQGDVQHLVDLSKEYGPVVHLVGNVVLLNDISMRKCYMTYKFKKSSYYKAFDFSGRGNLLSFREKEQHAK IKKLVLPAFSIKTLSNIEQSIYDIGSQGLVNFIQSKMKSHNQEVFDLYYLFHCSTFDVVTQIVFGTNFETTQNEENATKYVNVVAETQKAMFLRSMIPFLDYFPFPIEKLFKEHIIENIKLR ENNPNPDILQSLIDSEDPETGEKLSHDEIARECLILLFAGMDTTAITLTWTLYMLLKNPEIYKLVEDEVLEKFPNFNEPVSSEKAKNSLKYLEAALLESMRMHPVASGGIPRVVPEGGITAN GHFLPHKTVIFFPIYSQHHDPTLWEDPSKFDISRWLGPNKDKNKSQLMNFSVGPRSCIGRELAWNEIYLVLTNIIRHFKMELIDTDLTPANKFLYKPLEKRMRVRMEKR*
490	CYP5855C3	MSLDLLTNNELANIIRSHTILATFSAILLYLIYKVIVYPYYLGPLRNLPRPKNTLKHIYEVYKNKSKGNTEYFLQLSLKYGPVVHLYSNTVLLNNLSYKKYWMTNKFKKSSFYSAFDIAGLS SLFSTTDKDHHSKIKKLVLPAFSQKTLSNIEGTIYDIASQGLVNYIHAELRNGTTEVFDMFHMFHCSTFDVISELVFGSNFDTINDQQKAKYYFDIIKKTQKALFYRVIMPAYKLLPLPMEA VLGKIVQQNIDLRVNNPKSDILQSFIDSENQETGEKLTNKEIRVEGMTLLVAGMDSTANSLTWALYELLKNPEAYELVEKEMLEEFPSFNEPITVEKVKSNCKYLEAAILESMRLYPAVAGG MERVVPEGGISVDGHFLPANTIISHPIYNQHHDPKNWKNPKSYDIQRWIREGKEKNKSQLLTFGTGPRSCIARDLAWNEIYFVLANLIRHFKMELIDTKLTPAYKIFFKPEEKRMRVKITSR Y*
439	CYP5855C4	ILATLSAVLFYFLYKVIVYPYYLGPLRNLPRPKNTLKHIYEVYKNKSKGNTEYFLQLSLKYGPVVHLYSNTVLLNNLSYKKYWMTNKFKKSRFYSAFDIAGLSSLFSTTNKDHHSKIKKLVL PAFSQKTLSNIEGTIYDIASQGLVNYIHAELRNGTTEVFDMFHLFHCSTFDVISELVFGSNFDTINDQQKAKYYFDIIKKTQKALFMRVLVPIYKFLPLPMEEVLGKIVQQNIDLRVNNHKS DILQSLLDSEDPETGEKLSNEQIKTESMTLLVAGMDSTANSLTWALYELLKNLEAYELVEKEILEEFPNFNEPITVDKVKSNCKYLEAAILESMRLYPAVAGGMERVVPEDGITVDGYFLPD NTIISHPIYNQHHDPKNWKNPKSYDIQRWIGEDKEKNKAQLMTFGAGPRSCIARDLAWNEIILVFANIIRHFR
376	CYP5855C5	MISSFLISYLPSNYFQIIGIAIVGIIGYLFYSVIIYPFYLGPLKNLPRPSNPVKYIYFLYTQKLKGDCEHVLQLSLNYGPVVHYFGNVVLLNNLEYRKYWQTYKFKKSSFYTSFDVGGMPTL FSAIEKDYHSKVKKLVLPAFSVKTLANVEKTVYDIGSQGLVNYIQSTIKSGQTDIFDLFHLFHCSTFDVITQLVFGTNFNTTSNEDEADFYLNGLTGVQKAMFLRIMIPLYKLISFPMEKLF KNIVFDNIKLRENNPSSDILQSLIDSQDSETGEKLTNDQIAVECMTLLFAGMDTTANTLTWTIYELLRNPDVYELVEKEILEEFPNFNEPIPVEKAKSSLKYLEAALLESMRIYPVAPGGLP RVVPEGGKK*
380	CYP5855C6	LSLKYGPVVHLFNNVVLLNNLEYRKYWQTYKFKKSVFYTSFDVGGMPTLFSAIEKDYHSKVKKLVLPAFSVKTLANIEKAIYDIGSQGLVSHIQSTIKSGQTDEFDLYHLFHCSTFDVISEL VFGTNFNTTLDEAKAKYYVYSMGATQKAMFLRTMIPLYKLIPFPMEKLFKNIIIENIKLRENNPSPDILQSLIDSQDPETGEKLTNDQIAVECMTLLFAGMDTTANTLTWTLYEILRNPNVY ELVEKEILDEFPNFNEPISVEKAKSNLKYFEATLLESMRIYPVAPGGLPRVVPEGGVTIAGHYLPANTIILQPVYSMHNDPQNWKNPKVFDIQRWLGQDREKNKSMLMTFGAGPRSCIGREL AWNEMYLVLSNLIR
490	CYP5855C7	MSLEVTPSLSSGTYFNLQTFGALSIALILYAVYKTVIYPYYLGPLRNLPRPKNVIKYMYELYTKRLEGDAEHLLQYSLKYGPVVHYFGNVVLLNDLSFKKYWMTYKFKKSIFYTTFDMSGNP NLFSAIEKDYHSKIKRLVLPAFSVKTLDIIEKTVYDIGSEGLVSHIQSVIKSGQSDAFDLFHLFHCSTFDVITQLVFGTNFDTISDEAKADYYISALGDTQKAMFWRTMIPFYKLIPFPMEK LFKPVILENIKLRENNPHPDILQSLIDSKDPETGEKLTNEQITVECMTLLFAGMDTTANTLTWTLYELLKNPDIYELVEKEILEEFPNFNEPIPVEKAKSNLKYLEAALLESMRLYPVAPGG LPRVVPEGGVTIAGHFLPEKTIIFQPIYNLHHDPSNWKEPKVYDIQRWLGDERDNNKAKLMSFGAGPRSCIGRELAWNEMYLVLSNLIRNFKMELVDKELTPTFKFFYTPKEKRMRVKFSAR T*
378	CYP5855C8	MSLEVTPSLSSGTYFNLQTFGALSIALILYAVYKTVIYPYYLGPLRNLPRPKNIIKYMYELYTKRLGGDAEHLLQYSLKYGPVVHYFGNVVLLNDLSFKKYWMTYKFKKSIFYSTLDMSGNP NLFSAIEKDYHSKIKRLVLPAFSVKTLDIIEKTVYDIGSEGLVSHIQSVIKSGQNDVFDLFHLFHCSTFDVITQLVFGTNFDTISDKAKADYYISALGDTQKAVFWRTMIPFYKLIPFPMEK LFKPVIFENIKLRENNPHPDILQSLIDSKDPETGEKLTNEQIAAECMTLLFAGMDTTANTLTWTLYELLKNPDIYDLVEKEILEEFPNFNEPIPVEKAKSNLKYLEAVLLESMRLYPVVPGG LPRVVPEGGKR*
479	CYP5855C9	MSVVEFSNIPPTGNYYLHAFGVSLLALIIYILYFNVIYPYYLGPLRNLPRPKNVIKYLYDIITKRIEGDVEHQLLFNLKYGPIVHFHGDTVLLNGQSFRKYWMTYKYKKSEFHRAFDIGGYQ TLFSATEKDYHSKIKKLVLPAFSVKTLASVEKTVYDIGSQGLVSHIQSVIKSGQSDVFDLFHLFHCSTFDVITQLVFGTNFDTISDKDKAIYYISSLADTQKAVFWRTIFPPYKKVAFPMEK IFKPVIFENIKLRENNPNSDILQSLIDSIDPETGEKLTNEQIAVEFMTLLFGGEDTTANTLTWTLYELLKNPEIYNLVEKEILEEFPDFNESITLDRSKSKLKYLEASFLESMRMYPVAAGG LPRVVPEGGTKIILPIYSLHNDPKNWKNPKIFDIQRWLGEERENNKAQLMSFGAGPRSCIGRELAWNEMYLVLTNLIRNFRMELIDTDLTPCSTVFYKPKEMRMKVKIFIRK*
220	CYP5855C-fragment1	MEKLFKNIVFDNIKLRKNNPSPDILQSLIDSKGSETGEKLANDQIAVECMTLLFAGMDTTSNTLTWTLYELLKNPDIYDLVEKEILEEFPNFNEPIPVEKAKSNLKYLEAALLESMRLYPVV TGGLPRVVPEGGVTIAEHFLPANTVIFYPIYSQHHDPKNWKKPKTYDIQRWLGEDREKNKAQLMTFGAGPRSCIGRELAWNEMYLVLSNLIRNFRMEL
164	CYP5855C-fragment2	FIILYAGLDTASNTMTWTLFEILNNPKVYDLIKSEILSNFPDLTKPLNISDCESKLKYLEAAIWESMRKHPVVEMFARRVPSEGVTIGDHYLPEKTVITLNLYAYHNNPKIWKNPREFKINR WLGEEREKSMENFIGFGSGPRSCIGKDLAWAELFLVLANLIR
249	CYP5855C-fragment3	MEKLFENHIIRNIKLRENNPNPDILQSLIDSEDPETGEKLTDKQIAVECMTLLFAGMDTTANTLVWTLYELLKNPDIYKLVEKEILQEFPNFNEPIPVEKAKSNLKYFEAALLESMRLYPVA PGGLPRVVPEGGVTIAGYYLPEKTIIYHPIYNQHNDPRNWKNPKTYDIQRWIGEHREKNKAQLMTFGTGPRSCIGRELAWNEMYLVLTNLIRNFRMELIDKELTPTFKFLYKPEEKRMRVKI SLRN*
334	CYP5855D1	LFSSRDREFHSRRKKLISSAFSVKGLSLMEDRIASVGSESLVNYLNQNLMNEESKEFDLFKLFHYNTLDVISELIFGKNLNTTTDSQSAKFYFEGIEKTQKLLFMRLLVPFSNLIRLPMESM FKPIILDNIQKRRSTSKTHNDILQSLIDARDPDSGEGLKDLEIVDECLVLLFAGMDTTANTMTWTLYEILKHPDVYRLVRDEILEKFPNLNRPISYDLARNGLEYFDACVLESMRKNPVGAG PMPRVVPEGGLTVNGYYLPPKTVIAMDIYSQHNDPSIWPNPLKFDISRWLGPDRESNKSKLLSFGLGPTSCVGRELAWMEIYLVLVELIR
479	CYP5855D2	MMSLSTLAYLIPVALIYLCYSKIIYPYYLGPLKNIPRGKNAFKHYFSYIYDLIVGDASSHLKLAQKYGPVVHLVDETVLVNDPQIRKYYMSYKWPKAASYYKVFDFNGPNLFSALKKDFHVS

		MKKLLLPAFNNKSLAAMEDTIYKVGSESLVQYIDSFLEDNQSYEFDILDLFYSNTLDVISELVFGSSINATWDKEKGAQFMDLLSKSQFMGFIRSFIPFSYLFKLPMEPLLTPMILKNIHNR KQNNEKHHDILQTMIDAKDSETGAGLTDLEIVDECMALLFAAEDTTAITLSWTLYELLRHPEFYKLVADEIIEKFPNFNEPINSQDAKKELKYLEAAILESTRKHPAGADILPREVPEGGLT INGYHLPPKTVFTLDIYLEHNDPSFWENPRDYDINRWFGEDREAKKAKLVGFGLGPRSCIGRDLAWNEIFLVLANLIRHFSFELVDKELIPVNYFILKPKGNSFKVKISRRS*
489	CYP5855D3	MSSRLVSFIELKNFGTIGYVVSALFAYFAYSSIIYPYYLGPLRNIPRGKNAFWHYIAYMYDYITGDPAKHLKLIQKYGPVVHVRNNLVVINDASVRQYYMSYKFPKSKLYSLFNINGPNIFS ALNKDFHVKMKKLISPAFNNKALAAMESSIYKAGSESLVKYLDSYLDNEPNREFDFYHLFNCNTFDVITKMVFGTEFSTTWDEKKGYYYRDLLQKTVKGHFYRIFIPFYDRLELPIETVFKP IILENIHKRRESNGIHNDILQIMIDSEDPDTGAKLTDLEIVNECYILLLAGMDTTANTLTWTLYELLKHPEILELVTTEILNNFPNLSEPITINNAKKNLKYLEAALLETMRMHPGAGGSIP RVVPEGGVTICGHYIPPKSWIAVDIYTENNDPSFWENPREFNLDRWMGENREANKAKSIPFGLGNRSCIGRDLAWYELYMLLADLIRHFDFELVDEVLTPDYKLVYRPKEQTFRIKVSRKSH *
490	CYP5855D4	MLEDLINKAGEVIGSIGTLGYFLSALAAYFYYSRILYPYYLSPLRNLPRPKSGLWHYYSYLRQVFQGNASVNMDLSLKYGPIVHVKDKIVLVNDPIIRKSFTTYKFAKAESYSLFSINGPNL FSTTDKDFHQRIKKLILPAFNNKTLDAMEPTIYRVGSESLVQYLDSFMDQEPSKEFDLFHLFHTNTLDVISELVFGETLNTTWDKKKGIYYIDELAKTQYMAFLRTIVPFYNHIKYPMEKLF MPVIMENINKRRNSNEIHNDILQSMIDSKDPETGEKLSDQQIVDECMVLLFAGMDTTANTLTWTLYEIIKHPGIYELVSNEIIEKFPNLNEPISLNVAKNELKYLSAAILEAMRMHPVASGA LPREVPEGGITINGHYLPPKTVIAIDIYTQNNDPNFWENPRKFDLSRWLGPNAEINKNRLFTFGIGTRSCIGRDLAKNEIYLVLSNLIRHFSFELVDKELTPNNKFLYKPKEKRFKVKLSRR V*
312	CYP5855D5	MRRTFAGDAAKNMDLCLEYGPVVHLRDKLVVINDDSIKKNYMTYKFKKLITTNIKKLILPAFNNKTLAAMEPTVYRVGSESLVQYLDSFLDNEPSKEFDLFHLFHTNTLDVISELVFGETLN TTWDEKKGLYYIDELAKTQYMMFLRAVVPFYNHIKYPMEKLFMPVIMDNINKRRNSNEVHNDILQSMIDAKDPETGKGLTDLEIVDECMVLLFAGMDTTANTLTWTLYEMIKHPELYELIAN EVIEKFPNLNEPISLDVAKSELKYLSAAIQEAMRMHPVAAGQLPREVPEGGLTIQGHYLPQGVSNML*
482	CYP5855D6	MLEYLTGIGIFGFIICGLLAYFYYAAILYPYYLGPLRNIPRPKNGFWHFINHIRNELTGNPNTYIELSLKYGPIVHLRDKLVLINNSDIRKCYINYKFPKAKVYELLSYNGPNLFSTTSREY HASRKKLILPAFNNKALVTMEPTIYRVGSESLVQYLNSCLDSETSKEFDFYNLFHCNTLDVISELVFGETLNTTWDEEKRIFYIEELSKTIYATLLRALVPFYTYFTHPMEKLFKPMIMENI GKRRKLTEVNDDILQCMIDAEDPETGVKLTDSEIVDECLVLLFAGMDTTANALTWTIYELLRNPEVYELVAKEVLEKFPNLNEPISVDIAKNELKYLDSAITGAMRMHPPAAGILPREVPEG GLTIAGHYLPPKTEIAIDIYTQHNDPAFWENPRKFDIDRWLGPNAEFNKEKLFNWSTGPRSCIGRDLAKAEIYLVLTNLIRNFNFELIDKELTPMNISIYKPVERRFRVNVSRRS*
427	CYP5855D7	MMLGKMDVHLDLVQRYGSIVHVKDNMVLVQNPNLKKHYMTYKFAKHPIYKLLDISGPNLFSSTDKDFHASRKKLIAPAFSIKSLSKMESTIYRAGSDSLIKYLHSQLDKNESKEIDFYPLFN SNTMDIITELIFGESLNTTWDKSKSQYYSQLTTTSGYNIFLKAVLPFYKPLNSPIETFKPRILENIKIRRESTNVHYDILQSLIDAEDPETGGKLSDLEIFSECIVLLFAGIETTSTTLTWT LYEILKHPEVYKLVADEVLEKFSDLNNPVSISEAKTELKYLEAAILESMRINPVVTGALLRVVPEGGITVEGCYLPENTTMSMDSFAQHMDPALWKSPETYDISRWLGSDREKNKSLLFTWG FGPTSCVGRELAWAEIFLVLVNLLRNFNFELVDKTLTHYLTFMNKPKEKRFRVRISRRVR*
350	CYP5855D8	MVLISDPEIRKYYMSYKCPKASYYEVFDFNGPNLFSALKKDFHVNMKKLLLPAFNNKSLAAMEDTIYKVGSESLVQYIDSHLDEHSSYEFDILDLCYRNTLDVISELVFGTSINATWDKKKG KEFIDALANTQFMLFIRYFVPFSYLIKLPMEPILTPMILNNIHERKQKNEEHHDILQSMIDAKDPETGVGLTDLEIVDECMTLLFAAEDTTANTISWTLYELLKHPEVYKLVADEILEKFPN FNEPINSQDAKKELKYLDAAILESTRKNPAGADIIPRDVPEGGLTINGHYLPPKTVFTLDIYCEHNNPNFWENPREYDINRWFGEDREAKKARLAGFGLGPRSCIG
150	CYP5855D-fragment1	KAKNELKYLSAAILEGFRMHPVAAGQMPREVPEGGMTIQGHYLPQGTEITLDIYTQHNDPNFWKNPREFNLDRWLGPDTEANKNRLVTFGLGPRSCIGRDLARSEIYLVLANLLRNFNFELV DKELIPTNKFLYKPEGKRFIVKVARRV*
67	CYP5855D-fragment2	LAGFGLGTRSCVGRDLSWNELYLVLANLIRHFNFELVNKEVVSIYKFIHRPIDKKLGVKLTRRSKN*
120	CYP5855D-fragment3	RDLKYLEAAILESMRMHPAVAGSLPRQVPEDGLTVNSHYIPPKTSITIDIYTEHNDPSFWKNPREFNLHRWLGEDRETNRAKLAGFGLGTRACVGRDLAWSELFLVIANLIRNFNFELVD
153	CYP5855D-fragment4	YELLKHPEFYKLVADEIIEKFPNVNEPINSQDAKKELKYLEAAVLESTRMHPAGADILPREVPEEGLTINEYYLPPKTVFALDIYCEHNDPKFWENPREYDINRWLEDREAKKAKLVGFGLG PRSCIGRDLAWNEILLVLANLIRHFNFELVD
200	CYP5855D-fragment5	DILQSLIDSKDPENGEGLKDLEIVDECLVLLFGGMDTTANTMTWTLYEILKHPEVYKQVTSEILTHYPNLKEPITVEKTKSELKYLEACILESMRKNPVASGAIVRVVPESGITISGHYLPP GTSVGLDIYAQQNDPSIWKSPEIFDISRWLGEERELNKSKMFNFGIGPTSCIGRELAWSEIFLVLANLLRSFELEMID
67	CYP5855D-fragment6	HNDPNFWENPREFNLDRWLGPKADTYKNKLVTFGLGPRSCIGRDLARNEIYLVLANLIRHFNFEIVD
218	CYP5855D-fragment7	MSYSILKIIVTIIIVLLTYFAYAKIIYPYYLSPLRNLPRNPNAIKHWFKNLHHTITGRLDINLQLIQAYGPVVHIEDNYVLIQDNDFRKIYLTYKFKKDPIYSLLDVNGPNLFSSMDKGFHS SRRRLISPAFSLKNLQKMEPTIYRVGSDSLIKYIESKLINNKAEEIDIFQLFHSNTMDVITELIFGSSLNTTWDRKKAEYYNKIIEKTQKTIFLD*
405	CYP5855E1	MVLVNDLNARKWLLTYKFNKDDSYHIFNFRGPTIFSTADKEFHQIRKRLVAPAFSNKNLAKMEPTIYKVGSDHLAQFISSSMGDEKEVVLDMYNLFHRSTLDVIGELVFGQNLNCILDSKKA DHYMNELKKTQLLIVLRTLCPSIFTLFTYPILTLFEPTILKNINARRQDPSLRNEDILQSLIDAKDPETGEQLTDVQIVDECVTLLFAGMDTTANTLTWVLYEILRHPEVYALIEKEIFTAF PNAEDVATIERCKSELPALEATILESMRLHPVAAGPMFKKVPQGGVTLNGQFIPENTSVIFHVITYHLDPNYFENPKQFSIDRWLGPNREENKGKLLTFSMGPRGCVGRDLAWSEIYLVIVK LIRMFRMELVEKEPMKPELYFLYTPKDGKFDVKLTKRV*
98	CYP5855-fragment1	MKKFSDLEIVEECIVLLFDGVYTTSNTLIWTMYETLKHPEIYKSKNRVKILRGAILVSMRMNPIASGALLRVMPKGGIAIRRYCLTKNILKYVLSIA*
493	CYP5856A1	MLYAILFIVILASIYFSVNKVPTEFKGIPKVSLWSFIRSIQKEDTLQEHYHGIIEPAIKERGYALVFLKSSWSLAVTRSDIIKQVFRNSQQFTKDVIRHHKSLEYKFTGKQNIVRSNGDDWR RFRRPANPIFNQTFQSDIFAESVKEVLNALTDRVNEHGSNPFEVSDLMELMTLDILGKGIFDFDFEAVRLKGTSKYHHIYNSAFSGVFDPLYLLLPFIEKLKLKKRMKKHHDAIEFKKFILG IVEERRSDIKAGKANGKDLLATMLAADPESPFEPLTDEELVENLIVFFIAGHDTTANTLSYAMYYLARNPEIQEKLRNEIYTTLKIPKDHEKLVVASTEQFKEMEYLNCVIKEVMRISPAVV

		QMFRIVEEDFNIQNENIVVPKGTLITLSVYGAHHDPKTYPNPHKFEPERFLNGKYDTDIYLPFGGGSRMCVGMGFSLMEQRVYLTLLLQKFTIEIKEDNHDFNSLRLSGLVLTKAKDLKLNFHPRF*
458	CYP5856A2	MLQSIRKGETLQEHYHSIIEPVIKERGYALVFNRGHWSFTTTRPEVIKQIFRKTKQFTKYAIRHPESLENKFTGQQNIVRSNGDDWRRFRRPANPIFHQTFQPEMFAECVNDVLNVVTDRINEHGSKPFEVSDLMEFMTLDILGKGIFDFDFEAVKLQGTSRYNHIYNSVFSGIADPIYLTFPILEKLKLKNRVQKYEDANEFKNFILDIVNTRRKEIKSGKNDGKDLLSTMLTEDPDSPFEPLSDVELVENLIVFFVAGHDTTANTLSYAMYHLARNPEIQEKLRKEIYTTLNIPKDHDKLVAPSTEQLKDMEYLNCVIKEVMRISPAVVQIPRIVEEEFTIPNDNVVVPKGSMISMSIYGAHHDPKTYPNPQKFEPERFLNGKYDTDIYLPFGGGSRMCIGMGFSLMEQRVYLAMLLQKFTLEIKQDNPDYEQLRMVGLRLTKAQDLRLNFTPRF*
386	CYP5856A3	LEFKFTGEKNIVRSNGDDWRRFRRPANPIFHQTFQPDIFGECVNEVLNALTDRINENGSKPFEVSDLMEFMTLDILGKGIFDFDFEAVKLKGTSQYNHIYDSAFSGIFDPLYLLFPFLEKLKLQKRMQKHIDAIKFKKFILEIVEKRRAEMKAGKVGGKDLLATMLTVDPDSPFAPLTDQELVENLIVFFIAGHDTTANTLSYAMYHLARNPDIQEKLRSEIYNTLNIPKDHTEVVVPSAEQFKEMEYLNCVIKEVMRISPAVVQIPRMVEEDFTVPNDNVVIPKGSMIMMSVYGSHYDPKTYPNPKKFEPERFLNGKHETDIYLPFGGGSRMCIGMGFSLMEQRVYLAMLLQKFNIEIKEDNPDYDCLRLNGIALTKAKDLKLNF
493	CYP5856A4	MLYLLICCFIVALGVLYLIKVPENFKNVPRVSILSTLHSIITRKTVQEHHSKILAPKLSKSGFAIVWVRGGWSLHISKPSAIKQILKNTRVFTKFRIHPASLNRRFTGKRNIVRSDGEEWRRYRKPVNSIFIQSFCTKMFADCVKEALSVLEYRVNEFSGKPVVVSDLMEYMTLDILGKGVFGYDFKAIKNMGASHYHHLYKTVFNGVQSALFLYFPILEYFPIFGRSKLHKNCDEFNKFIGEMVSKRRKEIEKYKNSDYKDVLSLMLENDPDSPYEPFSDEEIIGNLNIFIIAGHDSTAHTLSYTMYYLAKDQNIQQKLRNEVYKTIEIPANQAEIVAPNTESLKDMVYMDCVLKEAMRMSPAVVNLHRHVAEEFQFPGEDIVVPKDNLVSIAIYGVQNDPKIHQNPKIFSPERFINTRYDTDTYLPFGGGSRMCLGMNFSLTEQKVYLSMLLQKFTIQIHKDNPDFERLRMNGLIFSKAKDLKLNFVQRF*
183	CYP5856A-fragment1	LTDKELINNLNIFFIAGHDTTANTLSYAMYHLAKNQHVQDKLRAEIYSTLQVDPNTKDLVIPTSEQLKSMNYLACFIKEVMRITPAVLQVNRELQSDYTIPTDKVVLPKGTQVMLSIYSIQHDPKIYPKPEEFDPERFLVGKYDTDIYMPFGGGTRMCVGMNFSLMEQKVYLSLLLQKFTLGI
458	CYP5856B1	MKAALTGKTIHYRFKEVVWPKLREKGYAIILGPFGWNVHIAKPAVAKNLFRNSNQFLKPVISNPGSLTELFLGEVNIVGSNGEQWKRLRRPANPIFGQSFQPEAFSPCVLETLDALDERVNRLQGQPIEVKDLMELMTLDVLGKGIFSHDFEAVKSLGNSNYHKLYKGIMSFLFNPFFVIVRPLAKLSFGPPERARKNINEFNSFILGLINQRRKDLKNGKFLDSKDLLSTMLRDDPDSPYEPLTDEELVHNFNIFFLAGHDTTANTLSYALYHLARNRDVQDKLRNEIYQKMNLDDNQEKLVVPTSEQLKNMEYLNLVIKETMRVSPAVQQIGRKLAEDYTIPEDNIVLPKGTSVTLSIFSILNDPKIYPNPEKFDPERFLNTKYDIDTYMPFGGGSRMCVGMNFSLMEQKIFLSLLLQKFDLRITKDNPDYEQLRVSGLGLTRPMDLKLNFEARL*
485	CYP5856B2	MIQLLIILGCITAFLAYLFKVPKHLQSFPRVSFFGLVNTIFTGRSLDEHYKKVLDPALKEKGYAAQFWLGKWNVVVTQPQIAKQFFRNTTTFHKELPNIHGLFGKFLGKSNIFFSNGEDWRRHRKPANPIFHQTFHPEQFASSIEDTFEELMERINQSPDEALPVTDLMGLMTLDVLGKGIFSVDFEAVKSKGSSRYHHLYTSIINQIPNPVYFFFQFLHRYPMGKMAEAHKNIGEFKEFTMKMIQQRRKDLKDHKFEDSKDLLTTLLKETELNQEDPLTDEELVHNLNVFFIAGHDTTASSLSYGMYHLAKNQEVQKKLRKEIYEVLEISPETKKLKSMEYLGLVIKETMRISPPVGLLNRALADDYTIPGDSVVIPKGTVVGISIYAVHNDPKNFKNPEKFEPERFLNAKYDTNVYMPFGGGSRMCIGTNFSLIEQKVYLSMLVQKFILNIKKTNPDYENLRIRGLGLNRPQDLKLNFVPRF*
458	CYP5856B3	MVKTLFTQKSADEYYIKVLAPALKHRGYASMLWRSNWIVVVSKPEIVSQLFRNTDRFPKSMSNFPGILGKYIGPSNILISNGDEWKRHRKPANPIFHQKFQPELFSPCIEEVLNSIMDKVSRNPEESIQVCDLMSLLTLDVLGKGIFSIDFEAIKSEGSSKYHQLYYSIMKQVGSPLYSLIPFLNHYPVGDMAKAYRSLDEFKEVIQALISQRRKDLKDKKFEDSKDLLSTLLRETEKNHEDPLTDEELVYNMNVFFAAGHDTTSSALACGMYYLAKNPIFQEKLRNEVLQALGTSSASDKITVPEESQIKEMNYLGLFIKEVMRLNPPISQVFRGLTEDYSIPDDDVILPKGTIINLPIYAIHHDPKIYPNPEEFNPERFLNGKYDTDVYMPFGGGSRMCVGFNFSLMEQKVFFIMLLQKFIIEIMPDNPDFEKLRIMELGQIRPLELKLNFKPVL*
204	CYP5856B-fragment1	MSLISISIILLLILCIYLHRKITTPPKFAKNLPVIPFLHTIYAAFTNKTVDYRFQNIEWPKLKEKGFAVMYGPIGWCIYVSKPDFAKKMFKDSNKFIKPIMKLKGSLGQLFAGDINIAASNGEEWKRLRRPANPIFSQTFQPEVYSPCVLETLDVLDERVNRLQGKPVEVKDLMELMTLDVLGRGIFSHDFEAIAVWTTNENS*
266	CYP5856B-fragment2	MRTRKKIIEFNNFILSLISQRRKDLESGKFLNSKDLLSTMLRSDPDSPYEPLTDEELVHNLNIFFLAGHDTTANTLSYALYHLARNRDVQDKLRNEIYQKMNLDDNQEKLVVPTSEQLKNMEYLNLVIKETMRISPAVLQIGRQLAEDYSIAEDNIVLPKGTNVGLSIYSILNDPAVYPNPEKFDPERFLNNKYEADTYIPFGGGSRICVGMNFSLMEQKIFLALLLQKFDLSVTKDNPDYEKLRISGLGLTRPMDLKLNFEARF*
203	CYP5856B-fragment3	DLLSTLLRETANNHEDPLTDEELVHNLNVFFVAGHDTTANTLACGMYQLAKNKQVQDKLRKEIYDTLQIQPGTNKIVVPTFDQIKNMEYLGLFIKEVMRINPSVAQIVRKLTEDYHIPSDDVVIPKGTTVNVSIYGIHHDPKIYPNPEKFDPERFLVGKHESDVYMPFGGGSRMCIGSNFSLMEQKVYLTMLLQKFTIDIKTT
267	CYP5856B-fragment4	MAKAYENLEEFNGFIQSLIDQRRKDMKDHKFEDSKDLLSTLLRETENNHDDPLTDEELVHNLNVFFVAGHDTTANTLSFGMYYLAKHRHVQDKLRKEIYDTLQINSGANKVVIPTAEQIKSMDYLGLFIKEVMRLNPPISQIFRTISEDYPLPDDNVILPKGTTVNLSIYSVHHDPKIYPNPEEFDPERFFNGKYDTDVYMPFGGGSRMCIGFNFSLMEQKIYLIMLLQRFDIHIGSNNPDFDKLRIKGMGMIKPTDLKLNFEPRF*
191	CYP5856B-fragment5	LTNEELVANLNVFIMAGHDTTANTLTYAMYYLAKYQDVQEKLRNEIYATLEVAPNNFDLAIPTAEHLKSMKYLSCFIKEVMRITPAVLQIDRQLSSDYTIPGENVVLPKGTHLNLSVYSIQKDPRIYPNPDEFNPERFLNSKYDTDVYMPFGGGTRMCVGMNFSLMEQKVFLIMLLQKFHLNIDKNNPDFK
108	CYP5856B-fragment6	MRISLTVPQMGRQLAEDYLIEEDNIVLPKGTNVGLSIYNILNDPAVYPNPERFLNIKYDMDTYMSFSRSSGVCARMNFGLIEQKIFLSLVLKNLELIIAKLTQIKNN*
226	CYP5856B-fragment7	MRKSDSNDQDRLTDEEIVHNLNSFFVAGHDTTANSLACEMYHLAKNPNIQEKLRKEVFTTLKIESNASKLAIPTIDQIKEMEYLELFIKEAMRINPPVAEISRVLNDNYIIPGDHVVLPKGLRVSVSIYGIHHDPKIYKNPEEFNPERFLNGKYDTDVYMPFGGGSRMCIGSNFSLIEQKVFLIMLLQKFKVDIKPENADFEKLRIRGTVHTKPEDLNLNFVPVF*
227	CYP5856B-fragment8	MLRKDPESPYEPLTDEELVQNINLFFFAGHDTTASTLSYALYSIARNRDIQDKLRNDIYQKMGLSDNHKKLVVPTSEQVKNMEYLHLVIKETMRMFPPILHLGRTLGEDYLIPEDNIVIPKGTNIALSIYSVLHDPKIYPNPEKFDPERFLNAKYDVDTYMPFGGGSRMCVGINFSLVEQKVFFALLLQKFDLKIAKDNPDHEQLRISGFGLTRPTDLKINFEARF*
191	CYP5856B-fragment9	SPYEPLTDEELVQNINLLFFAGHDTTASTLSYALYSLARNRGIQDKLRDDIFQIMGLNNNHKKLVIPTSEQVKNMEYLHLVIKETMRMFPPILHLGRTLGEDYPIPEDNIVIPKGTNIALSIYSILNDTKIYPNPEKFNPERFLNAKYDIDTYMPFGGGSRMCVGISFSLMQQKVFFALLLQKFELGIAKD

227	CYP5856B-fragment10	MLRKDPESPYEPLTDEELVQNINLIFLAGHDTTASTLCYALYNLARNRDVQDKLRNDIYQKMGLNGNHKKLVIPTSEQVKSMEYLHLVIKETMRMFPAIIHIGRQLAEDYPIPEDNIVIPKG<br>TSIALSIYSILNDPKIYPNPEKFNPERFLNAKYDIDTYMPFGGGSRMCVGISFSLMQQKVFLALLLQKFDLRIANDNPDYEQLRVSGFGLTRPTDLKISFEARF*
227	CYP5856B-fragment11	MLRKDPESPYEPLTDEELVHNINLIFLAGHDTTASTLSCALYNLARNRDIQDKLRNDIYQKMGLSDSHKKLVVPTSEQVKNMEYLHLVIKETMRMFPPILHLGRQLSEDYPIPEDNIVIPKG<br>TTIALSIYSILHDPKIYPNPEKFDPERFLNAKYDVDTYMPFGGGSRMCVGIKFTLMEQKVFFALLLQKFDLRIAKDNPDYEQLRVSGFGLSRPTDLKISFEARF*
191	CYP5856B-fragment12	SPYEPLTDEELVHNINLIFLAGHDTTASTLSCALYNLARNRDIQDKLRNDIYQKMGLNDNHKKLVVPTSEQVKNMEYLHLVIKETMRMFPPILHLGRQLSADYPIPEDNVVIPKGTTIALSI<br>YSILHDPKVYPNPEKFDPERFLNAKYDVDTYMPFGGGSRMCVGIKFTLMEQKVFFALLLQKFDLRIAKD
485	CYP5857A1	MSSYSIEDLSLLHVGVTVLLGYLGYVYYKNFHYPRHVGPLRSIPGPSNELLLSIRFMYARLTGNPAKFYKDLHAKYGPIVHSGLGVVAVNDPEATKTIYGSYKFIKSYRFEFFIENVQNIFS<br>TSDRKYHSIRKRMMSPAFNWKSVMDLEPQVTVHVVDNTLTAINEILSNGNTQIDVYELFHKSIGDAISDTVLGKCFNSLKIPDFPSYQLCSHIADTWGLKFAVPALGFLKSRHHPIINQNIV<br>EELKERRAGKYRKDILQTLVDAKDTETNSTFTDEEIIEEASILIFAGMETTAVALIWTFYLLGKNPQVYEKLVDELVTAFPDKNTKISYDMCKDLPYLTAVIHESLRVKSPAGVVLPRVVPE<br>GGATICGHFLPEGTVVGSSGVGIHLSESNVQDPESFTPERWLGPEAEKLKDMFFAFGLGPRGCVGKNLAWLEMYISLANVIRQFDFSMPKGTQLTGFDLFVLKGKEEKLLLNTSPRSC*
485	CYP5857A2	MLNYSTGDITLSHIALAAFIGYLGYSYYINFYYSRHIGPLKSIPGPSNELWQSIRFMYYRLSGKPREVYKELHAQYGPICHSGMSVISISDPEATKLIYSSHKFKKEPRYDFFIQNVSNIFS<br>TRDKKFHSIRKRIISPAFNWKTIMKLEPMISLHCVENLVDTINKTLDNGSTQIDIYELIHKSIGDAISDIVLGRCFDSLKKPDFPAYKLSIHISNTWGLKFTVPFLNFLKASHHPIVNENII<br>EELKLRRNGKKREDILQVLVDSKDYETNSTFSDAEIIEEACILIFAGMETTAISLIWTFYLLLSNPKTYENLVEELINAFPDKNTQISYDMCKDLPYLTAVIHESLRLKNPSGVALTRVVPE<br>GGATILGHFIPEGTIIGTGGYGIHQCEKNWPNADSYIPERWLGSDADKLKDMLFAFSQGPRNCVGKNLAWLEIYITIANLIRQYDFSMPTGTTLTEFDLFVLKGKEEKLILNTTPRSN*
469	CYP5857A3	MLGFLIEGVTLPHILLAIIIGYLGYFYYKNFHYPRHVGPLRSIPGPSNELLLSIRFMYARLTGNPAKFYKDLHAKYGPICHSGLGLVAISDPEATKLIYSSYKFEKEYRFEFCIENKRMISP<br>AFNWKSVMQLESQVALHCVDNTIFAINEVLDNGNTQVDVYDLFHKSIGDASSDLVIGRCFNSLKKHDFPATKLSGHIANSWGLKFALPFLGFLKSRHHPIINQSIAEELKERRAGKYRKDIL<br>QSLIDAKDTETNSTFTNEEIIEEASILIFAGMETTAISIIWAFYNLLKNPETYMKLSRELISTFPDINIYINYDMCKDLPYLTSVIHESLRLKTPAGCPLPRVVPEGGVNILGHFLPAGTIV<br>GASGIGIHLSEKNVQDPESFNPERWLGPEAEKLKDMFFAFGLGPRGCIGKNLAMLEIYITLANLIRKFNFSMPKGTTMTDFDLFVLKAKEEKLILKTVRRSN*
317	CYP5857A4	MISPAFNWKSVLQLEPQISLHVVDNTIAAINEYLAIGSTQVDVYELFHKSIADAISDLGLKLTIPGINFLKSRHRPIINQSIVEELKECKAGKYRKDILQTLVDAKDTETNSTFTDQEIIEE<br>ASILIFAGMETTAVALIWTLYLLEELINTFPDKSAKISYDMSKNLPYSTAVIHGSLRIKSPIGLLLPRAVPEEGITLCGHYLPEGVSLFNYYSSSASGIHLSERNFQDPESFTPERWSCSDA<br>DELKDMLFAFSLGTRGCVGMNLAWLEMYISLANVTRQFDFTIPEGTELTGFGLLVLKGKEEKPILNIIPRSN*
336	CYP5857A5	PQISLHVVDNTIAAINEYLNIDRTQVDVYELFHKSIGDAISDLVIGRCFNSLKKPNFPAYKISSYIADSWGFKFTFPFLRFLKSKYHPIINQYILEELKERRAGKYRKDILQTLVDAKDTET<br>NSTFTDQEIIEEASILIFAGMETTAVALIWTFYLLGKNPQAYEKLVDELVTAFPDKNTKISYDVCKDLPYLTAVIHESLRVKSPAGVVLPRVVPEGGATICGHFLPEGTVLGSSCVGIHFSE<br>INCHKAESFIPERWLSPEAEKLKDMFFTFGLGPRGCVGKNLAWLEMYISLANVIRQFDFSMPEGAELTEHDLFALKGKEQKLLLNATLRAN*
335	CYP5857A6	PQISLHVVDNTLIAINECLDNGSSQIDVYELFHKSIADAISDLVIGRCFNSLKKPNFPAYKLSSHIADSWGLKFSFPFLRFLKSKHHPIIIQNIVEELKERRDGKYRQDILQTLIDANDAET<br>NSAFTDEEIIEESSGLIFAGMETTAMGLIWTFYLLGKNPQAYEKLVDELVTAFPDKNTKISYDMCKDLPYLTAVIYESLRAKSPSSSLLPRVVPEGGATICAHFLPEGTIVGASSSGIHLSE<br>QNFKNADSFIPERWFGHEAQKLKSMLFSFGFGPRDCSGKSLAWLEMYISLANVIRKFDFTMPEDTQLTEFNLFLLKGKEQKLLLNVFPRS*
484	CYP5857A7	MSAYSVEDISLYHVLLTIIIGYLGYAYYKNFYYPRHVGPLKSIPGPSNELLLSIKFMHARLTGNSAKFYKDLHAEYGPICHSGLNVVAISDPEATKLIYTSNKYLKEHRYNCLHENVPNIFS<br>TCDRKYHSIRRRMISPVFNWKSIIQLEPQISLHVDNTITAINEYLNNGHNQVDVYELFHKSIADVISDLVIGRCFNSLKKPNFPAFKLSTQIAHSWGLKSTVPMLSFLKSNHHPMINKNIVE<br>ELKERRAGKYRQDILQSLVDAKDAETNSTFNDEEIIEEVSILIFAGMETTAVALIWTFYLLGKNPYSYEKLVDELVTAFPDKNTKISYDMCKDLPYLTAVINESLRVKSPSGILLPRIVPEG<br>GATICGHFLPEGTVVGSSANGIHLSEKNFKNADTFVPERWLGLEAQKLKDKLFTFSLGARRCAGQNLAWLEMHITLANIIRQYDFSMPADAELTGFDLFVLKGKEQKLLLNVFPRSN*
485	CYP5857A8	MSLYSVDCITVYHILLTAVVGYLGFAYYKNFHYPRHIGPLKSIPGPSNELLLTIKFLYARLTGNPAKFYKDLHAEYGPICHSGMGVIAISDPESTKIIYSSYRFKKDYSYELCYENVQNIFS<br>TCDRSYHSIRKRMISPAFNWKSIIQLEPQISLHVVDNTITAINECLDNGCNQVDVYELFHKSIADAISDLVIGRCFNSLKNPNFPAYKISSHIADSWGLKSTVSGLDFIKSRHHPIINQNIT<br>KELKERRAGKYRKDILQTLVDAKDTETNSTFNDVEIIEEASALIFAGMETTAVAIIWTFYLLGKNPQAYKKLVEELIVTFPDKNTKISYDMCKDLPYLTAVIHESLRIKSPAGVILPRVVPE<br>GGATICGHSIPEGTLIASSANGIHLSEKNFKDSESFIPERWVDPDAEKLKDMLFIFGLGPRGCVGKNLAWLEMYISLANVIRQFDFSMPESTQLTGFDLFVLKGKEEKLLLNATPRSS*
224	CYP5857A-fragment1	MLGFLIEGVTLPRVLLAIIIGYLGYLYYKNFHYPRHVGPLKTIPGPSNELLLTIKFIYARATGVPEKFYKDLHTKYGPICHSGLGLVAISDPEAAKLIYSSYKFEKEYRFEYCIENVQNIFS<br>TCDKKYHSIRKRMISLAFNWKSVIQLESHAAIHCVDNTIFAINEVLDNGNTQVDVYDLFHRSIGDVISDLVIGRCFNSLKKPNFPATKLSVCAPAFRLSEV*
175	CYP5857A-fragment2	IFAGLETTAMGIIWTFYLLGKNPQAYEKLVDELITAFPDKNTKITYDMCKNLPYLTAVIYESLRVKSPSGVVLPRVVPEGGATLCGHFLPEGTVVGSSSAGIHLSEQNFQKADSFLPERWLG<br>SNSDELKNMLFSFGLGPRGCVGKNLAWLEMYITIANIIRQFDFSMPKDTELTE
501	CYP5858A1	MSIYSLLVVILSIIIVRYISNLTRVPQNLQHIKGIPIWSTVYKFIKGVPRIKLADEWSPLFQEHGILRLYFGGKWKLINADAELLKKMSFNPEAFDKLTPNRFCPGILIDKIFGTNVLYSDY<br>HEWKRHRRVINPAFKKGWNLSTFSLCIEELFQRLGDNKKLEDLDIDNLMQRVTVEALGIEIMGIKFDGILSPNQHEFVKNFNYLMRAALTPAYFIFPKLDNSWNPFRFIAYKKLKLVNEYFE<br>DLINKRREIMKQEQDYSASDVLSLLIESNLNDVNGLTNEEVKNNTLIFFLAGQDTTSFTLCATLHLLAEHPEIQEKLRLEVISVLGKDEYINERFQVPTNEQLNRLEFTNAVIQESMRLYPA<br>VTILTNWICKKDYNHNGLMIPKNAIIDSYIYAINRSPKYFKNPNTFDPTRYLAGGIDITKLESNWFSFSVGNRMCLGQSFSIVEQKIVLSSLIRRYIVKPSGGRVPCGRELNVKAPFLLRTD<br>NLRLDFERVLNS*
498	CYP5858A2	MTDLINAYSVSGASALIAYLLLRSTRVPDELKHIPSIPFWPTVYKLATGMTMLEIMEEWIPYFEKYGTVTNFLMGKWKIATGDPELLKKITFDHENFDKLTPNEVVPGNLTDKLFGTNILFS<br>NHKVWKKHRRVVNGAFKKGWNLNVFSECVEELFEKLGDSTVKNADIDELMQRVTVEALGREIMGIKFDGISSPSQNEFVQNFNFVMRSALSPVYFIFPSIDRPWNPLRFEAYEKLKQVNIFF<br>DKLISDRKEMMKQDENYKSKDILSLMIESNMKDLNGLTEEDVRYNTLIFFLAGQDTTSFTLSAVLYLLAEHPEIQEKVRQEVIKVLGENEYVDGKIQVPTNEQLNQLEYLYAVIQETMRLYP

		AVTILTNWVSKNNYNYNGMNLPAGTVIDLYVYAINRNEKYFKNPNTFDPSRFLEGGIDTNKLDSNWFSFGAGNRMCLGQSFTLTQQKIVLSSILRRYRVKHSNVENIGKPLKTRPPFLLRSD<br>NLKLDLERI*
424	CYP5858A3	MGKWKVAVGDPDLLKKITFDHENFDKLTPNEVVPGILIDRLFGTNILFSNHKVWKKHRRVVNSAFKKGWNLNVFSECVEELFQKLGEYTVKNADIDELMQRVTVEALGREIMGIKFDGILSN<br>HNEFVQNFNFLMRSALIPIYSLLPWFDRPWNPLRFEAYEKLKQVNIFFDKLINDRKEMMKQDENYKSKDILSLMIESNMKDLNGLTEEDVRYNTLIFFLAGQDTTSFTLSAVLYLLAEHPEI<br>QEKVRQEVIKVLGENEYIDGKLQVPTNEQLNQLEYLYAVIQETMRLYPAVTILTNWVSKNNYNYNGMNLPAGTVIDIYVYAINRNEKYFKNPNTFDPSRYLEGGIDTNKLDSNWFSFGAGNR<br>MCLGQSFTLTQQKIVLSSILRRYRVKHSNVENIGKPLKTRLPFLLRSDNLRLDLERV*
179	CYP5858A-fragment1	MIDILTLIVYSVSGLSAAGIFSLIWITRVPNHLKHIPSVPLWSSIFKLAMGMPMIDLMEYWMPYFEKYGVWKIMTGDPELLKKIAYDHKSFDKLTVHEVIPGTLLDNVFGINIVFSNHSTWK<br>RHRKIVNSAFKKGWNLSVFSEIVDELFQKMGESGNINVDVEDLMQRVTVEALGREV*
167	CYP5858A-fragment2	LTDQEIKFDTLMFFLAGQDTTSFSLCAALHLLAEHPEIQDRLRSEVLMVLGKEEYIDDKIQTPSQSQLDQLKYMHAVIKETMRLYPAVSILYNWIAKEDHDYNGMLVPKNSIIDTYIYAIHR<br>NPKYFENPSKFDPSRFLSDSGEESKADNFWFGFSTGARMCLGSSF
167	CYP5858A-fragment3	FLAGQDTTTFTICATLHLLAEHPDIQDRVRKEVLNVLGKDEYIAGKFQTPTHEQLSQLEYTYAVMQESMRLYPAVSVLTHRVTQNDIQYKEHIIPKGTLIDTCVYAIHRNPDYFKDPNAFDP<br>SRYLNDGIDTTKFESNWFTFSSGSRICLGAGFSIAQQKILLSNII
164	CYP5858A-fragment4	FFLAGQDTTTFTICATLHLLAEHPEIQERARKEVLNVLGNEEYIDGKFQTPTIEQLNQLEYVNAIMQESMRLYPAVSVITHRLAQKDIQYKEHVIPKGTLVDTCVYAIHRNPDFFKDPNTFD<br>PSRYLNGGLDTTKFESNWFTFSSGGRICLGAGFSLTQQKIIL
166	CYP5858A-fragment5	NGLTDEEVRSNTLAMYLAGQNTTTFTICATLHILAEYPEIQEKMRQEVLSVLGKEEYINGTFQTPTNDQLNQLEYTYAIMQESMRLYPALSLLTYREAQKDVHYKNHIIPKGTIVASCVYAM<br>HRNPEYFKDPNTFDPSRYLNGKIDTTKVDSNWFSFSNGSRICIG
167	CYP5858A-fragment6	FLAGQDTTTLTICAALHLLAEHPEVQEKMRKEVLTVFGKDEYKNGKFQIPTNEQLNKLEYTYAVMQETMRLYPAVSVINHRVALKDIHHRGHTIPAGTLVNTIVYAIHRNPKYFKNPNEFLP<br>SRFLNGNIDTKKFESNWFPFSSGSRVCLGATFSTTQQKIVLSYIL
221	CYP5858A-fragment7	MSKTPSRLRNIKEIPLITIIRELSSGAIFIQVMDNIWVIWDANSELFGKIANDASSFDKIILHQFFSNSLLDNMMGINIVQSGTKEWKNHRKKERKKTAKDKTAQYNDILSLILASNIKDCN<br>GLTDDKIRQNTLIFFLGGQDTASLSICAALHLLAEHLEIQEIIRKEVLTVLGKDEYKNEKLQIPTNEQLRKLEYTYVAMQETMKLYSALTVINHRVAL*
167	CYP5858A-fragment8	FLAGQDTTTFTICAALHLLAEHPEIQEKMRQEVLAVFGKDEYETGKFQVPTHDQLNKLEYTHAVMKETMRLYPSVSVILHREAQKDIHHRGHIIPAGTIIDTVVYAIHRNPEYFKNPNEFIP<br>SRFLNGEIDTNKFESNWFAFSSGSRACIGAGFSTVEQKVVLSYIL
503	CYP5859A1	MNTINDLLPNSLESILLDWRLWTSIMAVGLTGFSYWYYTFIHIPHTYGPLSKIPGEGWPLWGRIKLNWHTVTGSRPIYIHGLHRKYGPTVLIGAHMVSCIDKKDVDKIYSTYQFPKADEYKT<br>YNVYGGDNIFSTRKRNFHALRRRVITPIVANSNLDIMEEVVYKTGTLALIDNIEKTFKKTSVIDLFDIFHRSTVDVIFELFFGSGINCLKNTYLPLVWILKVASQFLAIRPVIPFIGVFGDI<br>LISISVAFINLQWYIKPPTKGDHTVISQFLNAKDPETGKQLTWTEVTREMIVIIVGGMDTTAITLAWTFWLMLKHPEVYKEVQKEISEVFPNKDMAPCADTCKSNLPITEAVVLESMRMYPV<br>SAEYLPRTAPVGGTHLGGYEIPGDTLVFCSPYAYQRNPELWDKPDEFNYKRWLGPNSTELKSQLMVFSQGPRGCPGRALAWTEMFLITVALAQRYDMKLCEGQDHLGDRPLTYFLTKPASYS<br>LKAELTHRPQVSGS*
503	CYP5859A2	MNVISDLMPNKSESILLDWRLWTSMAAVAVTGFSYWYYTFIHIPHTQGPLSKIPGEGWPLWGRVKLNWHIIAGSRPRYIHSLHRKYGPTVLIGAHMVSCTAKRDVDKIYSTYQFPKAAEYKT<br>YNIYGGENIFSTRKREFHALRRRVITPIVANSNLDLMEEVVYKVGTLALVDNIEKKLKNTQVIDLFDVFHRSTIDAIFELFFGSGINSLKNTYLPVILTLEVGSFLLALSSVIKFISVFSDL<br>IINLVVAFIFLQWYIKPPINGDHTVISQFLNAKDPETGKQLTWTEVAREMIVIVVGGMDTTAVTLSWTFWLMLRYPEVYKEVQKEVDEIFPNKDIAPSADTCKSSLPITEAVALESMRMYPV<br>SGEYLPRTAPVSGTHLGGYEIPGDTLVFCSPYAYQRNPELWENPDEFDYKRWLGPNSTELKSQLMVFSQGPRGCPGRALAWTEMFLVTVSLAQRYDMKLCKGQDHLGDKPVSYFATKPRSYS<br>LKAEFTHRSQASSS*
158	CYP5859A-fragment1	LSRKETIREVLVIIIGGMDTTAITCSWAFWLILSNPEVYKKAQEEVDKVFPDKNIPIDVTTCKTKMPIIEAILLETMRLYPVSAMPLIRNPPATGTTLSGYEIPPNTNVFMTNYAYHRNPEL<br>WENPDKFDYTRWLIDDNTQLKSQLMVFSQGPRGCPG
127	CYP5859A-fragment2	MNPEAYKSIQIETDIIFPNRDMPLCPETCRTMMPITKDLKSQIMIFSQGPRGCLGRALTWMEIFFIITVMAQRYNFKLCPGQEKLGNKPAIYFMMKSSTCSIKVEIVHRERLNFLTLSHLLT<br>KSIY*
108	CYP5859A-fragment3	MVFSASYAYHRNPEIWDNPDDFNYKRWLIEDNTKLKSQNMVFSQGPRGCPGRALAWMEMFFITTAIAQRYNFRLCPGQEKLGNKPIAYFMMKPSTYSIKVEMEHRKN*
511	CYP5860A1	MNILESLNNYTYYVDNKLSIWVTSLLLIYLSYKVTKFIYNVYLHPLHNIPGPKYLLFSSIFTSYYQLTGKLPEKFNQWHTQYGPVIRTGHNRISLIDQRASKLVYTNQKFIKSKLTELLAKI<br>FGKNLFTLIDRNEHSKRRRIASQAFSQSNLDNMEPQIWQTGVLELLKKLDTKCEANELVNFIDEFHYLTVDIIGELAFGQQFNMIQNLEHELMDHIKNSTLMMVLHLSLPFLQYVRVPFLTK<br>FYNAVEYKNEFARKAVELKKASNSGKKDIMHYLLEARDSETGEKLTDKQLQDEAIILLFAGVDSTASTMSLCIYNLLKYPKVYASLLEELDRVKPFDQETGEIKPYLWCRENLPYLEAVIYE<br>TLRLTPAFPLGVPRVVPEQGTTVGDYYLPESTEVSVSIYSYQRSSQYFSNPNEFQPERWLDKTDTSIKDSIYAFGKGSRVCIGKHLAWMEITLTLIGILTKFELKPLPAWDKVKGDDEIINL<br>VHFKPSSGRISCRLSKRETNTL*
511	CYP5860A2	MNVLESLNSSTYYIDNRVSVWITSLLLAYSGYKVTKFVYNAYIHPLRNIPGPKHLLFSSIFQTYHQLTGKLPEKLNEWHTQYGPVIRLGPNQISVIDQRASKLVYTNQKFQKSKFTEYLARI<br>FGKNLFTLIDRTEHSKRRRIVSHAFSQSNLDNMQPQIWQTGVLELLKKLDTKCESYELVNFIDEFHYLTVDVIGELAFGQQFNMIQNLEHELMDHIKNSTLMMVLYMSFPFLQYVRVPFLSK<br>FYNAIEYKNEFAKKAVELKKASNSDKKDIMHYLLEARDSETGEKLTDRQLQDEAIVILFAGVDSTASTMSLCIYNLLKYPKVYANLLEELDRVKPFDQETGEIKPYLWCRENLPYLEAVVYE<br>TLRLTPAFPFGVPRVVPEQGATVGDYYLPENTEVSVSIYSYQRSSQYFSNPDEFHLERWLDKTDPSIKDSIYAFGKGSRVCIGKHLAWMEITLTLIGILTKFELKPLPVWDKVQGDDEIINF<br>VHFKPTSGRISCRLSKRETSSL*
438	CYP5860A3	NTLLFIYNVYFHPLSNVPGPKHLILSSIVKVYYQFRGTLQLNINRWHNLYGPVIRLGVNEISVIDQYYSTEIYCNQNYKKSKLMELLGVMFGKNLFTIRDRVEHINRKKMVCEAFNRRSLIR<br>MEAVIWEDGVKELLNKLENNTEEGQIVNLASEFHNLTFDVIGKLIFGQHFNLLANHSHQLIDNFNNFNIMLIMLTTFPFLRYIRVPFLSKFYNAIDYKNDFAKKAVKLKKLSNSREEDIMDH

		YLEARDPETDTKLTDKELQDETMILLFSGVDTTASTISMCIYHLLKYPNVYFNVLEELDRVKPVDEVTGKIRSFSWLKENIPYLGAVIYETIRLYPAFAFGVPRLVPEGGATAAGYYLPSDT<br>EVSVSIFSYQRSAQYFENPDEFIPSRWLRNSKDELKHSIIAFGRGSTTCIGKQLALIQITLTLVGLLTRFEL
423	CYP5861A1	ILLALIFIIYKLWNLLHVPQNIAHIPAVPLYKTFIATLSKMTFEEKRSYLYKDLLNEHGIARYFFRSKWTILIGDAHLANEIYLNADKFPKLLSTQMFPESLAAKFIGDNVVFSNDKLWKKY<br>RASVNSAFKIQFDTRLFIEGVSELIQVIDNNAGSEIDMSHYFRRLTMEVLGLGLMNIKFNSILTPNPEFNYHYNNIMKCLNIPITLIFQSLDTPSNPMMRSAYDSEPKDILSLMLLSMDKDE<br>KLTDKEIRDNIMMFVLAGHDTTALALTVAIYFLCKHPNFQDKARREALAIFGKPGNDKKLPFIDSIIKESLRMYPPVAFLPARLTKSEFIANDIQIPASSLILVETFHIQNSSKYWQNPSEF<br>NPYRFYESSNSTKIAYPATSWSPFGGGERMCSGLGFSMMEQRITLALLLLNYKFELN
226	CYP5861A-fragment1	MEKGDKLTDKEIRDNIMMFVMAGHDTTALSLVVAIHFLCKHPNYQDQARREALTVFGKPGNDKKVSISHEQLKQLPFIDSIIKESLRMYPPVAFLPPRVNKNELIVNGIHIPKHSIIVVETF<br>HIQNSTKYWQNPSEFNPYRFFESSDSAKSPHPATSWSPFGAGERMCSGSGFSMMEQRISLALLLLNYELEFSQESKRDSPLDYLCNSTLSSEGVIVNFNEILY*
151	CYP5861A-fragment2	AGHDTTAFALAVGLYFLCKNPEIQEKARQEALAVFGKPGADKRVPITHEQLKQIPFIESIIKEAMRYYPSVSRLPSRINQVELEVNGITIPKNQILTLEMIHFSRSAKYWENPDEFDPYRFY<br>NTTTKDMKHSFAWSTFGGGQRLCIGKDFS
308	CYP5862A1	MLGSRLSEDEEVIDAFYNFPVKVEHSVFSKLKYAQYPIVGRLYAKYLLRKEKVHLPYHELFFNKISEEVAARKSEQSVKEYQKPNDLLQRLLDEGFNTEDITLFVVNLIFASVVTTTNNSTL<br>ALYDMVTYPEYYNELYEEQLQIKKEFGDDISPDIVSKMLKLNSFIRETMRFRLEAVSSFRITRSDWILSNGVIIPKGSFIAVDSTSLHFDNELQDGSPYEFKPYRHFENGKLATKTEPQFLS<br>FGFGTHACPGRFLAIQEISTILSVIIRNYEISTQDGKPHPYMVKPAQFDLPKGEPLIFRKINN*
234	CYP5862A-fragment1	RKVEENAEGYQKPNDLLQRMYDDGYTIEQISSIMLSLAFASVMTTTNNSTISFYDMVTYPEYYNELYEEQLEVEKEFGDVINAEAVSKMVKLNSFIRESMRFRLAGVTNFRFNKNDWTLTNG<br>ITIPKGSLMGVDAFSLHFDNELQDGSPYEFKPFRHAANGKLATKVEPQNIAFGLGQHACPGRFLAIQEISTILSVIIRNYKVSTQDGKPHPYKVVPDQFALPKGEPLIFTKI
500	CYP5863A1	MSLVQSVITNSSKLLESLNLNNAIAILVVIAIFVAAITYQTDPNALPIPSRRLPIVGHLPFLGQFPYIVLHIWSKIYGKVLQIQLGMKSLIVISEFSTIKKLYNLDEFNLRHEPAVFKKHFN<br>SKGITFNSGDNWHSAKKLNIKILKDVLGRESAQTVILEQVKLIKNCIESGNPVDLVDVINPALNNIMVNTLFGFQFNWNDAKFQSLLHNLKSILKLSNHASWASSFPILEKLPLLGFDKPSK<br>DLDLHFTQLFQVINNWVKELETGEISVSNTNNYTLNYLKQVEENYDREQLAASLANLLMGGTETNSATMRWAFVFLLENSHILENVQNELKALNKDLITWEDRSKLHYLQAFLMEVQRVGCV<br>TTLGGSTLRVAGENCKIGDYQINKGAFIAANVYSIHYNEDYFSDPYKFNPERFLKDGELIIRSELIPFGMGKRRCPGEGLAKMEMFLIMANILNAFTFDAVDNSKIDSEINFTAGSVRSPLP<br>YELVFKTKERV*
477	CYP5864A1	MLLYLSLLLIIYITYIYIYQTYFSPLKDVPGPWYLRIHWIPERLMRQTGNFHRYITEYHRQYGPVVRTAPSLVSATSQEAVKSVLSNKKYIKSDFVQKLRDMIGSNILTIQPSDEHLARKKM<br>VLKSIKSMETELNQVIGNFGLDRMSEKWRNKEGSFKVNLINEFNFLVTDIIGALAFNQSFDCINSEKNELMSLTTKTIWYFAIRNITPWIFKLPIGPLKTLELAHIQLKEIAKKSLEASHNP<br>SNNISLIQQYKQTSQQIGDELTEEDLISESLILLIGGVDSTASGLSWLFYYLDQYPKVYLKLAYEIRSKKIDLKGLNVNEMKKEYPYLNKVIMESLRLSPPFATSFPRLVPESGLMIDGVYL<br>PPKTQIAINPYPTMRSPQNFSNPDEFKPDRWSEASGTGLSEPFIGFSKGTRMCVGKDFALRVLFLVTIEILSKFDLHCDYEQEKRDESVCQVTLLPTSGKFELEFQAINA*
284	CYP-fragment	MVRYGANNIKLSPSYLNKKEKLVDNLIGKYSRKISGKVDLNDLATRFSFEVISNILLDNSCEEMYDDFVSLANSATIVKLLVPTPFSTPEIGRIHERIISMKSTFYTELLELFDNKPKLAAK<br>AVSFIMLAGFETASTGIICAVHRLAFDQDLQEKVRTDQTLIPKLVSLSLFANPPLMQNFNMLTTSNIEIGDGIVLPKNTRVNIFINAVHNYPDFSMDNNADSFCTFSGGQRRCVGEGLARIE<br>IRAFIKLMLNFRFKTDDRLVPNSESAITAKPERIFIGVF*

Table S3. Multiple sequence alignment of *C. coronatus* P450s. P450 characteristic motifs EXXR and CXG and their variants were highlighted in yellow and grey background colors, respectively.

```
CLUSTAL O(1.2.4) multiple sequence alignment


CYP5854A-fragment5(6317)Conco1          ------------------------------------------------------------   0
CYP5856B-fragment6(2022)Conco1          ------------------------------------------------------------   0
CYP5862A1(43654)Conco1                  ------------------------------------------------------------   0
CYP5862A-fragment1(25548)Conco1         ------------------------------------------------------------   0
CYP-fragment(71373)Conco1               ------------------------------------------------------------   0
CYP5855A3(13529)Conco1                  -MSSLID---KVLEFWS-NTSYTELILVILVYQFIKFYY--KDIYKVHYSGKIAHLPQPH   53
CYP5855A-fragment1(11397)Conco1         MVSELAA---NIKQFWG-TVSYTEIVVLALVFSFVWLYY--SSIWLGFYTGPHVHLPRVK   54
CYP5855A1(44820)Conco1                  ------------------------------------------------------------   0
CYP5855A2(30387)Conco1                  ------------------------------------------------------------   0
CYP5855A4(43538)Conco1                  ------------------------------------------------------------   0
CYP51F14(41890)Conco1                   ------------------------------------------------------------   0
CYP5859A-fragment2(11509)Conco1         ------------------------------------------------------------   0
CYP5859A-fragment1(29099)Conco1         ------------------------------------------------------------   0
CYP5859A-fragment3(168491)Conco1        ------------------------------------------------------------   0
CYP5859A1(11907)Conco1                  -MNTINDLLPNSLESILLDWRLWTSIMAVGLTGFSYWYY--TFIHIPHTYGPLSKIPGEG   57
CYP5859A2(11511)Conco1                  -MNVISDLMPNKSESILLDWRLWTSMAAVAVTGFSYWYY--TFIHIPHTQGPLSKIPGEG   57
CYP5860A3(24597)Conco1                  ---------------------------------------NTLLFIYNVYFHPLSNVPGPK   21
CYP5860A1(168890)Conco1                 -MNILES-L-NNYTYYV-D---NKLSIWVTSLLLIYLSYKVTKFIYNVYLHPLHNIPGPK   53
CYP5860A2(12313)Conco1                  -MNVLES-L-NSSTYYI-D---NRVSVWITSLLLAYSGYKVTKFVYNAYIHPLRNIPGPK   53
CYP5863A1(2687)Conco1                   -MSLVQSVITNSSKLLE-S---LNLNNAIAILVVIAIFV--AAITYQTD---PNALPIPS   50
CYP5857A2(2640)Conco1                   ------M-LNYSTGDIT-L---SHIALAAFIGYLGYSYY--INFYYSRHIGPLKSIPGPS   47
CYP5857A4(2643)Conco1                   ------------------------------------------------------------   0
CYP5857A-fragment1(12733)Conco1         ------M-LGFLIEGVT-L---PRVLLAIIIGYLGYLYY--KNFHYPRHVGPLKTIPGPS   47
CYP5857A3(2641)Conco1                   ------M-LGFLIEGVT-L---PHILLAIIIGYLGYFYY--KNFHYPRHVGPLRSIPGPS   47
CYP5857A7(2648)Conco1                   ------M-SAYSVEDIS-L---YHVLLTIIIGYLGYAYY--KNFYYPRHVGPLKSIPGPS   47
CYP5857A6(2646)Conco1                   ------------------------------------------------------------   0
CYP5857A-fragment2(24936)Conco1         ------------------------------------------------------------   0
CYP5857A8(2649)Conco1                   ------M-SLYSVDCIT-V---YHILLTAVVGYLGFAYY--KNFHYPRHIGPLKSIPGPS   47
CYP5857A1(14904)Conco1                  ------M-SSYSIEDLS-L---LHVGVTVLLGYLGYVYY--KNFHYPRHVGPLRSIPGPS   47
CYP5857A5(2645)Conco1                   ------------------------------------------------------------   0
CYP5855B1(4344)Conco1                   ------M-LDYSSSMNL-A---FSLLLVLLIVYNIYRFY--YWIIYPYYLGPYKNVPRVQ   47
CYP5855B-fragment1(4348)Conco1          ------M-MDYNSSINV-A---FSLLIAVLIVYNIYNFY--YWIIYPYYLGPYKNIPRVQ   47
CYP5864A1(18764)Conco1                  ------------------------MLLYLSLLLIIYITY--I-YIYQTYFSPLKDVPGPW   33
CYP5855-fragment1(2292)Conco1           ------------------------------------------------------------   0
CYP5855C1(34338)Conco1                  ----------------M-Y---PEVILIFIIGFLSYFSY--KYVIYPYYLGPLSYLPRPK   38
CYP5855C2(2240)Conco1                   ----------------M-Y---KEIFILTIIFYLSRLVY--KYIIYPYYLGPLKNIPRPA   38
CYP5855C3(2245)Conco1                   -MSLDLL-TNNELANII-R---SHTILATFSAILLYLIY--KVIVYPYYLGPLRNLPRPK   52
CYP5855C4(21824)Conco1                  -------------------------ILATLSAVLFYFLY--KVIVYPYYLGPLRNLPRPK   33
```

```
CYP5855C5(2262)Conco1                -MISSFL---ISYLPSN-Y---FQIIGIAIVGIIGYLFY--SVIIYPFYLGPLKNLPRPS   50
CYP5855C9(2252)Conco1                -MSVVEF-SNIPPTGNY-Y---LHAFGVSLLALIIYILY--FNVIYPYYLGPLRNLPRPK   52
CYP5855C6(27512)Conco1               ------------------------------------------------------------   0
CYP5855C-fragment3(34299)Conco1      ------------------------------------------------------------   0
CYP5855C-fragment1(34389)Conco1      ------------------------------------------------------------   0
CYP5855C7(34382)Conco1               -MSLEVT-PSLSSGTYF-N---LQTFGALSIALILYAVY--KTVIYPYYLGPLRNLPRPK   52
CYP5855C8(2259)Conco1                -MSLEVT-PSLSSGTYF-N---LQTFGALSIALILYAVY--KTVIYPYYLGPLRNLPRPK   52
CYP5855D7(2257)Conco1                ------------------------------------------------------------   0
CYP5855D1(29975)Conco1               ------------------------------------------------------------   0
CYP5855D-fragment5(29026)Conco1      ------------------------------------------------------------   0
CYP5855D-fragment7(2254)Conco1       -------------MSYS-I---LKIIVTIIIVLLTYFAY--AKIIYPYYLSPLRNLPRNP   41
CYP5855C-fragment2(30530)Conco1      ------------------------------------------------------------   0
CYP5855E1(38687)Conco1               ------------------------------------------------------------   0
CYP5855D6(6708)Conco1                -----------MLEYLT-G---IGIFGFIICGLLAYFYY--AAILYPYYLGPLRNIPRPK   43
CYP5855D-fragment1(169043)Conco1     ------------------------------------------------------------   0
CYP5855D-fragment6(24158)Conco1      ------------------------------------------------------------   0
CYP5855D4(54364)Conco1               -MLE-DL-INKAGEVIG-S---IGTLGYFLSALAAYFYY--SRILYPYYLSPLRNLPRPK   51
CYP5855D5(76752)Conco1               ------------------------------------------------------------   0
CYP5855D-fragment2(2269)Conco1       ------------------------------------------------------------   0
CYP5855D3(54372)Conco1               -MSS-RL-V--SFIELK-N---FGTIGYVVSALFAYFAY--SSIIYPYYLGPLRNIPRGK   49
CYP5855D-fragment3(28450)Conco1      ------------------------------------------------------------   0
CYP5855D8(45649)Conco1               ------------------------------------------------------------   0
CYP5855D2(2268)Conco1                ---------------MM-S---LSTLAYLIPVALIYLCY--SKIIYPYYLGPLKNIPRGK   39
CYP5855D-fragment4(27931)Conco1      ------------------------------------------------------------   0
CYP5858A-fragment7(4474)Conco1       ---------------------------------------------MSKTPSRLRNIKEIP   15
CYP5856B-fragment1(2011)Conco1       ----------------------MSLISISIILLLILCIY--LHRKITTPPKFAKNLPVIP   36
CYP5858A-fragment1(68816)Conco1      ------------------MIDILTLIVYSVSGLSAAGIF--SLIWITRVPNHLKHIPSVP   40
CYP5858A-fragment2(31161)Conco1      ------------------------------------------------------------   0
CYP5858A-fragment5(21198)Conco1      ------------------------------------------------------------   0
CYP5858A-fragment3(23423)Conco1      ------------------------------------------------------------   0
CYP5858A-fragment4(23037)Conco1      ------------------------------------------------------------   0
CYP5858A-fragment6(21524)Conco1      ------------------------------------------------------------   0
CYP5858A-fragment8(27368)Conco1      ------------------------------------------------------------   0
CYP5858A1(68836)Conco1               ----------------------MSIYSLLVVILSIIIVR--YISNLTRVPQNLQHIKGIP   36
CYP5858A2(77667)Conco1               ------------------MTDLINAYSV--SGASALIAY--LLLRSTRVPDELKHIPSIP   38
CYP5858A3(36631)Conco1               ------------------------------------------------------------   0
CYP5861A-fragment2(21280)Conco1      ------------------------------------------------------------   0
CYP5861A1(30625)Conco1               -----------------------------ILLALIFIIY--KLWNLLHVPQNIAHIPAVP   29
CYP5861A-fragment1(36094)Conco1      ------------------------------------------------------------   0
CYP5856A4(2008)Conco1                ----------------------MLYLLICCFIVALG------VLYLIKVPENFKNVPRVS   32
CYP5856B2(45436)Conco1               ----------------------MIQLLIILGCITAF------LAYLFKVPKHLQSFPRVS   32
CYP5856B-fragment7(34125)Conco1      ------------------------------------------------------------   0
CYP5856B3(76636)Conco1               ------------------------------------------------------------   0
CYP5856B-fragment3(29023)Conco1      ------------------------------------------------------------   0
CYP5856B-fragment4(76635)Conco1      ------------------------------------------------------------   0
```

```
CYP5856A1(34083)Conco1                ----------------------MLYAILFIVILASI------YFSVNKVPTEFKGIPKVS   32
CYP5856A2(34094)Conco1                ------------------------------------------------------------   0
CYP5856A3(29445)Conco1                ------------------------------------------------------------   0
CYP5856B-fragment9(30415)Conco1       ------------------------------------------------------------   0
CYP5856B-fragment10(35455)Conco1      ------------------------------------------------------------   0
CYP5856B-fragment8(44763)Conco1       ------------------------------------------------------------   0
CYP5856B-fragment11(169617)Conco1     ------------------------------------------------------------   0
CYP5856B-fragment12(27598)Conco1      ------------------------------------------------------------   0
CYP5856B1(167158)Conco1               ------------------------------------------------------------   0
CYP5856B-fragment2(34058)Conco1       ------------------------------------------------------------   0
CYP5856A-fragment1(26325)Conco1       ------------------------------------------------------------   0
CYP5856B-fragment5(30225)Conco1       ------------------------------------------------------------   0
CYP5854A-fragment4(78559)Conco1       ------------------------------------------------------------   0
CYP5854A-fragment2(6332)Conco1        -----------------------MYLIAITLIIITYYSY--KWYNFYKLPEEWRDLPSIP   35
CYP5854A-fragment3(6331)Conco1        ------------------------------------------MYLVTAIPEVWKGIPVIP   18
CYP5854E-fragment3(14082)Conco1       ------------------------------------------------------------   0
CYP5854E-fragment5(9574)Conco1        ------------------------------------------------------------   0
CYP5854E-fragment4(9575)Conco1        ------------------------------------------------------------   0
CYP5854E5(14156)Conco1                ----------------------MLQYIA--VGVATFAVY--KAYNIFKCPDELKQLYAAP   34
CYP5854E-fragment2(42529)Conco1       ------------------------------------------------------------   0
CYP5854D1(9752)Conco1                 ----------------------MLTYII--TGLGLATVY--KVSKAIKCPKELEKVTSMP   34
CYP5854E-fragment13(126715)Conco1     ------------------------------------------------------------   0
CYP5854E8(11612)Conco1                ----------------------MFLNII--LIPIAYIAY--RVYQWGKCPSELKDLPSIG   34
CYP5854E-fragment17(6815)Conco1       ------------------------------------------------------------   0
CYP5854E12(104896)Conco1              ----------------------MLKYLG--TTLIAYISY--KVYNWTSCPEEIKHLPALR   34
CYP5854E15(12851)Conco1               ----------------------MIQYLF--SVLIAYATY--KIYKYTRCPDEIKHLPSLP   34
CYP5854E16(80744)Conco1               ----------------------MILYLV--AALVVYISY--KIYTWTRCPDEIKHLPSLP   34
CYP5854E17(80468)Conco1               ------------------------------------------------------------   0
CYP5854E13(77675)Conco1               ------------------------------------------------------------   0
CYP5854E14(12850)Conco1               ----------------------MIQYLS--ATLIAYLSY--KIYNLTRCPNEIKHLPSLP   34
CYP5854E7(11610)Conco1                ----------------------MLQYLA--TVLIAYASY--KIYNWTRCPDEIKHLPALP   34
CYP5854E-fragment16(8906)Conco1       ------------------------------------------------------------   0
CYP5854A3(78558)Conco1                ------------------------------------------------------------   0
CYP5854A1(154349)Conco1               -----------------------MYLIAILIVAITYLVL--KWRNFYKKPKEWEHIPSVS   35
CYP5854A2(17189)Conco1                ----------------------MMYLIAASVVVVTYFTL--KWLNIYKKPKEWKYIPTIS   36
CYP5854A-fragment1(6337)Conco1        ------------------------------------------------------------   0
CYP5854B1(5669)Conco1                 ----------------------MNCFLI--PLLSIYSGY--KLYKILKCPPELQNIPSLD   34
CYP5854B2(5666)Conco1                 ----------------------MYEWIL--SLLSIYSGY--KIYKIFKCPPELAHIPRLD   34
CYP5854B3(78236)Conco1                ------------------------------------------------------------   0
CYP5854B(38119)Conco1                 ------------------------------------------------------------   0
CYP5854C1(140971)Conco1               ------------------------------------------------------------   0
CYP5854E-fragment15(30483)Conco1      ------------------------------------------------------------   0
CYP5854E10(11616)Conco1               ------------------------------------------------------------   0
CYP5854E-fragment14(126716)Conco1     ------------------------------------------------------------   0
CYP5854E-fragment1(11608)Conco1       ----------------------------------MFAAY--RLYRLFKVPPELKDIPAAP   24
```

```
CYP5854E6(11609)Conco1                 ----------------------MLYYFF--FGLLAYIFY--KLYIFARCPDELKHLPAAP   34
CYP5854E-fragment6(12533)Conco1        ------------------------------------------------------------   0
CYP5854E-fragment7(8022)Conco1         ------------------------------------------------------------   0
CYP5854E9(11613)Conco1                 ------------------------------------------------MNTASSD--LYP   10
CYP5854E-fragment12(13308)Conco1       ------------------------------------------------------------   0
CYP5854E-fragment10(13854)Conco1       ------------------------------------------------------------   0
CYP5854E-fragment11(13446)Conco1       ------------------------------------------------------------   0
CYP5854E2(13461)Conco1                 ----------------------MIYYII--LGLVIYTVY--KIINFVRVPPELKNIPAVP   34
CYP5854E-fragment8(12835)Conco1        ------------------------------------------------------------   0
CYP5854E4(14118)Conco1                 ----------------------MIYYFT--LGLVIYAAY--KINKLLKVPPELKNIPALP   34
CYP5854E11(11719)Conco1                ----------------------MIIYLI--LGIFIFIAY--KIYNFFKVPPELKDIPSVP   34
CYP5854E1(13460)Conco1                 ------------------------------------------------------------   0
CYP5854E3(12836)Conco1                 ------------------------------------------------------------   0
CYP5854E-fragment9(11720)Conco1        ------------------------------------------------------------   0


CYP5854A-fragment5(6317)Conco1         ------------------------------------------------------------   0
CYP5856B-fragment6(2022)Conco1         ------------------------------------------------------------   0
CYP5862A1(43654)Conco1                 ------------------------------------------------------------   0
CYP5862A-fragment1(25548)Conco1        ------------------------------------------------------------   0
CYP-fragment(71373)Conco1              ------------------------------------------------------------   0
CYP5855A3(13529)Conco1                 YPLYHHFYR-YYLRIFGDIA-DYAKDHEKYGSV-------FVSASNV---VLTSNLEAFK   101
CYP5855A-fragment1(11397)Conco1        YPLYWYFYK-FYLRIYGDVS-AYSKIHDEHGKV-------ILFAKNG---VMISDLNAFK   102
CYP5855A1(44820)Conco1                 -------------------------MHKENGKV-------FLFVPNA---VLVSDINAFK   25
CYP5855A2(30387)Conco1                 ------------------------KIHETYGKV-------VCTAKNV---ALVSDIESYK   26
CYP5855A4(43538)Conco1                 --------------------------HKTYGKV-------ILIDKNS---VLISDIKAYK   24
CYP51F14(41890)Conco1                  ------------------------------------------------------------   0
CYP5859A-fragment2(11509)Conco1        ------------------------------------------------------------   0
CYP5859A-fragment1(29099)Conco1        ------------------------------------------------------------   0
CYP5859A-fragment3(168491)Conco1       ------------------------------------------------------------   0
CYP5859A1(11907)Conco1                 -WPLWGRIKLNWHTVTGSRPIYIHGLHRKYGPT-------VLIGAHM---VSCIDKKDVD   106
CYP5859A2(11511)Conco1                 -WPLWGRVKLNWHIIAGSRPRYIHSLHRKYGPT-------VLIGAHM---VSCTAKRDVD   106
CYP5860A3(24597)Conco1                 HLILSSIVKVYYQFRGTLQL-NINRWHNLYGPV-------IRLGVNE---ISVIDQYYST   70
CYP5860A1(168890)Conco1                YLLFSSIFTSYYQLTGKLPE-KFNQWHTQYGPV-------IRTGHNR---ISLIDQRASK   102
CYP5860A2(12313)Conco1                 HLLFSSIFQTYHQLTGKLPE-KLNEWHTQYGPV-------IRLGPNQ---ISVIDQRASK   102
CYP5863A1(2687)Conco1                  -RRLPIVGH--LPFLGQFPYIVLHIWSKIYGKV-------LQIQLGMKSLIVISEFSTIK   100
CYP5857A2(2640)Conco1                  -NELWQSIRFMYYRLSGKPREVYKELHAQYGPI-------CHSGMSV---ISISDPEATK   96
CYP5857A4(2643)Conco1                  ------------------------------------------------------------   0
CYP5857A-fragment1(12733)Conco1        -NELLLTIKFIYARATGVPEKFYKDLHTKYGPI-------CHSGLGL---VAISDPEAAK   96
CYP5857A3(2641)Conco1                  -NELLLSIRFMYARLTGNPAKFYKDLHAKYGPI-------CHSGLGL---VAISDPEATK   96
CYP5857A7(2648)Conco1                  -NELLLSIKFMHARLTGNSAKFYKDLHAEYGPI-------CHSGLNV---VAISDPEATK   96
CYP5857A6(2646)Conco1                  ------------------------------------------------------------   0
CYP5857A-fragment2(24936)Conco1        ------------------------------------------------------------   0
CYP5857A8(2649)Conco1                  -NELLLTIKFLYARLTGNPAKFYKDLHAEYGPI-------CHSGMGV---IAISDPESTK   96
CYP5857A1(14904)Conco1                 -NELLLSIRFMYARLTGNPAKFYKDLHAKYGPI-------VHSGLGV---VAVNDPEATK   96
```

```
CYP5857A5(2645)Conco1                ------------------------------------------------------------   0
CYP5855B1(4344)Conco1                -NGLVHYMKLYYHRLLGSPN-YYLQLSNDHGPV-------VHLVKNW---VLVNDPILKK   95
CYP5855B-fragment1(4348)Conco1       -NGLVHYMKLYYHRLLGSPN-YYLQLSNDHGPV-------VHLVKNW---VLVNDPILKK   95
CYP5864A1(18764)Conco1               YLRIHWIPERLMRQTGNFHR-YITEYHRQYGPV-------VRTAPSL---VSATSQEAVK   82
CYP5855-fragment1(2292)Conco1        ------------------------------------------------------------   0
CYP5855C1(34338)Conco1               -NAFSYIYNLYTSKLKGDCE-YLLNLNLEHGPV-------VHLLGSI---VLLSDTLMKK   86
CYP5855C2(2240)Conco1                INPFKYVFYLYKKRFQGDVQ-HLVDLSKEYGPV-------VHLVGNV---VLLNDISMRK   87
CYP5855C3(2245)Conco1                -NTLKHIYEVYKNKSKGNTE-YFLQLSLKYGPV-------VHLYSNT---VLLNNLSYKK   100
CYP5855C4(21824)Conco1               -NTLKHIYEVYKNKSKGNTE-YFLQLSLKYGPV-------VHLYSNT---VLLNNLSYKK   81
CYP5855C5(2262)Conco1                -NPVKYIYFLYTQKLKGDCE-HVLQLSLNYGPV-------VHYFGNV---VLLNNLEYRK   98
CYP5855C9(2252)Conco1                -NVIKYLYDIITKRIEGDVE-HQLLFNLKYGPI-------VHFHGDT---VLLNGQSFRK   100
CYP5855C6(27512)Conco1               -------------------------LSLKYGPV-------VHLFNNV---VLLNNLEYRK   25
CYP5855C-fragment3(34299)Conco1      ------------------------------------------------------------   0
CYP5855C-fragment1(34389)Conco1      ------------------------------------------------------------   0
CYP5855C7(34382)Conco1               -NVIKYMYELYTKRLEGDAE-HLLQYSLKYGPV-------VHYFGNV---VLLNDLSFKK   100
CYP5855C8(2259)Conco1                -NIIKYMYELYTKRLGGDAE-HLLQYSLKYGPV-------VHYFGNV---VLLNDLSFKK   100
CYP5855D7(2257)Conco1                -------------MMLGKMD-VHLDLVQRYGSI-------VHVKDNM---VLVQNPNLKK   36
CYP5855D1(29975)Conco1               ------------------------------------------------------------   0
CYP5855D-fragment5(29026)Conco1      ------------------------------------------------------------   0
CYP5855D-fragment7(2254)Conco1       -NAIKHWFKNLHHTITGRLD-INLQLIQAYGPV-------VHIEDNY---VLIQDNDFRK   89
CYP5855C-fragment2(30530)Conco1      ------------------------------------------------------------   0
CYP5855E1(38687)Conco1               ----------------------------------------------M---VLVNDLNARK   11
CYP5855D6(6708)Conco1                -NGFWHFINHIRNELTGNPN-TYIELSLKYGPI-------VHLRDKL---VLINNSDIRK   91
CYP5855D-fragment1(169043)Conco1     ------------------------------------------------------------   0
CYP5855D-fragment6(24158)Conco1      ------------------------------------------------------------   0
CYP5855D4(54364)Conco1               -SGLWHYYSYLRQVFQGNAS-VNMDLSLKYGPI-------VHVKDKI---VLVNDPIIRK   99
CYP5855D5(76752)Conco1               ----------MRRTFAGDAA-KNMDLCLEYGPV-------VHLRDKL---VVINDDSIKK   39
CYP5855D-fragment2(2269)Conco1       ------------------------------------------------------------   0
CYP5855D3(54372)Conco1               -NAFWHYIAYMYDYITGDPA-KHLKLIQKYGPV-------VHVRNNL---VVINDASVRQ   97
CYP5855D-fragment3(28450)Conco1      ------------------------------------------------------------   0
CYP5855D8(45649)Conco1               ----------------------------------------------M---VLISDPEIRK   11
CYP5855D2(2268)Conco1                -NAFKHYFSYIYDLIVGDAS-SHLKLAQKYGPV-------VHLVDET---VLVNDPQIRK   87
CYP5855D-fragment4(27931)Conco1      ------------------------------------------------------------   0
CYP5858A-fragment7(4474)Conco1       ---LITII---RELSSGAIFI--Q------------------VMDNI-WVIWDANSELFG   48
CYP5856B-fragment1(2011)Conco1       ---FLHTI---YAAFTNKTVD--YRFQNIEWPKLKEKGFAVMYGPIG-WCIYVSKPDFAK   87
CYP5858A-fragment1(68816)Conco1      ---LWSSI---FKLAMGMPMI--D-LM-EYWMP-------YFEKYGV-WKIMTGDPELLK   82
CYP5858A-fragment2(31161)Conco1      ------------------------------------------------------------   0
CYP5858A-fragment5(21198)Conco1      ------------------------------------------------------------   0
CYP5858A-fragment3(23423)Conco1      ------------------------------------------------------------   0
CYP5858A-fragment4(23037)Conco1      ------------------------------------------------------------   0
CYP5858A-fragment6(21524)Conco1      ------------------------------------------------------------   0
CYP5858A-fragment8(27368)Conco1      ------------------------------------------------------------   0
CYP5858A1(68836)Conco1               ---IWSTVY---KFIKGVP--RIK-LADEWSPLFQEHGILRLYF-GGKWKLINADAELLK   86
CYP5858A2(77667)Conco1               ---FWPTVY---KLATGMT--MLE-IMEEWIPYFEKYGTVTNFL-MGKWKIATGDPELLK   88
CYP5858A3(36631)Conco1               ---------------------------------------------MGKWKVAVGDPDLLK   15
```

```
CYP5861A-fragment2(21280)Conco1        ------------------------------------------------------------   0
CYP5861A1(30625)Conco1                 ---LYKTFI---ATLSKMT--FEEKRSYLYKDLLNEHGIARYFF-RSKWTILIGDAHLAN   80
CYP5861A-fragment1(36094)Conco1        ------------------------------------------------------------   0
CYP5856A4(2008)Conco1                  ---ILSTLH---SIITRKT--VQEHHSKILAPKLSKSGFAIVWV-RGGWSLHISKPSAIK   83
CYP5856B2(45436)Conco1                 ---FFGLVN---TIFTGRS--LDEHYKKVLDPALKEKGYAAQFW-LGKWNVVVTQPQIAK   83
CYP5856B-fragment7(34125)Conco1        ------------------------------------------------------------   0
CYP5856B3(76636)Conco1                 ------MVK---TLFTQKS--ADEYYIKVLAPALKHRGYASMLW-RSNWIVVVSKPEIVS   48
CYP5856B-fragment3(29023)Conco1        ------------------------------------------------------------   0
CYP5856B-fragment4(76635)Conco1        ------------------------------------------------------------   0
CYP5856A1(34083)Conco1                 ---LWSFIR---SIQKEDT--LQEHYHGIIEPAIKERGYALVFL-KSSWSLAVTRSDIIK   83
CYP5856A2(34094)Conco1                 ------MLQ---SIRKGET--LQEHYHSIIEPVIKERGYALVFN-RGHWSFTTTRPEVIK   48
CYP5856A3(29445)Conco1                 ------------------------------------------------------------   0
CYP5856B-fragment9(30415)Conco1        ------------------------------------------------------------   0
CYP5856B-fragment10(35455)Conco1       ------------------------------------------------------------   0
CYP5856B-fragment8(44763)Conco1        ------------------------------------------------------------   0
CYP5856B-fragment11(169617)Conco1      ------------------------------------------------------------   0
CYP5856B-fragment12(27598)Conco1       ------------------------------------------------------------   0
CYP5856B1(167158)Conco1                -------MK---AALTGKT--IHYRFKEVVWPKLREKGYAIILG-PFGWNVHIAKPAVAK   47
CYP5856B-fragment2(34058)Conco1        ------------------------------------------------------------   0
CYP5856A-fragment1(26325)Conco1        ------------------------------------------------------------   0
CYP5856B-fragment5(30225)Conco1        ------------------------------------------------------------   0
CYP5854A-fragment4(78559)Conco1        -------------MLTNPAP-MDTVFSNNILPYTEKIGIAVNLTLDRGQTLYVTDPECLK   46
CYP5854A-fragment2(6332)Conco1         ---FWKRLH---RMLTKPVP-INIAFTKHTLPKMEKLGIAVSCGIDKGQTLFISDPECLK   88
CYP5854A-fragment3(6331)Conco1         ---FWERIH---RTLTKPVP-IDVSFSKYMLPFMQKLGIAVYCSVNKGYTLFISDPECLK   71
CYP5854E-fragment3(14082)Conco1        ------------------------------------------------------------   0
CYP5854E-fragment5(9574)Conco1         -------------------------MDRHYQPYLNKHGIIRVFT-PFGWSLFVGDAKLCK   34
CYP5854E-fragment4(9575)Conco1         ------------------------------------------------------------   0
CYP5854E5(14156)Conco1                 ---LLSFIF---FTLDMGC--YRDKIDRYYQPYLNKHGIIRVLT-PVGWCLYVGNARLCK   85
CYP5854E-fragment2(42529)Conco1        ------------------------------------------------------------   0
CYP5854D1(9752)Conco1                  ---MSSFFT---FVSSQES--FDDKMKKDFQPVMDANGIIRVFG-MTGWCLMISDYAIIK   85
CYP5854E-fragment13(126715)Conco1      --------------------------MVAFQPLFNEYGVVRAFTVFWGWSLIVGSPEVAK   34
CYP5854E8(11612)Conco1                 ---LIEFIK---SIVSKGS--YPDKFKL-IQEKLNEHGIIRIFHISYGWSVMIGNPKLAK   85
CYP5854E-fragment17(6815)Conco1        --------------------------------MFDEYGLVRAFT-HFGWSVFIADPKLCK   27
CYP5854E12(104896)Conco1               ---YWAFFH---FVFSKDS--FDDKCRKDFQPMFDKYGVVRAFT-PFGWGVFIGDPKLCK   85
CYP5854E15(12851)Conco1                ---LWSFFT---LSLSNES--LPEKMKKEFQPMFDKYGVVRAFT-HFGWSVFIADPKLCK   85
CYP5854E16(80744)Conco1                ---FWSYFR---LSLSDES--FTEKMKKEFQPMFDEYGIVRAFT-HFGWSVLISDPKLCK   85
CYP5854E17(80468)Conco1                -------------------------MQKEFQPLIDEHGVIRVFT-HLGWTLFIGSPSACK   34
CYP5854E13(77675)Conco1                ------------------------------------------------------------   0
CYP5854E14(12850)Conco1                ---LWSFFC---FSFSNES--FPEKLKKEFQPMFDKYGIVRVFT-HFGWSVFIADSKLC-   84
CYP5854E7(11610)Conco1                 ---MMAFFH---FAFSKDS--LPEKMKKAFQPMFDEYGVVRAFT-HFGWSVFIADPKLCK   85
CYP5854E-fragment16(8906)Conco1        ------------------------------------------------------------   0
CYP5854A3(78558)Conco1                 ------------------------------------------------------------   0
CYP5854A1(154349)Conco1                ---LWGELY---RLFTDPQP-IDVAFEKYTLPILDDFGLATFCDLAKGYTLFVTNPGALK   88
CYP5854A2(17189)Conco1                 ---ALEGLY---RAFTNPVP-IDVDFEKHTLPYIDDNGMTTICDLFSGYSLFVTDPDSLK   89
CYP5854A-fragment1(6337)Conco1         ------------------------------------------------------------   0
```

```
CYP5854B1(5669)Conco1                  ---LSTLLK---ISTTKDG--FDEELKKHVGPLLDKYGIVKYFSHH-GWSVIISDPQLTK   85
CYP5854B2(5666)Conco1                  ---ILTIFK---IASTKDP--FDVEMAKNVGPLLDKHGVVRWFSQH-GWSILVADPQLTK   85
CYP5854B3(78236)Conco1                 ------------MGASKDA--FDDEMAKHMGPMLDKHGIVRYFGPH-GWSIIIADPQLAK   45
CYP5854B(38119)Conco1                  ------------------------------------------------------------   0
CYP5854C1(140971)Conco1                ------------------------------------------------------------   0
CYP5854E-fragment15(30483)Conco1       ------------------------------------------------------------   0
CYP5854E10(11616)Conco1                ------------------------------------------------------ARCLQK   6
CYP5854E-fragment14(126716)Conco1      ------------------------------------------------------------   0
CYP5854E-fragment1(11608)Conco1        ---LMTFIR---YIKDKRS--FGDKVEEYFQSQLNEFGAIRVLTHL-GWTVFIGSPKLCK   75
CYP5854E6(11609)Conco1                 ---LTAFFA---FHANKKLD-FAEKYKKYFAPYLNEHGVVRYLTHT-GWAIYIGEAQMAK   86
CYP5854E-fragment6(12533)Conco1        ------------------------------------------------------------   0
CYP5854E-fragment7(8022)Conco1         ------------------------------------------------------------   0
CYP5854E9(11613)Conco1                 ---L----------------------NF----LFVSCDRDTYLLKSLGWSVFIGSPSIAK   41
CYP5854E-fragment12(13308)Conco1       -------------------------MEYYYQPLLNKYGVVRVLTYE-GWSLFVGDGAIAK   34
CYP5854E-fragment10(13854)Conco1       ------------------------------------------------------------   0
CYP5854E-fragment11(13446)Conco1       ------------------------------------------------------------   0
CYP5854E2(13461)Conco1                 ---LSTFFC---FNLDKRY--YREKVNHYLQSYFNEFGVIRVFTHK-GWSVFIADAKICK   85
CYP5854E-fragment8(12835)Conco1        ------------------------------------------------------------   0
CYP5854E4(14118)Conco1                 ---LLTFFR---FSRDKRF--YIDKMNHYFQSYFNEFGIVRVFTHL-GWTVYIADAKICK   85
CYP5854E11(11719)Conco1                ---FW--------------------VDEYFQSHFNKFGVIRFFTVLGGWNVIIADAKICK   71
CYP5854E1(13460)Conco1                 -----------------------------------------------------------M   1
CYP5854E3(12836)Conco1                 ------------------------------------------------------------   0
CYP5854E-fragment9(11720)Conco1        ------------------------------------------------------------   0


CYP5854A-fragment5(6317)Conco1         ------------------------------------------------------------   0
CYP5856B-fragment6(2022)Conco1         ------------------------------------------------------------   0
CYP5862A1(43654)Conco1                 ------------------------------------------------------------   0
CYP5862A-fragment1(25548)Conco1        ------------------------------------------------------------   0
CYP-fragment(71373)Conco1              -----------------------------------------MVRYGA-NNIKLSPSYLNK   18
CYP5855A3(13529)Conco1                 -LLN-SYQIKKG-RVYSAFDLSN-PTIFS----------SRERQYHAKRKRLISPAFNVK   147
CYP5855A-fragment1(11397)Conco1        -MLN-SYQLKKS-VI-------------------------REREYHSKRKRLLSPAFSVR   134
CYP5855A1(44820)Conco1                 -LLN-SYQLKKS-LLYKAFDINN-PTIFS----------TREKEYHSKRKRLLSPAFSAK   71
CYP5855A2(30387)Conco1                 -FLN-GYKYKKG-KLYSAFDLKN-PNIFS----------SREKEFHSKRKRLLSPAFNAK   72
CYP5855A4(43538)Conco1                 -FLN-GYKFKKG-KIYSTFDFKN-SNIFS----------TRNKEFHSKRKRLLSPAFNAK   70
CYP51F14(41890)Conco1                  ------------------------------------------------------------   0
CYP5859A-fragment2(11509)Conco1        ------------------------------------------------------------   0
CYP5859A-fragment1(29099)Conco1        ------------------------------------------------------------   0
CYP5859A-fragment3(168491)Conco1       ------------------------------------------------------------   0
CYP5859A1(11907)Conco1                 KIYS-TYQFPKA-DEYKTYNVYGGDNIFS----------TRKRNFHALRRRVITPIVANS   154
CYP5859A2(11511)Conco1                 KIYS-TYQFPKA-AEYKTYNIYGGENIFS----------TRKREFHALRRRVITPIVANS   154
CYP5860A3(24597)Conco1                 EIYC-NQNYKKS-KLMELLGVMFGKNLFT----------IRDRVEHINRKKMVCEAFNRR   118
CYP5860A1(168890)Conco1                LVYT-NQKFIKS-KLTELLAKIFGKNLFT----------LIDRNEHSKRRRIASQAFSQS   150
CYP5860A2(12313)Conco1                 LVYT-NQKFQKS-KFTEYLARIFGKNLFT----------LIDRTEHSKRRRIVSHAFSQS   150
CYP5863A1(2687)Conco1                  KLYN-LDEF----------NLRHEPAVFKKHFNSKGITFNSGDNWHSAKKL------NIK   143
```

CYP5857A2(2640)Conco1	LIYS-SHKFKKE-PRYDFFIQNV-SNIFS----------TRDKKFHSIRKRIISPAFNWK	143
CYP5857A4(2643)Conco1	---MISPAFNWK	9
CYP5857A-fragment1(12733)Conco1	LIYS-SYKFEKE-YRFEYCIENV-QNIFS----------TCDKKYHSIRKRMISLAFNWK	143
CYP5857A3(2641)Conco1	LIYS-SYKFEKE-YRFEFCIEN---------------------------KRMISPAFNWK	127
CYP5857A7(2648)Conco1	LIYT-SNKYLKE-HRYNCLHENV-PNIFS----------TCDRKYHSIRRRMISPVFNWK	143
CYP5857A6(2646)Conco1	--	0
CYP5857A-fragment2(24936)Conco1	--	0
CYP5857A8(2649)Conco1	IIYS-SYRFKKD-YSYELCYENV-QNIFS----------TCDRSYHSIRKRMISPAFNWK	143
CYP5857A1(14904)Conco1	TIYG-SYKFIKS-YRFEFFIENV-QNIFS----------TSDRKYHSIRKRMMSPAFNWK	143
CYP5857A5(2645)Conco1	--	0
CYP5855B1(4344)Conco1	-LWS-NYQFPKS-YSYRVFNIYG-PNIFS----------SFDKDFHNFRKKMILPSFTKK	141
CYP5855B-fragment1(4348)Conco1	-LWS-SYQFPKS-YSYRAFNIYG-PNIFS----------SFDRDFHNFRKKMILPSFTKK	141
CYP5864A1(18764)Conco1	SVLS-NKKYIKSDFVQKLRDMIG-SNILT----------IQPSDEHLARKKMVLKS----	126
CYP5855-fragment1(2292)Conco1	--	0
CYP5855C1(34338)Conco1	-CIT-NSKFKKS-SHYESFDFSGKVNLFS----------VRDRDQHSKIKKLVSPAFSLK	133
CYP5855C2(2240)Conco1	-CYM-TYKFKKS-SYYKAFDFSGRGNLLS----------FREKEQHAKIKKLVLPAFSIK	134
CYP5855C3(2245)Conco1	-YWM-TNKFKKS-SFYSAFDIAGLSSLFS----------TTDKDHHSKIKKLVLPAFSQK	147
CYP5855C4(21824)Conco1	-YWM-TNKFKKS-RFYSAFDIAGLSSLFS----------TTNKDHHSKIKKLVLPAFSQK	128
CYP5855C5(2262)Conco1	-YWQ-TYKFKKS-SFYTSFDVGGMPTLFS----------AIEKDYHSKVKKLVLPAFSVK	145
CYP5855C9(2252)Conco1	-YWM-TYKYKKS-EFHRAFDIGGYQTLFS----------ATEKDYHSKIKKLVLPAFSVK	147
CYP5855C6(27512)Conco1	-YWQ-TYKFKKS-VFYTSFDVGGMPTLFS----------AIEKDYHSKVKKLVLPAFSVK	72
CYP5855C-fragment3(34299)Conco1	--	0
CYP5855C-fragment1(34389)Conco1	--	0
CYP5855C7(34382)Conco1	-YWM-TYKFKKS-IFYTTFDMSGNPNLFS----------AIEKDYHSKIKRLVLPAFSVK	147
CYP5855C8(2259)Conco1	-YWM-TYKFKKS-IFYSTLDMSGNPNLFS----------AIEKDYHSKIKRLVLPAFSVK	147
CYP5855D7(2257)Conco1	-HYM-TYKFAKH-PIYKLLDISG-PNLFS----------STDKDFHASRKKLIAPAFSIK	82
CYP5855D1(29975)Conco1	--------------------------LFS----------SRDREFHSRRKKLISSAFSVK	24
CYP5855D-fragment5(29026)Conco1	--	0
CYP5855D-fragment7(2254)Conco1	-IYL-TYKFKKD-PIYSLLDVNG-PNLFS----------SMDKGFHSSRRRLISPAFSLK	135
CYP5855C-fragment2(30530)Conco1	--	0
CYP5855E1(38687)Conco1	-WLL-TYKFNKD-DSYHIFNFRG-PTIFS----------TADKEFHQIRKRLVAPAFSNK	57
CYP5855D6(6708)Conco1	-CYI-NYKFPKA-KVYELLSYNG-PNLFS----------TTSREYHASRKKLILPAFNNK	137
CYP5855D-fragment1(169043)Conco1	--	0
CYP5855D-fragment6(24158)Conco1	--	0
CYP5855D4(54364)Conco1	-SFT-TYKFAKA-ESYSLFSING-PNLFS----------TTDKDFHQRIKKLILPAFNNK	145
CYP5855D5(76752)Conco1	-NYM-TYKFKKL--------------------------------ITTNIKKLILPAFNNK	65
CYP5855D-fragment2(2269)Conco1	--	0
CYP5855D3(54372)Conco1	-YYM-SYKFPKS-KLYSLFNING-PNIFS----------ALNKDFHVKMKKLISPAFNNK	143
CYP5855D-fragment3(28450)Conco1	--	0
CYP5855D8(45649)Conco1	-YYM-SYKCPKA-SYYEVFDFNG-PNLFS----------ALKKDFHVNMKKLLLPAFNNK	57
CYP5855D2(2268)Conco1	-YYM-SYKWPKAASYYKVFDFNG-PNLFS----------ALKKDFHVSMKKLLLPAFNNK	134
CYP5855D-fragment4(27931)Conco1	--	0
CYP5858A-fragment7(4474)Conco1	K---------------------------------------IANDASSFDKIILHQFFSNS	69
CYP5856B-fragment1(2011)Conco1	K---------------------------------------MFKDSNKFIKPIMK--LKGS	106
CYP5858A-fragment1(68816)Conco1	K---------------------------------------IAYDHKSFDKLTVHEVIPGT	103

```
CYP5858A-fragment2(31161)Conco1       ------------------------------------------------------------   0
CYP5858A-fragment5(21198)Conco1       ------------------------------------------------------------   0
CYP5858A-fragment3(23423)Conco1       ------------------------------------------------------------   0
CYP5858A-fragment4(23037)Conco1       ------------------------------------------------------------   0
CYP5858A-fragment6(21524)Conco1       ------------------------------------------------------------   0
CYP5858A-fragment8(27368)Conco1       ------------------------------------------------------------   0
CYP5858A1(68836)Conco1                KMSFNPEAFDKL-TPNR----FCPGILIDKIFGT-NVLYSDYHEWKRHR-RVINPAFKKG   139
CYP5858A2(77667)Conco1                KITFDHENFDKL-TPNE----VVPGNLTDKLFGT-NILFSNHKVWKKHR-RVVNGAFKKG   141
CYP5858A3(36631)Conco1                KITFDHENFDKL-TPNE----VVPGILIDRLFGT-NILFSNHKVWKKHR-RVVNSAFKKG   68
CYP5861A-fragment2(21280)Conco1       ------------------------------------------------------------   0
CYP5861A1(30625)Conco1                EIYLNADKFPKL-LSTQ----MFPESLAAKFIGD-NVVFSNDKLWKKYR-ASVNSAFKIQ   133
CYP5861A-fragment1(36094)Conco1       ------------------------------------------------------------   0
CYP5856A4(2008)Conco1                 QILKNTRVFTKF-RI-H------PASLNRRFTGKRNIVRSDGEEWRRYR-KPVNSIFIQS   134
CYP5856B2(45436)Conco1                QFFRNTTTFHKE-LPNI------HG-LFGKFLGKSNIFFSNGEDWRRHR-KPANPIFHQT   134
CYP5856B-fragment7(34125)Conco1       ------------------------------------------------------------   0
CYP5856B3(76636)Conco1                QLFRNTDRFPKS-MSNF------PG-ILGKYIGPSNILISNGDEWKRHR-KPANPIFHQK   99
CYP5856B-fragment3(29023)Conco1       ------------------------------------------------------------   0
CYP5856B-fragment4(76635)Conco1       ------------------------------------------------------------   0
CYP5856A1(34083)Conco1                QVFRNSQQFTKD-VIRH------HKSLEYKFTGKQNIVRSNGDDWRRFR-RPANPIFNQT   135
CYP5856A2(34094)Conco1                QIFRKTKQFTKY-AIRH------PESLENKFTGQQNIVRSNGDDWRRFR-RPANPIFHQT   100
CYP5856A3(29445)Conco1                --------------------------LEFKFTGEKNIVRSNGDDWRRFR-RPANPIFHQT   33
CYP5856B-fragment9(30415)Conco1       ------------------------------------------------------------   0
CYP5856B-fragment10(35455)Conco1      ------------------------------------------------------------   0
CYP5856B-fragment8(44763)Conco1       ------------------------------------------------------------   0
CYP5856B-fragment11(169617)Conco1     ------------------------------------------------------------   0
CYP5856B-fragment12(27598)Conco1      ------------------------------------------------------------   0
CYP5856B1(167158)Conco1               NLFRNSNQFLKP-VISN------PGSLTELFLGEVNIVGSNGEQWKRLR-RPANPIFGQS   99
CYP5856B-fragment2(34058)Conco1       ------------------------------------------------------------   0
CYP5856A-fragment1(26325)Conco1       ------------------------------------------------------------   0
CYP5856B-fragment5(30225)Conco1       ------------------------------------------------------------   0
CYP5854A-fragment4(78559)Conco1       QIMKNPDIFIKI-PFGE----D-NETLVREYFGVQQVVTTNGEDWKRMR-NVMNPIFNQS   99
CYP5854A-fragment2(6332)Conco1        QIMKNPDVFVKI-PFAV----E-NHTVLREHFGVQQVVTANGEDWRRMR-KVMNPMFNQS   141
CYP5854A-fragment3(6331)Conco1        QVMSNIDVFIKM-PFVG----K-NHTLLRDYFGAQQVVATNGEDWKRMR-KVMNPMFNQS   124
CYP5854E-fragment3(14082)Conco1       ------------------------------------------------------------   0
CYP5854E-fragment5(9574)Conco1        EISTKSDLYLKP-GAD-----CIMSVNMRRFFRKSQVGLSNSKEWKKHR-KIINPIFNQT   87
CYP5854E-fragment4(9575)Conco1        ------------------------------------------------------------   0
CYP5854E5(14156)Conco1                EIASQADIYHRF-NLL-----LIAPVNLRRFLGKSHVGFHNGEEWKKHR-KIINPIFNQT   138
CYP5854E-fragment2(42529)Conco1       ------------------------------------------------------------   0
CYP5854D1(9752)Conco1                 EIASKPEIFCKP-DPTK----QPTNLNSLKFFGKSHVAINNGQEWKRHR-KIINTIFSSK   139
CYP5854E-fragment13(126715)Conco1     EVFVKNNIFAKQ-VFKQ----SFKSSTIEKLFGPSQVVSNNGDEWKRHR-KIINPIFNQT   88
CYP5854E8(11612)Conco1                EVSSKDNIFPKH-DIKK----EVLSPHLKRFLGLSQVLGSVGDEWKRHR-KVVNPIFNQS   139
CYP5854E-fragment17(6815)Conco1       EASAKNDIFAKP-DINN----QPASKYLKKFFGPSQVISNNGDEWKRHR-KVINPIFNQT   81
CYP5854E12(104896)Conco1              EAAALVDVFPKQ-DLKN----APISGLLMKYFGRSQVISNNGDEWKRHR-KVINPIFNQT   139
CYP5854E15(12851)Conco1               EACAKNDIFIKQ-DFKK----APASENFLKFFGPSQVLRNNGTEWKRHR-KVINPIFNQT   139
CYP5854E16(80744)Conco1               EVSVKHEIFVKQ-SFSE----AQFSENLTKFFGVSQVLSNNGAEWKRHR-KVINPIFNQS   139
```

```
CYP5854E17(80468)Conco1             EISNKPDIFTKP-SFED----AKISRHLTSFFGKSQVVSNNGDEWKRHR-KVINPIFTQS   88
CYP5854E13(77675)Conco1             -------------------------------------------EWKRHR-KVINPIFNQT   16
CYP5854E14(12850)Conco1             -------IFIKT-DFTK----IPVSENFMKFFGPSQVLSNNGEEWKRHR-KVINPIFNQS   131
CYP5854E7(11610)Conco1              EACAKPEIFIKT-DFTK----IPVSEHLMKFFGGSQVLSNNGSEWKRHR-KVINPIFNQT   139
CYP5854E-fragment16(8906)Conco1     ------------------------------------------------------------   0
CYP5854A3(78558)Conco1              ------------------------------------------------------------   0
CYP5854A1(154349)Conco1             EIFKNTDIFVKE-PFAE----K-NETLFIEHFGEQQVVNANGEDWKRYR-KIMNPIFNQS   141
CYP5854A2(17189)Conco1              QILKNTDIFVKE-PFAE----K-NEALFKEHFGVQQVVSANGEDWKRMR-KVMNPIFNRS   142
CYP5854A-fragment1(6337)Conco1      ------------------------------------------------------------   0
CYP5854B1(5669)Conco1               LVFNNSDIFQKD-TNSA----INSNPHAVKFFGKQQIVNTNGEDWKRMR-KLMNPIFHKT   139
CYP5854B2(5666)Conco1               VIFNNPDIFEKD-SNSA----INANPHAIKFFGKEQIVNVNGEDWKRMR-KLMNPIFHQT   139
CYP5854B3(78236)Conco1              IIYNNSDVFYKN-TNSV----INLNPHSQKFFGKDQIVNINGEDWKRMR-KLMNPIFHQT   99
CYP5854B(38119)Conco1               ------------------------------------------------------------   0
CYP5854C1(140971)Conco1             -------MPQYE-EFYE----NSDYTNSDKFIGAESMGALNGHDWKRHR-KILLPYFTKP   47
CYP5854E-fragment15(30483)Conco1    ------------------------------------------------------------   0
CYP5854E10(11616)Conco1             IFLSKNDIFIKE-ELKK----F-LSSVSIKLGGPSQVVTNNGEEWKRHR-KVINPIFNQT   59
CYP5854E-fragment14(126716)Conco1   ------------------------------------------------------------   0
CYP5854E-fragment1(11608)Conco1     EVSTLSNIFEKI-VLNK----SKASFNFLRFVGDSQVASTNGQEWKKQR-KIINPIFNQT   129
CYP5854E6(11609)Conco1              EIQMKNDIFEKP-LLTA----S-LSAIFVRFFGLSQVLAINGNEWKRHR-KVINPIFNQT   139
CYP5854E-fragment6(12533)Conco1     ------------------------------------------------------------   0
CYP5854E-fragment7(8022)Conco1      ------------------------------------------------------------   0
CYP5854E9(11613)Conco1              EVLYKSDVFYKP-KFID----SYMSINFKRFLGFSQVVQNNGDEWKRHR-KVINPIFNQT   95
CYP5854E-fragment12(13308)Conco1    EVFSNPDIYCKP-DFKN----ARLSSNLKRFFGLNQALSANGLEWKRQR-KIINPIFNQT   88
CYP5854E-fragment10(13854)Conco1    ------------------------------------------------------------   0
CYP5854E-fragment11(13446)Conco1    ------------------------------------------------------------   0
CYP5854E2(13461)Conco1              EVSILSDVFQKP-EFSK----NVASKHLLRFFGESQVASANGEEWKKQR-KVINPIFNQT   139
CYP5854E-fragment8(12835)Conco1     ------------------------------------------------------------   0
CYP5854E4(14118)Conco1              EVTTLPEIFQKQ-TSSK----N-AADNFLRFFGASQVGSVNGEEWRKQR-KIINPIFNQT   138
CYP5854E11(11719)Conco1             EVSTLSDIFQKP-DFTK----FIPSKLFRKFFGVSQIFSANGAKWKRQR-KVINPIFNQS   125
CYP5854E1(13460)Conco1              SIKPLSDVFQKV-DISK----N-TSKLFLRFFGLSQVASANGEDWKKQR-KVINPIFNQT   54
CYP5854E3(12836)Conco1              ------------------------------------------------------------   0
CYP5854E-fragment9(11720)Conco1     ------------------------------------------------------------   0

CYP5854A-fragment5(6317)Conco1      --------------------------------------MPKAMNSAFNKTWKPELFEECF   22
CYP5856B-fragment6(2022)Conco1      ------------------------------------------------------------   0
CYP5862A1(43654)Conco1              ---------------------MLGSRLSE------DEEVIDAFYNFPVKVE---------   24
CYP5862A-fragment1(25548)Conco1     ------------------------------------------------------------   0
CYP-fragment(71373)Conco1           K---------------EKLVDNLIGKYSR--KISGKVDLNDLATRFSFEVISNILLDNSC   61
CYP5855A3(13529)Conco1              SMLSMEPKIL-KTG-TLNLLEFLNSQFNA-ETKQIKVNIINVFYQSTLDVISELVFGQSL   204
CYP5855A-fragment1(11397)Conco1     QMGLMEPLVL-ETG-TLNLVEHLDSQFNG-EIKNIRVNIYELFYRSTLDVISQLVFGESL   191
CYP5855A1(44820)Conco1              QVGLMESLIL-EMG-TLNLVEHLDSQLNG-EIINIRVNIYELFYRSTLDVISHLVFGESL   128
CYP5855A2(30387)Conco1              SMQAMEPIIL-QTG-TLNLIDYLSNEMELSREAELRVNVLEILYRSTLDVISKLVFGESL   130
CYP5855A4(43538)Conco1              SMQAMESIIL-QTG-TLNLIEYLSNEMELLNASELRVNVLELLYRSTFDVIGKLVFGGSR   128
CYP51F14(41890)Conco1               --------------------------------------ILHDMAKLIIKTASGCLLGHEI   22
```

```
CYP5859A-fragment2(11509)Conco1        ------------------------------------------------------------   0
CYP5859A-fragment1(29099)Conco1        ------------------------------------------------------------   0
CYP5859A-fragment3(168491)Conco1       ------------------------------------------------------------   0
CYP5859A1(11907)Conco1                 NLDIMEEVVY-KTG-TLALIDNIEKTFKK--T--SVIDLFDIFHRSTVDVIFELFFGSGI   208
CYP5859A2(11511)Conco1                 NLDLMEEVVY-KVG-TLALVDNIEKKLKN--T--QVIDLFDVFHRSTIDAIFELFFGSGI   208
CYP5860A3(24597)Conco1                 SLIRMEAVIW-EDG-VKELLNKLENNTEE--G--QIVNLASEFHNLTFDVIGKLIFGQHF   172
CYP5860A1(168890)Conco1                NLDNMEPQIW-QTG-VLELLKKLDTKCEA--N--ELVNFIDEFHYLTVDIIGELAFGQQF   204
CYP5860A2(12313)Conco1                 NLDNMQPQIW-QTG-VLELLKKLDTKCES--Y--ELVNFIDEFHYLTVDVIGELAFGQQF   204
CYP5863A1(2687)Conco1                  ILKDVLGRESAQTV-ILEQVKLIKNCIES--G--NPVDLVDVINPALNNIMVNTLFGFQF   198
CYP5857A2(2640)Conco1                  TIMKLEPMIS-LHC-VENLVDTINKTLDN--G-STQIDIYELIHKSIGDAISDIVLGRCF   198
CYP5857A4(2643)Conco1                  SVLQLEPQIS-LHV-VDNTIAAINEYLAI--G-STQVDVYELFHKSIADAISD-------   57
CYP5857A-fragment1(12733)Conco1        SVIQLESHAA-IHC-VDNTIFAINEVLDN--G-NTQVDVYDLFHRSIGDVISDLVIGRCF   198
CYP5857A3(2641)Conco1                  SVMQLESQVA-LHC-VDNTIFAINEVLDN--G-NTQVDVYDLFHKSIGDASSDLVIGRCF   182
CYP5857A7(2648)Conco1                  SIIQLEPQIS-LHV--DNTITAINEYLNN--G-HNQVDVYELFHKSIADVISDLVIGRCF   197
CYP5857A6(2646)Conco1                  ------PQIS-LHV-VDNTLIAINECLDN--G-SSQIDVYELFHKSIADAISDLVIGRCF   49
CYP5857A-fragment2(24936)Conco1        ------------------------------------------------------------   0
CYP5857A8(2649)Conco1                  SIIQLEPQIS-LHV-VDNTITAINECLDN--G-CNQVDVYELFHKSIADAISDLVIGRCF   198
CYP5857A1(14904)Conco1                 SVMDLEPQVT-VHV-VDNTLTAINEILSN--G-NTQIDVYELFHKSIGDAISDTVLGKCF   198
CYP5857A5(2645)Conco1                  ------PQIS-LHV-VDNTIAAINEYLNI--D-RTQVDVYELFHKSIGDAISDLVIGRCF   49
CYP5855B1(4344)Conco1                  NLKYN-KDIY-TIG-SQNLVDMFKDKISQ--GQSNIFDLYHYFECSALDVITTLSIGHSL   196
CYP5855B-fragment1(4348)Conco1         NLKYN-ELVY-QIG-SQNLVDMFKDKISQ--GHSNIFDLYHYFECSALDVITTLSIGHSL   196
CYP5864A1(18764)Conco1                 -IKSMETELNQVIG-NFGLDRMSEKWRNK--EGSFKVNLINEFNFLVTDIIGALAFNQSF   182
CYP5855-fragment1(2292)Conco1          ------------------------------------------------------------   0
CYP5855C1(34338)Conco1                 TLSSIENIVY-NIG-SQGLVNYIESKMKS--NNQEVFDFYHLFHCSTFDVITQIVFGTNY   189
CYP5855C2(2240)Conco1                  TLSNIEQSIY-DIG-SQGLVNFIQSKMKS--HNQEVFDLYYLFHCSTFDVVTQIVFGTNF   190
CYP5855C3(2245)Conco1                  TLSNIEGTIY-DIA-SQGLVNYIHAELRN--GTTEVFDMFHMFHCSTFDVISELVFGSNF   203
CYP5855C4(21824)Conco1                 TLSNIEGTIY-DIA-SQGLVNYIHAELRN--GTTEVFDMFHLFHCSTFDVISELVFGSNF   184
CYP5855C5(2262)Conco1                  TLANVEKTVY-DIG-SQGLVNYIQSTIKS--GQTDIFDLFHLFHCSTFDVITQLVFGTNF   201
CYP5855C9(2252)Conco1                  TLASVEKTVY-DIG-SQGLVSHIQSVIKS--GQSDVFDLFHLFHCSTFDVITQLVFGTNF   203
CYP5855C6(27512)Conco1                 TLANIEKAIY-DIG-SQGLVSHIQSTIKS--GQTDEFDLYHLFHCSTFDVISELVFGTNF   128
CYP5855C-fragment3(34299)Conco1        ------------------------------------------------------------   0
CYP5855C-fragment1(34389)Conco1        ------------------------------------------------------------   0
CYP5855C7(34382)Conco1                 TLDIIEKTVY-DIG-SEGLVSHIQSVIKS--GQSDAFDLFHLFHCSTFDVITQLVFGTNF   203
CYP5855C8(2259)Conco1                  TLDIIEKTVY-DIG-SEGLVSHIQSVIKS--GQNDVFDLFHLFHCSTFDVITQLVFGTNF   203
CYP5855D7(2257)Conco1                  SLSKMESTIY-RAG-SDSLIKYLHSQLDK--NESKEIDFYPLFNSNTMDIITELIFGESL   138
CYP5855D1(29975)Conco1                 GLSLMEDRIA-SVG-SESLVNYLNQNLMN--EESKEFDLFKLFHYNTLDVISELIFGKNL   80
CYP5855D-fragment5(29026)Conco1        ------------------------------------------------------------   0
CYP5855D-fragment7(2254)Conco1         NLQKMEPTIY-RVG-SDSLIKYIESKLIN--NKAEEIDIFQLFHSNTMDVITELIFGSSL   191
CYP5855C-fragment2(30530)Conco1        ------------------------------------------------------------   0
CYP5855E1(38687)Conco1                 NLAKMEPTIY-KVG-SDHLAQFISSSMGD--EKEVVLDMYNLFHRSTLDVIGELVFGQNL   113
CYP5855D6(6708)Conco1                  ALVTMEPTIY-RVG-SESLVQYLNSCLDS--ETSKEFDFYNLFHCNTLDVISELVFGETL   193
CYP5855D-fragment1(169043)Conco1       ------------------------------------------------------------   0
CYP5855D-fragment6(24158)Conco1        ------------------------------------------------------------   0
CYP5855D4(54364)Conco1                 TLDAMEPTIY-RVG-SESLVQYLDSFMDQ--EPSKEFDLFHLFHTNTLDVISELVFGETL   201
CYP5855D5(76752)Conco1                 TLAAMEPTVY-RVG-SESLVQYLDSFLDN--EPSKEFDLFHLFHTNTLDVISELVFGETL   121
```

```
CYP5855D-fragment2(2269)Conco1         ------------------------------------------------------------   0
CYP5855D3(54372)Conco1                 ALAAMESSIY-KAG-SESLVKYLDSYLDN--EPNREFDFYHLFNCNTFDVITKMVFGTEF   199
CYP5855D-fragment3(28450)Conco1        ------------------------------------------------------------   0
CYP5855D8(45649)Conco1                 SLAAMEDTIY-KVG-SESLVQYIDSHLDE--HSSYEFDILDLCYRNTLDVISELVFGTSI   113
CYP5855D2(2268)Conco1                  SLAAMEDTIY-KVG-SESLVQYIDSFLED--NQSYEFDILDLFYSNTLDVISELVFGSSI   190
CYP5855D-fragment4(27931)Conco1        ------------------------------------------------------------   0
CYP5858A-fragment7(4474)Conco1         LLDNM----------------------------------------------------MGI   77
CYP5856B-fragment1(2011)Conco1         -LGQL--------------------------------------------------FAGDI   115
CYP5858A-fragment1(68816)Conco1        LLDNV----------------------------------------------------FGI   111
CYP5858A-fragment2(31161)Conco1        ------------------------------------------------------------   0
CYP5858A-fragment5(21198)Conco1        ------------------------------------------------------------   0
CYP5858A-fragment3(23423)Conco1        ------------------------------------------------------------   0
CYP5858A-fragment4(23037)Conco1        ------------------------------------------------------------   0
CYP5858A-fragment6(21524)Conco1        ------------------------------------------------------------   0
CYP5858A-fragment8(27368)Conco1        ------------------------------------------------------------   0
CYP5858A1(68836)Conco1                 WNLST----F-SLC-IEELFQRLGD---N--KKLEDLDIDNLMQRVTVEALGIEIMGIKF   188
CYP5858A2(77667)Conco1                 WNLNV----F-SEC-VEELFEKLGD---S--T-VKNADIDELMQRVTVEALGREIMGIKF   189
CYP5858A3(36631)Conco1                 WNLNV----F-SEC-VEELFQKLGE---Y--T-VKNADIDELMQRVTVEALGREIMGIKF   116
CYP5861A-fragment2(21280)Conco1        ------------------------------------------------------------   0
CYP5861A1(30625)Conco1                 FDTRL----F-IEG-VSELIQVIDN---N--A-GSEIDMSHYFRRLTMEVLGLGLMNIKF   181
CYP5861A-fragment1(36094)Conco1        ------------------------------------------------------------   0
CYP5856A4(2008)Conco1                  FCTKM----F-ADC-VKEALSVLEYRVNE--FSGKPVVVSDLMEYMTLDILGKGVFGYDF   186
CYP5856B2(45436)Conco1                 FHPEQ----F-ASS-IEDTFEELMERINQ--SPDEALPVTDLMGLMTLDVLGKGIFSVDF   186
CYP5856B-fragment7(34125)Conco1        ------------------------------------------------------------   0
CYP5856B3(76636)Conco1                 FQPEL----F-SPC-IEEVLNSIMDKVSR--NPEESIQVCDLMSLLTLDVLGKGIFSIDF   151
CYP5856B-fragment3(29023)Conco1        ------------------------------------------------------------   0
CYP5856B-fragment4(76635)Conco1        ------------------------------------------------------------   0
CYP5856A1(34083)Conco1                 FQSDI----F-AES-VKEVLNALTDRVNE--HGSNPFEVSDLMELMTLDILGKGIFDFDF   187
CYP5856A2(34094)Conco1                 FQPEM----F-AEC-VNDVLNVVTDRINE--HGSKPFEVSDLMEFMTLDILGKGIFDFDF   152
CYP5856A3(29445)Conco1                 FQPDI----F-GEC-VNEVLNALTDRINE--NGSKPFEVSDLMEFMTLDILGKGIFDFDF   85
CYP5856B-fragment9(30415)Conco1        ------------------------------------------------------------   0
CYP5856B-fragment10(35455)Conco1       ------------------------------------------------------------   0
CYP5856B-fragment8(44763)Conco1        ------------------------------------------------------------   0
CYP5856B-fragment11(169617)Conco1      ------------------------------------------------------------   0
CYP5856B-fragment12(27598)Conco1       ------------------------------------------------------------   0
CYP5856B1(167158)Conco1                FQPEA----F-SPC-VLETLDALDERVNR--LQGQPIEVKDLMELMTLDVLGKGIFSHDF   151
CYP5856B-fragment2(34058)Conco1        ------------------------------------------------------------   0
CYP5856A-fragment1(26325)Conco1        ------------------------------------------------------------   0
CYP5856B-fragment5(30225)Conco1        ------------------------------------------------------------   0
CYP5854A-fragment4(78559)Conco1        WKPEL----F-GGCRFYQVIEEWDK------LEGKNIDIHDQIQR*--------------   133
CYP5854A-fragment2(6332)Conco1         WKPEL----F-GEC-FNQVIEEWDK------LEGKNILVQDQIQRMTLDVMGKAFFDFDF   189
CYP5854A-fragment3(6331)Conco1         WKPEL----F-GEC-FNQVIEEWDK------LEGKNVLIHDQIQRMTLDVMGKAFFDFDF   172
CYP5854E-fragment3(14082)Conco1        ------------------------------------------------------------   0
CYP5854E-fragment5(9574)Conco1         WSTEL----F-GNC-AQDLIDEYEK------MAGKDVKIHDKIQRMTLDVFGKAIFDVDF   135
CYP5854E-fragment4(9575)Conco1         ------------------------------------------------------------   0
```

```
CYP5854E5(14156)Conco1              WSTKL----F-GTS-IQNLIDELEK------MAGKDVKFYHVI-----------------   169
CYP5854E-fragment2(42529)Conco1     ------------------------------------------------------------   0
CYP5854D1(9752)Conco1               WDLEI----F-ENS-THDLIDILKL------SEGEEIEVYDNIQRLTLDILGKSIFNIDF   187
CYP5854E-fragment13(126715)Conco1   WNTQL----F-GSC-AQDVIDEWTK------EDGKDVKVGDLIQRMTLDVFGKAIFDYNF   136
CYP5854E8(11612)Conco1              WDTKW----K-KQC-N-----------------NSEVNIQDKIQRMTLDVFGKAIFDYDF   176
CYP5854E-fragment17(6815)Conco1     WNTEL----F-GEC-VRDVINDWEK------HAGGEIKIHDNIQRMTLDVFGKAIFDINF   129
CYP5854E12(104896)Conco1            WNTEL----F-GDC-AKDVISEWEK------HVGDEIKVRDIIQKMTLDVFGRAIFDVNF   187
CYP5854E15(12851)Conco1             WNTEL----F-GEC-ARDVINDWEK------HAGGEIKND-------FGCFGKAIFDINF   180
CYP5854E16(80744)Conco1             WNTEL----F-GEC-ARDVINDWEK------HAGGEIKVDDNIQRMTLDVFGKAIFDINF   187
CYP5854E17(80468)Conco1             WSTQL----F-GAC-AHDLINGWEK------QVDKDVKVHDLIQRMTLDVFGRAIFDIDF   136
CYP5854E13(77675)Conco1             WNTEL----F-GEC-VRDVVSEWEK------QAGSEIKVHDIIQRMTLDVFGRAIFDVNF   64
CYP5854E14(12850)Conco1             WNTEL----F-GEC-VRDVISEWEE------QTDKEIKIHDNIQRMTLDVFGKAIFDVNF   179
CYP5854E7(11610)Conco1              WNTEL----F-GEC-AKDVIAEWEK------QAGGEVKVHDTIQRMTLDVFGRAIFDINF   187
CYP5854E-fragment16(8906)Conco1     ------------------------------------------------------------   0
CYP5854A3(78558)Conco1              --------------------------------------------------MGKAFFDFDF   10
CYP5854A1(154349)Conco1             WKPEL----F-GEC-FNQVIEEWDR------LEGKDILIHDQIQRMTLDVMGKAFFDFDF   189
CYP5854A2(17189)Conco1              WKPEL----F-GEC-FNQVIEEWDK------LEGKDILIHDQIQRMTLDVMGKAFFDFDF   190
CYP5854A-fragment1(6337)Conco1      ------------------------------------------------------------   0
CYP5854B1(5669)Conco1               WPIDK----L-SSC-ARDLIDSWSL------TSGENVEIHENIQKLTLDVLGQAVFNMDF   187
CYP5854B2(5666)Conco1               WPIQT----L-SQC-TRDVIDSWSQ------TDGKGIDIRDNIQKMTLDVLGHTVFNMDF   187
CYP5854B3(78236)Conco1              WPIDT----L-SKC-TRDVIDIWSQ------TDGLNVEVHDNIQKLTLDVLGHTVFNTDF   147
CYP5854B(38119)Conco1               ------------------------------------------------------------   0
CYP5854C1(140971)Conco1             WPIEL----F-SNY-CQLVINKWTM---K--GDNQEILLLDDLQRMTLDCLGKGLFDIEF   96
CYP5854E-fragment15(30483)Conco1    ------------------------------------------------------------   0
CYP5854E10(11616)Conco1             WNTRL----F-GSC-AQDLIEEWAK------EDGKEVKVRDLIQRMTLDVFGKAIFDINF   107
CYP5854E-fragment14(126716)Conco1   ------------------------------------------------------------   0
CYP5854E-fragment1(11608)Conco1     WSTEM----F-GNS-VQDLIDEWEK------MEGD-------------------------   152
CYP5854E6(11609)Conco1              FSTEL----F-GNC-ASDLIDEWVK------MEGEE------------------------   163
CYP5854E-fragment6(12533)Conco1     ------------------------------------------------------------   0
CYP5854E-fragment7(8022)Conco1      ------------------------------------------------------------   0
CYP5854E9(11613)Conco1              WSTEL----F-GNF-AQDLIDEWGK------VEGKEVKVQDKVQRLTLDVFSKAIFDIDF   143
CYP5854E-fragment12(13308)Conco1    WSTEL----F-GNC-VKDLIEEWDK------EEGTEVKISDKIQRMTLDVFGKAIFNIEF   136
CYP5854E-fragment10(13854)Conco1    ------------------------------------------------------------   0
CYP5854E-fragment11(13446)Conco1    ------------------------------------------------------------   0
CYP5854E2(13461)Conco1              WSTEL----F-GNC-AQDLIDEWEK------MEGKEFKVRDKIQRMTLDVLGKSIFDMEF   187
CYP5854E-fragment8(12835)Conco1     ------------------------------------------------------------   0
CYP5854E4(14118)Conco1              WSTEL----F-GTC-AQDLIDEWEK------MDGKELKVHDKIQRMTLDVFGKSIFDMEF   186
CYP5854E11(11719)Conco1             WSTEL----F-GNC-IKDLIDEWEK------MDGNEVKIIDKIQRMALDVFGKLIFDIDF   173
CYP5854E1(13460)Conco1              WSTEI----F-GKC-VQDLIDEWEK------MDGKEFKIHDKIQRMALDVFGKYIFDIEF   102
CYP5854E3(12836)Conco1              ------------------------------------------------------------   0
CYP5854E-fragment9(11720)Conco1     ------------------------------------------------------------   0


CYP5854A-fragment5(6317)Conco1      NQAV---------------------EEWYKLEGK--KFS-FMIKF--KAPEYNIFADS--   54
CYP5856B-fragment6(2022)Conco1      ------------------------------------------------------------   0
```

CYP5862A1(43654)Conco1	--------HSV--------FSKLKYAQYPIVGRL--YAKYLLRKEKVHLPYHELFFNKIS	66
CYP5862A-fragment1(25548)Conco1	--	0
CYP-fragment(71373)Conco1	EEMY-DDFVSL-----------ANS---ATIVKLLVPTP-FS------TPEIG-------	92
CYP5855A3(13529)Conco1	NTLSGDASNFK------FYMREVQKIQWVLGMSP--LFP-FLKKY--YSA-GEIFKKMIV	252
CYP5855A-fragment1(11397)Conco1	HCIE-NPEEFK------YYMSVISRIQLFLGLVI--PFP-FLRKF--ERP-SKPFERIVE	238
CYP5855A1(44820)Conco1	HCIE-NPEEFK------HYMKIISNIQKMFSLYP--LFP-ILKRL--ERP-TKPFERIVE	175
CYP5855A2(30387)Conco1	NCID-NPEDFK------YYMKMIFRAQIILGTSP--IIP-FIKRY---LP-TAIFETIIV	176
CYP5855A4(43538)Conco1	NFVN-NPKEFR------NIMKLIFRTQLVLGLIV--MIP-FVKKY--FLP-TDIVGRVIG	175
CYP51F14(41890)Conco1	RAKL-DDSVAD------IYHDLDGGFTPMQFMFEWLPLPSYYKRDAAHKKMSDLFY----	71
CYP5859A-fragment2(11509)Conco1	--	0
CYP5859A-fragment1(29099)Conco1	--	0
CYP5859A-fragment3(168491)Conco1	--	0
CYP5859A1(11907)Conco1	NCLK-NTYLPL--------VWILKVASQFLAIRP--VIP-FIGVF--GDILISISVAFIN	254
CYP5859A2(11511)Conco1	NSLK-NTYLPV--------ILTLEVGSFLLALSS--VIK-FISVF--SDLIINLVVAFIF	254
CYP5860A3(24597)Conco1	NLLA-NHSHQL--------IDNFNNFNIMLIMLT--TFP-FLRYI--RVPFLSKFYNAID	218
CYP5860A1(168890)Conco1	NMIQ-NLEHEL--------MDHIKNSTLMMVLHL--SLP-FLQYV--RVPFLTKFYNAVE	250
CYP5860A2(12313)Conco1	NMIQ-NLEHEL--------MDHIKNSTLMMVLYM--SFP-FLQYV--RVPFLSKFYNAIE	250
CYP5863A1(2687)Conco1	NWND-AKFQSLL-HNLKSILKLSNHASWA----S--SFP-I--LE--KLPLLGFDKPSKD	245
CYP5857A2(2640)Conco1	DSLK-KPDFPA------YKLSIHISNTWG--LKF--TVP-FLNFL--KASHHPIVNENII	244
CYP5857A4(2643)Conco1	---------------------------LG--LKL--TIP-GINFL--KSRHRPIINQSIV	83
CYP5857A-fragment1(12733)Conco1	NSLK-KPNFPA------TKLSVCAPA-FR--LSE--V*----------------------	223
CYP5857A3(2641)Conco1	NSLK-KHDFPA------TKLSGHIANSWG--LKF--ALP-FLGFL--KSRHHPIINQSIA	228
CYP5857A7(2648)Conco1	NSLK-KPNFPA------FKLSTQIAHSWG--LKS--TVP-MLSFL--KSNHHPMINKNIV	243
CYP5857A6(2646)Conco1	NSLK-KPNFPA------YKLSSHIADSWG--LKF--SFP-FLRFL--KSKHHPIIIQNIV	95
CYP5857A-fragment2(24936)Conco1	--	0
CYP5857A8(2649)Conco1	NSLK-NPNFPA------YKISSHIADSWG--LKS--TVS-GLDFI--KSRHHPIINQNIT	244
CYP5857A1(14904)Conco1	NSLK-IPDFPS------YQLCSHIADTWG--LKF--AVP-ALGFL--KSRHHPIINQNIV	244
CYP5857A5(2645)Conco1	NSLK-KPNFPA------YKISSYIADSWG--FKF--TFP-FLRFL--KSKYHPIINQYIL	95
CYP5855B1(4344)Conco1	NTVR-DETMSK------EFFKVWTHTQYVLFLKG--LIP-SISTI--NVPTVERYKYLIE	244
CYP5855B-fragment1(4348)Conco1	NTVR-DETISK------EFFKFWTHTQYVLFLKG--LIP-SISAM--SVPIVERYKYLIE	244
CYP5864A1(18764)Conco1	DCIN-SEKNEL--------MSLTTKTIWYFAIRN--ITPWIFKLP--IGPLKTL----EL	225
CYP5855-fragment1(2292)Conco1	--	0
CYP5855C1(34338)Conco1	ETLS-NEENAQ------KYVNVVAETQKSMFLRS--IVP-FIKYF--AFPIEKLFKEIII	237
CYP5855C2(2240)Conco1	ETTQ-NEENAT------KYVNVVAETQKAMFLRS--MIP-FLDYF--PFPIEKLFKEHII	238
CYP5855C3(2245)Conco1	DTIN-DQQKAK------YYFDIIKKTQKALFYRV--IMP-AYKLL--PLPMEAVLGKIVQ	251
CYP5855C4(21824)Conco1	DTIN-DQQKAK------YYFDIIKKTQKALFMRV--LVP-IYKFL--PLPMEEVLGKIVQ	232
CYP5855C5(2262)Conco1	NTTS-NEDEAD------FYLNGLTGVQKAMFLRI--MIP-LYKLI--SFPMEKLFKNIVF	249
CYP5855C9(2252)Conco1	DTIS-DKDKAI------YYISSLADTQKAVFWRT--IFP-PYKKV--AFPMEKIFKPVIF	251
CYP5855C6(27512)Conco1	NTTL-DEAKAK------YYVYSMGATQKAMFLRT--MIP-LYKLI--PFPMEKLFKNIII	176
CYP5855C-fragment3(34299)Conco1	--MEKLFENHII	10
CYP5855C-fragment1(34389)Conco1	--MEKLFKNIVF	10
CYP5855C7(34382)Conco1	DTIS-DEAKAD------YYISALGDTQKAMFWRT--MIP-FYKLI--PFPMEKLFKPVIL	251
CYP5855C8(2259)Conco1	DTIS-DKAKAD------YYISALGDTQKAVFWRT--MIP-FYKLI--PFPMEKLFKPVIF	251
CYP5855D7(2257)Conco1	NTTW-DKSKSQ------YYSQLTTTSGYNIFLKA--VLP-FYKPL--NSPIE-TFKPRIL	185
CYP5855D1(29975)Conco1	NTTT-DSQSAK------FYFEGIEKTQKLLFMRL--LVP-FSNLI--RLPMESMFKPIIL	128

```
CYP5855D-fragment5(29026)Conco1        ------------------------------------------------------------   0
CYP5855D-fragment7(2254)Conco1         NTTW-DRKKAE------YYNKIIEKTQKTIFLD*--------------------------   217
CYP5855C-fragment2(30530)Conco1        ------------------------------------------------------------   0
CYP5855E1(38687)Conco1                 NCIL-DSKKAD------HYMNELKKTQLLIVLRT--LCPSIFTLF--TYPILTLFEPTIL   162
CYP5855D6(6708)Conco1                  NTTW-DEEKRI------FYIEELSKTIYATLLRA--LVP-FYTYF--THPMEKLFKPMIM   241
CYP5855D-fragment1(169043)Conco1       ------------------------------------------------------------   0
CYP5855D-fragment6(24158)Conco1        ------------------------------------------------------------   0
CYP5855D4(54364)Conco1                 NTTW-DKKKGI------YYIDELAKTQYMAFLRT--IVP-FYNHI--KYPMEKLFMPVIM   249
CYP5855D5(76752)Conco1                 NTTW-DEKKGL------YYIDELAKTQYMMFLRA--VVP-FYNHI--KYPMEKLFMPVIM   169
CYP5855D-fragment2(2269)Conco1         ------------------------------------------------------------   0
CYP5855D3(54372)Conco1                 STTW-DEKKGY------YYRDLLQKTVKGHFYRI--FIP-FYDRL--ELPIETVFKPIIL   247
CYP5855D-fragment3(28450)Conco1        ------------------------------------------------------------   0
CYP5855D8(45649)Conco1                 NATW-DKKKGK------EFIDALANTQFMLFIRY--FVP-FSYLI--KLPMEPILTPMIL   161
CYP5855D2(2268)Conco1                  NATW-DKEKGA------QFMDLLSKSQFMGFIRS--FIP-FSYLF--KLPMEPLLTPMIL   238
CYP5855D-fragment4(27931)Conco1        ------------------------------------------------------------   0
CYP5858A-fragment7(4474)Conco1         NIVQ------------------SGTKEWK-------------------------------   88
CYP5856B-fragment1(2011)Conco1         NIAA------------------SNGEEWK-------------------------------   126
CYP5858A-fragment1(68816)Conco1        NIVF------------------SNHSTWK-------------------------------   122
CYP5858A-fragment2(31161)Conco1        ------------------------------------------------------------   0
CYP5858A-fragment5(21198)Conco1        ------------------------------------------------------------   0
CYP5858A-fragment3(23423)Conco1        ------------------------------------------------------------   0
CYP5858A-fragment4(23037)Conco1        ------------------------------------------------------------   0
CYP5858A-fragment6(21524)Conco1        ------------------------------------------------------------   0
CYP5858A-fragment8(27368)Conco1        ------------------------------------------------------------   0
CYP5858A1(68836)Conco1                 DGIL-SPNQHEFVKNFNYLMRAAL-TPAY----F--IFP-KLDNS--WNPFRFIAYKKLK   237
CYP5858A2(77667)Conco1                 DGIS-SPSQNEFVQNFNFVMRSAL-SPVY----F--IFP-SIDRP--WNPLRFEAYEKLK   238
CYP5858A3(36631)Conco1                 DGIL-S-NHNEFVQNFNFLMRSAL-IPIY----S--LLP-WFDRP--WNPLRFEAYEKLK   164
CYP5861A-fragment2(21280)Conco1        ------------------------------------------------------------   0
CYP5861A1(30625)Conco1                 NSIL-TPNP-EFNYHYNNIMKCLN-IPIT----L--IFQ-SLDTP--SNPMMRSAYD---   226
CYP5861A-fragment1(36094)Conco1        ------------------------------------------------------------   0
CYP5856A4(2008)Conco1                  KAIK-NMGASHYHHLYKTVFNGVQ-SALF----L--YFP-ILEYF--PIFGRSKLHKNCD   235
CYP5856B2(45436)Conco1                 EAVK-SKGSSRYHHLYTSIINQIP-NPVY----F--FFQ-FLHRY--PMGKMAEAHKNIG   235
CYP5856B-fragment7(34125)Conco1        ------------------------------------------------------------   0
CYP5856B3(76636)Conco1                 EAIK-SEGSSKYHQLYYSIMKQVG-SPLY----S--LIP-FLNHY--PVGDMAKAYRSLD   200
CYP5856B-fragment3(29023)Conco1        ------------------------------------------------------------   0
CYP5856B-fragment4(76635)Conco1        ---------------------------------------------------MAKAYENLE   9
CYP5856A1(34083)Conco1                 EAVR-LKGTSKYHHIYNSAFSGVF-DPLY----L--LLP-FIEKL--KLKKRMKKHHDAI   236
CYP5856A2(34094)Conco1                 EAVK-LQGTSRYNHIYNSVFSGIA-DPIY----L--TFP-ILEKL--KLKNRVQKYEDAN   201
CYP5856A3(29445)Conco1                 EAVK-LKGTSQYNHIYDSAFSGIF-DPLY----L--LFP-FLEKL--KLQKRMQKHIDAI   134
CYP5856B-fragment9(30415)Conco1        ------------------------------------------------------------   0
CYP5856B-fragment10(35455)Conco1       ------------------------------------------------------------   0
CYP5856B-fragment8(44763)Conco1        ------------------------------------------------------------   0
CYP5856B-fragment11(169617)Conco1      ------------------------------------------------------------   0
CYP5856B-fragment12(27598)Conco1       ------------------------------------------------------------   0
CYP5856B1(167158)Conco1                EAVK-SLGNSNYHKLYKGIMSFLF-NPFF----V--IVR-PLAKL--SFGPPERARKNIN   200
```

```
CYP5856B-fragment2(34058)Conco1        ----------------------------------------------------MRTRKKII   8
CYP5856A-fragment1(26325)Conco1        ------------------------------------------------------------   0
CYP5856B-fragment5(30225)Conco1        ------------------------------------------------------------   0
CYP5854A-fragment4(78559)Conco1        ------------------------------------------------------------   133
CYP5854A-fragment2(6332)Conco1         EAVK-NPESELY-QLYHNVVSGLFKNIAY----I--IFP-ILDHI--PFMKRHELYNN--   236
CYP5854A-fragment3(6331)Conco1         EAVK-NPESELY-QLYHNLVSGLYKKIIY----R--LFP-ILDRI--PYLKRYELFDR--   219
CYP5854E-fragment3(14082)Conco1        ------------------------------------------------------------   0
CYP5854E-fragment5(9574)Conco1         KSVK-NPSSKLY-NLYHKIFEQLFGQPVY----L--LFP-FMEYM--PFFRRTELSHQLD   184
CYP5854E-fragment4(9575)Conco1         ------------------------------------------------------------   0
CYP5854E5(14156)Conco1                 -NSK-NPNSKIY-NLYDSIFKQLFGQPVY----L--LFP-FMEYV--PFFRRTKLSHQLD   217
CYP5854E-fragment2(42529)Conco1        ------------------------------------------------------------   0
CYP5854D1(9752)Conco1                  KSLK-DPDSKLY-STYTYIINRLFNEPMY----F--IMP-FLEHL--PYFKRSDLSRKID   236
CYP5854E-fragment13(126715)Conco1      NVII-LNLK*--------------------------------------------------   144
CYP5854E8(11612)Conco1                 KAVK-EQDSELY-NLYVSIFDGTF-NPLY----S--VFP-FLDNL--PYFKRPEVSKKLD   224
CYP5854E-fragment17(6815)Conco1        NAVK-GKSSRLY-HLYNDIVEKISGQIIY----L--IAP-FIENI--PYFSRTKLRQQIN   178
CYP5854E12(104896)Conco1               NAVK-GNESRLY-VLYNSIIGQAFGQVLY----L--FAP-FMEHV--PFFRRPKLSKEID   236
CYP5854E15(12851)Conco1                KAVE-DKSSRLY-HLYNDIIEKVLGQAIY----N--IAP-FMEYM--PFFIRTKLRKQLN   229
CYP5854E16(80744)Conco1                KSVE-DKSSRLY-HLYNDITEKVLGQAMY----I--VAP-FMDNM--PYFSRPKLRQQLN   236
CYP5854E17(80468)Conco1                KAVK-DPDSKLF-HLYNDITEEMFSNPLY----I--LIG-FLDDL--PYIGRTKLENQIK   185
CYP5854E13(77675)Conco1                DAVK-DKSSRLY-NLYNDITDKIAGQALY----L--VAP-FMEHV--PYFRRHKLAQQLN   113
CYP5854E14(12850)Conco1                EAVI-DKSSRIY-HLYNDITDKISGQTLY----L--IAP-FMEYI--PYFRRPKLRQQIN   228
CYP5854E7(11610)Conco1                 NAVK-DKASRLY-HLYNDITQQCFGQALY----L--IAP-FMEHV--PYFRRPNLGKQLD   236
CYP5854E-fragment16(8906)Conco1        ------------------------------------------------------------   0
CYP5854A3(78558)Conco1                 QAVK-NSKSELY-QLYHNIVNGIFKDVSY----L--LFP-IIDHI--PFFKRYELHKKVA   59
CYP5854A1(154349)Conco1                ESVK-NPESKLY-KIYHDITSGLFSNILY----L--VLP-VLDHI--PYLKRYDLHAKVA   238
CYP5854A2(17189)Conco1                 EAVR-NPESKLY-KLYHDITNGLFKDIAY----L--IFP-ILDHI--PYLKRYALHAKVA   239
CYP5854A-fragment1(6337)Conco1         ------------------------------------------------------------   0
CYP5854B1(5669)Conco1                  ECIK-NPQSKLY-NQYHEISTQLFANLSY----L--FFP-VLDKL--PYFKRPELYKQID   236
CYP5854B2(5666)Conco1                  ESVK-NPDSKLY-RIYHTASSELMGKLAY----L--FFP-ILEKL--PFFKRPELHKQID   236
CYP5854B3(78236)Conco1                 ESVK-NPDSELY-NRYHTIAKDVFGQIIY----F--FFP-ILDKV--PYFKRPKLQSLIS   196
CYP5854B(38119)Conco1                  ------------------------------------------------------------   0
CYP5854C1(140971)Conco1                NASI-EPNNRLF-HLWNTLNT-KSNSPWY----E--TFP-ILDQL--PLFKRPEFDDEIL   144
CYP5854E-fragment15(30483)Conco1       ------------------------------------------------------------   0
CYP5854E10(11616)Conco1                DV----------------------------------------DDL--KLFRRTELDNKLN   125
CYP5854E-fragment14(126716)Conco1      ------------------------------------------------------------   0
CYP5854E-fragment1(11608)Conco1        ------------------------------------------------------------   152
CYP5854E6(11609)Conco1                 -AVK-NAKVKLY-TLYAYIAKEIFGKQLY----I--MFS-ILEYL--PFTRRPKLAQNLK   211
CYP5854E-fragment6(12533)Conco1        ------------------------------------------------------------   0
CYP5854E-fragment7(8022)Conco1         ------------------------------------------------------------   0
CYP5854E9(11613)Conco1                 KSIK-NEDSKLF-NLYHKVSKQIIDYPVY----I--IMP-FLEYL--PFTRRPELAKKLG   192
CYP5854E-fragment12(13308)Conco1       KSVK-NANSKLY-TLYTDIFEQLFSNPVY----L--FFP-FLEHT--PFFKRPKLTKDLD   185
CYP5854E-fragment10(13854)Conco1       ---------MLY-KLYHGIFEELLGSPIY----F--LFS-FLENI--PFFRRPELSRKID   41
CYP5854E-fragment11(13446)Conco1       ---------MLY-KLYHGIFEELLGSPLY----F--LFP-FLENI--PFFKRPELSRKIE   41
CYP5854E2(13461)Conco1                 KSVK-NADSKLY-SLYHDIAEDIFGHPIY----F--LFP-FLEYV--PFFKRPELSRKLD   236
CYP5854E-fragment8(12835)Conco1        ------------------------------------------------------------   0
```

```
CYP5854E4(14118)Conco1               KSVK-NADSKLY-NLYHDIAKEVFGHPFY----I--LFP-FLEKV--PFLKRPQLANKLD   235
CYP5854E11(11719)Conco1              KSVK-NDDSKLY-NLYHYISEQMFGNVIY----I--MFP-FLEYV--PFFRRPELSNKLN   222
CYP5854E1(13460)Conco1               KSVK-NNDSKLY-NLYHSISEELFAHPIY----I--LFP-FLENI--PFFRRPELSDKIE   151
CYP5854E3(12836)Conco1               ------------------------------------------------------------   0
CYP5854E-fragment9(11720)Conco1      ------------------------------------------------------------   0


CYP5854A-fragment5(6317)Conco1       --------LIKRRKADK--------DSLFTKFVQASIS-SNSQE---AA-----------   83
CYP5856B-fragment6(2022)Conco1       ------------------------------------------------------------   0
CYP5862A1(43654)Conco1               -------EEVAARKSEQS----VKEYQKPNDLLQRLLD---------EG-----------   95
CYP5862A-fragment1(25548)Conco1      ------------RKVEEN----AEGYQKPNDLLQRMYD---------DG-----------   24
CYP-fragment(71373)Conco1            --------RIHERIISM-------KSTFYTELL-ELFD--NKPK----------------   118
CYP5855A3(13529)Conco1               -------ENMESRRLEK--------SK-NTDILQSLMD-TQDGEVDGGGSG---------   286
CYP5855A-fragment1(11397)Conco1      -------ENMRKRRENK--------SS-HSDIL---------------------------   255
CYP5855A1(44820)Conco1               -------ENMRKRRENK--------SS-HSDILQSMMD-TQDED---SGIK---------   206
CYP5855A2(30387)Conco1               -------ENMKIRRLEK--------TQ-HSDILQSMLD-TQDEN---TGKK---------   207
CYP5855A4(43538)Conco1               -------QIINKRRLEK--------SQ-HWDILQSMID-TQKG----LDLK---------   205
CYP51F14(41890)Conco1                -------DIVQERRKSE---------KQNDDVVDALMR--NSYK---DG-----------   99
CYP5859A-fragment2(11509)Conco1      ------------------------------------------------------------   0
CYP5859A-fragment1(29099)Conco1      ------------------------------------------------------------   0
CYP5859A-fragment3(168491)Conco1     ------------------------------------------------------------   0
CYP5859A1(11907)Conco1               -------LQWYIKPPTK---------G-DHTVISQFLN-AKDPE---TGKQ---------   284
CYP5859A2(11511)Conco1               -------LQWYIKPPIN---------G-DHTVISQFLN-AKDPE---TGKQ---------   284
CYP5860A3(24597)Conco1               YKNDFAKKAVKLKKLSN--------SR-EEDIMDHYLE-ARDPE---TDTK---------   256
CYP5860A1(168890)Conco1              YKNEFARKAVELKKASN--------SG-KKDIMHYLLE-ARDSE---TGEK---------   288
CYP5860A2(12313)Conco1               YKNEFAKKAVELKKASN--------SD-KKDIMHYLLE-ARDSE---TGEK---------   288
CYP5863A1(2687)Conco1                -------LDLH-----------------FTQLFQVINNWVKELE---TGEISVSN--TNN   276
CYP5857A2(2640)Conco1                -------EELKLRRNG----------KKREDILQVLVD-SKDYE---TNST---------   274
CYP5857A4(2643)Conco1                -------EELKECKAG----------KYRKDILQTLVD-AKDTE---TNST---------   113
CYP5857A-fragment1(12733)Conco1      ------------------------------------------------------------   223
CYP5857A3(2641)Conco1                -------EELKERRAG----------KYRKDILQSLID-AKDTE---TNST---------   258
CYP5857A7(2648)Conco1                -------EELKERRAG----------KYRQDILQSLVD-AKDAE---TNST---------   273
CYP5857A6(2646)Conco1                -------EELKERRDG----------KYRQDILQTLID-ANDAE---TNSA---------   125
CYP5857A-fragment2(24936)Conco1      ------------------------------------------------------------   0
CYP5857A8(2649)Conco1                -------KELKERRAG----------KYRKDILQTLVD-AKDTE---TNST---------   274
CYP5857A1(14904)Conco1               -------EELKERRAG----------KYRKDILQTLVD-AKDTE---TNST---------   274
CYP5857A5(2645)Conco1                -------EELKERRAG----------KYRKDILQTLVD-AKDTE---TNST---------   125
CYP5855B1(4344)Conco1                -------QSVKYRQSEN---------IQFDDTLQSLMD-GQDPQ---TGEN---------   275
CYP5855B-fragment1(4348)Conco1       -------QSIKRR*----------------------------------------------   250
CYP5864A1(18764)Conco1               -------AHIQLKEIAK----------------KSLEA-SHNPS---NNISLIQ------   252
CYP5855-fragment1(2292)Conco1        ------------------------------------------------------------   0
CYP5855C1(34338)Conco1               -------ENIKLRENS----------P-NPDILQSLID-SEDPE---TGEKLTHEEIAQE   275
CYP5855C2(2240)Conco1                -------ENIKLRENN----------P-NPDILQSLID-SEDPE---TGE----------   266
CYP5855C3(2245)Conco1                -------QNIDLRVNN----------P-KSDILQSFID-SENQE---TGEK---------   280
CYP5855C4(21824)Conco1               -------QNIDLRVNN----------H-KSDILQSLLD-SEDPE---TGEK---------   261
```

```
CYP5855C5(2262)Conco1                 -------DNIKLRENN----------P-SSDILQSLID-SQDSE---TGEK---------   278
CYP5855C9(2252)Conco1                 -------ENIKLRENN----------P-NSDILQSLID-SIDPE---TGEK---------   280
CYP5855C6(27512)Conco1                -------ENIKLRENN----------P-SPDILQSLID-SQDPE---TGEK---------   205
CYP5855C-fragment3(34299)Conco1       -------RNIKLRENN----------P-NPDILQSLID-SEDPE---TGEK---------   39
CYP5855C-fragment1(34389)Conco1       -------DNIKLRKNN----------P-SPDILQSLID-SKGSE---TGEK---------   39
CYP5855C7(34382)Conco1                -------ENIKLRENN----------P-HPDILQSLID-SKDPE---TGEK---------   280
CYP5855C8(2259)Conco1                 -------ENIKLRENN----------P-HPDILQSLID-SKDPE---TGEK---------   280
CYP5855D7(2257)Conco1                 -------ENIKIRREST--------NV-HYDILQSLID-AEDPE---TGGK---------   216
CYP5855D1(29975)Conco1                -------DNIQKRRSTS--------KT-HNDILQSLID-ARDPD---SGEG---------   159
CYP5855D-fragment5(29026)Conco1       ------------------------------DILQSLID-SKDPE---NGEG---------   17
CYP5855D-fragment7(2254)Conco1        ------------------------------------------------------------   217
CYP5855C-fragment2(30530)Conco1       ------------------------------------------------------------   0
CYP5855E1(38687)Conco1                -------KNINARRQDP--------SLRNEDILQSLID-AKDPE---TGEQ---------   194
CYP5855D6(6708)Conco1                 -------ENIGKRRKLT--------EV-NDDILQCMID-AEDPE---TGVK---------   272
CYP5855D-fragment1(169043)Conco1      ------------------------------------------------------------   0
CYP5855D-fragment6(24158)Conco1       ------------------------------------------------------------   0
CYP5855D4(54364)Conco1                -------ENINKRRNSN--------EI-HNDILQSMID-SKDPE---TGEK---------   280
CYP5855D5(76752)Conco1                -------DNINKRRNSN--------EV-HNDILQSMID-AKDPE---TGKG---------   200
CYP5855D-fragment2(2269)Conco1        ------------------------------------------------------------   0
CYP5855D3(54372)Conco1                -------ENIHKRRESN--------GI-HNDILQIMID-SEDPD---TGAK---------   278
CYP5855D-fragment3(28450)Conco1       ------------------------------------------------------------   0
CYP5855D8(45649)Conco1                -------NNIHERKQKN--------EE-HHDILQSMID-AKDPE---TGVG---------   192
CYP5855D2(2268)Conco1                 -------KNIHNRKQNN--------EK-HHDILQTMID-AKDSE---TGAG---------   269
CYP5855D-fragment4(27931)Conco1       ------------------------------------------------------------   0
CYP5858A-fragment7(4474)Conco1        ----------NHRKKERKKTAKDKTA-QYNDILSLILA-SNIKD----------------   120
CYP5856B-fragment1(2011)Conco1        ----------RLRRPANPIFSQTFQPEVYSPCVLETLD-VLDER----------------   159
CYP5858A-fragment1(68816)Conco1       ----------RHRKIVNSAFKKGWNLSVFSEIVDELFQ-K--------------------   151
CYP5858A-fragment2(31161)Conco1       ------------------------------------------------------------   0
CYP5858A-fragment5(21198)Conco1       ------------------------------------------------N-----------   1
CYP5858A-fragment3(23423)Conco1       ------------------------------------------------------------   0
CYP5858A-fragment4(23037)Conco1       ------------------------------------------------------------   0
CYP5858A-fragment6(21524)Conco1       ------------------------------------------------------------   0
CYP5858A-fragment8(27368)Conco1       ------------------------------------------------------------   0
CYP5858A1(68836)Conco1                LVNEYFEDLINKRREIM---KQEQD-YSASDVLSLLIE-SNLND---VN-----------   278
CYP5858A2(77667)Conco1                QVNIFFDKLISDRKEMM---KQDEN-YKSKDILSLMIE-SNMKD---LN-----------   279
CYP5858A3(36631)Conco1                QVNIFFDKLINDRKEMM---KQDEN-YKSKDILSLMIE-SNMKD---LN-----------   205
CYP5861A-fragment2(21280)Conco1       ------------------------------------------------------------   0
CYP5861A1(30625)Conco1                --------------------------SEPKDILSLMLL-S-MDK---DE-----------   244
CYP5861A-fragment1(36094)Conco1       -----------------------------------------MEK---GD-----------   5
CYP5856A4(2008)Conco1                 EFNKFIGEMVSKRRKEI---EKYKN-SDYKDVLSLMLE-NDPDS---PYE----------   277
CYP5856B2(45436)Conco1                EFKEFTMKMIQQRRKDL---KDHKF-EDSKDLLTTLLK-ETELN---QED----------   277
CYP5856B-fragment7(34125)Conco1       ------------------------------------MR-KSDSN---DQD----------   10
CYP5856B3(76636)Conco1                EFKEVIQALISQRRKDL---KDKKF-EDSKDLLSTLLR-ETEKN---HED----------   242
CYP5856B-fragment3(29023)Conco1       ------------------------------DLLSTLLR-ETANN---HED----------   16
CYP5856B-fragment4(76635)Conco1       EFNGFIQSLIDQRRKDM---KDHKF-EDSKDLLSTLLR-ETENN---HDD----------   51
```

CYP5856A1(34083)Conco1	EFKKFILGIVEERRSDI---KAGKA--NGKDLLATMLA-ADPES---PFE----------	277
CYP5856A2(34094)Conco1	EFKNFILDIVNTRRKEI---KSGKN--DGKDLLSTMLT-EDPDS---PFE----------	242
CYP5856A3(29445)Conco1	KFKKFILEIVEKRRAEM---KAGKV--GGKDLLATMLT-VDPDS---PFA----------	175
CYP5856B-fragment9(30415)Conco1	---S---PYE----------	4
CYP5856B-fragment10(35455)Conco1	-----------------------------------MLR-KDPES---PYE----------	11
CYP5856B-fragment8(44763)Conco1	-----------------------------------MLR-KDPES---PYE----------	11
CYP5856B-fragment11(169617)Conco1	-----------------------------------MLR-KDPES---PYE----------	11
CYP5856B-fragment12(27598)Conco1	---S---PYE----------	4
CYP5856B1(167158)Conco1	EFNSFILGLINQRRKDL---KNGKF-LDSKDLLSTMLR-DDPDS---PYE----------	242
CYP5856B-fragment2(34058)Conco1	EFNNFILSLISQRRKDL---ESGKF-LNSKDLLSTMLR-SDPDS---PYE----------	50
CYP5856A-fragment1(26325)Conco1	--	0
CYP5856B-fragment5(30225)Conco1	--	0
CYP5854A-fragment4(78559)Conco1	--	133
CYP5854A-fragment2(6332)Conco1	--	236
CYP5854A-fragment3(6331)Conco1	--	219
CYP5854E-fragment3(14082)Conco1	--	0
CYP5854E-fragment5(9574)Conco1	EYQEFIQEMIALRKDEL---KKGTL-VDNRDLISALVK-SNENS---SEV----------	226
CYP5854E-fragment4(9575)Conco1	--	0
CYP5854E5(14156)Conco1	EYHEFIQEMIALRMDEL---KKGTL-VENRDLVSALVK-SNDNS---SET----------	259
CYP5854E-fragment2(42529)Conco1	--	0
CYP5854D1(9752)Conco1	EYDQFVESIIDSRYEAI---KNGEVNTENKDLITKLIL-SNMEE---DES----------	279
CYP5854E-fragment13(126715)Conco1	--	144
CYP5854E8(11612)Conco1	AYHEFIEGIIKLKEKEI---VEGKE-NNSKNLVSSLIQ-SNIKL---GDD----------	266
CYP5854E-fragment17(6815)Conco1	EYHGLIEEIMEVKKKQY---HESS--EKSKDLITAFIE-SNEKE---DEF----------	219
CYP5854E12(104896)Conco1	EYYELIEEMIAEKKRQI---QEGS--TRSKDLITAFIE-SNEKE---GEF----------	277
CYP5854E15(12851)Conco1	EYHKFIEEMMEMKKKQY---HEGT--EKSKDLITAFIE-SNEKE---GEL----------	270
CYP5854E16(80744)Conco1	EYHEFIEEMMELKKKQY---YEGT--EKSKDLITAFIE-SNEKE---GEF----------	277
CYP5854E17(80468)Conco1	KYHEFVEEMLNLRSREL---KEGKS-NNSQNLITSFLK-SNEKE---DES----------	227
CYP5854E13(77675)Conco1	EYHEFVEEMIKIKKKQY---HESS--EKPKDLITALIE-SNEKE---VEF----------	154
CYP5854E14(12850)Conco1	KYHEFVEEMMEMKKKQY---RKGT--EKPKDLITAFIE-SNEKE---DQY----------	269
CYP5854E7(11610)Conco1	EYHGFVEEMMAQKKKQL---HEGS--AKSKDLITAFIE-SNEKE---DEF----------	277
CYP5854E-fragment16(8906)Conco1	----------------------------SKDLITAFIE-SNEKE---DEF----------	18
CYP5854A3(78558)Conco1	QYNEFVDNLVDCRKKEI---LEGKA--NKDNLLTQFAQ-ASVPL---DGK----------	100
CYP5854A1(154349)Conco1	EYNKFVDDLIESRKKQI---LNGEA--HKDNLLTQFVQ-ASMPS---SEK----------	279
CYP5854A2(17189)Conco1	EYNLFVENLIERRRQEI---LDGKA--NNDNLLTQFVQ-ASMPT---DKE----------	280
CYP5854A-fragment1(6337)Conco1	--	0
CYP5854B1(5669)Conco1	SYDEFVKQMVALKKEEL---IENPNLAGKDDLLSKMLL-SSMQA---DDE----------	279
CYP5854B2(5666)Conco1	DYDNFVKELIVKKRAEL---KESPEIEK-NDLLSKLIM-ASME-----DG----------	276
CYP5854B3(78236)Conco1	SYDKYVFDMVTTRREEL---KLNPDSKA-QDLLSKLVL-ASEE-----DG----------	236
CYP5854B(38119)Conco1	--	0
CYP5854C1(140971)Conco1	EYDRLIEDFISQRMKLI---EKKKA-DPKDNLLNAMIS-SYLKG---LMS----------	186
CYP5854E-fragment15(30483)Conco1	--	0
CYP5854E10(11616)Conco1	EYLDFVQSIINKKKEEL---QIDSE-KSGDNLVSAFLK-SNEKT---DDQ----------	167
CYP5854E-fragment14(126716)Conco1	------MDIINKKKEEL---ASGSE-KSNQNLISTFLM-SNEKT---DDQ----------	36
CYP5854E-fragment1(11608)Conco1	--	152

```
CYP5854E6(11609)Conco1                EYHGYIEDLIKSKTQEL---REGKL-SNKGDLISALIE-SNEKS---QEY----------   253
CYP5854E-fragment6(12533)Conco1       ------------------------------------------------------------   0
CYP5854E-fragment7(8022)Conco1        ------------------------------------------------------------   0
CYP5854E9(11613)Conco1                EYHEFIEDMIENKKIDL---KKGKL-SKKGDLISAFIE-SNENS---KDQ----------   234
CYP5854E-fragment12(13308)Conco1      EYHEFIEEMINVKKEEL---KNGTL-NSSKDLISALIH-SNENS---QEY----------   227
CYP5854E-fragment10(13854)Conco1      QYHEFIQELIKLKQNEL---KNGTL-KSNGDLISALVH-SNENS---EEY----------   83
CYP5854E-fragment11(13446)Conco1      QYQEFIQELIKLKQNEL---NNGAL-KSNGDLISALLH-SNENS---EEY----------   83
CYP5854E2(13461)Conco1                QYHEFIEELIKLKKEEL---KNVTL-KSNRDLISALIQ-SNENS---EES----------   278
CYP5854E-fragment8(12835)Conco1       ------------------------------------------------------------   0
CYP5854E4(14118)Conco1                QYHEFIQELIKLKQNEL---NNGTL-KSNGDLISALVQ-SNENS---EEG----------   277
CYP5854E11(11719)Conco1               QYHEFIEEILKLKNEEL---KNGTL-NSNGDLISAFLQ-SNENS---EEN----------   264
CYP5854E1(13460)Conco1                QYHEFIQELIKLKQNEL---NNGTL-NSNGDLISAFIQ-SNENS---EEY----------   193
CYP5854E3(12836)Conco1                ------------------------------------------------------------   0
CYP5854E-fragment9(11720)Conco1       ------------------------------------------------------------   0


CYP5854A-fragment5(6317)Conco1        ------------VSTRELR--LEN----MFVTGQKTTANIIIATIYYLSRYPEIQENCIL   125
CYP5856B-fragment6(2022)Conco1        ------------------------------------------------------------   0
CYP5862A1(43654)Conco1                ------------FNTEDITLFVVN----LIFASVVTTTNNSTLALYDMVTYPEYYNELYE   139
CYP5862A-fragment1(25548)Conco1       ------------YTIEQISSIMLS----LAFASVMTTTNNSTISFYDMVTYPEYYNELYE   68
CYP-fragment(71373)Conco1             ----------------LAAKAVSF----IMLAGFETASTGIICAVHRLAFDQDLQEKVRT   158
CYP5855A3(13529)Conco1                ------------LRDDEIIDEAIT----LMFAGIDTTSNTLIFTIYEILKDRNLYNRITD   330
CYP5855A-fragment1(11397)Conco1       ------------------------------------------------------------   255
CYP5855A1(44820)Conco1                ------------LRDDEIIDEAMT----LLVAGFDTTSNSLIFTIYEVLKNPTIYNKLVA   250
CYP5855A2(30387)Conco1                ------------LRDDEIIDEAIT----LLFAGIDTTSNTLIFTIYEILKNRDLYNRITG   251
CYP5855A4(43538)Conco1                ------------LRDDEIIDEALV----LLFAGIDTTANTLIFTIYEILKNKDLYTRIVD   249
CYP51F14(41890)Conco1                 ----------TMMTDRQVAHMMIA----LLMGGQHTSSTTSSWTLLLLAQNPEIQEELLK   145
CYP5859A-fragment2(11509)Conco1       -------------------------------------------------MNPEAYKSIQI   11
CYP5859A-fragment1(29099)Conco1       ------------LSRKETIREVLV----IIIGGMDTTAITCSWAFWLILSNPEVYKKAQE   44
CYP5859A-fragment3(168491)Conco1      ------------------------------------------------------------   0
CYP5859A1(11907)Conco1                ------------LTWTEVTREMIV----IIVGGMDTTAITLAWTFWLMLKHPEVYKEVQK   328
CYP5859A2(11511)Conco1                ------------LTWTEVAREMIV----IVVGGMDTTAVTLSWTFWLMLRYPEVYKEVQK   328
CYP5860A3(24597)Conco1                ------------LTDKELQDETMI----LLFSGVDTTASTISMCIYHLLKYPNVYFNVLE   300
CYP5860A1(168890)Conco1               ------------LTDKQLQDEAII----LLFAGVDSTASTMSLCIYNLLKYPKVYASLLE   332
CYP5860A2(12313)Conco1                ------------LTDRQLQDEAIV----ILFAGVDSTASTMSLCIYNLLKYPKVYANLLE   332
CYP5863A1(2687)Conco1                 YTLNYLKQVEENYDREQLAASLAN----LLMGGTETNSATMRWAFVFLLENSHILENVQN   332
CYP5857A2(2640)Conco1                 ------------FSDAEIIEEACI----LIFAGMETTAISLIWTFYLLLSNPKTYENLVE   318
CYP5857A4(2643)Conco1                 ------------FTDQEIIEEASI----LIFAGMETTAVALIWTLYLL-----------E   146
CYP5857A-fragment1(12733)Conco1       ------------------------------------------------------------   223
CYP5857A3(2641)Conco1                 ------------FTNEEIIEEASI----LIFAGMETTAISIIWAFYNLLKNPETYMKLSR   302
CYP5857A7(2648)Conco1                 ------------FNDEEIIEEVSI----LIFAGMETTAVALIWTFYLLGKNPYSYEKLVD   317
CYP5857A6(2646)Conco1                 ------------FTDEEIIEESSG----LIFAGMETTAMGLIWTFYLLGKNPQAYEKLVD   169
CYP5857A-fragment2(24936)Conco1       -----------------------------IFAGLETTAMGIIWTFYLLGKNPQAYEKLVD   31
CYP5857A8(2649)Conco1                 ------------FNDVEIIEEASA----LIFAGMETTAVAIIWTFYLLGKNPQAYKKLVE   318
CYP5857A1(14904)Conco1                ------------FTDEEIIEEASI----LIFAGMETTAVALIWTFYLLGKNPQVYEKLVD   318
```

```
CYP5857A5(2645)Conco1               ------------FTDQEIIEEASI----LIFAGMETTAVALIWTFYLLGKNPQAYEKLVD   169
CYP5855B1(4344)Conco1               ------------LTLTEIIEEFFI----ILYAGLDTTSNTMTWTLYEILKNPKLYNLIKQ   319
CYP5855B-fragment1(4348)Conco1      ------------------------------------------------------------   250
CYP5864A1(18764)Conco1              QYKQTSQQIGDELTEEDLISESLI----LLIGGVDSTASGLSWLFYYLDQYPKVYLKLAY   308
CYP5855-fragment1(2292)Conco1       ---------MKKFSDLEIVEECIV----LLFDGVYTTSNTLIWTMYETLKHPEIYKS---   44
CYP5855C1(34338)Conco1              CMILEDPETGEKLSHDEIARECLI----LLLAGMDTTAITLSWTLYMILKNPEIYKLVED   331
CYP5855C2(2240)Conco1               -----------KLSHDEIARECLI----LLFAGMDTTAITLTWTLYMLLKNPEIYKLVED   311
CYP5855C3(2245)Conco1               ------------LTNKEIRVEGMT----LLVAGMDSTANSLTWALYELLKNPEAYELVEK   324
CYP5855C4(21824)Conco1              ------------LSNEQIKTESMT----LLVAGMDSTANSLTWALYELLKNLEAYELVEK   305
CYP5855C5(2262)Conco1               ------------LTNDQIAVECMT----LLFAGMDTTANTLTWTIYELLRNPDVYELVEK   322
CYP5855C9(2252)Conco1               ------------LTNEQIAVEFMT----LLFGGEDTTANTLTWTLYELLKNPEIYNLVEK   324
CYP5855C6(27512)Conco1              ------------LTNDQIAVECMT----LLFAGMDTTANTLTWTLYEILRNPNVYELVEK   249
CYP5855C-fragment3(34299)Conco1     ------------LTDKQIAVECMT----LLFAGMDTTANTLVWTLYELLKNPDIYKLVEK   83
CYP5855C-fragment1(34389)Conco1     ------------LANDQIAVECMT----LLFAGMDTTSNTLTWTLYELLKNPDIYDLVEK   83
CYP5855C7(34382)Conco1              ------------LTNEQITVECMT----LLFAGMDTTANTLTWTLYELLKNPDIYELVEK   324
CYP5855C8(2259)Conco1               ------------LTNEQIAAECMT----LLFAGMDTTANTLTWTLYELLKNPDIYDLVEK   324
CYP5855D7(2257)Conco1               ------------LSDLEIFSECIV----LLFAGIETTSTTLTWTLYEILKHPEVYKLVAD   260
CYP5855D1(29975)Conco1              ------------LKDLEIVDECLV----LLFAGMDTTANTMTWTLYEILKHPDVYRLVRD   203
CYP5855D-fragment5(29026)Conco1     ------------LKDLEIVDECLV----LLFGGMDTTANTMTWTLYEILKHPEVYKQVTS   61
CYP5855D-fragment7(2254)Conco1      ------------------------------------------------------------   217
CYP5855C-fragment2(30530)Conco1     ----------------------FI----ILYAGLDTASNTMTWTLFEILNNPKVYDLIKS   34
CYP5855E1(38687)Conco1              ------------LTDVQIVDECVT----LLFAGMDTTANTLTWVLYEILRHPEVYALIEK   238
CYP5855D6(6708)Conco1               ------------LTDSEIVDECLV----LLFAGMDTTANALTWTIYELLRNPEVYELVAK   316
CYP5855D-fragment1(169043)Conco1    ------------------------------------------------------------   0
CYP5855D-fragment6(24158)Conco1     ------------------------------------------------------------   0
CYP5855D4(54364)Conco1              ------------LSDQQIVDECMV----LLFAGMDTTANTLTWTLYEIIKHPGIYELVSN   324
CYP5855D5(76752)Conco1              ------------LTDLEIVDECMV----LLFAGMDTTANTLTWTLYEMIKHPELYELIAN   244
CYP5855D-fragment2(2269)Conco1      ------------------------------------------------------------   0
CYP5855D3(54372)Conco1              ------------LTDLEIVNECYI----LLLAGMDTTANTLTWTLYELLKHPEILELVTT   322
CYP5855D-fragment3(28450)Conco1     ------------------------------------------------------------   0
CYP5855D8(45649)Conco1              ------------LTDLEIVDECMT----LLFAAEDTTANTISWTLYELLKHPEVYKLVAD   236
CYP5855D2(2268)Conco1               ------------LTDLEIVDECMA----LLFAAEDTTAITLSWTLYELLRHPEFYKLVAD   313
CYP5855D-fragment4(27931)Conco1     ---------------------------------------------YELLKHPEFYKLVAD   15
CYP5858A-fragment7(4474)Conco1      ---------CNGLTDDKIRQNTLI----FFLGGQDTASLSICAALHLLAEHLEIQEIIRK   167
CYP5856B-fragment1(2011)Conco1      ---------VNR----------------------------------LQGKPVEVKDLMEL   176
CYP5858A-fragment1(68816)Conco1     ---------MGE----------------------------------SGNINVDVEDLMQR   168
CYP5858A-fragment2(31161)Conco1     ------------LTDQEIKFDTLM----FFLAGQDTTSFSLCAALHLLAEHPEIQDRLRS   44
CYP5858A-fragment5(21198)Conco1     -----------GLTDEEVRSNTLA----MYLAGQNTTTFTICATLHILAEYPEIQEKMRQ   46
CYP5858A-fragment3(23423)Conco1     -----------------------------FLAGQDTTTFTICATLHLLAEHPDIQDRVRK   31
CYP5858A-fragment4(23037)Conco1     ----------------------------FFLAGQDTTTFTICATLHLLAEHPEIQERARK   32
CYP5858A-fragment6(21524)Conco1     -----------------------------FLAGQDTTTLTICAALHLLAEHPEVQEKMRK   31
CYP5858A-fragment8(27368)Conco1     -----------------------------FLAGQDTTTFTICAALHLLAEHPEIQEKMRQ   31
CYP5858A1(68836)Conco1              -----------GLTNEEVKNNTLI----FFLAGQDTTSFTLCATLHLLAEHPEIQEKLRL   323
CYP5858A2(77667)Conco1              -----------GLTEEDVRYNTLI----FFLAGQDTTSFTLSAVLYLLAEHPEIQEKVRQ   324
CYP5858A3(36631)Conco1              -----------GLTEEDVRYNTLI----FFLAGQDTTSFTLSAVLYLLAEHPEIQEKVRQ   250
```

```
CYP5861A-fragment2(21280)Conco1        -------------------------------AGHDTTAFALAVGLYFLCKNPEIQEKARQ   29
CYP5861A1(30625)Conco1                 -----------KLTDKEIRDNIMM----FVLAGHDTTALALTVAIYFLCKHPNFQDKARR   289
CYP5861A-fragment1(36094)Conco1        -----------KLTDKEIRDNIMM----FVMAGHDTTALSLVVAIHFLCKHPNYQDQARR   50
CYP5856A4(2008)Conco1                  -----------PFSDEEIIGNLNI----FIIAGHDSTAHTLSYTMYYLAKDQNIQQKLRN   322
CYP5856B2(45436)Conco1                 -----------PLTDEELVHNLNV----FFIAGHDTTASSLSYGMYHLAKNQEVQKKLRK   322
CYP5856B-fragment7(34125)Conco1        -----------RLTDEEIVHNLNS----FFVAGHDTTANSLACEMYHLAKNPNIQEKLRK   55
CYP5856B3(76636)Conco1                 -----------PLTDEELVYNMNV----FFAAGHDTTSSALACGMYYLAKNPIFQEKLRN   287
CYP5856B-fragment3(29023)Conco1        -----------PLTDEELVHNLNV----FFVAGHDTTANTLACGMYQLAKNKQVQDKLRK   61
CYP5856B-fragment4(76635)Conco1        -----------PLTDEELVHNLNV----FFVAGHDTTANTLSFGMYYLAKHRHVQDKLRK   96
CYP5856A1(34083)Conco1                 -----------PLTDEELVENLIV----FFIAGHDTTANTLSYAMYYLARNPEIQEKLRN   322
CYP5856A2(34094)Conco1                 -----------PLSDVELVENLIV----FFVAGHDTTANTLSYAMYHLARNPEIQEKLRK   287
CYP5856A3(29445)Conco1                 -----------PLTDQELVENLIV----FFIAGHDTTANTLSYAMYHLARNPDIQEKLRS   220
CYP5856B-fragment9(30415)Conco1        -----------PLTDEELVQNINL----LFFAGHDTTASTLSYALYSLARNRGIQDKLRD   49
CYP5856B-fragment10(35455)Conco1       -----------PLTDEELVQNINL----IFLAGHDTTASTLCYALYNLARNRDVQDKLRN   56
CYP5856B-fragment8(44763)Conco1        -----------PLTDEELVQNINL----FFFAGHDTTASTLSYALYSIARNRDIQDKLRN   56
CYP5856B-fragment11(169617)Conco1      -----------PLTDEELVHNINL----IFLAGHDTTASTLSCALYNLARNRDIQDKLRN   56
CYP5856B-fragment12(27598)Conco1       -----------PLTDEELVHNINL----IFLAGHDTTASTLSCALYNLARNRDIQDKLRN   49
CYP5856B1(167158)Conco1                -----------PLTDEELVHNFNI----FFLAGHDTTANTLSYALYHLARNRDVQDKLRN   287
CYP5856B-fragment2(34058)Conco1        -----------PLTDEELVHNLNI----FFLAGHDTTANTLSYALYHLARNRDVQDKLRN   95
CYP5856A-fragment1(26325)Conco1        ------------LTDKELINNLNI----FFIAGHDTTANTLSYAMYHLAKNQHVQDKLRA   44
CYP5856B-fragment5(30225)Conco1        ------------LTNEELVANLNV----FIMAGHDTTANTLTYAMYYLAKYQDVQEKLRN   44
CYP5854A-fragment4(78559)Conco1        ------------------------------------------------------------   133
CYP5854A-fragment2(6332)Conco1         ------------------------------------------------------------   236
CYP5854A-fragment3(6331)Conco1         ------------------------------------------------------------   219
CYP5854E-fragment3(14082)Conco1        ------------------------------------------------------------   0
CYP5854E-fragment5(9574)Conco1         -----------KLTMEEIRANLNV----FIVAGHDTTSNTLTSTLYYLARYPDIQEKLRS   271
CYP5854E-fragment4(9575)Conco1         ------------------------------------------------------------   0
CYP5854E5(14156)Conco1                 -----------KLTMEEIRENLSV----FISTGHDTVTNTLTSTLYYLARYPDVQEKLRA   304
CYP5854E-fragment2(42529)Conco1        ------------------------------------------------------------   0
CYP5854D1(9752)Conco1                  ----------KRLTGDEIRDNIKI----FIMAGQDTTANTITTALYYLARYHDIQTKLRS   325
CYP5854E-fragment13(126715)Conco1      ------------------------------------------------------------   144
CYP5854E8(11612)Conco1                 -----------KLSNDEIRDDLSI----FIIAGHDTTSNTLISTLYYLARYPQVQDKLRT   311
CYP5854E-fragment17(6815)Conco1        -----------KLTTDEIRVIDVTLSDKYILISN*-------------------------   242
CYP5854E12(104896)Conco1               -----------KLTTDEIKHNINA----FISAGHDTTSNTLTTALYYLARYPETQEKLRA   322
CYP5854E15(12851)Conco1                -----------KLTNEEIRDNITI----FILAGHDTTSNTLSSTLYYLARYPEIQDKLRK   315
CYP5854E16(80744)Conco1                -----------KLTNDEIRDNIAM----FILAGHDTTSNTLTSTLYYLARYPEIQDKLRK   322
CYP5854E17(80468)Conco1                -----------KLTMDEIRDNITI----FILAGHDTTSNTLASTLYYLARYPEIQDKLRS   272
CYP5854E13(77675)Conco1                -----------KLTNDEIRDNITI----FILAGHDTTSNTLSTTLYYLARYPEIQDKLRK   199
CYP5854E14(12850)Conco1                -----------KLTNDEIRDNITI----FILAGHDTTSNTLSSTLYYLARYPEIQDKLRK   314
CYP5854E7(11610)Conco1                 -----------KLTNDEIRDNITI----FILAGHDTTSNTLASTLYYLARYPEIQDKLRK   322
CYP5854E-fragment16(8906)Conco1        -----------KLTNDEIRDNISV----FILAGHDTTSNTLSSTLYYLARYPEIQEKLRK   63
CYP5854A3(78558)Conco1                 ---------DPLLTPRELRDNLKI----FFIAGHDTTANTITAIIYYLARYPEIQEKLRA   147
CYP5854A1(154349)Conco1                ---------DAVMTDRELRDNLKI----FFVAGHDTTANTITAIIYYLSRYPEVQDKLRS   326
CYP5854A2(17189)Conco1                 ---------EAVLSDRELRDNLKI----FFVAGHDTTANTISAIIYYLARYPDVQAKLRS   327
CYP5854A-fragment1(6337)Conco1         ------------------------------------------------------------   0
```

```
CYP5854B1(5669)Conco1               ---------NSKMTEKEIIDNLKS----FFIAGHDTTSNTLTATLYYLARYPEIQDKLRS   326
CYP5854B2(5666)Conco1               ---------KALMSEREIRDNLKS----IFIAGHDTTSNTLTTTLYYLARYPEIQDKLRA   323
CYP5854B3(78236)Conco1              ---------KTLISDREIADNLKA----FFIAGHDTTSSTLAATFYYLARYPEVQDKLRT   283
CYP5854B(38119)Conco1               ----------------------------FLIAGHDTTSNTLTATLYYLARYPDVQNKLRT   32
CYP5854C1(140971)Conco1             -----------GLELEEIRDNMKL----LILAGHDTTAYTLTSILYLLAKNPDIQSKLRS   231
CYP5854E-fragment15(30483)Conco1    ----------------------------------------------------EIQQKLYE   8
CYP5854E10(11616)Conco1             -----------KLTMEEIRDNLLV----FIIAGHDTTSNALTSTLYYLARYPEIQDKLRA   212
CYP5854E-fragment14(126716)Conco1   -----------KLTMNEIRDNIII----FILAGHDTTSNTLTSTLYYLARYPEIQDKLRS   81
CYP5854E-fragment1(11608)Conco1     ------------------------------------------------------------   152
CYP5854E6(11609)Conco1              -----------KLTMEEIRDNLNI----FIIAGHDTTSNTLTSTLYYLARYPELQDKLRA   298
CYP5854E-fragment6(12533)Conco1     ------------------------------------------------------------   0
CYP5854E-fragment7(8022)Conco1      ------------------------------------------------------------   0
CYP5854E9(11613)Conco1              -----------KLTMEEIRDNLNI----FIFAGHDTTSNTLTSTLYYLARYPEIQDKLRA   279
CYP5854E-fragment12(13308)Conco1    -----------KLTMEEIRDNLNL----FIIAGHDTTSNTLMSTLYYLARYPEIQNELRS   272
CYP5854E-fragment10(13854)Conco1    -----------KLTMDQIRDNLNV----FTIAGHDTTSNTLISTLYYLARYPEIQDKLRR   128
CYP5854E-fragment11(13446)Conco1    -----------KLTMDQIRDNLNV----FIIAGHDTTSNTLVSTIYYLARYPEIQDKLRD   128
CYP5854E2(13461)Conco1              -----------NLRIEEIRDNLNI----FIIAGHDTTSNTMTSTIYYLARYPEIQDKLRR   323
CYP5854E-fragment8(12835)Conco1     ------------------------------------------------------------   0
CYP5854E4(14118)Conco1              -----------KLTMEEIRDNLNF----FIMAGHDTTSNTLTGTLYYLARYPEIQDKLRG   322
CYP5854E11(11719)Conco1             -----------KLSMEEIRDNLNV----FIIAGHDTTSNTLASTIYYLARYPEIQDKLRG   309
CYP5854E1(13460)Conco1              -----------KLTIEQIRDNLGI----FIIAGHDTTSNTLTSTLYYLARYPETQDKLRG   238
CYP5854E3(12836)Conco1              ------------------------------------------------------------   0
CYP5854E-fragment9(11720)Conco1     ------------------------------------------------------------   0


CYP5854A-fragment5(6317)Conco1      K*----------------------------------------------------------   126
CYP5856B-fragment6(2022)Conco1      ----------------------------------------MRISLTVPQM-GRQLAEDYL   19
CYP5862A1(43654)Conco1              EQLQIKKEFG-----DDISP---DIV-SKMLKLNSFIRETMRFRLEAVSS--FRITRSDW   188
CYP5862A-fragment1(25548)Conco1     EQLEVEKEFG-----DVINA---EAV-SKMVKLNSFIRESMRFRLAGVTN--FRFNKNDW   117
CYP-fragment(71373)Conco1           DQT----------------------------LIPKLVSLSLFANPPLMQN-FNMLTTSNI   189
CYP5855A3(13529)Conco1              QILKEFPDPN-----AKITV---EECRTKLTLLEAKLFQKAGLW*---------------   366
CYP5855A-fragment1(11397)Conco1     ------------------------------------------------------------   255
CYP5855A1(44820)Conco1              EILKEFPNPD-----AKITV---EDCRKRLPLLEATILESFRFRPVAFGP-ITRIVPAGG   301
CYP5855A2(30387)Conco1              QILKEFPDPN-----AKITV---EDCRKRLSFLEATLFESLRYYPVAFGP-IPRIVPSGG   302
CYP5855A4(43538)Conco1              EILREFPDPN-----AIIAA---EDCRKRLPLLEATLFESLRFHPVAFGP-IPRIVPCEG   300
CYP51F14(41890)Conco1               EQKEVFGEDL-----KDLTL---DGLK-KCTLLESCIKETLRMRPPIINV-MRYVKQNCE   195
CYP5859A-fragment2(11509)Conco1     ETDIIFPNRD-----MPLCP---ETCRTMMPITK--------------------------   37
CYP5859A-fragment1(29099)Conco1     EVDKVFPDKN-----IPIDV---TTCKTKMPIIEAILLETMRLYPVSAMP-LIRNPPATG   95
CYP5859A-fragment3(168491)Conco1    ------------------------------------------------------------   0
CYP5859A1(11907)Conco1              EISEVFPNKD-----MAPCA---DTCKSNLPITEAVVLESMRMYPVSAEY-LPRTAPVGG   379
CYP5859A2(11511)Conco1              EVDEIFPNKD-----IAPSA---DTCKSSLPITEAVALESMRMYPVSGEY-LPRTAPVSG   379
CYP5860A3(24597)Conco1              ELDRVKPVDEVTG--KIRSF---SWLKENIPYLGAVIYETIRLYPAFAFG-VPRLVPEGG   354
CYP5860A1(168890)Conco1             ELDRVKPFDQETG--EIKPY---LWCRENLPYLEAVIYETLRLTPAFPLG-VPRVVPEQG   386
CYP5860A2(12313)Conco1              ELDRVKPFDQETG--EIKPY---LWCRENLPYLEAVVYETLRLTPAFPFG-VPRVVPEQG   386
CYP5863A1(2687)Conco1               ELKA----LN-----KDLIT---WEDRSKLHYLQAFLMEVQRVGCVTTLGGSTLRVAGEN   380
```

```
CYP5857A2(2640)Conco1               ELINAFPDKN-----TQISY---DMCK-DLPYLTAVIHESLRLKNPSGVA-LTRVVPEGG   368
CYP5857A4(2643)Conco1               ELINTFPDKS-----AKISY---DMSK-NLPYSTAVIHGSLRIKSPIGLL-LPRAVPEEG   196
CYP5857A-fragment1(12733)Conco1     ------------------------------------------------------------   223
CYP5857A3(2641)Conco1               ELISTFPDIN-----IYINY---DMCK-DLPYLTSVIHESLRLKTPAGCP-LPRVVPEGG   352
CYP5857A7(2648)Conco1               ELVTAFPDKN-----TKISY---DMCK-DLPYLTAVINESLRVKSPSGIL-LPRIVPEGG   367
CYP5857A6(2646)Conco1               ELVTAFPDKN-----TKISY---DMCK-DLPYLTAVIYESLRAKSPSSSL-LPRVVPEGG   219
CYP5857A-fragment2(24936)Conco1     ELITAFPDKN-----TKITY---DMCK-NLPYLTAVIYESLRVKSPSGVV-LPRVVPEGG   81
CYP5857A8(2649)Conco1               ELIVTFPDKN-----TKISY---DMCK-DLPYLTAVIHESLRIKSPAGVI-LPRVVPEGG   368
CYP5857A1(14904)Conco1              ELVTAFPDKN-----TKISY---DMCK-DLPYLTAVIHESLRVKSPAGVV-LPRVVPEGG   368
CYP5857A5(2645)Conco1               ELVTAFPDKN-----TKISY---DVCK-DLPYLTAVIHESLRVKSPAGVV-LPRVVPEGG   219
CYP5855B1(4344)Conco1               EILNNFPDLT-----KPINL---SDCEAKLVHLEAAIWESLRMHSVVE-T-FARRVPEGG   369
CYP5855B-fragment1(4348)Conco1      ------------------------------------------------------------   250
CYP5864A1(18764)Conco1              EIRSKKIDLK------GLNV---NEMKKEYPYLNKVIMESLRLSPPFATS-FPRLVPESG   358
CYP5855-fragment1(2292)Conco1       --------------------------KNRVKILRGAILVSMRMNPIASGA-LLRVMPKGG   77
CYP5855C1(34338)Conco1              EVLEKFPNFN-----EPVSS---EKAKNSLKYLEAALLESMRMHPVASGG-IPRVVPEGG   382
CYP5855C2(2240)Conco1               EVLEKFPNFN-----EPVSS---EKAKNSLKYLEAALLESMRMHPVASGG-IPRVVPEGG   362
CYP5855C3(2245)Conco1               EMLEEFPSFN-----EPITV---EKVKSNCKYLEAAILESMRLYPAVAGG-MERVVPEGG   375
CYP5855C4(21824)Conco1              EILEEFPNFN-----EPITV---DKVKSNCKYLEAAILESMRLYPAVAGG-MERVVPEDG   356
CYP5855C5(2262)Conco1               EILEEFPNFN-----EPIPV---EKAKSSLKYLEAALLESMRIYPVAPGG-LPRVVPEGG   373
CYP5855C9(2252)Conco1               EILEEFPDFN-----ESITL---DRSKSKLKYLEASFLESMRMYPVAAGG-LPRVVPEGG   375
CYP5855C6(27512)Conco1              EILDEFPNFN-----EPISV---EKAKSNLKYFEATLLESMRIYPVAPGG-LPRVVPEGG   300
CYP5855C-fragment3(34299)Conco1     EILQEFPNFN-----EPIPV---EKAKSNLKYFEAALLESMRLYPVAPGG-LPRVVPEGG   134
CYP5855C-fragment1(34389)Conco1     EILEEFPNFN-----EPIPV---EKAKSNLKYLEAALLESMRLYPVVTGG-LPRVVPEGG   134
CYP5855C7(34382)Conco1              EILEEFPNFN-----EPIPV---EKAKSNLKYLEAALLESMRLYPVAPGG-LPRVVPEGG   375
CYP5855C8(2259)Conco1               EILEEFPNFN-----EPIPV---EKAKSNLKYLEAVLLESMRLYPVVPGG-LPRVVPEGG   375
CYP5855D7(2257)Conco1               EVLEKFSDLN-----NPVSI---SEAKTELKYLEAAILESMRINPVVTGA-LLRVVPEGG   311
CYP5855D1(29975)Conco1              EILEKFPNLN-----RPISY---DLARNGLEYFDACVLESMRKNPVGAGP-MPRVVPEGG   254
CYP5855D-fragment5(29026)Conco1     EILTHYPNLK-----EPITV---EKTKSELKYLEACILESMRKNPVASGA-IVRVVPESG   112
CYP5855D-fragment7(2254)Conco1      ------------------------------------------------------------   217
CYP5855C-fragment2(30530)Conco1     EILSNFPDLT-----KPLNI---SDCESKLKYLEAAIWESMRKHPVVE-M-FARRVPSEG   84
CYP5855E1(38687)Conco1              EIFTAFPNAE-----DVATI---ERCKSELPALEATILESMRLHPVAAGP-MFKKVPQGG   289
CYP5855D6(6708)Conco1               EVLEKFPNLN-----EPISV---DIAKNELKYLDSAITGAMRMHPPAAGI-LPREVPEGG   367
CYP5855D-fragment1(169043)Conco1    ------------------------KAKNELKYLSAAILEGFRMHPVAAGQ-MPREVPEGG   35
CYP5855D-fragment6(24158)Conco1     ------------------------------------------------------------   0
CYP5855D4(54364)Conco1              EIIEKFPNLN-----EPISL---NVAKNELKYLSAAILEAMRMHPVASGA-LPREVPEGG   375
CYP5855D5(76752)Conco1              EVIEKFPNLN-----EPISL---DVAKSELKYLSAAIQEAMRMHPVAAGQ-LPREVPEGG   295
CYP5855D-fragment2(2269)Conco1      ------------------------------------------------------------   0
CYP5855D3(54372)Conco1              EILNNFPNLS-----EPITI---NNAKKNLKYLEAALLETMRMHPGAGGS-IPRVVPEGG   373
CYP5855D-fragment3(28450)Conco1     ---------------------------RDLKYLEAAILESMRMHPAVAGS-LPRQVPEDG   32
CYP5855D8(45649)Conco1              EILEKFPNFN-----EPINS---QDAKKELKYLDAAILESTRKNPAGADI-IPRDVPEGG   287
CYP5855D2(2268)Conco1               EIIEKFPNFN-----EPINS---QDAKKELKYLEAAILESTRKHPAGADI-LPREVPEGG   364
CYP5855D-fragment4(27931)Conco1     EIIEKFPNVN-----EPINS---QDAKKELKYLEAAVLESTRMHPAGADI-LPREVPEEG   66
CYP5858A-fragment7(4474)Conco1      EVLTVLGKDEYKNEKLQIPT---NEQLRKLEYTYVAMQETMKLYSALTVI-NHRVAL*--   220
CYP5856B-fragment1(2011)Conco1      MTLDVLGRGIFSHDFEAIAVWTTNENS*--------------------------------   203
CYP5858A-fragment1(68816)Conco1     VTVEALGREV*-------------------------------------------------   178
```

CYP5858A-fragment2(31161)Conco1	EVLMVLGKEEYIDDKIQTPS---QSQLDQLKYMHAVIKETMRLYPAVSIL-YNWIAKEDH	100
CYP5858A-fragment5(21198)Conco1	EVLSVLGKEEYINGTFQTPT---NDQLNQLEYTYAIMQESMRLYPALSLL-TYREAQKDV	102
CYP5858A-fragment3(23423)Conco1	EVLNVLGKDEYIAGKFQTPT---HEQLSQLEYTYAVMQESMRLYPAVSVL-THRVTQNDI	87
CYP5858A-fragment4(23037)Conco1	EVLNVLGNEEYIDGKFQTPT---IEQLNQLEYVNAIMQESMRLYPAVSVI-THRLAQKDI	88
CYP5858A-fragment6(21524)Conco1	EVLTVFGKDEYKNGKFQIPT---NEQLNKLEYTYAVMQETMRLYPAVSVI-NHRVALKDI	87
CYP5858A-fragment8(27368)Conco1	EVLAVFGKDEYETGKFQVPT---HDQLNKLEYTHAVMKETMRLYPSVSVI-LHREAQKDI	87
CYP5858A1(68836)Conco1	EVISVLGKDEYINERFQVPT---NEQLNRLEFTNAVIQESMRLYPAVTIL-TNWICKKDY	379
CYP5858A2(77667)Conco1	EVIKVLGENEYVDGKIQVPT---NEQLNQLEYLYAVIQETMRLYPAVTIL-TNWVSKNNY	380
CYP5858A3(36631)Conco1	EVIKVLGENEYIDGKLQVPT---NEQLNQLEYLYAVIQETMRLYPAVTIL-TNWVSKNNY	306
CYP5861A-fragment2(21280)Conco1	EALAVFGKPG--ADKRVPIT---HEQLKQIPFIESIIKEAMRYYPSVSRL-PSRINQVEL	83
CYP5861A1(30625)Conco1	EALAIFGKPG--NDKK-------------LPFIDSIIKESLRMYPPVAFL-PARLTKSEF	333
CYP5861A-fragment1(36094)Conco1	EALTVFGKPG--NDKKVSIS---HEQLKQLPFIDSIIKESLRMYPPVAFL-PPRVNKNEL	104
CYP5856A4(2008)Conco1	EVYKTIEIPAN-QAEIVAPN---TESLKDMVYMDCVLKEAMRMSPAVVNL-HRHVAEEFQ	377
CYP5856B2(45436)Conco1	EIYEVLEISPE------------TKKLKSMEYLGLVIKETMRISPPVGLL-NRALADDYT	369
CYP5856B-fragment7(34125)Conco1	EVFTTLKIESN-ASKLAIPT---IDQIKEMEYLELFIKEAMRINPPVAEI-SRVLNDNYI	110
CYP5856B3(76636)Conco1	EVLQALGTSSA-SDKITVPE---ESQIKEMNYLGLFIKEVMRLNPPISQV-FRGLTEDYS	342
CYP5856B-fragment3(29023)Conco1	EIYDTLQIQPG-TNKIVVPT---FDQIKNMEYLGLFIKEVMRINPSVAQI-VRKLTEDYH	116
CYP5856B-fragment4(76635)Conco1	EIYDTLQINSG-ANKVVIPT---AEQIKSMDYLGLFIKEVMRLNPPISQI-FRTISEDYP	151
CYP5856A1(34083)Conco1	EIYTTLKIPKD-HEKLVVAS---TEQFKEMEYLNCVIKEVMRISPAVVQM-FRIVEEDFN	377
CYP5856A2(34094)Conco1	EIYTTLNIPKD-HDKLVAPS---TEQLKDMEYLNCVIKEVMRISPAVVQI-PRIVEEEFT	342
CYP5856A3(29445)Conco1	EIYNTLNIPKD-HTEVVVPS---AEQFKEMEYLNCVIKEVMRISPAVVQI-PRMVEEDFT	275
CYP5856B-fragment9(30415)Conco1	DIFQIMGLNNN-HKKLVIPT---SEQVKNMEYLHLVIKETMRMFPPILHL-GRTLGEDYP	104
CYP5856B-fragment10(35455)Conco1	DIYQKMGLNGN-HKKLVIPT---SEQVKSMEYLHLVIKETMRMFPAIIHI-GRQLAEDYP	111
CYP5856B-fragment8(44763)Conco1	DIYQKMGLSDN-HKKLVVPT---SEQVKNMEYLHLVIKETMRMFPPILHL-GRTLGEDYL	111
CYP5856B-fragment11(169617)Conco1	DIYQKMGLSDS-HKKLVVPT---SEQVKNMEYLHLVIKETMRMFPPILHL-GRQLSEDYP	111
CYP5856B-fragment12(27598)Conco1	DIYQKMGLNDN-HKKLVVPT---SEQVKNMEYLHLVIKETMRMFPPILHL-GRQLSADYP	104
CYP5856B1(167158)Conco1	EIYQKMNLDDN-QEKLVVPT---SEQLKNMEYLNLVIKETMRVSPAVQQI-GRKLAEDYT	342
CYP5856B-fragment2(34058)Conco1	EIYQKMNLDDN-QEKLVVPT---SEQLKNMEYLNLVIKETMRISPAVLQI-GRQLAEDYS	150
CYP5856A-fragment1(26325)Conco1	EIYSTLQVDPN-TKDLVIPT---SEQLKSMNYLACFIKEVMRITPAVLQV-NRELQSDYT	99
CYP5856B-fragment5(30225)Conco1	EIYATLEVAPN-NFDLAIPT---AEHLKSMKYLSCFIKEVMRITPAVLQI-DRQLSSDYT	99
CYP5854A-fragment4(78559)Conco1	--	133
CYP5854A-fragment2(6332)Conco1	--	236
CYP5854A-fragment3(6331)Conco1	--	219
CYP5854E-fragment3(14082)Conco1	--LSALGDPN--PLKVQIPT---IEQLKNILLLDLVNKELMRIMTTVSAF-LKKSTTIST	52
CYP5854E-fragment5(9574)Conco1	QVLSALDNPS--PQKLKFQL---LSN*----------------------------------	292
CYP5854E-fragment4(9575)Conco1	---MTTASAV-QRKSASIST	16
CYP5854E5(14156)Conco1	QVLSALGDPS--PSKVQIPT---IEQLKNIPLLDMVIKESMRIMTTVPAI-PKKSTIIST	358
CYP5854E-fragment2(42529)Conco1	-------------------T---IEQLKNIPLLDMVIKESMRIMTAVSAF-PKKSTIIST	37
CYP5854D1(9752)Conco1	EILNVLGNPT----LLTTPT---LDQLKSMPYLSQVIKESMRIVTTSSAV-TRIAAQDYT	377
CYP5854E-fragment13(126715)Conco1	--	144
CYP5854E8(11612)Conco1	QILGVLENPS----SVVIPT---TDQLKNMPYLDLVNKESMRIMSTAAQI-QRICTKEHT	363
CYP5854E-fragment17(6815)Conco1	--	242
CYP5854E12(104896)Conco1	EILEALDNPT----ELVTPT---IDQLKRIPYLDMTIKESMRILTTAANV-QRDTVKTHT	374
CYP5854E15(12851)Conco1	EVLEALGHPT----ELVTPT---IDQLKHIPYMDLITKESMRIMTTVASL-QRSTAQTHT	367
CYP5854E16(80744)Conco1	EVLEALGHPT----ELTTPT---IDQLKNVPYMDLVNKESMRIMTTTASL-QRYAAQTHT	374

CYP5854E17(80468)Conco1	EVLEVLGNPS----ELTVPT---IDQLKNMPYMDLVNKESMRIMTTASSI-QRDTFQTHT	324
CYP5854E13(77675)Conco1	EVLEALGHPI----DLTTPT---VDQLKNIHYMDMVNKESMRIMATAGVL-QRDTAQNHT	251
CYP5854E14(12850)Conco1	EVLEALGHPA----ELTTPT---IDQLKNIPYMDLVNKESMRIMATAVDV-QRDAAQPYT	366
CYP5854E7(11610)Conco1	EVLEALDHPT----ELVTPT---VDQLKHVPYMDLVTKESMRIMTTAVNL-QRDTAQTYT	374
CYP5854E-fragment16(8906)Conco1	EVLEALGHPV----ELTTPT---VDQLKNLPYMDLINKESMRIMTTAANL-QRHTAQTHT	115
CYP5854A3(78558)Conco1	EVIEIMGKSD----KTLIPN---NEQLKKMEYLNMIIKETIRIMTTAAVL-DRRADKPFT	199
CYP5854A1(154349)Conco1	EIIEVMGEPE----CVRVPT---VDELKQMNYLNMVIKESTRCMSAAPAA-QRRTTKSFT	378
CYP5854A2(17189)Conco1	EIIEIMGESD----SVRVPT---VDQLKQMDYLNMVIKESTRCMTTAAVL-SRRAAKPFT	379
CYP5854A-fragment1(6337)Conco1	--	0
CYP5854B1(5669)Conco1	EILQVMNNPQ----VLTNPT---VDQLKQMEYLNLVIKESMRAMATVSVL-ERVCTSEFQ	378
CYP5854B2(5666)Conco1	EILGVMNNPQ----VLTNPT---LEQLKQMDYLNMVIKESMRIMATASVL-ERVATKPFQ	375
CYP5854B3(78236)Conco1	EILEVMNNPQ----VLTNPT---LEQLKQMDYLNMVIKESMRRMATVSII-ERVSNAPFN	335
CYP5854B(38119)Conco1	EILEVMNSPQ----VLTNPT---FEELKQMKYLNMVIKESMRIMTTAVAV-ERISTNPFQ	84
CYP5854C1(140971)Conco1	EILNTLNNPE----KLTTPT---SEQLPQMEYLTLVIKESMRLLTPASEA-EREASQEYT	283
CYP5854E-fragment15(30483)Conco1	EVMTALENSK----SLTIPT---VAELKSMPYLDMINKESMRIMTTVTAL-QREAMEDCV	60
CYP5854E10(11616)Conco1	QVLEALGNPS----SVQIPN---VKQLKNIPLLDMVNKESMRIMTTAAAV-TRTAVQDCA	264
CYP5854E-fragment14(126716)Conco1	QILKALENPT----PVQIPT---IEQLKNIPLLDMVNKESMRAMTTVVSV-QRNTTEDYT	133
CYP5854E-fragment1(11608)Conco1	--	152
CYP5854E6(11609)Conco1	QIINATGDKT----QVKIPT---IDQLRKIPLLDKVNKESMRIMVTVPVV-QRIANSIYT	350
CYP5854E-fragment6(12533)Conco1	-----------------------------MPLMDMVNKESMRIMTTTASM-QRVCSSIHT	30
CYP5854E-fragment7(8022)Conco1	--	0
CYP5854E9(11613)Conco1	QVLAAMGSPK----QVTIPT---VEQLKKIPLMDMVSKESMRMMTTVNTT-ERVSKSHHT	331
CYP5854E-fragment12(13308)Conco1	QVLTAMGSPK----HVQ---	285
CYP5854E-fragment10(13854)Conco1	QILEAMEYPK----NVQIPT---IDQLKKMPYMDMVNKESTRIMTTVAEL-QREPASAFT	180
CYP5854E-fragment11(13446)Conco1	QVLEAMDYPK----N---	139
CYP5854E2(13461)Conco1	QILEAMDYPK----NVQIPT---IDQLKKMPYMDMVNKESMRIMTAATEL-QRVSSSAYT	375
CYP5854E-fragment8(12835)Conco1	-----MDYPK----NVQIPT---NDQLKKMPFMDIVNKESMRIMTTSSEI-QRLSYFDHT	47
CYP5854E4(14118)Conco1	QVLDAMNYPK----NVQIPT---IDQLKNIPFIDMVIKESMRIMTTVAAI-QRISSCTYT	374
CYP5854E11(11719)Conco1	QVLDAMNYPK----NVQIPT---VDQLKNIPFMDLVNKESMRIMTTVSSV-QRESSSTYT	361
CYP5854E1(13460)Conco1	QVLEAMDYPK----NVQIPT---NDQLKSVPFMDMVIKESMRIMTTASAV-QRTSSTTYT	290
CYP5854E3(12836)Conco1	-----MDYPK----NVQIPT---NDQLKKIPFMDMVNKESMRIMTTASAI-QKRPSSTYT	47
CYP5854E-fragment9(11720)Conco1	-----MNYPK----NVQIPT---IDQLKHVPFMDMVNKESMRIMTTASAV-QRRASTTYT	47

CYP5854A-fragment5(6317)Conco1	--	126
CYP5856B-fragment6(2022)Conco1	IEEDNIVLPKGTN----VGLSIYNILNDPAVY-PNPERFLNIK-----------------	57
CYP5862A1(43654)Conco1	ILSNGVIIPKGSF----IAVDSTSLHFDNELQDGSPYEFKPYRHFENGKLA---------	235
CYP5862A-fragment1(25548)Conco1	TLTNGITIPKGSL----MGVDAFSLHFDNELQDGSPYEFKPFRHAANGKLA---------	164
CYP-fragment(71373)Conco1	EIGDGIVLPKNTR----VNIFINAVHNY--------------------------------	213
CYP5855A3(13529)Conco1	--	366
CYP5855A-fragment1(11397)Conco1	--	255
CYP5855A1(44820)Conco1	VTIDGHFIPEGTL----IALNSYAINYSEEHF-PHASEFNIDKWLSPER-----------	345
CYP5855A2(30387)Conco1	VTIDGRFIPEDTV----IAYNTFAVNRNEEYF-PTPNEFNIDKWLSPEK-----------	346
CYP5855A4(43538)Conco1	ATIDGHFIPEGTE----IMFNTFAVHRSEENF-PSPNEFNIDKWLGPEK-----------	344
CYP51F14(41890)Conco1	IPNTSYVIPQGYY----VMSAPIITQLDEKCF-PNAESYLPKRWLDPSIAAGTNKTPTEN	250

```
CYP5859A-fragment2(11509)Conco1       ------------------------------------------------------------   37
CYP5859A-fragment1(29099)Conco1       TTLSGYEIPPNTN----VFMTNYAYHRNPELW-ENPDKFDYTRWLIDDN-----------   139
CYP5859A-fragment3(168491)Conco1      ------------M----VFSASYAYHRNPEIW-DNPDDFNYKRWLIEDN-----------   32
CYP5859A1(11907)Conco1                THLGGYEIPGDTL----VFCSPYAYQRNPELW-DKPDEFNYKRWLGPNS-----------   423
CYP5859A2(11511)Conco1                THLGGYEIPGDTL----VFCSPYAYQRNPELW-ENPDEFDYKRWLGPNS-----------   423
CYP5860A3(24597)Conco1                ATAAGYYLPSDTE----VSVSIFSYQRSAQYF-ENPDEFIPSRWLRNSK-----------   398
CYP5860A1(168890)Conco1               TTVGDYYLPESTE----VSVSIYSYQRSSQYF-SNPNEFQPERWLDKTD-----------   430
CYP5860A2(12313)Conco1                ATVGDYYLPENTE----VSVSIYSYQRSSQYF-SNPDEFHLERWLDKTD-----------   430
CYP5863A1(2687)Conco1                 CKIGDYQINKGAF----IAANVYSIHYNEDYF-SDPYKFNPERFLKDGE-----------   424
CYP5857A2(2640)Conco1                 ATILGHFIPEGTI----IGTGGYGIHQCEKNW-PNADSYIPERWLGSDA-----------   412
CYP5857A4(2643)Conco1                 ITLCGHYLPEGVSLFNYYSSSASGIHLSERNF-QDPESFTPERWSCSDA-----------   244
CYP5857A-fragment1(12733)Conco1       ------------------------------------------------------------   223
CYP5857A3(2641)Conco1                 VNILGHFLPAGTI----VGASGIGIHLSEKNV-QDPESFNPERWLGPEA-----------   396
CYP5857A7(2648)Conco1                 ATICGHFLPEGTV----VGSSANGIHLSEKNF-KNADTFVPERWLGLEA-----------   411
CYP5857A6(2646)Conco1                 ATICAHFLPEGTI----VGASSSGIHLSEQNF-KNADSFIPERWFGHEA-----------   263
CYP5857A-fragment2(24936)Conco1       ATLCGHFLPEGTV----VGSSSAGIHLSEQNF-QKADSFLPERWLGSNS-----------   125
CYP5857A8(2649)Conco1                 ATICGHSIPEGTL----IASSANGIHLSEKNF-KDSESFIPERWVDPDA-----------   412
CYP5857A1(14904)Conco1                ATICGHFLPEGTV----VGSSGVGIHLSESNV-QDPESFTPERWLGPEA-----------   412
CYP5857A5(2645)Conco1                 ATICGHFLPEGTV----LGSSCVGIHFSEINC-HKAESFIPERWLSPEA-----------   263
CYP5855B1(4344)Conco1                 ------------------------------------------------------------   369
CYP5855B-fragment1(4348)Conco1        ------------------------------------------------------------   250
CYP5864A1(18764)Conco1                LMIDGVYLPPKTQ----IAINPYPTMRSPQNF-SNPDEFKPDRWSEASG-----------   402
CYP5855-fragment1(2292)Conco1         IAIRRYCLTKNIL----KYVLSIA*-----------------------------------   97
CYP5855C1(34338)Conco1                ITANGHFLPHKTV----IFFPIYSQHHDPTLW-EDPSKFDISRWLGPNK-----------   426
CYP5855C2(2240)Conco1                 ITANGHFLPHKTV----IFFPIYSQHHDPTLW-EDPSKFDISRWLGPNK-----------   406
CYP5855C3(2245)Conco1                 ISVDGHFLPANTI----ISHPIYNQHHDPKNW-KNPKSYDIQRWIREGK-----------   419
CYP5855C4(21824)Conco1                ITVDGYFLPDNTI----ISHPIYNQHHDPKNW-KNPKSYDIQRWIGEDK-----------   400
CYP5855C5(2262)Conco1                 KK*---------------------------------------------------------   375
CYP5855C9(2252)Conco1                 TK---------------IILPIYSLHNDPKNW-KNPKIFDIQRWLGEER-----------   408
CYP5855C6(27512)Conco1                VTIAGHYLPANTI----ILQPVYSMHNDPQNW-KNPKVFDIQRWLGQDR-----------   344
CYP5855C-fragment3(34299)Conco1       VTIAGYYLPEKTI----IYHPIYNQHNDPRNW-KNPKTYDIQRWIGEHR-----------   178
CYP5855C-fragment1(34389)Conco1       VTIAEHFLPANTV----IFYPIYSQHHDPKNW-KKPKTYDIQRWLGEDR-----------   178
CYP5855C7(34382)Conco1                VTIAGHFLPEKTI----IFQPIYNLHHDPSNW-KEPKVYDIQRWLGDER-----------   419
CYP5855C8(2259)Conco1                 KR*---------------------------------------------------------   377
CYP5855D7(2257)Conco1                 ITVEGCYLPENTT----MSMDSFAQHMDPALW-KSPETYDISRWLGSDR-----------   355
CYP5855D1(29975)Conco1                LTVNGYYLPPKTV----IAMDIYSQHNDPSIW-PNPLKFDISRWLGPDR-----------   298
CYP5855D-fragment5(29026)Conco1       ITISGHYLPPGTS----VGLDIYAQQNDPSIW-KSPEIFDISRWLGEER-----------   156
CYP5855D-fragment7(2254)Conco1        ------------------------------------------------------------   217
CYP5855C-fragment2(30530)Conco1       VTIGDHYLPEKTV----ITLNLYAYHNNPKIW-KNPREFKINRWLGEER-----------   128
CYP5855E1(38687)Conco1                VTLNGQFIPENTS----VIFHVITYHLDPNYF-ENPKQFSIDRWLGPNR-----------   333
CYP5855D6(6708)Conco1                 LTIAGHYLPPKTE----IAIDIYTQHNDPAFW-ENPRKFDIDRWLGPNA-----------   411
CYP5855D-fragment1(169043)Conco1      MTIQGHYLPQGTE----ITLDIYTQHNDPNFW-KNPREFNLDRWLGPDT-----------   79
CYP5855D-fragment6(24158)Conco1       -------------------------HNDPNFW-ENPREFNLDRWLGPKA-----------   23
CYP5855D4(54364)Conco1                ITINGHYLPPKTV----IAIDIYTQNNDPNFW-ENPRKFDLSRWLGPNA-----------   419
CYP5855D5(76752)Conco1                LTIQGHYLPQGVS----NML*---------------------------------------   311
```

```
CYP5855D-fragment2(2269)Conco1         ------------------------------------------------------------   0
CYP5855D3(54372)Conco1                 VTICGHYIPPKSW----IAVDIYTENNDPSFW-ENPREFNLDRWMGENR-----------   417
CYP5855D-fragment3(28450)Conco1        LTVNSHYIPPKTS----ITIDIYTEHNDPSFW-KNPREFNLHRWLGEDR-----------   76
CYP5855D8(45649)Conco1                 LTINGHYLPPKTV----FTLDIYCEHNNPNFW-ENPREYDINRWFGEDR-----------   331
CYP5855D2(2268)Conco1                  LTINGYHLPPKTV----FTLDIYLEHNDPSFW-ENPRDYDINRWFGEDR-----------   408
CYP5855D-fragment4(27931)Conco1        LTINEYYLPPKTV----FALDIYCEHNDPKFW-ENPREYDINRWLE-DR-----------   109
CYP5858A-fragment7(4474)Conco1         ------------------------------------------------------------   220
CYP5856B-fragment1(2011)Conco1         ------------------------------------------------------------   203
CYP5858A-fragment1(68816)Conco1        ------------------------------------------------------------   178
CYP5858A-fragment2(31161)Conco1        DYN-GMLVPKNSI----IDTYIYAIHRNPKYF-ENPSKFDPSRFLSDSG--EE-------   145
CYP5858A-fragment5(21198)Conco1        HYK-NHIIPKGTI----VASCVYAMHRNPEYF-KDPNTFDPSRYLNGKI--DT-------   147
CYP5858A-fragment3(23423)Conco1        QYK-EHIIPKGTL----IDTCVYAIHRNPDYF-KDPNAFDPSRYLNDGI--DT-------   132
CYP5858A-fragment4(23037)Conco1        QYK-EHVIPKGTL----VDTCVYAIHRNPDFF-KDPNTFDPSRYLNGGL--DT-------   133
CYP5858A-fragment6(21524)Conco1        HHR-GHTIPAGTL----VNTIVYAIHRNPKYF-KNPNEFLPSRFLNGNI--DT-------   132
CYP5858A-fragment8(27368)Conco1        HHR-GHIIPAGTI----IDTVVYAIHRNPEYF-KNPNEFIPSRFLNGEI--DT-------   132
CYP5858A1(68836)Conco1                 NHN-GLMIPKNAI----IDSYIYAINRSPKYF-KNPNTFDPTRYLAGGI--DI-------   424
CYP5858A2(77667)Conco1                 NYN-GMNLPAGTV----IDLYVYAINRNEKYF-KNPNTFDPSRFLEGGI--DT-------   425
CYP5858A3(36631)Conco1                 NYN-GMNLPAGTV----IDIYVYAINRNEKYF-KNPNTFDPSRYLEGGI--DT-------   351
CYP5861A-fragment2(21280)Conco1        EVN-GITIPKNQI----LTLEMIHFSRSAKYW-ENPDEFDPYRFYNTTT--KD-------   128
CYP5861A1(30625)Conco1                 IAN-DIQIPASSL----ILVETFHIQNSSKYW-QNPSEFNPYRFYESSNSTKI-------   380
CYP5861A-fragment1(36094)Conco1        IVN-GIHIPKHSI----IVVETFHIQNSTKYW-QNPSEFNPYRFFESSDSAKS-------   151
CYP5856A4(2008)Conco1                  FPGEDIVVPKDNL----VSIAIYGVQNDPKIH-QNPKIFSPERFINTR------------   420
CYP5856B2(45436)Conco1                 IPGDSVVIPKGTV----VGISIYAVHNDPKNF-KNPEKFEPERFLNAK------------   412
CYP5856B-fragment7(34125)Conco1        IPGDHVVLPKGLR----VSVSIYGIHHDPKIY-KNPEEFNPERFLNGK------------   153
CYP5856B3(76636)Conco1                 IPDDDVILPKGTI----INLPIYAIHHDPKIY-PNPEEFNPERFLNGK------------   385
CYP5856B-fragment3(29023)Conco1        IPSDDVVIPKGTT----VNVSIYGIHHDPKIY-PNPEKFDPERFLVGK------------   159
CYP5856B-fragment4(76635)Conco1        LPDDNVILPKGTT----VNLSIYSVHHDPKIY-PNPEEFDPERFFNGK------------   194
CYP5856A1(34083)Conco1                 IQNENIVVPKGTL----ITLSVYGAHHDPKTY-PNPHKFEPERFLNGK------------   420
CYP5856A2(34094)Conco1                 IPNDNVVVPKGSM----ISMSIYGAHHDPKTY-PNPQKFEPERFLNGK------------   385
CYP5856A3(29445)Conco1                 VPNDNVVIPKGSM----IMMSVYGSHYDPKTY-PNPKKFEPERFLNGK------------   318
CYP5856B-fragment9(30415)Conco1        IPEDNIVIPKGTN----IALSIYSILNDTKIY-PNPEKFNPERFLNAK------------   147
CYP5856B-fragment10(35455)Conco1       IPEDNIVIPKGTS----IALSIYSILNDPKIY-PNPEKFNPERFLNAK------------   154
CYP5856B-fragment8(44763)Conco1        IPEDNIVIPKGTN----IALSIYSVLHDPKIY-PNPEKFDPERFLNAK------------   154
CYP5856B-fragment11(169617)Conco1      IPEDNIVIPKGTT----IALSIYSILHDPKIY-PNPEKFDPERFLNAK------------   154
CYP5856B-fragment12(27598)Conco1       IPEDNVVIPKGTT----IALSIYSILHDPKVY-PNPEKFDPERFLNAK------------   147
CYP5856B1(167158)Conco1                IPEDNIVLPKGTS----VTLSIFSILNDPKIY-PNPEKFDPERFLNTK------------   385
CYP5856B-fragment2(34058)Conco1        IAEDNIVLPKGTN----VGLSIYSILNDPAVY-PNPEKFDPERFLNNK------------   193
CYP5856A-fragment1(26325)Conco1        IPTDKVVLPKGTQ----VMLSIYSIQHDPKIY-PKPEEFDPERFLVGK------------   142
CYP5856B-fragment5(30225)Conco1        IPGENVVLPKGTH----LNLSVYSIQKDPRIY-PNPDEFNPERFLNSK------------   142
CYP5854A-fragment4(78559)Conco1        ------------------------------------------------------------   133
CYP5854A-fragment2(6332)Conco1         ------------------------------------------------------------   236
CYP5854A-fragment3(6331)Conco1         ------------------------------------------------------------   219
CYP5854E-fragment3(14082)Conco1        L-----------------------IHHNPNDF-PDPYKFKLERFADNSN-----------   77
CYP5854E-fragment5(9574)Conco1         ------------------------------------------------------------   292
CYP5854E-fragment4(9575)Conco1         LSN-GLTIPAKTT----IFLQLWAIHHNPKAF-PDPFEFKPERFADNSN-----------   59
```

CYP5854E5(14156)Conco1	LSN-GLTIPAKTA----VFLHYWVVHHNPNDF-PDPYEFKPERFADISN-----------	401
CYP5854E-fragment2(42529)Conco1	LSN-GLTIPAKTA----LFLHYWVVHHNPNDF-PDPYEFKPERFDDISN-----------	80
CYP5854D1(9752)Conco1	LSN-GVTIPKGTD----IYCHLWASHHN-SSF-PNAHEFKPERFVDGDG-----------	419
CYP5854E-fragment13(126715)Conco1	--	144
CYP5854E8(11612)Conco1	LSN-GLTIPKNTN----IMLHLWGIHHNPNAF-ENPDEFLPERFDQLTN-----------	406
CYP5854E-fragment17(6815)Conco1	--	242
CYP5854E12(104896)Conco1	LSN-GLTIPKGTQ----IFFHLWGIHNNASAF-PNPEKFNPERFSDIHN-----------	417
CYP5854E15(12851)Conco1	LSN-GITIPKGTA----VFLHLWGLHHS-SAF-SKPDEFNPERFSDAHG-----------	409
CYP5854E16(80744)Conco1	LSN-GVTIPKNTQ----VLLHLWGLHHS-SAF-SKPDEFNPERFSDAHG-----------	416
CYP5854E17(80468)Conco1	LSN-GLTIPKSTP----IFLHLWGIHHNSKAF-PNPFEFNPDRFKDMGS-----------	367
CYP5854E13(77675)Conco1	LSN-GLTIPKGTS----LFLHLWGLHKNSSAF-HKPDEFNPNRFSDFIA-----------	294
CYP5854E14(12850)Conco1	LSN-GVTIPKGTH----IFLHLWGLHMNPSAF-SKPEEFNPDRFSDLHS-----------	409
CYP5854E7(11610)Conco1	LSN-GLTIPKGTA----VFFHLWGLHKNPTAF-PNPEEFNPDRFSDLHN-----------	417
CYP5854E-fragment16(8906)Conco1	LSN-GVTIPKNTK----VFLHLWGLHHS-SAF-SKPEEFNPERFSDPHG-----------	157
CYP5854A3(78558)Conco1	FSN-GVHVPVGKI----VSIHMWGLLHSPKYY-PNPDKFDPERFRDPNC-----------	242
CYP5854A1(154349)Conco1	LSN-GVEIPEGKT----VTLHMWGLFHNPKYF-PEPNKFDPERFRDPNC-----------	421
CYP5854A2(17189)Conco1	FSN-GVQVPEGRM----VNLHMWGLFHNPKYF-PEPNKFDPERFRDPNC-----------	422
CYP5854A-fragment1(6337)Conco1	--	0
CYP5854B1(5669)Conco1	LTD-SIKLTKNTP----VFLMMYQVHQNPRYF-SNPEKFDPERFRDPAS-----------	421
CYP5854B2(5666)Conco1	LTN-SLYIPARTA----VFVMLWKVHHQSEYF-AKPEVFDPERFRDPAS-----------	418
CYP5854B3(78236)Conco1	LTN-TIYLPAKTP----VFILPWQTHQQSQYF-SNPKEFNPERFRDPSS-----------	378
CYP5854B(38119)Conco1	LTN-SIYLPSSTP----VYLLMWQVHKQSKHF-PDPKLFNPERFRDPSS-----------	127
CYP5854C1(140971)Conco1	LSN-GITIPKGTV----VSLNLWAIHHNPEFY-ENPGEFNPERFKLNKN-----------	326
CYP5854E-fragment15(30483)Conco1	LSN-GLMVPKGTQ----VHLQLWAIHHDPKIF-NNPDVFDPERFRE--------------	100
CYP5854E10(11616)Conco1	LIN-GLTIPKGTK----VMVNLWGIHHNDKAF-KNPEEFNPYRFESLTT-----------	307
CYP5854E-fragment14(126716)Conco1	LSN-GLTIPKDTN----LWVNLWGIHHNDKAF-KNPEEFNPYRFESLSS-----------	176
CYP5854E-fragment1(11608)Conco1	--	152
CYP5854E6(11609)Conco1	LSN-GLVVPKGTY----IYLHLWGIHHNPSTF-PCPDEFNPNRFDDISN-----------	393
CYP5854E-fragment6(12533)Conco1	LGK-GLIVPKNTP----IFVHLWGIHHNPITF-PDPYEFKPERFSDISN-----------	73
CYP5854E-fragment7(8022)Conco1	--	0
CYP5854E9(11613)Conco1	FSN-GLSVPKDTP----IFVHMCGVHYNPSAF-SNPFEFNPERFSDISS-----------	374
CYP5854E-fragment12(13308)Conco1	--	285
CYP5854E-fragment10(13854)Conco1	LSN-GITIPKNTP----IFLHLWGVHHNPSAF-PNPFEFNPNRFEDISN-----------	223
CYP5854E-fragment11(13446)Conco1	--	139
CYP5854E2(13461)Conco1	LSN-GITIPKNTP----IFLHLWGVHHNPSNF-SNPFEFNPNRFEDISN-----------	418
CYP5854E-fragment8(12835)Conco1	LSN-GMTIPKNTP----IFLHLWGVHHNPSAF-PNPFEFNPNRFEDISN-----------	90
CYP5854E4(14118)Conco1	LRN-GVTIPKNTP----VFVQLWGVHHNPSTF-PNPFEFNPNRFEDISS-----------	417
CYP5854E11(11719)Conco1	LSN-GITIPKDTF----VIVHLWGAHNNPSAF-PNPFEFNPNRFEDISN-----------	404
CYP5854E1(13460)Conco1	LSN-GVTIPKNTP----VFVQLWGVHHNPSAF-PNPFEFNPNRFEDISN-----------	333
CYP5854E3(12836)Conco1	LSN-GMKIPKDTP----VFLHLWGVHHNPSAF-PNPFEFNPNRFEDISN-----------	90
CYP5854E-fragment9(11720)Conco1	LSN-GMTIPKDTP----IFLHLWGVHHNPSAF-PNPFEFNPNRFEDISN-----------	90

CYP5854A-fragment5(6317)Conco1	--	126
CYP5856B-fragment6(2022)Conco1	-------------YDMDTYMSFSRSSGVCARMNFGLIEQKIFLSLVLKNLELIIAKL---	101

```
CYP5862A1(43654)Conco1                 ------------TKTEPQFLSFGFGTHACPGRFLAIQEISTILSVIIRNYEISTQDGKPH   283
CYP5862A-fragment1(25548)Conco1        ------------TKVEPQNIAFGLGQHACPGRFLAIQEISTILSVIIRNYKVSTQDGKPH   212
CYP-fragment(71373)Conco1              -------PDFSMDNNADSFCTFSGGQRRCVGEGLARIEIRAFIK-LMLNFRFKTDDR---   262
CYP5855A3(13529)Conco1                 ------------------------------------------------------------   366
CYP5855A-fragment1(11397)Conco1        ------------------------------------------------------------   255
CYP5855A1(44820)Conco1                 ------------ETYKSKLYTFSSGPRSCIGRELAWMEMFLVLSHLLHRFELELVPD---   390
CYP5855A2(30387)Conco1                 ------------EVYKSRLYAFSTGPRGCIGKELAWTEMLLILSHLLHRFNI--------   386
CYP5855A4(43538)Conco1                 ------------EEYKSKLYAFSTGPRACIGKELAWMEMLLILSHLLHRFDIEIDPK---   389
CYP51F14(41890)Conco1                  VIEEHVWATMNSSSARSSYLPFGAGRHRCVGEQFAYMQISTIISVILRNYRIKLNTE---   307
CYP5859A-fragment2(11509)Conco1        -------------DLKSQIMIFSQGPRGCLGRALTWMEIFFIITVMAQRYNFKLCPGQEK   84
CYP5859A-fragment1(29099)Conco1        ------------TQLKSQLMVFSQGPRGCPG-----------------------------   158
CYP5859A-fragment3(168491)Conco1       ------------TKLKSQNMVFSQGPRGCPGRALAWMEMFFITTAIAQRYNFRLCPGQEK   80
CYP5859A1(11907)Conco1                 ------------TELKSQLMVFSQGPRGCPGRALAWTEMFLITVALAQRYDMKLCEGQDH   471
CYP5859A2(11511)Conco1                 ------------TELKSQLMVFSQGPRGCPGRALAWTEMFLVTVSLAQRYDMKLCKGQDH   471
CYP5860A3(24597)Conco1                 ------------DELKHSIIAFGRGSTTCIGKQLALIQITLTLVGLLTRFEL--------   438
CYP5860A1(168890)Conco1                ------------TSIKDSIYAFGKGSRVCIGKHLAWMEITLTLIGILTKFELKPLPAWDK   478
CYP5860A2(12313)Conco1                 ------------PSIKDSIYAFGKGSRVCIGKHLAWMEITLTLIGILTKFELKPLPVWDK   478
CYP5863A1(2687)Conco1                  ------------LIIRSELIPFGMGKRRCPGEGLAKMEMFLIMANILNAFTFDAVDN---   469
CYP5857A2(2640)Conco1                  ------------DKLKDMLFAFSQGPRNCVGKNLAWLEIYITIANLIRQYDFSMPTG---   457
CYP5857A4(2643)Conco1                  ------------DELKDMLFAFSLGTRGCVGMNLAWLEMYISLANVTRQFDFTIPEG---   289
CYP5857A-fragment1(12733)Conco1        ------------------------------------------------------------   223
CYP5857A3(2641)Conco1                  ------------EKLKDMFFAFGLGPRGCIGKNLAMLEIYITLANLIRKFNFSMPKG---   441
CYP5857A7(2648)Conco1                  ------------QKLKDKLFTFSLGARRCAGQNLAWLEMHITLANIIRQYDFSMPAD---   456
CYP5857A6(2646)Conco1                  ------------QKLKSMLFSFGFGPRDCSGKSLAWLEMYISLANVIRKFDFTMPED---   308
CYP5857A-fragment2(24936)Conco1        ------------DELKNMLFSFGLGPRGCVGKNLAWLEMYITIANIIRQFDFSMPKD---   170
CYP5857A8(2649)Conco1                  ------------EKLKDMLFIFGLGPRGCVGKNLAWLEMYISLANVIRQFDFSMPES---   457
CYP5857A1(14904)Conco1                 ------------EKLKDMFFAFGLGPRGCVGKNLAWLEMYISLANVIRQFDFSMPKG---   457
CYP5857A5(2645)Conco1                  ------------EKLKDMFFTFGLGPRGCVGKNLAWLEMYISLANVIRQFDFSMPEG---   308
CYP5855B1(4344)Conco1                  ------------------------------------------------------------   369
CYP5855B-fragment1(4348)Conco1         ------------------------------------------------------------   250
CYP5864A1(18764)Conco1                 ------------TGLSEPFIGFSKGTRMCVGKDFALRVLFLVTIEILSKFDLHCDYE---   447
CYP5855-fragment1(2292)Conco1          ------------------------------------------------------------   97
CYP5855C1(34338)Conco1                 ------------EKNKSQLISFSVGPRSCIGRELAWNEMYLVLTNIIRNFKMELVDT---   471
CYP5855C2(2240)Conco1                  ------------DKNKSQLMNFSVGPRSCIGRELAWNEIYLVLTNIIRHFKMELIDT---   451
CYP5855C3(2245)Conco1                  ------------EKNKSQLLTFGTGPRSCIARDLAWNEIYFVLANLIRHFKMELIDT---   464
CYP5855C4(21824)Conco1                 ------------EKNKAQLMTFGAGPRSCIARDLAWNEIILVFANIIRHFR---------   439
CYP5855C5(2262)Conco1                  ------------------------------------------------------------   375
CYP5855C9(2252)Conco1                  ------------ENNKAQLMSFGAGPRSCIGRELAWNEMYLVLTNLIRNFRMELIDT---   453
CYP5855C6(27512)Conco1                 ------------EKNKSMLMTFGAGPRSCIGRELAWNEMYLVLSNLIR------------   380
CYP5855C-fragment3(34299)Conco1        ------------EKNKAQLMTFGTGPRSCIGRELAWNEMYLVLTNLIRNFRMELIDK---   223
CYP5855C-fragment1(34389)Conco1        ------------EKNKAQLMTFGAGPRSCIGRELAWNEMYLVLSNLIRNFRMEL------   220
CYP5855C7(34382)Conco1                 ------------DNNKAKLMSFGAGPRSCIGRELAWNEMYLVLSNLIRNFKMELVDK---   464
CYP5855C8(2259)Conco1                  ------------------------------------------------------------   377
CYP5855D7(2257)Conco1                  ------------EKNKSLLFTWGFGPTSCVGRELAWAEIFLVLVNLLRNFNFELVDK---   400
CYP5855D1(29975)Conco1                 ------------ESNKSKLLSFGLGPTSCVGRELAWMEIYLVLVELIR------------   334
```

```
CYP5855D-fragment5(29026)Conco1        ------------ELNKSKMFNFGIGPTSCIGRELAWSEIFLVLANLLRSFELEMID----   200
CYP5855D-fragment7(2254)Conco1         ------------------------------------------------------------   217
CYP5855C-fragment2(30530)Conco1        ------------EKSMENFIGFGSGPRSCIGKDLAWAELFLVLANLIR------------   164
CYP5855E1(38687)Conco1                 ------------EENKGKLLTFSMGPRGCVGRDLAWSEIYLVIVKLIRMFRMELVEK---   378
CYP5855D6(6708)Conco1                  ------------EFNKEKLFNWSTGPRSCIGRDLAKAEIYLVLTNLIRNFNFELIDK---   456
CYP5855D-fragment1(169043)Conco1       ------------EANKNRLVTFGLGPRSCIGRDLARSEIYLVLANLLRNFNFELVDK---   124
CYP5855D-fragment6(24158)Conco1        ------------DTYKNKLVTFGLGPRSCIGRDLARNEIYLVLANLIRHFNFEIVD----   67
CYP5855D4(54364)Conco1                 ------------EINKNRLFTFGIGTRSCIGRDLAKNEIYLVLSNLIRHFSFELVDK---   464
CYP5855D5(76752)Conco1                 ------------------------------------------------------------   311
CYP5855D-fragment2(2269)Conco1         ------------------LAGFGLGTRSCVGRDLSWNELYLVLANLIRHFNFELVNK---   39
CYP5855D3(54372)Conco1                 ------------EANKAKSIPFGLGNRSCIGRDLAWYELYMLLADLIRHFDFELVDE---   462
CYP5855D-fragment3(28450)Conco1        ------------ETNRAKLAGFGLGTRACVGRDLAWSELFLVIANLIRNFNFELVD----   120
CYP5855D8(45649)Conco1                 ------------EAKKARLAGFGLGPRSCIG-----------------------------   350
CYP5855D2(2268)Conco1                  ------------EAKKAKLVGFGLGPRSCIGRDLAWNEIFLVLANLIRHFSFELVDK---   453
CYP5855D-fragment4(27931)Conco1        ------------EAKKAKLVGFGLGPRSCIGRDLAWNEILLVLANLIRHFNFELVD----   153
CYP5858A-fragment7(4474)Conco1         ------------------------------------------------------------   220
CYP5856B-fragment1(2011)Conco1         ------------------------------------------------------------   203
CYP5858A-fragment1(68816)Conco1        ------------------------------------------------------------   178
CYP5858A-fragment2(31161)Conco1        ------------SKADNFWFGFSTGARMCLGSSF--------------------------   167
CYP5858A-fragment5(21198)Conco1        ------------TKVDSNWFSFSNGSRICIG-----------------------------   166
CYP5858A-fragment3(23423)Conco1        ------------TKFESNWFTFSSGSRICLGAGFSIAQQKILLSNII-------------   167
CYP5858A-fragment4(23037)Conco1        ------------TKFESNWFTFSSGGRICLGAGFSLTQQKIIL-----------------   164
CYP5858A-fragment6(21524)Conco1        ------------KKFESNWFPFSSGSRVCLGATFSTTQQKIVLSYIL-------------   167
CYP5858A-fragment8(27368)Conco1        ------------NKFESNWFAFSSGSRACIGAGFSTVEQKVVLSYIL-------------   167
CYP5858A1(68836)Conco1                 ------------TKLESNWFSFSVGNRMCLGQSFSIVEQKIVLSSLIRRYIVKPSGGRVP   472
CYP5858A2(77667)Conco1                 ------------NKLDSNWFSFGAGNRMCLGQSFTLTQQKIVLSSILRRYRVKHSNV-EN   472
CYP5858A3(36631)Conco1                 ------------NKLDSNWFSFGAGNRMCLGQSFTLTQQKIVLSSILRRYRVKHSNV-EN   398
CYP5861A-fragment2(21280)Conco1        ------------MKHSFAWSTFGGGQRLCIGKDFS-------------------------   151
CYP5861A1(30625)Conco1                 ------------AYPATSWSPFGGGERMCSGLGFSMMEQRITLALLLLNYKFELN-----   423
CYP5861A-fragment1(36094)Conco1        ------------PHPATSWSPFGAGERMCSGSGFSMMEQRISLALLLLNYELEFSQESK-   198
CYP5856A4(2008)Conco1                  -------------YDTDTYLPFGGGSRMCLGMNFSLTEQKVYLSMLLQKFTIQIHKD-NP   466
CYP5856B2(45436)Conco1                 -------------YDTNVYMPFGGGSRMCIGTNFSLIEQKVYLSMLVQKFILNIKKT-NP   458
CYP5856B-fragment7(34125)Conco1        -------------YDTDVYMPFGGGSRMCIGSNFSLIEQKVFLIMLLQKFKVDIKPE-NA   199
CYP5856B3(76636)Conco1                 -------------YDTDVYMPFGGGSRMCVGFNFSLMEQKVFFIMLLQKFIIEIMPD-NP   431
CYP5856B-fragment3(29023)Conco1        -------------HESDVYMPFGGGSRMCIGSNFSLMEQKVYLTMLLQKFTIDIKTT---   203
CYP5856B-fragment4(76635)Conco1        -------------YDTDVYMPFGGGSRMCIGFNFSLMEQKIYLIMLLQRFDIHIGSN-NP   240
CYP5856A1(34083)Conco1                 -------------YDTDIYLPFGGGSRMCVGMGFSLMEQRVYLTLLLQKFTIEIKED-NH   466
CYP5856A2(34094)Conco1                 -------------YDTDIYLPFGGGSRMCIGMGFSLMEQRVYLAMLLQKFTLEIKQD-NP   431
CYP5856A3(29445)Conco1                 -------------HETDIYLPFGGGSRMCIGMGFSLMEQRVYLAMLLQKFNIEIKED-NP   364
CYP5856B-fragment9(30415)Conco1        -------------YDIDTYMPFGGGSRMCVGISFSLMQQKVFFALLLQKFELGIAKD---   191
CYP5856B-fragment10(35455)Conco1       -------------YDIDTYMPFGGGSRMCVGISFSLMQQKVFLALLLQKFDLRIAND-NP   200
CYP5856B-fragment8(44763)Conco1        -------------YDVDTYMPFGGGSRMCVGINFSLVEQKVFFALLLQKFDLKIAKD-NP   200
CYP5856B-fragment11(169617)Conco1      -------------YDVDTYMPFGGGSRMCVGIKFTLMEQKVFFALLLQKFDLRIAKD-NP   200
CYP5856B-fragment12(27598)Conco1       -------------YDVDTYMPFGGGSRMCVGIKFTLMEQKVFFALLLQKFDLRIAKD---   191
CYP5856B1(167158)Conco1                -------------YDIDTYMPFGGGSRMCVGMNFSLMEQKIFLSLLLQKFDLRITKD-NP   431
```

```
CYP5856B-fragment2(34058)Conco1        -------------YEADTYIPFGGGSRICVGMNFSLMEQKIFLALLLQKFDLSVTKD-NP   239
CYP5856A-fragment1(26325)Conco1        -------------YDTDIYMPFGGGTRMCVGMNFSLMEQKVYLSLLLQKFTLGI------   183
CYP5856B-fragment5(30225)Conco1        -------------YDTDVYMPFGGGTRMCVGMNFSLMEQKVFLIMLLQKFHLNIDKN-NP   188
CYP5854A-fragment4(78559)Conco1        ------------------------------------------------------------   133
CYP5854A-fragment2(6332)Conco1         ------------------------------------------------------------   236
CYP5854A-fragment3(6331)Conco1         ------------------------------------------------------------   219
CYP5854E-fragment3(14082)Conco1        -------------EESRNWQPFGNGPRKCIGSTFALLEQRVTLSICYRSLNFVLARI-IQ   123
CYP5854E-fragment5(9574)Conco1         ------------------------------------------------------------   292
CYP5854E-fragment4(9575)Conco1         -------------EESKNWQAFITGPRTCLGSTFSLTEQRVTLSMLLQKFEFCISED-NP   105
CYP5854E5(14156)Conco1                 -------------EATKNWQSFGTGPRKCIANTFALMETRVTISMLLQKFEFCISED-NP   447
CYP5854E-fragment2(42529)Conco1        -------------EATKNWQPFGTGPRTCKNI*---------------------------   99
CYP5854D1(9752)Conco1                  -----------------EWCPFFLGTRKCIGMNFSLLELKVNLVLILQQFDLSMSES-NP   461
CYP5854E-fragment13(126715)Conco1      ------------------------------------------------------------   144
CYP5854E8(11612)Conco1                 -------------EESRNWQPFATGARSCIGMSFSLVEQRVTIAMLLQAFEFSISPQ-NP   452
CYP5854E-fragment17(6815)Conco1        ------------------------------------------------------------   242
CYP5854E12(104896)Conco1               -------------QESRNWQPFMTGPRSCIGMTLSLMEQRVTIAMLLQKFEFIIQKE-NP   463
CYP5854E15(12851)Conco1                -------------EES--------------------------------------------   412
CYP5854E16(80744)Conco1                -------------EESRN------------------------------------------   421
CYP5854E17(80468)Conco1                -------------EESRNWQPFLTGIRSCIGTTFSLMEQRVTIAMLLQKFEFSITNS-NP   413
CYP5854E13(77675)Conco1                ----------------------------------------------KKVFEFSINKI-NP   307
CYP5854E14(12850)Conco1                -------------EESRNWLAFSTGPRSCIGMTFSLMEQRVTIAMLLQKFEFSITKN-NP   455
CYP5854E7(11610)Conco1                 -------------EESRNWQPFLTGPRSCIGMTFSLMEQRVTIAMLLQKFEFSIGKE-DP   463
CYP5854E-fragment16(8906)Conco1        -------------EESRNWQPFLTGPRSCIGMTFSLMEQRVTIAMLLQKFEFSIAKS-NP   203
CYP5854A3(78558)Conco1                 -------------EANKNWMPFLIGARTCIGMSFSLMEQRVALAMLVQTYTFEITPE-NP   288
CYP5854A1(154349)Conco1                -------------EANKSWIPFITGARTCIGMSFSLMEQRVTLAMLLQTYTFKISPG-HP   467
CYP5854A2(17189)Conco1                 -------------EANKSWIPFLTGSRTCIGMSFSLMEQRVALSMLVQAYIFQINSD-HP   468
CYP5854A-fragment1(6337)Conco1         -----------------------------------LMEQRVALVMLVQAYTFQIPAD-HP   24
CYP5854B1(5669)Conco1                  -------------VESKNWQPFITGPRACIGMTLSLMEQRISLVLLLQKFKFYIKES-NP   467
CYP5854B2(5666)Conco1                  -------------VESKNWQPFITGPRACIGMTLSLIEQRISLVLLLQKFYFEIDPS-NP   464
CYP5854B3(78236)Conco1                 -------------VESKNWQPFITGPRACIGMTLSLMEQRVSLVLLLQKFIFSIDES-NP   424
CYP5854B(38119)Conco1                  -------------PESKNWLPFLQGPRACNNFYNTL*-----------------------   150
CYP5854C1(140971)Conco1                -------------GENQNWQPFLMGQRSCIGMSFSLTEIKVSLILLLQQFEFTLSEN-NP   372
CYP5854E-fragment15(30483)Conco1       ------------------------------------------------------------   100
CYP5854E10(11616)Conco1                -------------EDSRNWQPFITGARTCVGNTFSLVEQRVTIAMLLQKFEFSIDSN-NP   353
CYP5854E-fragment14(126716)Conco1      -------------DDSRNFLSFISGARSCVGNTFSLVEQRVTIAMLLQKFEFSINSN-NP   222
CYP5854E-fragment1(11608)Conco1        ------------------------------------------------------------   152
CYP5854E6(11609)Conco1                 -------------KQSKLFQAFTLGNRTCLGSAFSLMEQRVTISMLLQKFEFSISND-NP   439
CYP5854E-fragment6(12533)Conco1        -------------EANKNWMPFTLGARICPGSTFSFMEQRVTLAMLLQKFDFSISND-NP   119
CYP5854E-fragment7(8022)Conco1         -------------------MLFILGARICSGSTFTFMEQRVTLAMLLQKFDFSISND-NP   40
CYP5854E9(11613)Conco1                 -------------EESKNWLAFGLGNRTCLGFTFSLMEQRVTLAMLLQKFEFSINKE-NP   420
CYP5854E-fragment12(13308)Conco1       ------------------------------------------------------------   285
CYP5854E-fragment10(13854)Conco1       -------------QES-------------IGSTFSLMEQRVTIAMLLQKFEFSISSD-NP   256
CYP5854E-fragment11(13446)Conco1       ------------------------------------------------------------   139
CYP5854E2(13461)Conco1                 -------------QESKNWQPFILGNRTCIGSTFSLVEQRVTIAMLLQKFEFSISSD-NP   464
CYP5854E-fragment8(12835)Conco1        -------------QESKNWQPFISGNRACIGSTFSLMEQRVTLAMLLQKFEFSISSD-NP   136
```

```
CYP5854E4(14118)Conco1              -------------QESKNWQPFLYGSRTCIGSTFSLMEQRVTLAMLLQKFEFSISSD-NP   463
CYP5854E11(11719)Conco1             -------------QESKNWQPFTLGNRTCLGSTFSLMEQRVTLAMLLQRFEFSISSD-NP   450
CYP5854E1(13460)Conco1              -------------QESKNWQPFLYGNRTCLGSTFSLMEQRVTLAMLLQKFEFSISSD-NP   379
CYP5854E3(12836)Conco1              -------------QESKNWQPFTLGNRTCIGGTFSLMEQRVTLAMLLQKFEFSISSD-NP   136
CYP5854E-fragment9(11720)Conco1     -------------QESKNWQPFILGNRTCLGSTFSLMEQRVTLAMLLQKFEFSISSD-NP   136


CYP5854A-fragment5(6317)Conco1      ------------------------------------------------------------   126
CYP5856B-fragment6(2022)Conco1      TQ--IKNN*---------------------------------------------------   107
CYP5862A1(43654)Conco1              PY--MVKPAQFD-LP----KGEPLIFRK---INN*-------------------------   307
CYP5862A-fragment1(25548)Conco1     PY--KVVPDQFA-LP----KGEPLIFTK---I----------------------------   234
CYP-fragment(71373)Conco1           ----LVPNSESAITA----KPERIFIGVF*------------------------------   283
CYP5855A3(13529)Conco1              ------------------------------------------------------------   366
CYP5855A-fragment1(11397)Conco1     ------------------------------------------------------------   255
CYP5855A1(44820)Conco1              AK--LTPVSRFLLSP----KENSLYVNLKKRVF*--------------------------   417
CYP5855A2(30387)Conco1              ------------------------------------------------------------   386
CYP5855A4(43538)Conco1              AN--IIPANGFLLTP----KEKCIYATFKKRIF*--------------------------   416
CYP51F14(41890)Conco1               AGFPNINFATLICSP----LDPN-LIEYEPRN*---------------------------   334
CYP5859A-fragment2(11509)Conco1     LG--NKPAIYFMMKS----STCSIKVEIVHRERLNFLTLSHLLTKSIY*-----------   126
CYP5859A-fragment1(29099)Conco1     ------------------------------------------------------------   158
CYP5859A-fragment3(168491)Conco1    LG--NKPIAYFMMKP----STYSIKVEMEHRKN*--------------------------   107
CYP5859A1(11907)Conco1              LG--DRPLTYFLTKP----ASYSLKAELTHRPQVSGS*----------------------   502
CYP5859A2(11511)Conco1              LG--DKPVSYFATKP----RSYSLKAEFTHRSQASSS*----------------------   502
CYP5860A3(24597)Conco1              ------------------------------------------------------------   438
CYP5860A1(168890)Conco1             VKGDDEIINLVHFKP----SSGRISCRLSKRETNTL*-----------------------   510
CYP5860A2(12313)Conco1              VQGDDEIINFVHFKP----TSGRISCRLSKRETSSL*-----------------------   510
CYP5863A1(2687)Conco1               SK--IDSEINFTAGSVRSPLPYELVFKTKERV*---------------------------   499
CYP5857A2(2640)Conco1               TT--LTEFDLFVLKG----KEEKLILNTTPRSN*--------------------------   484
CYP5857A4(2643)Conco1               TE--LTGFGLLVLKG----KEEKPILNIIPRSN*--------------------------   316
CYP5857A-fragment1(12733)Conco1     ------------------------------------------------------------   223
CYP5857A3(2641)Conco1               TT--MTDFDLFVLKA----KEEKLILKTVRRSN*--------------------------   468
CYP5857A7(2648)Conco1               AE--LTGFDLFVLKG----KEQKLLLNVFPRSN*--------------------------   483
CYP5857A6(2646)Conco1               TQ--LTEFNLFLLKG----KEQKLLLNVFPRS*---------------------------   334
CYP5857A-fragment2(24936)Conco1     TE--LTE-----------------------------------------------------   175
CYP5857A8(2649)Conco1               TQ--LTGFDLFVLKG----KEEKLLLNATPRSS*--------------------------   484
CYP5857A1(14904)Conco1              TQ--LTGFDLFVLKG----KEEKLLLNTSPRSC*--------------------------   484
CYP5857A5(2645)Conco1               AE--LTEHDLFALKG----KEQKLLLNATLRAN*--------------------------   335
CYP5855B1(4344)Conco1               ------------------------------------------------------------   369
CYP5855B-fragment1(4348)Conco1      ------------------------------------------------------------   250
CYP5864A1(18764)Conco1              QEKRDESVCQVTLLP----TSGKFELEFQAINA*--------------------------   476
CYP5855-fragment1(2292)Conco1       ------------------------------------------------------------   97
CYP5855C1(34338)Conco1              D---LTPTNKFLYRP----LEKRMRVRMEKRY*---------------------------   496
CYP5855C2(2240)Conco1               D---LTPANKFLYKP----LEKRMRVRMEKR*----------------------------   475
CYP5855C3(2245)Conco1               K---LTPAYKIFFKP----EEKRMRVKITSRY*---------------------------   489
CYP5855C4(21824)Conco1              ------------------------------------------------------------   439
```

```
CYP5855C5(2262)Conco1                 ------------------------------------------------------------   375
CYP5855C9(2252)Conco1                 D---LTPCSTVFYKP----KEMRMKVKIFIRK*---------------------------   478
CYP5855C6(27512)Conco1                ------------------------------------------------------------   380
CYP5855C-fragment3(34299)Conco1       E---LTPTFKFLYKP----EEKRMRVKISLRN*---------------------------   248
CYP5855C-fragment1(34389)Conco1       ------------------------------------------------------------   220
CYP5855C7(34382)Conco1                E---LTPTFKFFYTP----KEKRMRVKFSART*---------------------------   489
CYP5855C8(2259)Conco1                 ------------------------------------------------------------   377
CYP5855D7(2257)Conco1                 T---LTHYLTFMNKP----KEKRFRVRISRRVR*--------------------------   426
CYP5855D1(29975)Conco1                ------------------------------------------------------------   334
CYP5855D-fragment5(29026)Conco1       ------------------------------------------------------------   200
CYP5855D-fragment7(2254)Conco1        ------------------------------------------------------------   217
CYP5855C-fragment2(30530)Conco1       ------------------------------------------------------------   164
CYP5855E1(38687)Conco1                EP--MKPELYFLYTP----KDGKFDVKLTKRV*---------------------------   404
CYP5855D6(6708)Conco1                 E---LTPMNISIYKP----VERRFRVNVSRRS*---------------------------   481
CYP5855D-fragment1(169043)Conco1      E---LIPTNKFLYKP----EGKRFIVKVARRV*---------------------------   149
CYP5855D-fragment6(24158)Conco1       ------------------------------------------------------------   67
CYP5855D4(54364)Conco1                E---LTPNNKFLYKP----KEKRFKVKLSRRV*---------------------------   489
CYP5855D5(76752)Conco1                ------------------------------------------------------------   311
CYP5855D-fragment2(2269)Conco1        E---VVSIYKFIHRP----IDKKLGVKLTRRSKN*-------------------------   66
CYP5855D3(54372)Conco1                V---LTPDYKLVYRP----KEQTFRIKVSRKSH*--------------------------   488
CYP5855D-fragment3(28450)Conco1       ------------------------------------------------------------   120
CYP5855D8(45649)Conco1                ------------------------------------------------------------   350
CYP5855D2(2268)Conco1                 E---LIPVNYFILKP----KGNSFKVKISRRS*---------------------------   478
CYP5855D-fragment4(27931)Conco1       ------------------------------------------------------------   153
CYP5858A-fragment7(4474)Conco1        ------------------------------------------------------------   220
CYP5856B-fragment1(2011)Conco1        ------------------------------------------------------------   203
CYP5858A-fragment1(68816)Conco1       ------------------------------------------------------------   178
CYP5858A-fragment2(31161)Conco1       ------------------------------------------------------------   167
CYP5858A-fragment5(21198)Conco1       ------------------------------------------------------------   166
CYP5858A-fragment3(23423)Conco1       ------------------------------------------------------------   167
CYP5858A-fragment4(23037)Conco1       ------------------------------------------------------------   164
CYP5858A-fragment6(21524)Conco1       ------------------------------------------------------------   167
CYP5858A-fragment8(27368)Conco1       ------------------------------------------------------------   167
CYP5858A1(68836)Conco1                CGRELNVKAPFLLRT------DNLRLDFERVLNS*-------------------------   500
CYP5858A2(77667)Conco1                IGKPLKTRPPFLLRS------DNLKLDLERI*----------------------------   497
CYP5858A3(36631)Conco1                IGKPLKTRLPFLLRS------DNLRLDLERV*----------------------------   423
CYP5861A-fragment2(21280)Conco1       ------------------------------------------------------------   151
CYP5861A1(30625)Conco1                ------------------------------------------------------------   423
CYP5861A-fragment1(36094)Conco1       RDSPLDYLCNSTLSS------EGVIVNFNEILY*--------------------------   225
CYP5856A4(2008)Conco1                 DFERLRMNGLIFSKA------KDLKLNFVQRF*---------------------------   492
CYP5856B2(45436)Conco1                DYENLRIRGLGLNRP------QDLKLNFVPRF*---------------------------   484
CYP5856B-fragment7(34125)Conco1       DFEKLRIRGTVHTKP------EDLNLNFVPVF*---------------------------   225
CYP5856B3(76636)Conco1                DFEKLRIMELGQIRP------LELKLNFKPVL*---------------------------   457
CYP5856B-fragment3(29023)Conco1       ------------------------------------------------------------   203
CYP5856B-fragment4(76635)Conco1       DFDKLRIKGMGMIKP------TDLKLNFEPRF*---------------------------   266
```

```
CYP5856A1(34083)Conco1             DFNSLRLSGLVLTKA------KDLKLNFHPRF*---------------------------   492
CYP5856A2(34094)Conco1             DYEQLRMVGLRLTKA------QDLRLNFTPRF*---------------------------   457
CYP5856A3(29445)Conco1             DYDCLRLNGIALTKA------KDLKLNF--------------------------------   386
CYP5856B-fragment9(30415)Conco1    ------------------------------------------------------------   191
CYP5856B-fragment10(35455)Conco1   DYEQLRVSGFGLTRP------TDLKISFEARF*---------------------------   226
CYP5856B-fragment8(44763)Conco1    DHEQLRISGFGLTRP------TDLKINFEARF*---------------------------   226
CYP5856B-fragment11(169617)Conco1  DYEQLRVSGFGLSRP------TDLKISFEARF*---------------------------   226
CYP5856B-fragment12(27598)Conco1   ------------------------------------------------------------   191
CYP5856B1(167158)Conco1            DYEQLRVSGLGLTRP------MDLKLNFEARL*---------------------------   457
CYP5856B-fragment2(34058)Conco1    DYEKLRISGLGLTRP------MDLKLNFEARF*---------------------------   265
CYP5856A-fragment1(26325)Conco1    ------------------------------------------------------------   183
CYP5856B-fragment5(30225)Conco1    DFK---------------------------------------------------------   191
CYP5854A-fragment4(78559)Conco1    ------------------------------------------------------------   133
CYP5854A-fragment2(6332)Conco1     ------------------------------------------------------------   236
CYP5854A-fragment3(6331)Conco1     ------------------------------------------------------------   219
CYP5854E-fragment3(14082)Conco1    I----TIS*---------------------------------------------------   127
CYP5854E-fragment5(9574)Conco1     ------------------------------------------------------------   292
CYP5854E-fragment4(9575)Conco1     NYHKLNVTSIGIVCP------NDLSLEIKIRT*---------------------------   131
CYP5854E5(14156)Conco1             NYHKLNIASNFLLYP------KDLSLEIKIRT*---------------------------   473
CYP5854E-fragment2(42529)Conco1    ------------------------------------------------------------   99
CYP5854D1(9752)Conco1              DYEKLRLNSMNVIKP------LDLKLKFKNLL*---------------------------   487
CYP5854E-fragment13(126715)Conco1  ------------------------------------------------------------   144
CYP5854E8(11612)Conco1             SYEKIHISTSALVKP------DNLKLIIKLRE*---------------------------   478
CYP5854E-fragment17(6815)Conco1    ------------------------------------------------------------   242
CYP5854E12(104896)Conco1           NYEKLFITPMGLVHP------KDLTLTVKLRD*---------------------------   489
CYP5854E15(12851)Conco1            ------------------------------------------------------------   412
CYP5854E16(80744)Conco1            ------------------------------------------------------------   421
CYP5854E17(80468)Conco1            DYDKLRITASGIVRP------RDLHLHIKSRA*---------------------------   439
CYP5854E13(77675)Conco1            DYEKLRITPFGIVRP------LDLHLVIKLRQ*---------------------------   333
CYP5854E14(12850)Conco1            DYEKLRITPSGIVRP------RDLHLVVKLRK*---------------------------   481
CYP5854E7(11610)Conco1             NYENLNISPSGLVHP------KDLALTIKLRA*---------------------------   489
CYP5854E-fragment16(8906)Conco1    DYDKLRISPSGNVRP------LDLSLSIKLRS*---------------------------   229
CYP5854A3(78558)Conco1             DYEKLRIVTFGFPRA------RDFHVDLVRINQS*-------------------------   316
CYP5854A1(154349)Conco1            DYERLRLPVMGFPKP------TDFKVDLIRRIPN*-------------------------   495
CYP5854A2(17189)Conco1             DYEKLRLPVFGFPRP------EDFKVDLIRRFTN*-------------------------   496
CYP5854A-fragment1(6337)Conco1     DYDKLRLSLFGFPRP------RDFKVDLIRRVPN*-------------------------   52
CYP5854B1(5669)Conco1              DYKKLRLNSSGILRP------RDLHLDFIKLI*---------------------------   493
CYP5854B2(5666)Conco1              DYDRLRLNSSGMLCP------KDLHLDIRQRI*---------------------------   490
CYP5854B3(78236)Conco1             DYKQLRLTTNGIIRP------RDLRLNLTQRI*---------------------------   450
CYP5854B(38119)Conco1              ------------------------------------------------------------   150
CYP5854C1(140971)Conco1            DFEKVRLNSNFMIFP------KDLRLNVKNRLY*--------------------------   399
CYP5854E-fragment15(30483)Conco1   ------------------------------------------------------------   100
CYP5854E10(11616)Conco1            DFHSLRLTQGSIIRP------IDLHLKVKVRN*---------------------------   379
CYP5854E-fragment14(126716)Conco1  DFHKLRLTNNTIIHP------VDLHLNIKVRN*---------------------------   248
CYP5854E-fragment1(11608)Conco1    ------------------------------------------------------------   152
```

```
CYP5854E6(11609)Conco1               DYYRLRITDSVIIHP------KEVRLVIKSRF*---------------------------   465
CYP5854E-fragment6(12533)Conco1      DYNSLRVTAAIIAKP------KDLAICIKSRS*---------------------------   145
CYP5854E-fragment7(8022)Conco1       DYNSLRVTAAIIAKP------KDLAICIISRS*---------------------------   66
CYP5854E9(11613)Conco1               DYKQLRVTSSFISRP------QDLAITIKARV*---------------------------   446
CYP5854E-fragment12(13308)Conco1     ------------------------------------------------------------   285
CYP5854E-fragment10(13854)Conco1     DYHKLRIATGFIVRP------DDLSIRVKVRV*---------------------------   282
CYP5854E-fragment11(13446)Conco1     ------------------------------------------------------------   139
CYP5854E2(13461)Conco1               DYHSLRVS-SGIARP------KDLSVQVKIIA*---------------------------   489
CYP5854E-fragment8(12835)Conco1      DYHKLRIGSLGIVRP------KDLSIRVE--------------------IVKVPPELKNI   170
CYP5854E4(14118)Conco1               DYHKLRIGSLGIVRP------KELSIRVKVRA*---------------------------   489
CYP5854E11(11719)Conco1              DYHSLRIGSIGIVRP------KDLSIRIKARN*---------------------------   476
CYP5854E1(13460)Conco1               DYHSLRINSDFIVRP------YELSIRIKVRA*---------------------------   405
CYP5854E3(12836)Conco1               DYHKLRISSTKILRP------KDLSIQIKKMIFYFILGLITYIGYKINKFVKVPPELKSI   190
CYP5854E-fragment9(11720)Conco1      DYHKLRISSTGIVRP------KDLSIQVKVRA*---------------------------   162


CYP5854A-fragment5(6317)Conco1       ------------------------------------------------------------   126
CYP5856B-fragment6(2022)Conco1       ------------------------------------------------------------   107
CYP5862A1(43654)Conco1               ------------------------------------------------------------   307
CYP5862A-fragment1(25548)Conco1      ------------------------------------------------------------   234
CYP-fragment(71373)Conco1            ------------------------------------------------------------   283
CYP5855A3(13529)Conco1               ------------------------------------------------------------   366
CYP5855A-fragment1(11397)Conco1      ------------------------------------------------------------   255
CYP5855A1(44820)Conco1               ------------------------------------------------------------   417
CYP5855A2(30387)Conco1               ------------------------------------------------------------   386
CYP5855A4(43538)Conco1               ------------------------------------------------------------   416
CYP51F14(41890)Conco1                ------------------------------------------------------------   334
CYP5859A-fragment2(11509)Conco1      ------------------------------------------------------------   126
CYP5859A-fragment1(29099)Conco1      ------------------------------------------------------------   158
CYP5859A-fragment3(168491)Conco1     ------------------------------------------------------------   107
CYP5859A1(11907)Conco1               ------------------------------------------------------------   502
CYP5859A2(11511)Conco1               ------------------------------------------------------------   502
CYP5860A3(24597)Conco1               ------------------------------------------------------------   438
CYP5860A1(168890)Conco1              ------------------------------------------------------------   510
CYP5860A2(12313)Conco1               ------------------------------------------------------------   510
CYP5863A1(2687)Conco1                ------------------------------------------------------------   499
CYP5857A2(2640)Conco1                ------------------------------------------------------------   484
CYP5857A4(2643)Conco1                ------------------------------------------------------------   316
CYP5857A-fragment1(12733)Conco1      ------------------------------------------------------------   223
CYP5857A3(2641)Conco1                ------------------------------------------------------------   468
CYP5857A7(2648)Conco1                ------------------------------------------------------------   483
CYP5857A6(2646)Conco1                ------------------------------------------------------------   334
CYP5857A-fragment2(24936)Conco1      ------------------------------------------------------------   175
CYP5857A8(2649)Conco1                ------------------------------------------------------------   484
CYP5857A1(14904)Conco1               ------------------------------------------------------------   484
```

```
CYP5857A5(2645)Conco1                  ------------------------------------------------------------   335
CYP5855B1(4344)Conco1                  ------------------------------------------------------------   369
CYP5855B-fragment1(4348)Conco1         ------------------------------------------------------------   250
CYP5864A1(18764)Conco1                 ------------------------------------------------------------   476
CYP5855-fragment1(2292)Conco1          ------------------------------------------------------------   97
CYP5855C1(34338)Conco1                 ------------------------------------------------------------   496
CYP5855C2(2240)Conco1                  ------------------------------------------------------------   475
CYP5855C3(2245)Conco1                  ------------------------------------------------------------   489
CYP5855C4(21824)Conco1                 ------------------------------------------------------------   439
CYP5855C5(2262)Conco1                  ------------------------------------------------------------   375
CYP5855C9(2252)Conco1                  ------------------------------------------------------------   478
CYP5855C6(27512)Conco1                 ------------------------------------------------------------   380
CYP5855C-fragment3(34299)Conco1        ------------------------------------------------------------   248
CYP5855C-fragment1(34389)Conco1        ------------------------------------------------------------   220
CYP5855C7(34382)Conco1                 ------------------------------------------------------------   489
CYP5855C8(2259)Conco1                  ------------------------------------------------------------   377
CYP5855D7(2257)Conco1                  ------------------------------------------------------------   426
CYP5855D1(29975)Conco1                 ------------------------------------------------------------   334
CYP5855D-fragment5(29026)Conco1        ------------------------------------------------------------   200
CYP5855D-fragment7(2254)Conco1         ------------------------------------------------------------   217
CYP5855C-fragment2(30530)Conco1        ------------------------------------------------------------   164
CYP5855E1(38687)Conco1                 ------------------------------------------------------------   404
CYP5855D6(6708)Conco1                  ------------------------------------------------------------   481
CYP5855D-fragment1(169043)Conco1       ------------------------------------------------------------   149
CYP5855D-fragment6(24158)Conco1        ------------------------------------------------------------   67
CYP5855D4(54364)Conco1                 ------------------------------------------------------------   489
CYP5855D5(76752)Conco1                 ------------------------------------------------------------   311
CYP5855D-fragment2(2269)Conco1         ------------------------------------------------------------   66
CYP5855D3(54372)Conco1                 ------------------------------------------------------------   488
CYP5855D-fragment3(28450)Conco1        ------------------------------------------------------------   120
CYP5855D8(45649)Conco1                 ------------------------------------------------------------   350
CYP5855D2(2268)Conco1                  ------------------------------------------------------------   478
CYP5855D-fragment4(27931)Conco1        ------------------------------------------------------------   153
CYP5858A-fragment7(4474)Conco1         ------------------------------------------------------------   220
CYP5856B-fragment1(2011)Conco1         ------------------------------------------------------------   203
CYP5858A-fragment1(68816)Conco1        ------------------------------------------------------------   178
CYP5858A-fragment2(31161)Conco1        ------------------------------------------------------------   167
CYP5858A-fragment5(21198)Conco1        ------------------------------------------------------------   166
CYP5858A-fragment3(23423)Conco1        ------------------------------------------------------------   167
CYP5858A-fragment4(23037)Conco1        ------------------------------------------------------------   164
CYP5858A-fragment6(21524)Conco1        ------------------------------------------------------------   167
CYP5858A-fragment8(27368)Conco1        ------------------------------------------------------------   167
CYP5858A1(68836)Conco1                 ------------------------------------------------------------   500
CYP5858A2(77667)Conco1                 ------------------------------------------------------------   497
CYP5858A3(36631)Conco1                 ------------------------------------------------------------   423
```

```
CYP5861A-fragment2(21280)Conco1          ------------------------------------------------------------   151
CYP5861A1(30625)Conco1                   ------------------------------------------------------------   423
CYP5861A-fragment1(36094)Conco1          ------------------------------------------------------------   225
CYP5856A4(2008)Conco1                    ------------------------------------------------------------   492
CYP5856B2(45436)Conco1                   ------------------------------------------------------------   484
CYP5856B-fragment7(34125)Conco1          ------------------------------------------------------------   225
CYP5856B3(76636)Conco1                   ------------------------------------------------------------   457
CYP5856B-fragment3(29023)Conco1          ------------------------------------------------------------   203
CYP5856B-fragment4(76635)Conco1          ------------------------------------------------------------   266
CYP5856A1(34083)Conco1                   ------------------------------------------------------------   492
CYP5856A2(34094)Conco1                   ------------------------------------------------------------   457
CYP5856A3(29445)Conco1                   ------------------------------------------------------------   386
CYP5856B-fragment9(30415)Conco1          ------------------------------------------------------------   191
CYP5856B-fragment10(35455)Conco1         ------------------------------------------------------------   226
CYP5856B-fragment8(44763)Conco1          ------------------------------------------------------------   226
CYP5856B-fragment11(169617)Conco1        ------------------------------------------------------------   226
CYP5856B-fragment12(27598)Conco1         ------------------------------------------------------------   191
CYP5856B1(167158)Conco1                  ------------------------------------------------------------   457
CYP5856B-fragment2(34058)Conco1          ------------------------------------------------------------   265
CYP5856A-fragment1(26325)Conco1          ------------------------------------------------------------   183
CYP5856B-fragment5(30225)Conco1          ------------------------------------------------------------   191
CYP5854A-fragment4(78559)Conco1          ------------------------------------------------------------   133
CYP5854A-fragment2(6332)Conco1           ------------------------------------------------------------   236
CYP5854A-fragment3(6331)Conco1           ------------------------------------------------------------   219
CYP5854E-fragment3(14082)Conco1          ------------------------------------------------------------   127
CYP5854E-fragment5(9574)Conco1           ------------------------------------------------------------   292
CYP5854E-fragment4(9575)Conco1           ------------------------------------------------------------   131
CYP5854E5(14156)Conco1                   ------------------------------------------------------------   473
CYP5854E-fragment2(42529)Conco1          ------------------------------------------------------------   99
CYP5854D1(9752)Conco1                    ------------------------------------------------------------   487
CYP5854E-fragment13(126715)Conco1        ------------------------------------------------------------   144
CYP5854E8(11612)Conco1                   ------------------------------------------------------------   478
CYP5854E-fragment17(6815)Conco1          ------------------------------------------------------------   242
CYP5854E12(104896)Conco1                 ------------------------------------------------------------   489
CYP5854E15(12851)Conco1                  ------------------------------------------------------------   412
CYP5854E16(80744)Conco1                  ------------------------------------------------------------   421
CYP5854E17(80468)Conco1                  ------------------------------------------------------------   439
CYP5854E13(77675)Conco1                  ------------------------------------------------------------   333
CYP5854E14(12850)Conco1                  ------------------------------------------------------------   481
CYP5854E7(11610)Conco1                   ------------------------------------------------------------   489
CYP5854E-fragment16(8906)Conco1          ------------------------------------------------------------   229
CYP5854A3(78558)Conco1                   ------------------------------------------------------------   316
CYP5854A1(154349)Conco1                  ------------------------------------------------------------   495
CYP5854A2(17189)Conco1                   ------------------------------------------------------------   496
CYP5854A-fragment1(6337)Conco1           ------------------------------------------------------------   52
```

```
CYP5854B1(5669)Conco1                  ------------------------------------------------------------ 493
CYP5854B2(5666)Conco1                  ------------------------------------------------------------ 490
CYP5854B3(78236)Conco1                 ------------------------------------------------------------ 450
CYP5854B(38119)Conco1                  ------------------------------------------------------------ 150
CYP5854C1(140971)Conco1                ------------------------------------------------------------ 399
CYP5854E-fragment15(30483)Conco1       ------------------------------------------------------------ 100
CYP5854E10(11616)Conco1                ------------------------------------------------------------ 379
CYP5854E-fragment14(126716)Conco1      ------------------------------------------------------------ 248
CYP5854E-fragment1(11608)Conco1        ------------------------------------------------------------ 152
CYP5854E6(11609)Conco1                 ------------------------------------------------------------ 465
CYP5854E-fragment6(12533)Conco1        ------------------------------------------------------------ 145
CYP5854E-fragment7(8022)Conco1         ------------------------------------------------------------ 66
CYP5854E9(11613)Conco1                 ------------------------------------------------------------ 446
CYP5854E-fragment12(13308)Conco1       ------------------------------------------------------------ 285
CYP5854E-fragment10(13854)Conco1       ------------------------------------------------------------ 282
CYP5854E-fragment11(13446)Conco1       ------------------------------------------------------------ 139
CYP5854E2(13461)Conco1                 ------------------------------------------------------------ 489
CYP5854E-fragment8(12835)Conco1        PAVPLFTFFRLLLDKRCFRDKIDHYLQSYFNEFGVIRVFTHLGRMDCVYR*--------- 220
CYP5854E4(14118)Conco1                 ------------------------------------------------------------ 489
CYP5854E11(11719)Conco1                ------------------------------------------------------------ 476
CYP5854E1(13460)Conco1                 ------------------------------------------------------------ 405
CYP5854E3(12836)Conco1                 PAVPLLTFLHYILDKRCYRDKVNDYLQGYFNEFGVIRVLTHLGWTVFIADAKICKEVNAL 250
CYP5854E-fragment9(11720)Conco1        ------------------------------------------------------------ 162


CYP5854A-fragment5(6317)Conco1         ------------------------------------------------------------ 126
CYP5856B-fragment6(2022)Conco1         ------------------------------------------------------------ 107
CYP5862A1(43654)Conco1                 ------------------------------------------------------------ 307
CYP5862A-fragment1(25548)Conco1        ------------------------------------------------------------ 234
CYP-fragment(71373)Conco1              ------------------------------------------------------------ 283
CYP5855A3(13529)Conco1                 ------------------------------------------------------------ 366
CYP5855A-fragment1(11397)Conco1        ------------------------------------------------------------ 255
CYP5855A1(44820)Conco1                 ------------------------------------------------------------ 417
CYP5855A2(30387)Conco1                 ------------------------------------------------------------ 386
CYP5855A4(43538)Conco1                 ------------------------------------------------------------ 416
CYP51F14(41890)Conco1                  ------------------------------------------------------------ 334
CYP5859A-fragment2(11509)Conco1        ------------------------------------------------------------ 126
CYP5859A-fragment1(29099)Conco1        ------------------------------------------------------------ 158
CYP5859A-fragment3(168491)Conco1       ------------------------------------------------------------ 107
CYP5859A1(11907)Conco1                 ------------------------------------------------------------ 502
CYP5859A2(11511)Conco1                 ------------------------------------------------------------ 502
CYP5860A3(24597)Conco1                 ------------------------------------------------------------ 438
CYP5860A1(168890)Conco1                ------------------------------------------------------------ 510
CYP5860A2(12313)Conco1                 ------------------------------------------------------------ 510
CYP5863A1(2687)Conco1                  ------------------------------------------------------------ 499
```

```
CYP5857A2(2640)Conco1              ------------------------------------------------------------  484
CYP5857A4(2643)Conco1              ------------------------------------------------------------  316
CYP5857A-fragment1(12733)Conco1    ------------------------------------------------------------  223
CYP5857A3(2641)Conco1              ------------------------------------------------------------  468
CYP5857A7(2648)Conco1              ------------------------------------------------------------  483
CYP5857A6(2646)Conco1              ------------------------------------------------------------  334
CYP5857A-fragment2(24936)Conco1    ------------------------------------------------------------  175
CYP5857A8(2649)Conco1              ------------------------------------------------------------  484
CYP5857A1(14904)Conco1             ------------------------------------------------------------  484
CYP5857A5(2645)Conco1              ------------------------------------------------------------  335
CYP5855B1(4344)Conco1              ------------------------------------------------------------  369
CYP5855B-fragment1(4348)Conco1     ------------------------------------------------------------  250
CYP5864A1(18764)Conco1             ------------------------------------------------------------  476
CYP5855-fragment1(2292)Conco1      ------------------------------------------------------------  97
CYP5855C1(34338)Conco1             ------------------------------------------------------------  496
CYP5855C2(2240)Conco1              ------------------------------------------------------------  475
CYP5855C3(2245)Conco1              ------------------------------------------------------------  489
CYP5855C4(21824)Conco1             ------------------------------------------------------------  439
CYP5855C5(2262)Conco1              ------------------------------------------------------------  375
CYP5855C9(2252)Conco1              ------------------------------------------------------------  478
CYP5855C6(27512)Conco1             ------------------------------------------------------------  380
CYP5855C-fragment3(34299)Conco1    ------------------------------------------------------------  248
CYP5855C-fragment1(34389)Conco1    ------------------------------------------------------------  220
CYP5855C7(34382)Conco1             ------------------------------------------------------------  489
CYP5855C8(2259)Conco1              ------------------------------------------------------------  377
CYP5855D7(2257)Conco1              ------------------------------------------------------------  426
CYP5855D1(29975)Conco1             ------------------------------------------------------------  334
CYP5855D-fragment5(29026)Conco1    ------------------------------------------------------------  200
CYP5855D-fragment7(2254)Conco1     ------------------------------------------------------------  217
CYP5855C-fragment2(30530)Conco1    ------------------------------------------------------------  164
CYP5855E1(38687)Conco1             ------------------------------------------------------------  404
CYP5855D6(6708)Conco1              ------------------------------------------------------------  481
CYP5855D-fragment1(169043)Conco1   ------------------------------------------------------------  149
CYP5855D-fragment6(24158)Conco1    ------------------------------------------------------------  67
CYP5855D4(54364)Conco1             ------------------------------------------------------------  489
CYP5855D5(76752)Conco1             ------------------------------------------------------------  311
CYP5855D-fragment2(2269)Conco1     ------------------------------------------------------------  66
CYP5855D3(54372)Conco1             ------------------------------------------------------------  488
CYP5855D-fragment3(28450)Conco1    ------------------------------------------------------------  120
CYP5855D8(45649)Conco1             ------------------------------------------------------------  350
CYP5855D2(2268)Conco1              ------------------------------------------------------------  478
CYP5855D-fragment4(27931)Conco1    ------------------------------------------------------------  153
CYP5858A-fragment7(4474)Conco1     ------------------------------------------------------------  220
CYP5856B-fragment1(2011)Conco1     ------------------------------------------------------------  203
CYP5858A-fragment1(68816)Conco1    ------------------------------------------------------------  178
```

```
CYP5858A-fragment2(31161)Conco1        ------------------------------------------------------------   167
CYP5858A-fragment5(21198)Conco1        ------------------------------------------------------------   166
CYP5858A-fragment3(23423)Conco1        ------------------------------------------------------------   167
CYP5858A-fragment4(23037)Conco1        ------------------------------------------------------------   164
CYP5858A-fragment6(21524)Conco1        ------------------------------------------------------------   167
CYP5858A-fragment8(27368)Conco1        ------------------------------------------------------------   167
CYP5858A1(68836)Conco1                 ------------------------------------------------------------   500
CYP5858A2(77667)Conco1                 ------------------------------------------------------------   497
CYP5858A3(36631)Conco1                 ------------------------------------------------------------   423
CYP5861A-fragment2(21280)Conco1        ------------------------------------------------------------   151
CYP5861A1(30625)Conco1                 ------------------------------------------------------------   423
CYP5861A-fragment1(36094)Conco1        ------------------------------------------------------------   225
CYP5856A4(2008)Conco1                  ------------------------------------------------------------   492
CYP5856B2(45436)Conco1                 ------------------------------------------------------------   484
CYP5856B-fragment7(34125)Conco1        ------------------------------------------------------------   225
CYP5856B3(76636)Conco1                 ------------------------------------------------------------   457
CYP5856B-fragment3(29023)Conco1        ------------------------------------------------------------   203
CYP5856B-fragment4(76635)Conco1        ------------------------------------------------------------   266
CYP5856A1(34083)Conco1                 ------------------------------------------------------------   492
CYP5856A2(34094)Conco1                 ------------------------------------------------------------   457
CYP5856A3(29445)Conco1                 ------------------------------------------------------------   386
CYP5856B-fragment9(30415)Conco1        ------------------------------------------------------------   191
CYP5856B-fragment10(35455)Conco1       ------------------------------------------------------------   226
CYP5856B-fragment8(44763)Conco1        ------------------------------------------------------------   226
CYP5856B-fragment11(169617)Conco1      ------------------------------------------------------------   226
CYP5856B-fragment12(27598)Conco1       ------------------------------------------------------------   191
CYP5856B1(167158)Conco1                ------------------------------------------------------------   457
CYP5856B-fragment2(34058)Conco1        ------------------------------------------------------------   265
CYP5856A-fragment1(26325)Conco1        ------------------------------------------------------------   183
CYP5856B-fragment5(30225)Conco1        ------------------------------------------------------------   191
CYP5854A-fragment4(78559)Conco1        ------------------------------------------------------------   133
CYP5854A-fragment2(6332)Conco1         ------------------------------------------------------------   236
CYP5854A-fragment3(6331)Conco1         ------------------------------------------------------------   219
CYP5854E-fragment3(14082)Conco1        ------------------------------------------------------------   127
CYP5854E-fragment5(9574)Conco1         ------------------------------------------------------------   292
CYP5854E-fragment4(9575)Conco1         ------------------------------------------------------------   131
CYP5854E5(14156)Conco1                 ------------------------------------------------------------   473
CYP5854E-fragment2(42529)Conco1        ------------------------------------------------------------   99
CYP5854D1(9752)Conco1                  ------------------------------------------------------------   487
CYP5854E-fragment13(126715)Conco1      ------------------------------------------------------------   144
CYP5854E8(11612)Conco1                 ------------------------------------------------------------   478
CYP5854E-fragment17(6815)Conco1        ------------------------------------------------------------   242
CYP5854E12(104896)Conco1               ------------------------------------------------------------   489
CYP5854E15(12851)Conco1                ------------------------------------------------------------   412
CYP5854E16(80744)Conco1                ------------------------------------------------------------   421
```

```
CYP5854E17(80468)Conco1            ------------------------------------------------------------   439
CYP5854E13(77675)Conco1            ------------------------------------------------------------   333
CYP5854E14(12850)Conco1            ------------------------------------------------------------   481
CYP5854E7(11610)Conco1             ------------------------------------------------------------   489
CYP5854E-fragment16(8906)Conco1    ------------------------------------------------------------   229
CYP5854A3(78558)Conco1             ------------------------------------------------------------   316
CYP5854A1(154349)Conco1            ------------------------------------------------------------   495
CYP5854A2(17189)Conco1             ------------------------------------------------------------   496
CYP5854A-fragment1(6337)Conco1     ------------------------------------------------------------   52
CYP5854B1(5669)Conco1              ------------------------------------------------------------   493
CYP5854B2(5666)Conco1              ------------------------------------------------------------   490
CYP5854B3(78236)Conco1             ------------------------------------------------------------   450
CYP5854B(38119)Conco1              ------------------------------------------------------------   150
CYP5854C1(140971)Conco1            ------------------------------------------------------------   399
CYP5854E-fragment15(30483)Conco1   ------------------------------------------------------------   100
CYP5854E10(11616)Conco1            ------------------------------------------------------------   379
CYP5854E-fragment14(126716)Conco1  ------------------------------------------------------------   248
CYP5854E-fragment1(11608)Conco1    ------------------------------------------------------------   152
CYP5854E6(11609)Conco1             ------------------------------------------------------------   465
CYP5854E-fragment6(12533)Conco1    ------------------------------------------------------------   145
CYP5854E-fragment7(8022)Conco1     ------------------------------------------------------------   66
CYP5854E9(11613)Conco1             ------------------------------------------------------------   446
CYP5854E-fragment12(13308)Conco1   ------------------------------------------------------------   285
CYP5854E-fragment10(13854)Conco1   ------------------------------------------------------------   282
CYP5854E-fragment11(13446)Conco1   ------------------------------------------------------------   139
CYP5854E2(13461)Conco1             ------------------------------------------------------------   489
CYP5854E-fragment8(12835)Conco1    ------------------------------------------------------------   220
CYP5854E4(14118)Conco1             ------------------------------------------------------------   489
CYP5854E11(11719)Conco1            ------------------------------------------------------------   476
CYP5854E1(13460)Conco1             ------------------------------------------------------------   405
CYP5854E3(12836)Conco1             SDVFQKSSSSKNSSSKLLRRFIGVSQVAAVNGAEWKKQRKVINPIFNQTWSTELFGNCAQ   310
CYP5854E-fragment9(11720)Conco1    ------------------------------------------------------------   162


CYP5854A-fragment5(6317)Conco1     ------------------------------------------------------------   126
CYP5856B-fragment6(2022)Conco1     ------------------------------------------------------------   107
CYP5862A1(43654)Conco1             ------------------------------------------------------------   307
CYP5862A-fragment1(25548)Conco1    ------------------------------------------------------------   234
CYP-fragment(71373)Conco1          ------------------------------------------------------------   283
CYP5855A3(13529)Conco1             ------------------------------------------------------------   366
CYP5855A-fragment1(11397)Conco1    ------------------------------------------------------------   255
CYP5855A1(44820)Conco1             ------------------------------------------------------------   417
CYP5855A2(30387)Conco1             ------------------------------------------------------------   386
CYP5855A4(43538)Conco1             ------------------------------------------------------------   416
CYP51F14(41890)Conco1              ------------------------------------------------------------   334
```

```
CYP5859A-fragment2(11509)Conco1        ------------------------------------------------------------   126
CYP5859A-fragment1(29099)Conco1        ------------------------------------------------------------   158
CYP5859A-fragment3(168491)Conco1       ------------------------------------------------------------   107
CYP5859A1(11907)Conco1                 ------------------------------------------------------------   502
CYP5859A2(11511)Conco1                 ------------------------------------------------------------   502
CYP5860A3(24597)Conco1                 ------------------------------------------------------------   438
CYP5860A1(168890)Conco1                ------------------------------------------------------------   510
CYP5860A2(12313)Conco1                 ------------------------------------------------------------   510
CYP5863A1(2687)Conco1                  ------------------------------------------------------------   499
CYP5857A2(2640)Conco1                  ------------------------------------------------------------   484
CYP5857A4(2643)Conco1                  ------------------------------------------------------------   316
CYP5857A-fragment1(12733)Conco1        ------------------------------------------------------------   223
CYP5857A3(2641)Conco1                  ------------------------------------------------------------   468
CYP5857A7(2648)Conco1                  ------------------------------------------------------------   483
CYP5857A6(2646)Conco1                  ------------------------------------------------------------   334
CYP5857A-fragment2(24936)Conco1        ------------------------------------------------------------   175
CYP5857A8(2649)Conco1                  ------------------------------------------------------------   484
CYP5857A1(14904)Conco1                 ------------------------------------------------------------   484
CYP5857A5(2645)Conco1                  ------------------------------------------------------------   335
CYP5855B1(4344)Conco1                  ------------------------------------------------------------   369
CYP5855B-fragment1(4348)Conco1         ------------------------------------------------------------   250
CYP5864A1(18764)Conco1                 ------------------------------------------------------------   476
CYP5855-fragment1(2292)Conco1          ------------------------------------------------------------   97
CYP5855C1(34338)Conco1                 ------------------------------------------------------------   496
CYP5855C2(2240)Conco1                  ------------------------------------------------------------   475
CYP5855C3(2245)Conco1                  ------------------------------------------------------------   489
CYP5855C4(21824)Conco1                 ------------------------------------------------------------   439
CYP5855C5(2262)Conco1                  ------------------------------------------------------------   375
CYP5855C9(2252)Conco1                  ------------------------------------------------------------   478
CYP5855C6(27512)Conco1                 ------------------------------------------------------------   380
CYP5855C-fragment3(34299)Conco1        ------------------------------------------------------------   248
CYP5855C-fragment1(34389)Conco1        ------------------------------------------------------------   220
CYP5855C7(34382)Conco1                 ------------------------------------------------------------   489
CYP5855C8(2259)Conco1                  ------------------------------------------------------------   377
CYP5855D7(2257)Conco1                  ------------------------------------------------------------   426
CYP5855D1(29975)Conco1                 ------------------------------------------------------------   334
CYP5855D-fragment5(29026)Conco1        ------------------------------------------------------------   200
CYP5855D-fragment7(2254)Conco1         ------------------------------------------------------------   217
CYP5855C-fragment2(30530)Conco1        ------------------------------------------------------------   164
CYP5855E1(38687)Conco1                 ------------------------------------------------------------   404
CYP5855D6(6708)Conco1                  ------------------------------------------------------------   481
CYP5855D-fragment1(169043)Conco1       ------------------------------------------------------------   149
CYP5855D-fragment6(24158)Conco1        ------------------------------------------------------------   67
CYP5855D4(54364)Conco1                 ------------------------------------------------------------   489
CYP5855D5(76752)Conco1                 ------------------------------------------------------------   311
```

CYP5855D-fragment2(2269)Conco1	--	66
CYP5855D3(54372)Conco1	--	488
CYP5855D-fragment3(28450)Conco1	--	120
CYP5855D8(45649)Conco1	--	350
CYP5855D2(2268)Conco1	--	478
CYP5855D-fragment4(27931)Conco1	--	153
CYP5858A-fragment7(4474)Conco1	--	220
CYP5856B-fragment1(2011)Conco1	--	203
CYP5858A-fragment1(68816)Conco1	--	178
CYP5858A-fragment2(31161)Conco1	--	167
CYP5858A-fragment5(21198)Conco1	--	166
CYP5858A-fragment3(23423)Conco1	--	167
CYP5858A-fragment4(23037)Conco1	--	164
CYP5858A-fragment6(21524)Conco1	--	167
CYP5858A-fragment8(27368)Conco1	--	167
CYP5858A1(68836)Conco1	--	500
CYP5858A2(77667)Conco1	--	497
CYP5858A3(36631)Conco1	--	423
CYP5861A-fragment2(21280)Conco1	--	151
CYP5861A1(30625)Conco1	--	423
CYP5861A-fragment1(36094)Conco1	--	225
CYP5856A4(2008)Conco1	--	492
CYP5856B2(45436)Conco1	--	484
CYP5856B-fragment7(34125)Conco1	--	225
CYP5856B3(76636)Conco1	--	457
CYP5856B-fragment3(29023)Conco1	--	203
CYP5856B-fragment4(76635)Conco1	--	266
CYP5856A1(34083)Conco1	--	492
CYP5856A2(34094)Conco1	--	457
CYP5856A3(29445)Conco1	--	386
CYP5856B-fragment9(30415)Conco1	--	191
CYP5856B-fragment10(35455)Conco1	--	226
CYP5856B-fragment8(44763)Conco1	--	226
CYP5856B-fragment11(169617)Conco1	--	226
CYP5856B-fragment12(27598)Conco1	--	191
CYP5856B1(167158)Conco1	--	457
CYP5856B-fragment2(34058)Conco1	--	265
CYP5856A-fragment1(26325)Conco1	--	183
CYP5856B-fragment5(30225)Conco1	--	191
CYP5854A-fragment4(78559)Conco1	--	133
CYP5854A-fragment2(6332)Conco1	--	236
CYP5854A-fragment3(6331)Conco1	--	219
CYP5854E-fragment3(14082)Conco1	--	127
CYP5854E-fragment5(9574)Conco1	--	292
CYP5854E-fragment4(9575)Conco1	--	131

```
CYP5854E5(14156)Conco1                  ------------------------------------------------------------   473
CYP5854E-fragment2(42529)Conco1         ------------------------------------------------------------   99
CYP5854D1(9752)Conco1                   ------------------------------------------------------------   487
CYP5854E-fragment13(126715)Conco1       ------------------------------------------------------------   144
CYP5854E8(11612)Conco1                  ------------------------------------------------------------   478
CYP5854E-fragment17(6815)Conco1         ------------------------------------------------------------   242
CYP5854E12(104896)Conco1                ------------------------------------------------------------   489
CYP5854E15(12851)Conco1                 ------------------------------------------------------------   412
CYP5854E16(80744)Conco1                 ------------------------------------------------------------   421
CYP5854E17(80468)Conco1                 ------------------------------------------------------------   439
CYP5854E13(77675)Conco1                 ------------------------------------------------------------   333
CYP5854E14(12850)Conco1                 ------------------------------------------------------------   481
CYP5854E7(11610)Conco1                  ------------------------------------------------------------   489
CYP5854E-fragment16(8906)Conco1         ------------------------------------------------------------   229
CYP5854A3(78558)Conco1                  ------------------------------------------------------------   316
CYP5854A1(154349)Conco1                 ------------------------------------------------------------   495
CYP5854A2(17189)Conco1                  ------------------------------------------------------------   496
CYP5854A-fragment1(6337)Conco1          ------------------------------------------------------------   52
CYP5854B1(5669)Conco1                   ------------------------------------------------------------   493
CYP5854B2(5666)Conco1                   ------------------------------------------------------------   490
CYP5854B3(78236)Conco1                  ------------------------------------------------------------   450
CYP5854B(38119)Conco1                   ------------------------------------------------------------   150
CYP5854C1(140971)Conco1                 ------------------------------------------------------------   399
CYP5854E-fragment15(30483)Conco1        ------------------------------------------------------------   100
CYP5854E10(11616)Conco1                 ------------------------------------------------------------   379
CYP5854E-fragment14(126716)Conco1       ------------------------------------------------------------   248
CYP5854E-fragment1(11608)Conco1         ------------------------------------------------------------   152
CYP5854E6(11609)Conco1                  ------------------------------------------------------------   465
CYP5854E-fragment6(12533)Conco1         ------------------------------------------------------------   145
CYP5854E-fragment7(8022)Conco1          ------------------------------------------------------------   66
CYP5854E9(11613)Conco1                  ------------------------------------------------------------   446
CYP5854E-fragment12(13308)Conco1        ------------------------------------------------------------   285
CYP5854E-fragment10(13854)Conco1        ------------------------------------------------------------   282
CYP5854E-fragment11(13446)Conco1        ------------------------------------------------------------   139
CYP5854E2(13461)Conco1                  ------------------------------------------------------------   489
CYP5854E-fragment8(12835)Conco1         ------------------------------------------------------------   220
CYP5854E4(14118)Conco1                  ------------------------------------------------------------   489
CYP5854E11(11719)Conco1                 ------------------------------------------------------------   476
CYP5854E1(13460)Conco1                  ------------------------------------------------------------   405
CYP5854E3(12836)Conco1                  DLIDEWEKMDGKEFKIHDKIKRMTLDVFGKSIFDMEFKSVKNDDSKLYNLYHDIFEELFG   370
CYP5854E-fragment9(11720)Conco1         ------------------------------------------------------------   162


CYP5854A-fragment5(6317)Conco1          ---------------------        126
CYP5856B-fragment6(2022)Conco1          ---------------------        107
```

```
CYP5862A1(43654)Conco1                      ---------------------        307
CYP5862A-fragment1(25548)Conco1             ---------------------        234
CYP-fragment(71373)Conco1                   ---------------------        283
CYP5855A3(13529)Conco1                      ---------------------        366
CYP5855A-fragment1(11397)Conco1             ---------------------        255
CYP5855A1(44820)Conco1                      ---------------------        417
CYP5855A2(30387)Conco1                      ---------------------        386
CYP5855A4(43538)Conco1                      ---------------------        416
CYP51F14(41890)Conco1                       ---------------------        334
CYP5859A-fragment2(11509)Conco1             ---------------------        126
CYP5859A-fragment1(29099)Conco1             ---------------------        158
CYP5859A-fragment3(168491)Conco1            ---------------------        107
CYP5859A1(11907)Conco1                      ---------------------        502
CYP5859A2(11511)Conco1                      ---------------------        502
CYP5860A3(24597)Conco1                      ---------------------        438
CYP5860A1(168890)Conco1                     ---------------------        510
CYP5860A2(12313)Conco1                      ---------------------        510
CYP5863A1(2687)Conco1                       ---------------------        499
CYP5857A2(2640)Conco1                       ---------------------        484
CYP5857A4(2643)Conco1                       ---------------------        316
CYP5857A-fragment1(12733)Conco1             ---------------------        223
CYP5857A3(2641)Conco1                       ---------------------        468
CYP5857A7(2648)Conco1                       ---------------------        483
CYP5857A6(2646)Conco1                       ---------------------        334
CYP5857A-fragment2(24936)Conco1             ---------------------        175
CYP5857A8(2649)Conco1                       ---------------------        484
CYP5857A1(14904)Conco1                      ---------------------        484
CYP5857A5(2645)Conco1                       ---------------------        335
CYP5855B1(4344)Conco1                       ---------------------        369
CYP5855B-fragment1(4348)Conco1              ---------------------        250
CYP5864A1(18764)Conco1                      ---------------------        476
CYP5855-fragment1(2292)Conco1               ---------------------        97
CYP5855C1(34338)Conco1                      ---------------------        496
CYP5855C2(2240)Conco1                       ---------------------        475
CYP5855C3(2245)Conco1                       ---------------------        489
CYP5855C4(21824)Conco1                      ---------------------        439
CYP5855C5(2262)Conco1                       ---------------------        375
CYP5855C9(2252)Conco1                       ---------------------        478
CYP5855C6(27512)Conco1                      ---------------------        380
CYP5855C-fragment3(34299)Conco1             ---------------------        248
CYP5855C-fragment1(34389)Conco1             ---------------------        220
CYP5855C7(34382)Conco1                      ---------------------        489
CYP5855C8(2259)Conco1                       ---------------------        377
CYP5855D7(2257)Conco1                       ---------------------        426
CYP5855D1(29975)Conco1                      ---------------------        334
```

```
CYP5855D-fragment5(29026)Conco1        ---------------------        200
CYP5855D-fragment7(2254)Conco1         ---------------------        217
CYP5855C-fragment2(30530)Conco1        ---------------------        164
CYP5855E1(38687)Conco1                 ---------------------        404
CYP5855D6(6708)Conco1                  ---------------------        481
CYP5855D-fragment1(169043)Conco1       ---------------------        149
CYP5855D-fragment6(24158)Conco1        ---------------------        67
CYP5855D4(54364)Conco1                 ---------------------        489
CYP5855D5(76752)Conco1                 ---------------------        311
CYP5855D-fragment2(2269)Conco1         ---------------------        66
CYP5855D3(54372)Conco1                 ---------------------        488
CYP5855D-fragment3(28450)Conco1        ---------------------        120
CYP5855D8(45649)Conco1                 ---------------------        350
CYP5855D2(2268)Conco1                  ---------------------        478
CYP5855D-fragment4(27931)Conco1        ---------------------        153
CYP5858A-fragment7(4474)Conco1         ---------------------        220
CYP5856B-fragment1(2011)Conco1         ---------------------        203
CYP5858A-fragment1(68816)Conco1        ---------------------        178
CYP5858A-fragment2(31161)Conco1        ---------------------        167
CYP5858A-fragment5(21198)Conco1        ---------------------        166
CYP5858A-fragment3(23423)Conco1        ---------------------        167
CYP5858A-fragment4(23037)Conco1        ---------------------        164
CYP5858A-fragment6(21524)Conco1        ---------------------        167
CYP5858A-fragment8(27368)Conco1        ---------------------        167
CYP5858A1(68836)Conco1                 ---------------------        500
CYP5858A2(77667)Conco1                 ---------------------        497
CYP5858A3(36631)Conco1                 ---------------------        423
CYP5861A-fragment2(21280)Conco1        ---------------------        151
CYP5861A1(30625)Conco1                 ---------------------        423
CYP5861A-fragment1(36094)Conco1        ---------------------        225
CYP5856A4(2008)Conco1                  ---------------------        492
CYP5856B2(45436)Conco1                 ---------------------        484
CYP5856B-fragment7(34125)Conco1        ---------------------        225
CYP5856B3(76636)Conco1                 ---------------------        457
CYP5856B-fragment3(29023)Conco1        ---------------------        203
CYP5856B-fragment4(76635)Conco1        ---------------------        266
CYP5856A1(34083)Conco1                 ---------------------        492
CYP5856A2(34094)Conco1                 ---------------------        457
CYP5856A3(29445)Conco1                 ---------------------        386
CYP5856B-fragment9(30415)Conco1        ---------------------        191
CYP5856B-fragment10(35455)Conco1       ---------------------        226
CYP5856B-fragment8(44763)Conco1        ---------------------        226
CYP5856B-fragment11(169617)Conco1      ---------------------        226
CYP5856B-fragment12(27598)Conco1       ---------------------        191
CYP5856B1(167158)Conco1                ---------------------        457
```

```
CYP5856B-fragment2(34058)Conco1        ---------------------        265
CYP5856A-fragment1(26325)Conco1        ---------------------        183
CYP5856B-fragment5(30225)Conco1        ---------------------        191
CYP5854A-fragment4(78559)Conco1        ---------------------        133
CYP5854A-fragment2(6332)Conco1         ---------------------        236
CYP5854A-fragment3(6331)Conco1         ---------------------        219
CYP5854E-fragment3(14082)Conco1        ---------------------        127
CYP5854E-fragment5(9574)Conco1         ---------------------        292
CYP5854E-fragment4(9575)Conco1         ---------------------        131
CYP5854E5(14156)Conco1                 ---------------------        473
CYP5854E-fragment2(42529)Conco1        ---------------------        99
CYP5854D1(9752)Conco1                  ---------------------        487
CYP5854E-fragment13(126715)Conco1      ---------------------        144
CYP5854E8(11612)Conco1                 ---------------------        478
CYP5854E-fragment17(6815)Conco1        ---------------------        242
CYP5854E12(104896)Conco1               ---------------------        489
CYP5854E15(12851)Conco1                ---------------------        412
CYP5854E16(80744)Conco1                ---------------------        421
CYP5854E17(80468)Conco1                ---------------------        439
CYP5854E13(77675)Conco1                ---------------------        333
CYP5854E14(12850)Conco1                ---------------------        481
CYP5854E7(11610)Conco1                 ---------------------        489
CYP5854E-fragment16(8906)Conco1        ---------------------        229
CYP5854A3(78558)Conco1                 ---------------------        316
CYP5854A1(154349)Conco1                ---------------------        495
CYP5854A2(17189)Conco1                 ---------------------        496
CYP5854A-fragment1(6337)Conco1         ---------------------        52
CYP5854B1(5669)Conco1                  ---------------------        493
CYP5854B2(5666)Conco1                  ---------------------        490
CYP5854B3(78236)Conco1                 ---------------------        450
CYP5854B(38119)Conco1                  ---------------------        150
CYP5854C1(140971)Conco1                ---------------------        399
CYP5854E-fragment15(30483)Conco1       ---------------------        100
CYP5854E10(11616)Conco1                ---------------------        379
CYP5854E-fragment14(126716)Conco1      ---------------------        248
CYP5854E-fragment1(11608)Conco1        ---------------------        152
CYP5854E6(11609)Conco1                 ---------------------        465
CYP5854E-fragment6(12533)Conco1        ---------------------        145
CYP5854E-fragment7(8022)Conco1         ---------------------        66
CYP5854E9(11613)Conco1                 ---------------------        446
CYP5854E-fragment12(13308)Conco1       ---------------------        285
CYP5854E-fragment10(13854)Conco1       ---------------------        282
CYP5854E-fragment11(13446)Conco1       ---------------------        139
CYP5854E2(13461)Conco1                 ---------------------        489
CYP5854E-fragment8(12835)Conco1        ---------------------        220
```

```
CYP5854E4(14118)Conco1                ---------------------        489
CYP5854E11(11719)Conco1               ---------------------        476
CYP5854E1(13460)Conco1                ---------------------        405
CYP5854E3(12836)Conco1                HPIYILFPILENLPFFKRPQL        391
CYP5854E-fragment9(11720)Conco1       ---------------------        162
```

PLEASE NOTE: Showing colors on large alignments is slow.

Table S4. Protein sequences used to create EXXR and CXG motif logos.

CYP5854 family		
**P450**	**EXXR motif sequence**	**CXG motif sequence**
CYP5854A1(154349)Conco1	ESTR	FITGARTCIG
CYP5854A2(17189)Conco1	ESTR	FLTGSRTCIG
CYP5854B1(5669)Conco1	ESMR	FITGPRACIG
CYP5854B2(5666)Conco1	ESMR	FITGPRACIG
CYP5854B3(78236)Conco1	ESMR	FITGPRACIG
CYP5854B(38119)Conco1	ESMR	FLQGPRACNN
CYP5854C1(140971)Conco1	ESMR	FLMGQRSCIG
CYP5854D1(9752)Conco1	ESMR	FFLGTRKCIG
CYP5854E1(13460)Conco1	ESMR	FLYGNRTCLG
CYP5854E2(13461)Conco1	ESMR	FILGNRTCIG
CYP5854E3(12836)Conco1	ESMR	FTLGNRTCIG
CYP5854E4(14118)Conco1	ESMR	FLYGSRTCIG
CYP5854E5(14156)Conco1	ESMR	FGTGPRKCIA
CYP5854E6(11609)Conco1	ESMR	FTLGNRTCLG
CYP5854E7(11610)Conco1	ESMR	FLTGPRSCIG
CYP5854E8(11612)Conco1	ESMR	FATGARSCIG
CYP5854E9(11613)Conco1	ESMR	FGLGNRTCLG
CYP5854E10(11616)Conco1	ESMR	FITGARTCVG
CYP5854E11(11719)Conco1	ESMR	FTLGNRTCLG
CYP5854E12(104896)Conco1	ESMR	FMTGPRSCIG
CYP5854E14(12850)Conco1	ESMR	FSTGPRSCIG
CYP5854E17(80468)Conco1	ESMR	FLTGIRSCIG
CYP5854E-fragment3(14082)Conco1	ELMR	FGNGPRKCIG
CYP5854E-fragment6(12533)Conco1	ESMR	FTLGARICPG
CYP5854E-fragment8(12835)Conco1	ESMR	FISGNRACIG

CYP5854E-fragment9(11720)Conco1	ESMR	FILGNRTCLG
CYP5854E-fragment14(126716)Conco1	ESMR	FISGARSCVG
CYP5854E-fragment16(8906)Conco1	ESMR	FLTGPRSCIG
**CYP5855 family**		
CYP5855A1(44820)Conco1	ESFR	FSSGPRSCIG
CYP5855A2(30387)Conco1	ESLR	FSTGPRGCIG
CYP5855A4(43538)Conco1	ESLR	FSTGPRACIG
CYP5855C1(34338)Conco1	ESMR	FSVGPRSCIG
CYP5855C2(2240)Conco1	ESMR	FSVGPRSCIG
CYP5855C3(2245)Conco1	ESMR	FGTGPRSCIA
CYP5855C4(21824)Conco1	ESMR	FGAGPRSCIA
CYP5855C6(27512)Conco1	ESMR	FGAGPRSCIG
CYP5855C7(34382)Conco1	ESMR	FGAGPRSCIG
CYP5855C9(2252)Conco1	ESMR	FGAGPRSCIG
CYP5855C-fragment1(34389)Conco1	ESMR	FGAGPRSCIG
CYP5855C-fragment2(30530)Conco1	ESMR	FGSGPRSCIG
CYP5855C-fragment3(34299)Conco1	ESMR	FGTGPRSCIG
CYP5855D1(29975)Conco1	ESMR	FGLGPTSCVG
CYP5855D2(2268)Conco1	ESTR	FGLGPRSCIG
CYP5855D3(54372)Conco1	ETMR	FGLGNRSCIG
CYP5855D4(54364)Conco1	EAMR	FGIGTRSCIG
CYP5855D6(6708)Conco1	GAMR	WSTGPRSCIG
CYP5855D7(2257)Conco1	ESMR	WGFGPTSCVG
CYP5855D8(45649)Conco1	ESTR	FGLGPRSCIG
CYP5855D-fragment1(169043)Conco1	EGFR	FGLGPRSCIG
CYP5855D-fragment3(28450)Conco1	ESMR	FGLGTRACVG
CYP5855D-fragment4(27931)Conco1	ESTR	FGLGPRSCIG
CYP5855D-fragment5(29026)Conco1	ESMR	FGIGPTSCIG
CYP5855E1(38687)Conco1	ESMR	FSMGPRGCVG
**CYP5856 family**		
CYP5856A1(34083)Conco1	EVMR	FGGGSRMCVG

CYP5856A2(34094)Conco1	EVMR	FGGGSRMCIG
CYP5856A3(29445)Conco1	EVMR	FGGGSRMCIG
CYP5856A4(2008)Conco1	EAMR	FGGGSRMCLG
CYP5856A-fragment1(26325)Conco1	EVMR	FGGGTRMCVG
CYP5856B1(167158)Conco1	ETMR	FGGGSRMCVG
CYP5856B2(45436)Conco1	ETMR	FGGGSRMCIG
CYP5856B3(76636)Conco1	EVMR	FGGGSRMCVG
CYP5856B-fragment2(34058)Conco1	ETMR	FGGGSRICVG
CYP5856B-fragment3(29023)Conco1	EVMR	FGGGSRMCIG
CYP5856B-fragment4(76635)Conco1	EVMR	FGGGSRMCIG
CYP5856B-fragment5(30225)Conco1	EVMR	FGGGTRMCVG
CYP5856B-fragment7(34125)Conco1	EAMR	FGGGSRMCIG
CYP5856B-fragment8(44763)Conco1	ETMR	FGGGSRMCVG
CYP5856B-fragment9(30415)Conco1	ETMR	FGGGSRMCVG
CYP5856B-fragment10(35455)Conco1	ETMR	FGGGSRMCVG
CYP5856B-fragment11(169617)Conco1	ETMR	FGGGSRMCVG
CYP5856B-fragment12(27598)Conco1	ETMR	FGGGSRMCVG

Table S5. Analysis of P450 tandem duplications in *C. coronatus*. P450 tandem duplications were analyzed by synteny analysis of *C. coronatus* P450s. The genetic location of *C. coronatus* P450s was presented with scaffold number, location on the scaffold with start and end point of the P450 genes and DNA strand.

Scaffold number	P450 name	Protein Id	Location on DNA		
			Start	End	Strand
scaffold_15	CYP5856B-fragment10	35455	166855	167535	(+)
scaffold_15	CYP5856B-fragment9	30415	162859	163431	(+)
scaffold_172	CYP5854E-fragment4	9575	2680	3123	(+)
scaffold_172	CYP5854E-fragment5	9574	1522	2632	(+)
scaffold_2	CYP5856A4	2008	344183	345661	(-)
scaffold_2	CYP5856A3	29445	403320	404477	(+)
scaffold_2	CYP5856B-fragment2	34058	359091	359888	(-)
scaffold_2	CYP5856A2	34094	334573	335946	(-)
scaffold_2	CYP5856B2	45436	319543	324572	(+)
scaffold_2	CYP5856B3	76636	313472	315146	(+)
scaffold_2	CYP5856A-fragment1	26325	331585	332133	(+)
scaffold_2	CYP5856B-fragment3	29023	307016	307624	(+)
scaffold_2	CYP5856B-fragment5	30225	327922	328494	(+)
scaffold_2	CYP5856B-fragment7	34125	317391	318068	(+)
scaffold_2	CYP5856B-fragment1	2011	354869	355578	(-)
scaffold_2	CYP5856B-fragment6	2022	395630	395953	(+)
scaffold_2	CYP5856A1	34083	339825	341303	(+)
scaffold_2	CYP5856B-fragment4	76635	311225	312066	(+)
scaffold_2	CYP5856B1	167158	362574	363947	(-)

scaffold_24	CYP5861A1	30625	151935	153304	(-)
scaffold_24	CYP5861A-fragment1	36094	144653	145330	(-)
scaffold_280	CYP5855A2	30387	20215	21423	(-)
scaffold_280	CYP5855A4	43538	23645	24952	(-)
scaffold_280	CYP5855A-fragment1	11397	8698	9507	(-)
scaffold_289	CYP5859A-fragment1	29099	22464	22986	(-)
scaffold_289	CYP5859A-fragment2	11509	29398	30041	(+)
scaffold_289	CYP5859A2	11511	34727	36433	(+)
scaffold_298	CYP5854E6	11609	8699	10635	(-)
scaffold_298	CYP5854E9	11613	29539	31273	(-)
scaffold_298	CYP5854E7	11610	16651	18528	(+)
scaffold_298	CYP5854E8	11612	24493	26476	(-)
scaffold_298	CYP5854E10	11616	35983	37566	(+)
scaffold_298	CYP5854E-fragment14	126716	21048	22064	(-)
scaffold_298	CYP5854E-fragment13	126715	22065	22938	(-)
scaffold_298	CYP5854E17	80468	322	2297	(-)
scaffold_298	CYP5854E-fragment1	11608	5682	6289	(+)
scaffold_30	CYP5855C-fragment2	30530	12294	12839	(+)
scaffold_30	CYP5855B-fragment1	4348	18426	19178	(-)
scaffold_30	CYP5855B1	4344	1015	2121	(+)
scaffold_308	CYP5854E11	11719	28806	30734	(+)
scaffold_308	CYP5854E-fragment9	11720	33492	34031	(+)
scaffold_31	CYP5858A2	77667	126992	128567	(+)
scaffold_31	CYP5858A-fragment5	21198	147157	147654	(+)

scaffold_31	CYP5858A-fragment6	21524	164719	165219	(-)
scaffold_31	CYP5858A-fragment4	23037	160773	161264	(-)
scaffold_31	CYP5858A-fragment3	23423	141899	142399	(+)
scaffold_31	CYP5858A-fragment8	27368	159659	160159	(+)
scaffold_31	CYP5858A-fragment2	31161	83255	83755	(+)
scaffold_31	CYP5858A-fragment1	68816	82409	83051	(+)
scaffold_31	CYP5858A-fragment7	4474	179089	180148	(-)
scaffold_31	CYP5858A3	36631	137231	138502	(-)
scaffold_31	CYP5858A1	68836	135169	136671	(-)
scaffold_328	CYP5859A1	11907	449	2153	(-)
scaffold_328	CYP5859A-fragment3	168491	43	375	(+)
scaffold_373	CYP5860A2	12313	232	1963	(-)
scaffold_373	CYP5860A1	168890	21223	22954	(-)
scaffold_4	CYP5855C2	2240	202018	203501	(+)
scaffold_4	CYP5855C3	2245	223177	224691	(-)
scaffold_4	CYP5855D7	2257	262229	263560	(-)
scaffold_4	CYP5855C8	2259	273165	274326	(-)
scaffold_4	CYP5855C5	2262	284153	285308	(-)
scaffold_4	CYP5855D2	2268	309864	311349	(+)
scaffold_4	CYP5855C4	21824	228620	229982	(+)
scaffold_4	CYP5855C6	27512	239726	244424	(-)
scaffold_4	CYP5855D1	29975	254455	255515	(-)
scaffold_4	CYP5855C1	34338	193278	198516	(+)
scaffold_4	CYP5855C7	34382	231614	236387	(-)
scaffold_4	CYP5855D8	45649	330070	331388	(+)
scaffold_4	CYP5855D3	54372	304093	305693	(+)
scaffold_4	CYP5855D5	76752	294579	296037	(-)

scaffold_4	CYP5855D-fragment2	2269	319856	320056	(-)
scaffold_4	CYP5855D-fragment6	24158	294607	294807	(-)
scaffold_4	CYP5855D-fragment4	27931	301481	301989	(+)
scaffold_4	CYP5855D-fragment5	29026	257788	258438	(+)
scaffold_4	CYP5855C-fragment3	34299	204790	205580	(-)
scaffold_4	CYP5855C-fragment1	34389	270322	271029	(+)
scaffold_4	CYP5855D-fragment1	169043	287093	287595	(-)
scaffold_4	CYP5855-fragment1	2292	389767	390122	(+)
scaffold_4	CYP5855C9	2252	249780	251300	(+)
scaffold_4	CYP5855D4	54364	260007	261526	(+)
scaffold_4	CYP5855D-fragment7	2254	257030	257683	(+)
scaffold_442	CYP5854E-fragment8	12835	8787	12510	(-)
scaffold_442	CYP5854E3	12836	13222	17456	(-)
scaffold_445	CYP5854E14	12850	4183	6048	(+)
scaffold_445	CYP5854E15	12851	8693	10293	(+)
scaffold_445	CYP5854E16	80744	18781	20419	(+)
scaffold_54	CYP5854B2	5666	11523	13383	(-)
scaffold_54	CYP5854B1	5669	19377	21738	(-)
scaffold_54	CYP5854B3	78236	15050	16933	(+)
scaffold_54	CYP5854B	38119	29157	29660	(+)
scaffold_574	CYP5854E2	13461	8474	10389	(+)
scaffold_574	CYP5854E1	13460	3600	5106	(+)
scaffold_594	CYP5855A1	44820	328	1633	(-)
scaffold_594	CYP5855A3	13529	4219	5371	(-)

scaffold_69	CYP5854A2	17189	45136	47026	(+)
scaffold_69	CYP5854A-fragment3	6331	34362	35318	(-)
scaffold_69	CYP5854A-fragment2	6332	38051	39000	(-)
scaffold_69	CYP5854A3	78558	29781	30897	(-)
scaffold_69	CYP5854A1	154349	78494	80537	(-)
scaffold_69	CYP5854A-fragment5	6317	2923	3657	(+)
scaffold_69	CYP5854A-fragment1	6337	72925	73083	(-)
scaffold_69	CYP5854A-fragment4	78559	30939	31583	(-)
scaffold_8	CYP5857A2	2640	38973	40633	(-)
scaffold_8	CYP5857A7	2648	68397	70051	(-)
scaffold_8	CYP5857A8	2649	73378	75031	(-)
scaffold_8	CYP5863A1	2687	206112	207611	(-)
scaffold_8	CYP5857A3	2641	44202	45860	(-)
scaffold_8	CYP5857A4	2643	53940	55043	(-)
scaffold_8	CYP5857A5	2645	58779	59839	(-)
scaffold_8	CYP5857A6	2646	63516	64579	(-)
scaffold_8	CYP5857A1	14904	49924	51587	(-)
scaffold_8	CYP5857A-fragment2	24936	189632	194300	(-)

Table S6. Comparative analysis of P450 diversity percentage among entomopathogenic fungi. The P450 diversity percentage in different fungal species was calculated following the method described elsewhere [49, 51].

**Species**	**Number of P450s**	**Number of P450 families**	**P450 diversity percentage**
Conidiobolus coronatus	142	12	8
Beauveria bassiana	83	49	59
Cordyceps militaris	57	37	65
Metarhizium acridum	100	67	67
Metarhizium anisopliae	123	87	71

Figure S1. Phylogenetic analysis of *C. coronatus* P450s. Bootstrap replications used to evaluate each node of the phylogenetic tree are presented in the phylogenetic tree.

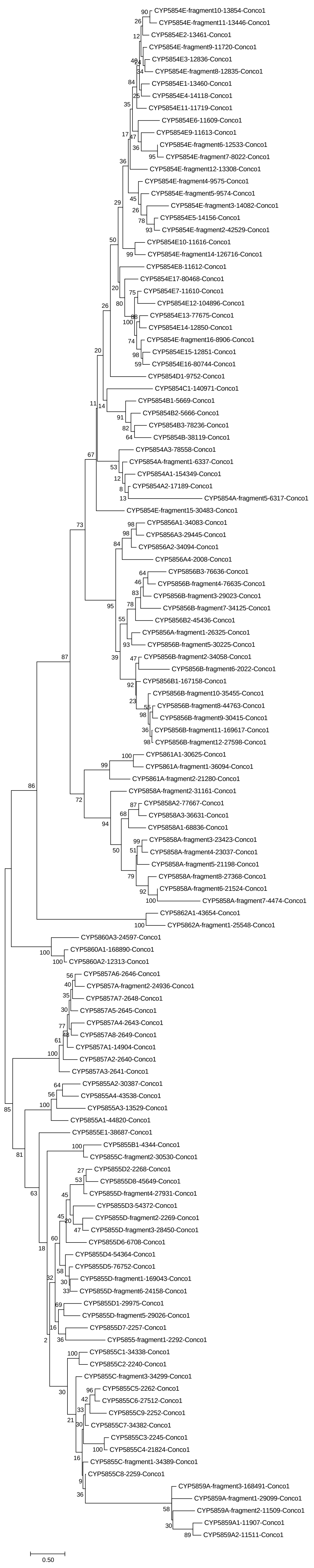

CYP5854E-fragment10-13854-Conco1
CYP5854E-fragment11-13446-Conco1
CYP5854E2-13461-Conco1
CYP5854E-fragment9-11720-Conco1
CYP5854E3-12836-Conco1
CYP5854E-fragment8-12835-Conco1
CYP5854E1-13460-Conco1
CYP5854E4-14118-Conco1
CYP5854E11-11719-Conco1
CYP5854E6-11609-Conco1
CYP5854E9-11613-Conco1
CYP5854E-fragment6-12533-Conco1
CYP5854E-fragment7-8022-Conco1
CYP5854E-fragment12-13308-Conco1
CYP5854E-fragment4-9575-Conco1
CYP5854E-fragment5-9574-Conco1
CYP5854E-fragment3-14082-Conco1
CYP5854E5-14156-Conco1
CYP5854E-fragment2-42529-Conco1
CYP5854E10-11616-Conco1
CYP5854E-fragment14-126716-Conco1
CYP5854E8-11612-Conco1
CYP5854E17-80468-Conco1
CYP5854E7-11610-Conco1
CYP5854E12-104896-Conco1
CYP5854E13-77675-Conco1
CYP5854E14-12850-Conco1
CYP5854E-fragment16-8906-Conco1
CYP5854E15-12851-Conco1
CYP5854E16-80744-Conco1
CYP5854D1-9752-Conco1
CYP5854C1-140971-Conco1
CYP5854B1-5669-Conco1
CYP5854B2-5666-Conco1
CYP5854B3-78236-Conco1
CYP5854B-38119-Conco1
CYP5854A3-78558-Conco1
CYP5854A-fragment1-6337-Conco1
CYP5854A1-154349-Conco1
CYP5854A2-17189-Conco1
CYP5854A-fragment5-6317-Conco1
CYP5854E-fragment15-30483-Conco1
CYP5856A1-34083-Conco1
CYP5856A3-29445-Conco1
CYP5856A2-34094-Conco1
CYP5856A4-2008-Conco1
CYP5856B3-76636-Conco1
CYP5856B-fragment4-76635-Conco1
CYP5856B-fragment3-29023-Conco1
CYP5856B-fragment7-34125-Conco1
CYP5856B2-45436-Conco1
CYP5856A-fragment1-26325-Conco1
CYP5856B-fragment5-30225-Conco1
CYP5856B-fragment2-34058-Conco1
CYP5856B-fragment6-2022-Conco1
CYP5856B1-167158-Conco1
CYP5856B-fragment10-35455-Conco1
CYP5856B-fragment8-44763-Conco1
CYP5856B-fragment9-30415-Conco1
CYP5856B-fragment11-169617-Conco1
CYP5856B-fragment12-27598-Conco1
CYP5861A1-30625-Conco1
CYP5861A-fragment1-36094-Conco1
CYP5861A-fragment2-21280-Conco1
CYP5858A-fragment2-31161-Conco1
CYP5858A2-77667-Conco1
CYP5858A3-36631-Conco1
CYP5858A1-68836-Conco1
CYP5858A-fragment3-23423-Conco1
CYP5858A-fragment4-23037-Conco1
CYP5858A-fragment5-21198-Conco1
CYP5858A-fragment8-27368-Conco1
CYP5858A-fragment6-21524-Conco1
CYP5858A-fragment7-4474-Conco1
CYP5862A1-43654-Conco1
CYP5862A-fragment1-25548-Conco1
CYP5860A3-24597-Conco1
CYP5860A1-168890-Conco1
CYP5860A2-12313-Conco1
CYP5857A6-2646-Conco1
CYP5857A-fragment2-24936-Conco1
CYP5857A7-2648-Conco1
CYP5857A5-2645-Conco1
CYP5857A4-2643-Conco1
CYP5857A8-2649-Conco1
CYP5857A1-14904-Conco1
CYP5857A2-2640-Conco1
CYP5857A3-2641-Conco1
CYP5855A2-30387-Conco1
CYP5855A4-43538-Conco1
CYP5855A3-13529-Conco1
CYP5855A1-44820-Conco1
CYP5855E1-38687-Conco1
CYP5855B1-4344-Conco1
CYP5855C-fragment2-30530-Conco1
CYP5855D2-2268-Conco1
CYP5855D8-45649-Conco1
CYP5855D-fragment4-27931-Conco1
CYP5855D3-54372-Conco1
CYP5855D-fragment2-2269-Conco1
CYP5855D-fragment3-28450-Conco1
CYP5855D6-6708-Conco1
CYP5855D4-54364-Conco1
CYP5855D5-76752-Conco1
CYP5855D-fragment1-169043-Conco1
CYP5855D-fragment6-24158-Conco1
CYP5855D1-29975-Conco1
CYP5855D-fragment5-29026-Conco1
CYP5855D7-2257-Conco1
CYP5855-fragment1-2292-Conco1
CYP5855C1-34338-Conco1
CYP5855C2-2240-Conco1
CYP5855C-fragment3-34299-Conco1
CYP5855C5-2262-Conco1
CYP5855C6-27512-Conco1
CYP5855C9-2252-Conco1
CYP5855C7-34382-Conco1
CYP5855C3-2245-Conco1
CYP5855C4-21824-Conco1
CYP5855C-fragment1-34389-Conco1
CYP5855C8-2259-Conco1
CYP5859A-fragment3-168491-Conco1
CYP5859A-fragment1-29099-Conco1
CYP5859A-fragment2-11509-Conco1
CYP5859A1-11907-Conco1
CYP5859A2-11511-Conco1
0.50
